# A comprehensive review of *Olea europaea* L. metabolites: from structure with confidence scoring to source profiling with biological relevance for therapeutic exploration

**DOI:** 10.1039/d6ra00708b

**Published:** 2026-07-07

**Authors:** Yasmin Mounir Mohamaden, Seham S. El-Hawary, Samar M. Bassam, Amira Safwat El Senousy, Mohamed El Raey

**Affiliations:** a Department of Pharmacognosy, Faculty of Pharmacy, Cairo University Kasr El Aini 11562 Cairo Egypt yasmin.m.mohamaden@pharma.cu.edu.eg yasmin.m0unir.nm@gmail.com seham.elhawary@pharma.cu.edu.eg; b Department of Pharmacognosy, College of Pharmacy, Arab Academy for Science, Technology and Maritime Transport (AASTMT) Alexandria Egypt yasmin.mounir@aast.edu; c Department of Pharmacognosy and Natural Products, Faculty of Pharmacy, Pharos University in Alexandria Canal El Mahmoudia street, Beside Green Plaza Complex 21648 Alexandria Egypt; d Department of Phytochemistry and Plant Systematics, Pharmaceutical Division, National Research Centre Dokki 12622 Cairo Egypt

## Abstract

*Olea europaea* L. (olive tree) stands as an enduring emblem of Mediterranean culture and a treasure trove of structurally diverse phytochemicals with profound biological potential. This review presents a structured, data-driven overview of over 300 metabolites previously reported across various olive sources, including leaves, fruits, olive oil, pomace, flowers, *etc.* These compounds are systematically classified into 15 chemical classes, each described through its basic skeleton and position numbering, highlighting structural features that allow clear differentiation between closely related compounds. Secoiridoids emerge as the dominant class, with oleuropein and ligustroside serving as key representatives, and biosynthetic intermediates such as tyrosol, hydroxytyrosol, and elenolic acid playing central roles. The distribution of metabolites across organs revealed that leaves represent the most abundant source, followed by fruits and olive oil, while pomace also contained a considerable amount, emphasizing its potential value as a by-product for future exploitation. For each metabolite, comprehensive chemical identifiers (PubChem ID, SMILES, InChI, formula, and exact mass) and, for the first time, an analytical confidence level (validated *via* a five-level scoring system) are provided in the SI. This analysis reveals a predominance of moderately validated compounds (Level 3), highlighting a critical need for further structural elucidation. Collectively, this robust and cheminformatics-ready dataset serves as an accessible resource, poised to accelerate future studies in virtual screening, molecular docking, and network pharmacology. It also critically guides efforts to expand structural validation for low-confidence compounds and encourages exploration of underrepresented organs, thereby significantly enriching the landscape of therapeutic mapping. Additionally, this review integrates recent insights into isolation yields, impurity profiles, and toxicological aspects of *Olea europaea* metabolites, thereby providing a holistic framework for their therapeutic evaluation and safe exploitation.

## Abstract 2

*Olea europaea* L. (olive tree) is a rich source of structurally diverse metabolites. The graphical abstract highlights their distribution across plant parts, chemical classes, confidence levels of identification, and potential applications in pharmacology and drug discovery.

## Graphical abstract

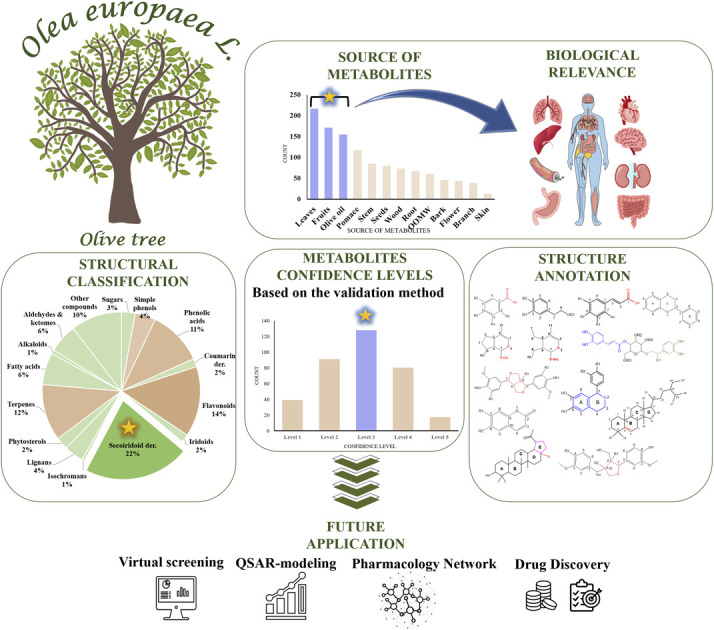

## 1. Introduction

The Oleaceae family encompasses around 30 genera and approximately 500–600 species, predominantly distributed across temperate and tropical regions of Asia and Malaysia.^1^ Within this family, the genus *Olea*, derived from the Latin “oleum” (oil) and the Greek “elaia” (olive), is subdivided into three subgenera: *Tetrapilus*, *Paniculatae*, and *Olea*.^2,3^ Among these, *Olea europaea* L. stands out as the most extensively cultivated species, particularly in the Mediterranean Basin, where its agricultural and cultural significance dates back to antiquity.^4^ Over time, its cultivation expanded globally, including South Africa, Australia, China, and Japan.^5^ This global expansion underscores a growing interest in its diverse array of phytochemicals, which contribute significantly to its acclaimed health benefits.

Beyond its botanical significance, the olive tree holds deep cultural and economic importance. It occupies over eight million hectares across Mediterranean landscapes, with Spain leading global production at approximately 9.8 million tons annually, and Tunisia ranking fourth.^2,6^ Symbolizing peace and triumph, the olive tree has been historically revered, from its presence in ancient Olympic rituals^7^ to its mention in religious scriptures such as the Quran and the Bible.^1,8^

Phytochemically, *Olea europaea* is a rich source of diverse metabolites, including sugars, phenolic acids, secoiridoids, flavonoids, lignans, phytosterols, tocopherols, and fatty acids, *etc.*.^2,9^ These constituents contribute to the health-promoting properties of olive-derived products, particularly olive oil, which is uniquely extracted from the fruit rather than the seeds (*e.g.*, sunflower), setting it apart from conventional edible oils.^10,11^ With total phenolic content ranging from 800 mg kg^−1^ to 1 g kg^−1^, olive oil's nutritional profile is both distinctive and significant.^12^

The identification of phytoconstituents in *Olea europaea* presents challenges due to the structural similarities of many compounds, such as oleoside *vs.* secologanin, oleuropein *vs.* ligustroside, and oleocanthal *vs.* oleacein, as well as oleuricine A *vs.* B.^13^ Various analytical techniques, including TLC, HPLC, RP/HPLC-QTOF-MS, and GC/MS, have been employed to resolve these complexities.^14,15^ However, extensive research and diverse analytical approaches, no existing review offers a unified, structured, and cheminformatics-ready compilation of *Olea europaea* phytoconstituents. This absence limits comprehensive large-scale computational analyses and targeted drug discovery efforts.

This review offers a structured, data-driven, and unprecedented compilation of olive metabolites, addressing a key gap by primarily focusing on their chemical diversity and phytochemical composition in *Olea europaea* L. It highlights the chemotaxonomic significance and biological relevance across predominant sources. The generated dataset is organized by chemical class, mapped to specific plant organs, and enriched with cheminformatics-ready identifiers (InChI, SMILES, PubChem ID, CAS numbers, and ChEMBL), alongside a novel five-confidence-level scoring system based on the validation methods reported in the original references.

By integrating phytochemistry, biological relevance, and cheminformatic accessibility, this review provides a structured and computationally compatible platform. This platform is designed to support drug discovery, functional food innovation, and systems-level exploration of olive-based therapeutics. It serves as both a reliable reference and a practical resource, facilitating *in silico* workflows, such as molecular docking and network pharmacology, and guiding subsequent *in vitro* and *in vivo* investigations, thereby significantly reducing both cost and environmental impact. This work also emphasizes the necessity for future research to focus on structural validation of low-confidence compounds and systematically investigate underrepresented plant organs in order to further enhance and optimize therapeutic mapping.

## 2. Taxonomic classification of *Olea europaea* L

The hierarchical taxonomic classification of *Olea europaea* L. is presented below from kingdom to species^16^

Kingdom: Plantae

Subkingdom: Viridiplantae

Infrakingdom: Streptophyta

Superdivision: Embryophyta

Division: Tracheophyta

Subdivision: Spermatophytina

Class: Magnoliopsida

Superorder: Asteranae

Order: Lamiales

Family: Oleaceae

Genus: *Olea* L

Species: *Olea europaea* L. (olive)

## 3. Literature mining and compound curation

This review combines a traditional literature review with systematic data compilation from diverse scientific resources, including Google Scholar, PubMed, Web of Science, Dictionary of Natural Products (DNP), KEGG COMPOUND Databases, and specialized platforms such as PubChem, FooDB, Phenol-Explorer, ChemSpider, and J-GLOBAL. Our search covered an extensive publication timeline, from 1961 to 2025, employing curated combinations of keywords such as “*Olea europaea*”, “olive metabolites”, “phytochemicals”, “secondary metabolites”, “chemical composition”, “bioactive compounds”, “polyphenols”, “iridoids”, “secoiridoids”, “lignans”, “flavonoids”, “fatty acids”, “Biology”, “olive leaves”, “olive fruit”, and “olive oil” to comprehensively retrieve data on *Olea europaea* L. phytoconstituents.

For each compound, we observed molecular descriptors (*e.g.*, exact mass, calculated *via* an Exact Mass Calculator), alongside structural identifiers (InChI, SMILES), and unique database IDs (CID, CAS Number, ChEMBL ID). Chemical structures were drawn using ChemDraw Professional 15.0. To enhance data visualization and ensure accessibility, compound distributions and classification charts were generated using Microsoft Excel (Version 2108 Build 14334.20296). Graphical abstract and schematic illustrations were designed using Canva and PowerPoint (same version) to support clarity and editorial presentation.

## 4. Comprehensive phytochemical inventory of *Olea europaea* L. Metabolites

### 4.1. Overview of the compiled metabolite dataset

**Fig. 1:**
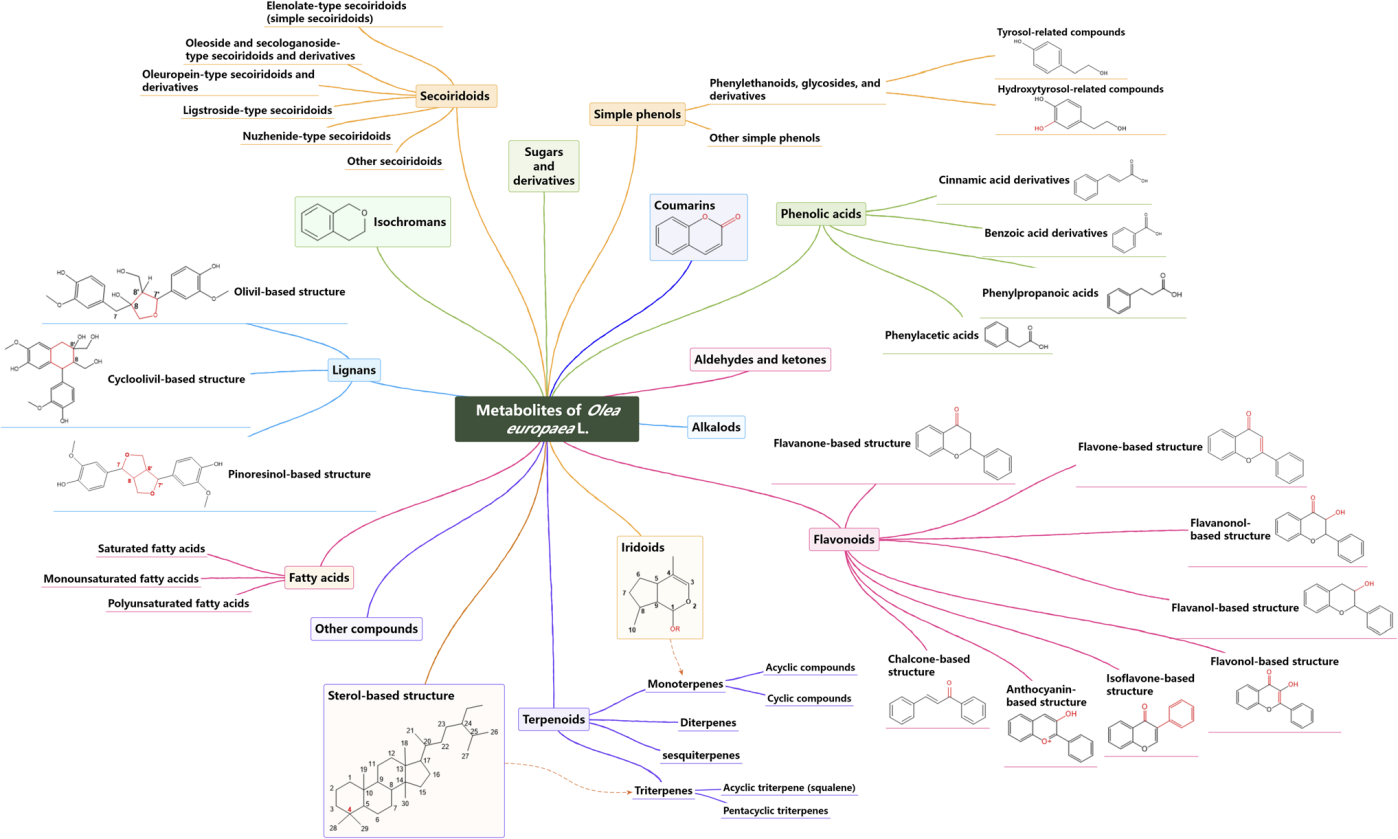
Overview of metabolite classes reported in *Olea europaea* L. according to core structural skeletons.

To enhance visual comprehension of the dataset structure, Fig. 1 presents an overview of metabolite classes in *Olea europaea* L. based on their core skeletons, generated using Nice Mind compiled by SI Table S1, which summarizes 355 compounds across 15 chemical classes, offering a quick comparative insight into their relative abundance within the compiled inventory. This table captures key chemical and structural details for each compound, including its molecular formula, exact mass, and identifiers such as InChI, SMILES, CID, CAS number, and ChEMBL ID. It also documents where each metabolite is found within the plant, including the leaves, fruits, seeds, flowers, bark, stem, and other parts. In addition, Confidence levels (levels 1–5) were assigned to the identified compounds based on the method of validation reported in previous studies, ranging from highest to lowest certainty: (level 1) isolation and full structural elucidation, including 2D NMR; (level 2) co-chromatography with a chemical or botanical standard, considering spectroscopic/spectrometric data (UV, HRMS, MS/MS); (level 3) chromatography with Rt and UV/HRMS/MS/MS data supported by comparison of both chromatographic and spectroscopic parameters with literature reports; (level 4) chromatography with UV/HRMS/MS/MS detector only; and (level 5) no supporting data or comments provided, in which case the compound was rejected.

### 4.2. Chemical class distribution and metabolite occurrence across olive sources

**Fig. 2:**
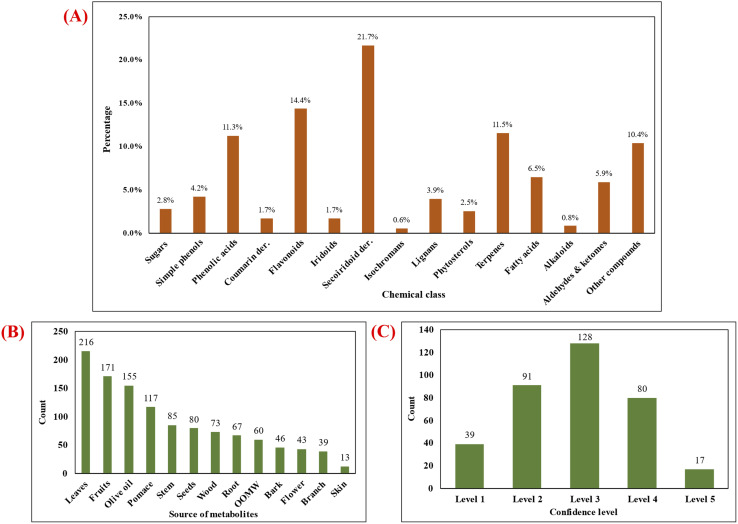
Distribution of metabolites in *Olea europaea* L.: (A) percentage of different chemical classes, (B) occurrence across the source of metabolites, and (C) confidence levels (1–5) based on analytical validation.

Fig. 2A vividly illustrates the diverse chemical landscape of over 300 metabolites identified across various organs of *Olea europaea* L. Notably, secoiridoids stand out as the predominant chemical class, accounting for a significant 21.7% of all detected phytochemicals. These compounds are particularly characteristic of olives and are largely responsible for their distinctive pharmacological and nutritional properties. Following closely, major classes include flavonoids (14.4%), phenolic acids (11.3%), and terpenes (11.5%), all of which contribute substantially to the plant's well-documented antioxidant, anti-inflammatory, and cardioprotective properties. Beyond these, a range of other compounds like fatty acids (6.5%), aldehydes and ketones (5.9%), simple phenols (4.2%), lignans (3.9%), and phytosterols (2.5%) are present at moderate to lower levels, yet collectively offer further pharmacological relevance. Even minor classes, such as isochromans (0.6%) and alkaloids (0.8%), underscore the remarkable structural diversity inherent in olive metabolites.

As illustrated in Fig. 2B, the distribution of metabolites across the olive sources reveals intriguing insights. Olive leaves had the richest source, boasting the highest number of identified metabolites (216), followed by fruits (171), and olive oil (155). This abundance underscores their crucial roles in both traditional uses and industrial applications. Moving down, pomace, a valuable solid residue comprising skin, pulp, stone, and kernel, contributes a substantial 117 compounds. Given that pomace constitutes around 35–40% of the olive milling process,^17^ its rich metabolite profile highlights considerable potential for sustainable valorization and medicinal uses. Furthermore, while organs such as stems (85), seeds (80), wood (73), and roots (67) yield a moderate number of compounds, sources like bark (46), flowers (43), branches (39), and skin (13) exhibit a smaller yet noteworthy count. This varied distribution not only emphasizes the need for more in-depth research into these less-explored sources but also points towards promising avenues for novel metabolite discovery.

### 4.3. Confidence levels in metabolite identification and computational implications

In Fig. 2C, the reliability of compound identification was assessed through a tiered confidence-level system based on analytical validation methods. A significant majority of metabolites, precisely 128 compounds, were confidently assigned to Level 3. This level primarily signifies identifications based on robust chromatographic and spectrometric data, further supported by thorough literature comparisons. A substantial number of compounds also fell into Level 2 (91 compounds) and Level 4 (80 compounds), reflecting identifications achieved either through co-chromatography with authentic standards or *via* advanced detection techniques such as UV, HRMS, and MS/MS, respectively.

Strikingly, only 39 compounds achieved the highest confidence, Level 1, indicating full structural elucidation often relies on methods such as 2D NMR. This low number highlights a notable scarcity of unequivocally characterized olive metabolites in existing literature. Conversely, 17 compounds were categorized under Level 5, due to insufficient or entirely missing analytical data, thus rendering their identification unreliable for definitive claims.

This unique unimodal distribution, skewed predominantly towards Level 3 in Fig. 2C, strongly suggests a widespread reliance on MS-based analytical techniques within olive phytochemical research. This reveals a critical gap in metabolite validation and underscores the need for more rigorous confirmatory methods, such as NMR spectroscopy or the synthesis of reference standards, to significantly enhance annotation and strengthen the evidence base.

Despite these limitations, the prevalence of Level 2 and 3 identifications actually provides a practical and robust foundation for various computational applications. These tiers offer sufficient structural reliability for initial virtual screening, where even approximate molecular features are often adequate for docking simulations and target prediction. Furthermore, the breadth of annotated compounds across these confidence levels supports network pharmacology approaches. This enables the construction of compound-target-pathway maps, thereby providing plausible insights into complex biological interactions. By integrating cheminformatics-ready identifiers with graded experimental validation, the dataset bridges the gap between laboratory annotation and *in silico* modeling, thus facilitating both innovative hypothesis generation and informed therapeutic prioritization.

## 5. Structural diversity of *Olea europaea* L. metabolite classes

### 5.1. Sugars

**Fig. 3:**
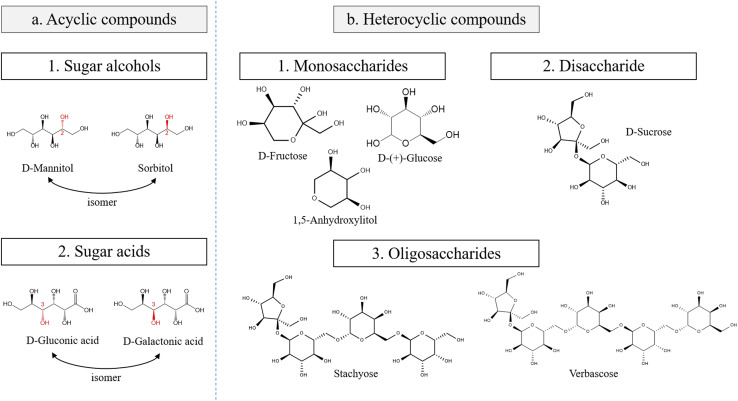
Structural classification of carbohydrates: (a) acyclic compounds, including sugar alcohols and sugar acids; (b) heterocyclic compounds, encompassing representative monosaccharides, disaccharides, and oligosaccharides in *Olea europaea* L.

All sugars in *Olea europaea* have been identified across olive fruits, leaves, and stems.^1^ This review classifies sugars into two main groups based on their molecular framework, as outlined in FOODB: acyclic and heterocyclic compounds. Acyclic compounds, a subset of monosaccharides, are further divided into sugar alcohols, including d-mannitol and d-sorbitol, both six-carbon sugars,^2^ and sugar acids, such as d-gluconic and d-galactonic acids, which are six-carbon structural isomers differing in the stereochemical configuration of the hydroxyl group at C-3 Fig. 3.^18^ On the other hand, heterocyclic compounds, classified according to,^19^ include monosaccharides such as d-fructose, d-(+)-glucose, and 1,5-anhydroxylitol,^20–22^ disaccharide like d-sucrose, and oligosaccharides consisting of 3–10 monosaccharide units, such as stachyose and d-verbascose.^18^

### 5.2. Simple phenols

#### 5.2.1 Phenylethanoids, glycosides, and derivatives

**Fig. 4:**
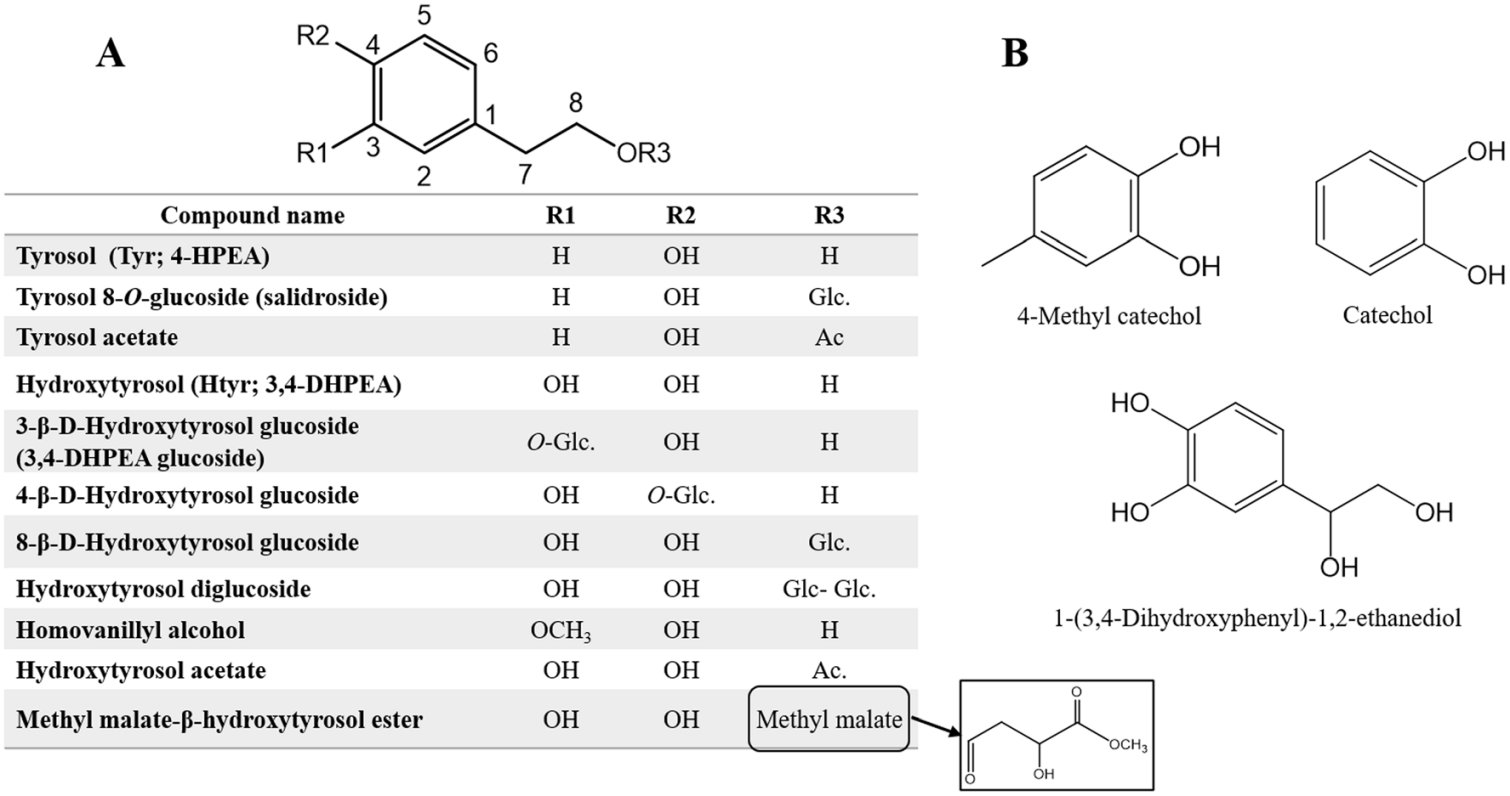
Classification of simple phenolic compounds in *Olea europaea* L.: phenylethanoids (A) and other simple phenols (B).

Phenylethanoids, a notable class of simple phenols, possess a C_6_–C_2_ skeleton^23^ and represent the predominant biophenols in *O. europaea*. They consist of one or more hydroxyl groups attached to an aromatic ring, which may be substituted with or without a side chain of up to three carbon atoms, forming a C_6_–C_0–3_ backbone with position numbering as described in,^24^Fig. 4. The main phenylethanoid compounds that were isolated and identified in olive tree are tyrosol (Tyr), also known as *ρ*-hydroxyphenyl ethanol (*ρ*-HPEA), and hydroxytyrosol (HTyr; 3,4-DHPEA). These molecules serve as precursors to several secoiridoid-related compounds, such as ligustroside and oleuropein, as will be discussed in detail later in this review. While these phenylethanoids are less abundant in other *Olea* species, they are recognized as key contributors to the health benefits associated with olive fruit and oil, including antitumor, anti-inflammatory, antioxidant, antibacterial, antiviral, neuroprotective, and hepatoprotective effects.^25–27^

Simple phenols derived from tyrosol as a precursor include tyrosol 8-*O*-glucoside (salidroside),^28^ tyrosol acetate,^1^ and hydroxytyrosol. Hydroxytyrosol (HTyr; 3,4-DHPEA) is a major simple phenolic compound in olive leaf extract and serves as a precursor to various secoiridoid derivatives in *O. europaea,* such as oleuropein and its derivatives, that have been identified as hydroxytyrosol-related compounds.^2^ Among its glycosylated derivatives, HTyr glucoside (3,4-DHPEA glucoside) exists in three possible isomeric forms: 3-glucoside, 4-glucoside, and 8-glucoside.^29,30^ Research indicates that 8-glucoside is predominant in olive leaves, whereas 4-glucoside is a major component in olive mill wastewater and olive fruit.^31^ Other notable derivatives include HTyr diglucoside, homovanillyl alcohol, HTyr acetate, and methyl malate-β-hydroxytyrosol ester.^18,32,33^

#### 5.2.2 Other simple phenols

Catechol (benzene-1,2-diol),^34^ 4-methyl catechol,^35^ and 1-(3,4-dihydroxyphenyl)-1,2-ethanediol^36^ that are structurally unrelated to Tyr or HTyr, as illustrated in Fig. 4.

### 5.3. Phenolic acids

Phenolic acids are a class of aromatic compounds widely distributed in *O. europaea*, characterized by a carboxyl functional group attached to a phenol ring.^23,37^ This review categorizes phenolic acids in olive into four distinct subclasses: cinnamic acid, benzoic acid, phenylpropanoic acid, and phenylacetic acid derivatives, based on their structural variations and functional properties.

#### 5.3.1 Cinnamic acid derivatives

**Fig. 5:**
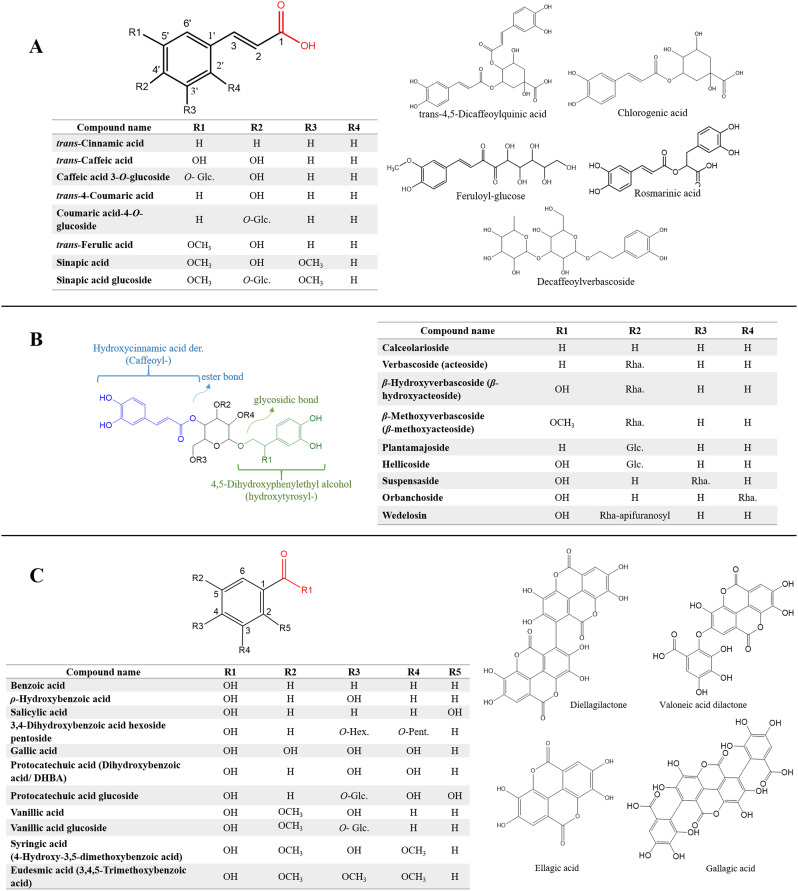
Subclasses of phenolic acids reported in *Olea europaea* L., featuring parent structures of (A) cinnamic acid compounds, (B) hydroxycinnamic acid derivatives (verbascosides), and (c) benzoic acid derivatives.

Cinnamic acid derivatives possess a basic C_6_–C_3_ skeleton, as seen in caffeic acid and its glucoside forms, as well as in cinnamic acid, which lacks hydroxyl groups on its aromatic ring.^38^ Additionally, *ρ*-coumaric acid is classified as a hydroxycinnamic acid and glucoside.^39^ Moreover, these compounds also feature substitutions by methoxy groups, as observed in ferulic (*trans*-3-M,4-HCA) and sinapic acids, which contain one and two methoxy groups, respectively.^40^ The latter is also found in a glycosidic form, as illustrated in Fig. 5A, with positional numbering. Chlorogenic acid, *trans*-4,5-dicaffeoylquinic acid (*trans*-4,5-DCQA or isochlorogenic acid C), rosmarinic acid, decaffeoylverbascoside, and feruloyl-glucose are further examples of hydroxycinnamic acid derivatives.^1,34,40–42^

#### 5.3.2 Verbascoside

Verbascoside (also known as acteoside, kusaginin, and caffeoylphenyl ethanoid),^43^ a hydroxycinnamic acid derivative, represents the predominant hydroxycinnamic acid derivative in *Olea europaea*, with its concentration increasing in fruits during ripening.^44^ It consists of caffeic acid and hydroxytyrosol, linked *via* a glucopyranose unit to which a rhamnosyl moiety is attached Fig. 5B.^45^ First isolated from mullein by^46^ and structurally confirmed by,^47^ verbascoside exhibits neuroprotective, antioxidant, antineoplastic, and anti-inflammatory properties. Calceolarioside, verbascoside of monosaccharide derivative (no rhamnosyl moiety), was first reported in olive leaves (Fig. 5B) by.^48^ Additional derivatives, including β-hydroxyverbascoside (β-hydroxyacteoside) and β-methoxyverbascoside (β-methoxyacteoside),^34,48^ as well as the addition of a glucosyl moiety instead of a rhamnosyl unit in verbascoside or hydroxyverbascoside form, plantamajoside, and hellicoside, respectively.^1,42^ Other structurally related compounds include suspensaside, orobanchoside, and wedelosin.^1^

#### 5.3.3 Benzoic acid derivatives

Benzoic acid derivatives share a characteristic C_6_–C_1_ structure, consisting of a carboxyl (–COOH) group attached to an aromatic ring. Their structural diversity arises from hydroxyl and methoxy substitutions, forming various phenolic compounds.^23^ For instance, gallic acid possesses hydroxyl groups at the 3, 4, and 5 positions, while its dimeric form, ellagic acid, displays further structural complexity.^43,49^ Eudesmic acid (3,4,5-trimethoxybenzoic acid) features three methoxy groups,^50^ whereas syringic acid has two.^51^ Salicylic acid is characterized by a hydroxyl group at the C-2 (ortho) position, in contrast to *ρ*-hydroxybenzoic acid, which features the hydroxyl group at the C-4 (ref. 52) position.^4^ Additional derivatives include vanillic acid, protocatechuic acid (a dihydroxybenzoic acid), and their glycosylated forms, such as 3,4-dihydroxybenzoic acid hexoside pentoside and valoneic acid dilactone,^2,53^ as illustrated in Fig. 5C. The positional numbering of these compounds is mentioned in.^38^

#### 5.3.4 Phenylacetic acids

**Fig. 6:**
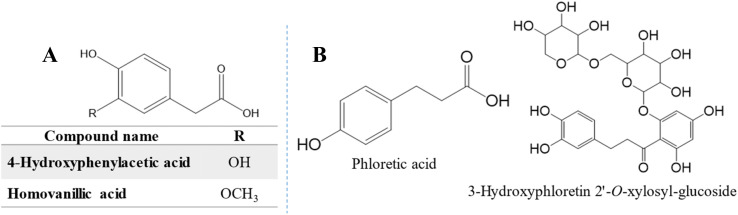
Phenolic acids cont. reported in *Olea europaea* L. showing (A) phenylacetic acid and (B) phenylpropanoic acid compounds.

Phenylacetic acids, characterized by a C_6_–C_2_ skeleton (Fig. 6A), include compounds such as 4-hydroxyphenylacetic acid and homovanillic acid.^28,54^

#### 5.3.5 Phenylpropanoic acids

Phenylpropanoic acids, characterized by a C_6_–C_3_ skeleton,^55^Fig. 6B, include phloretic acid^56^ and 3-hydroxyphloretin 2′-*O*-xylosyl-glucoside.^53^

### 5.4. Coumarins

**Fig. 7:**
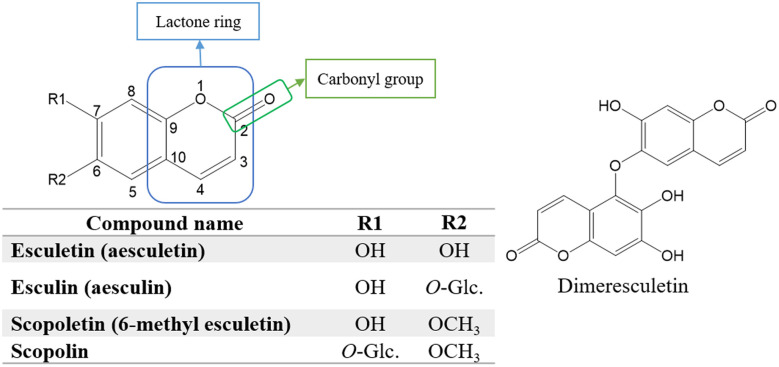
Basic skeleton of coumarin-based compounds reported in *Olea europaea* L.

Coumarin is a heterocyclic organic compound (C_9_H_6_O_2_) belonging to the benzopyrone (benzo-α-pyrone) family, characterized by a benzene ring fused with an unsaturated lactone ring, forming the C_6_–C_3_ skeleton,^57,58^Fig. 7. *O. europaea* contains hydroxycoumarin (esculetin/aesculetin), its glucoside, esculin/aesculin, and its isomer cichoriin.^59^ Additionally, scopoletin (6-methylesculetin) and its glucoside, scopolin (scopoletin 7-*O*-glucoside), as well as dimeresculetin, a hydroxycoumarin dimer, were detected.^18,59,60^ Structural details and numbered positions of the basic skeleton are depicted in Fig. 7, based on.^61^

### 5.5. Flavonoids

**Fig. 8:**
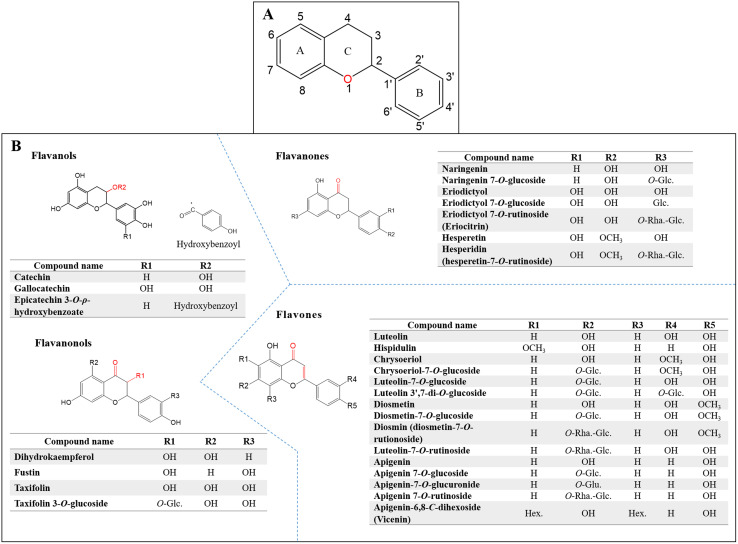
Flavonoid core structure with positional numbering (A) and representative subclasses reported in *Olea europaea* L. (B).

**Fig. 9:**
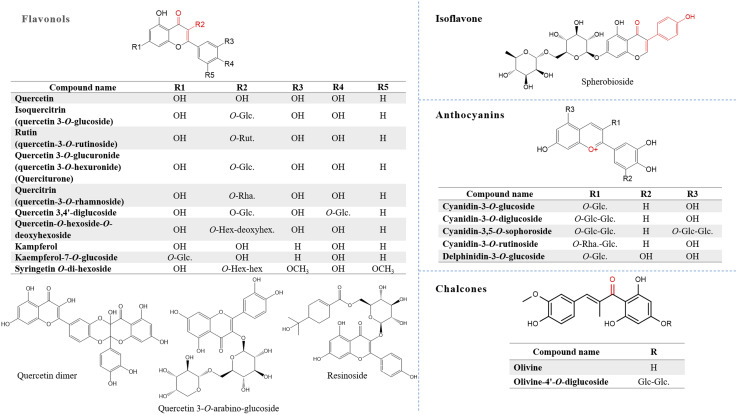
Flavonoid subclasses cont. reported in *Olea europaea* L., showing flavonols, isoflavones, anthocyanins, and chalcones.

Flavonoids are secondary metabolites abundantly found in seeds and fruits, contributing to flavor, fragrance, pigmentation, and various biological functions.^62^ Structurally, flavonoids consist of three rings: A, B, and C. Rings A and C form the chromane ring, with the C ring being the central heterocyclic pyran ring comprising an oxygen atom at position 1. This central ring connects the two aromatic rings (A and B), establishing the flavone backbone with a C15 (C_6_–C_3_–C_6_) configuration, according to the official IUPAC ref. 63 and 64, Fig. 8A. Flavonoid subclasses are distinguished based on unsaturation and functional groups attached to the C ring. Flavones, isoflavones, and flavonols contain a double bond at positions 2 and 3 of the C ring, along with a keto group at position 4, defining the anthoxanthin. Isoflavones differ by the attachment of the B ring at C-3 rather than C-2, while flavones, flavonols, flavanones, flavanonols, flavanols (catechins), anthocyanins, and chalcones exhibit variations in C-ring substitution patterns,^48^Fig. 8 and 9. Flavonoids exist either as free aglycones, where hydroxyl groups are typically found at positions 5 and 7 of the A ring, or as glycosides when conjugated to sugars, commonly through glycosylation at position 7 of the A ring through a glycosidic linkage. Glycosidic sugars include glucose, rhamnose, glucorhamnose, or deoxyhexose.^65^

#### 5.5.1 Flavones

Flavones, a subclass of flavonoids found in *O*. *europaea*, are characterized by a double bond between C-2 and C-3 and a ketone (C=O) group at position C-4 on the central (C) ring, as described in Fig. 8B. Among the aglycone flavones identified in olive leaves and fruits, luteolin^43^ and apigenin contribute significantly to their antioxidant and anti-inflammatory properties.^62^ Other aglycones include diosmetin (luteolin 4′-methyl ether), which has a methoxy group at position 4′ on the B ring,^33^ hispidulin, distinguished by a methoxy substitution at position 6 on the A ring,^42^ and chrysoeriol. Flavones also occur extensively as glycosylated derivatives. Apigenin-6,8-*C*-dihexoside (vicenin) is a *C*-glycoside in which the sugar moiety is directly bound to the flavone skeleton *via* carbon–carbon bonds.^41^ In contrast, at position C-7 on the A ring yields a variety of compounds including chrysoeriol-7-*O*-glucoside, luteolin-7-*O*-glucoside, luteolin-7-*O*-rutinoside, and luteolin 3′,7-di-*O*-glucoside.^42,66^ Further *O*-glycosylated derivatives of diosmetin include diosmetin-7-*O*-glucoside (luteolin 4′-methyl ether 7-*O*-glucoside) and diosmetin rutinoside (luteolin 4′-methyl ether 7-*O*-rutinoside/diosmin).^40,43^ Similarly, apigenin undergoes glycosylation at position 7 to produce its glucoside, glucuronide, and rutinoside.^28,40^

#### 5.5.2 Flavanonols

Flavanonols are structurally related to both flavonols and flavanones. Their distinction from flavonols lies in the absence of a double bond between positions 2 and 3 in the C ring, classifying them as dihydroflavonols. Conversely, flavanonols differ from flavanones by the presence of a hydroxyl (–OH) group at position 3 of the C ring (Fig. 8B). Notable examples of this subclass include dihydrokaempferol,^50,67^ fustin, and taxifolin, along with its glucoside.^43,59^

#### 5.5.3 Flavanols

Flavanols, called flavan-3-ols or catechins, share a structural relationship with flavonoids but lack the keto group at position 4 on the C ring. The common flavan-3-ols include catechin,^68^ gallocatechin,^69^ and epicatechin 3-*O-ρ*-hydroxybenzoate,^53^ as illustrated in Fig. 8B.

#### 5.5.4 Flavanones

Flavanones, also known as dihydroflavones, are structurally characterized by the absence of a double bond between C-2 and C-3 in the C ring. They are biosynthetically derived from 2′-hydroxychalcones through intramolecular ring closure.^62^ Among the predominant aglycone flavanones are naringenin and eriodictyol, along with their glucoside, eriodictyol rutinoside (eriocitrin).^18,40^ In addition, hesperetin and its *O*-glycosylated derivative, hesperetin 7-*O*-rutinoside (hesperidin), have been reported in olive tissues,^4,51^ as shown in Fig. 8B.

#### 5.5.5 Flavonols

Flavonols, a subgroup of flavonoids presents in *O. europaea*, closely resemble flavones but are distinguished by the presence of a hydroxyl (–OH) group at position 3 on the C ring, which facilitates glycosylation at this position (Fig. 9). Key flavonols identified include quercetin and kaempferol,^42^ along with their glycosylated derivatives such as isoquercitrin (quercetin 3-*O*-glucoside), rutin (quercetin-3-*O*-rutinoside),^18^ quercitrin (quercetin-3-*O*-rhamnoside), quercetin 3-*O*-glucuronide (querciturone),^34^ quercetin 3,4′-diglucoside, kaempferol-7-*O*-glucoside,^43^ and syringetin *O*-di-hexoside.^34^ Other notable compounds in *O. europaea* include quercetin dimer,^34^ quercetin 3-arabino-glucoside, and resinoside (structurally related to kaempferol).^70,71^

#### 5.5.6 Isoflavones

Isoflavones, called phyto-estrogens due to their structural similarity to mammalian estrogen,^72^ are distinguished from other flavonoids by the attachment of the B ring to the C ring at position C-3, rather than C-2 as in most other subclasses (Fig. 9). In *O. europaea*, the isoflavone spherobioside has been identified.^14^

#### 5.5.7 Anthocyanins

Anthocyanins are plant pigments responsible for the purple coloration of olives. They are characterized by the presence of an oxygen atom carrying a positive charge at position C-1 of the C ring, along with hydroxyl substitutions at positions C-3, C-5, and C-7. The B ring also typically carries one or more hydroxyl groups (Fig. 9). The most common anthocyanins in olive are cyanidin 3-*O*-glucoside and cyanidin 3-*O*-rutinoside, which together account for approximately 90% of the total anthocyanin content in olive fruit.^35,39^ Additional anthocyanins identified include delphinidin-3-*O*-glucoside, cyanidin-3-*O*-diglucoside, and cyanidin-3,5-*O*-sophoroside, the latter being reported for the first time in olives. Sophoroside is defined as a disaccharide composed of two glucose units linked *via* a β-1,2-glycosidic bond.^39^

#### 5.5.8 Chalcones

Chalcones, a distinct subgroup of flavonoids, are structurally characterized by their open-chain configuration, resulting from the absence of the C ring typically found in the flavonoid backbone (C_6_–C_3_–C_6_).^73^*O. europaea*, the chalcone olivine has been identified as a representative compound^48^ (Fig. 9).

### 5.6. Iridoids

**Fig. 10:**
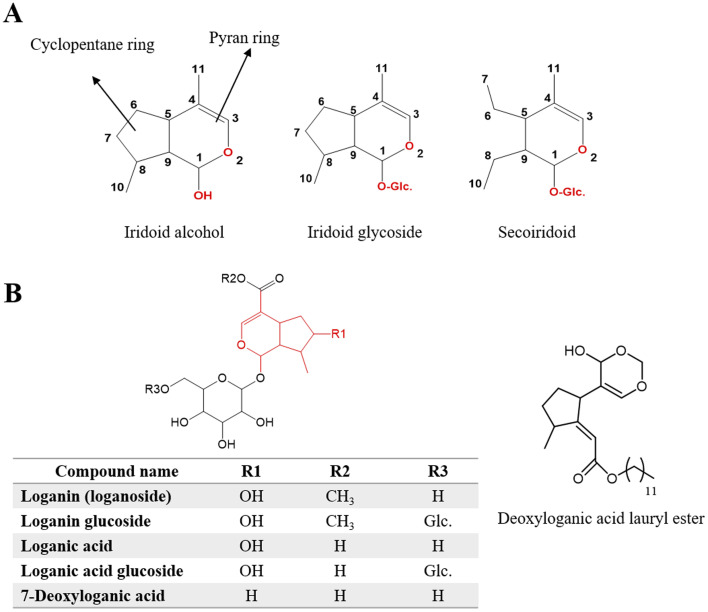
General structures of iridoid and secoiridoid with position numbering (A), and representative iridoids reported in *Olea europaea* L. (B).

Iridoids, a class of monoterpenes possessing a hemiacetal hydroxyl group, are characterized by a six-membered pyran ring fused to a cyclopentane ring, forming what is frequently referred to as a pyranoid or iridan skeleton.^74,75^ The pyran ring features a hydroxyl group at C-1, making iridoid alcohols inherently, leading to their tendency to form glycoside compounds upon combining with sugar unit,^75^Fig. 10A. Among the major reported iridoid glycosides, loganin (loganoside) has been identified in various parts of *O.europaea*, including wood and pomace, and other parts, alongside loganin glucoside, loganic acid^43^ and its glucoside.^48^ Additionally, 7-deoxyloganic acid, a precursor to loganic acid and loganin,^76^ and deoxyloganic acid lauryl ester have been reported,^77^Fig. 10B.

### 5.7. Secoiridoids

Secoiridoids, a subclass of monoterpenes (terpenes), are among the most extensively studied compounds in *Olea europaea*, with oleuropein and ligustroside being particularly abundant in its leaves, fruits, and seeds. These compounds contribute significantly to olive-related health benefits, including antioxidant, anti-inflammatory, and cardioprotective properties.^39,78^ Structurally, secoiridoids share similarities with iridoids, but their cyclopentane ring undergoes cleavage between C7 and C8, forming structures characterized by the presence of elenolic acid (EA) or its derivatives,^79^Fig. 10A. Additionally, they exhibit structural variations compared to iridoid glycosides.^75^ For the first time in this review, secoiridoids are classified into distinct subgroups based on the type of nucleus linked to secoiridoid base skeleton: 1. elenolic acid/elenolate-related compounds, 2. oleoside and secologanoside derivatives (sugar-conjugated), 3. oleuropein derivatives (hydroxytyrosol-based structure), 4. ligustroside derivatives (tyrosol-based structure), 5. Nuzhenide-type secoiridoids, and 6. other secoiridoids not included in previous classification. The conjugations primarily occur at position 7, where oxidation leads to carboxylic acid formation, which is subsequently esterified with various groups to generate different compounds. Many secoiridoids in olive, such as secologanin and secologanic acid, originate from deoxyloganic acid, a common intermediate. Also, elenolic acid glucoside (oleoside 11-methyl ester) is the primary precursor to oleuropein and ligustroside.^76^

#### 5.7.1 Elenolic acid/elenolate-type secoiridoids (simple secoiridoids)

**Fig. 11:**
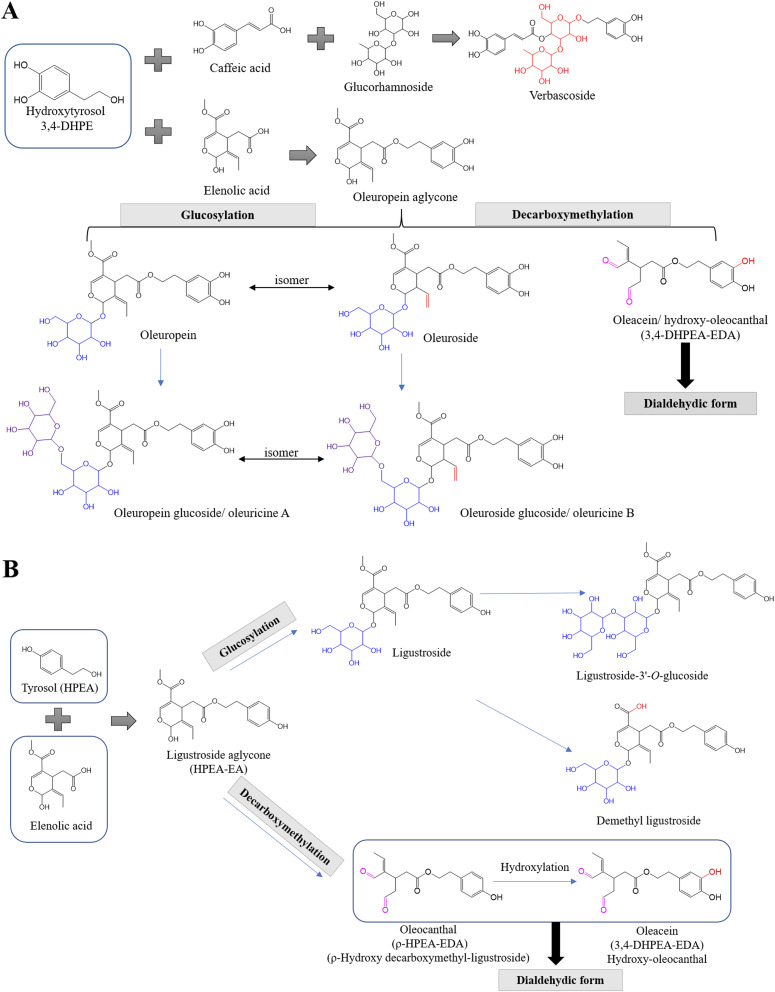
(A & B). Schematic representation highlighting tyrosol, hydroxytyrosol, and elenolic acid as core building blocks for major secoiridoids compounds in *Olea europaea* L., demonstrating their central role in generating structurally diverse derivatives with distinct functional groups.

**Fig. 12:**
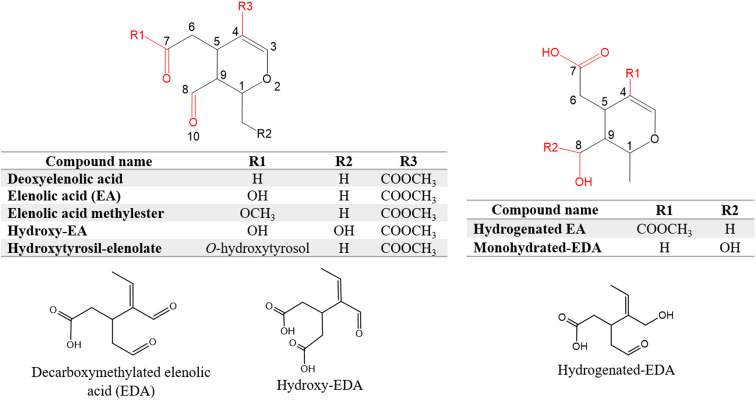
Basic elenolate-type secoiridoid skeleton with position numbering and representative derivatives in *Olea europaea* L.

Elenolic acid (EA), also known as elenaic acid, is one of the most significant secondary metabolites in *O*. *europaea*.^34,80^ It is a non-phenolic compound containing dicarboxylic functional groups at C-7 and C-11, existing either in free form or as a methyl ethyl ester derivative.^76^ EA and its structural analogs constitute the iridoid framework and serve as precursors for the biosynthesis of key secoiridoid compounds, which exhibit numerous health benefits when conjugated with other molecules,^81^Fig. 11. For example, ligustroside aglycone (*ρ*-HPEA-EA) and oleuropein aglycone (3,4-DHPEA-EA) are formed *via* the esterification of EA with tyrosol (*ρ*-HPEA) or hydroxytyrosol (3,4-DHPEA), respectively, through an ester linkage.^82^ Additionally, several derivatives related to the elenolic acid-based skeleton have been identified, including deoxyelenolic acid, elenolic acid methylester, hydroxy-EA, elenolic acid decarboxymethylated (EDA),^19^ hydroxy-EDA, hydroxytyrosil–elenolate,^1^ hydrogenated-EA, hydrogenated-EDA, and monohydrated-EDA,^83^Fig. 12.

#### 5.7.2 Oleoside and secologanoside-type secoiridoids and derivatives

**Fig. 13:**
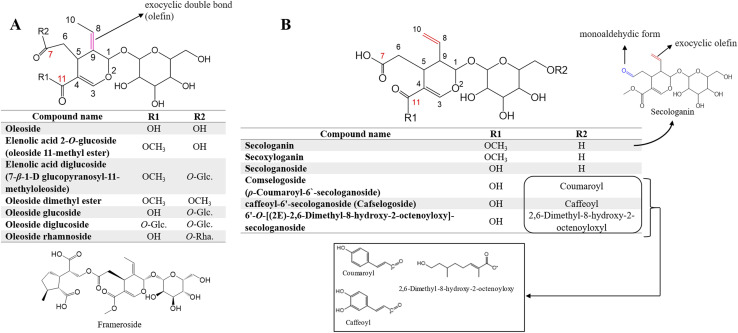
Basic skeletons of oleoside (A) and secologanoside-type secoiridoids (B) with derivatives reported in *Olea europaea* L.

Oleosides are non-phenolic compounds that may become involved in esterification with phenolic moieties. Most secoiridoids isolated from the Oleaceae family are predominantly found in olive fruits and leaves.^1,2^ The class name refers to the kind of compound conjugated or linked to the secoiridoid nucleus.^76^ Secologanoside is derived from oleoside, but the key structural difference lies in the position of the exocyclic olefin (C=C) bond. In oleoside, this bond is situated between carbon atoms C8–C9, whereas in secologanoside, the exocyclic C=C bond shifts from C8–C9 to C8–C10,^82,84^Fig. 13. Additionally, various derivatives of oleoside and secologanoside are formed through conjugation with different functional groups containing a carboxylic acid at position C-7, leading to structural diversity within this class.

Oleoside 11-methyl ester, also known as elenolic acid 2-*O*-glucoside,^2^ is a degradation product and precursor of oleuropein. Several oleoside-type secoiridoid derivatives have been identified in *O. europaea*, including elenolic acid diglucoside (also known as 7-β-1-d-glucopyranosyl-11-methyloleoside or glucosyl-methyloleoside),^14^ oleoside dimethyl ester,^33^ and frameroside.^34^ Furthermore, glycosylation at position 7 leads to the formation of various oleoside derivatives, such as oleoside glucoside (6′-β-glucopyranosyl-oleoside), oleoside diglucoside, and oleoside rhamnoside (rhamnopyranosyl oleoside),^1,33^Fig. 13A.

Secologanin, a monoaldehydic derivative of secologanoside, originates from deoxyloganic acid, a monoterpene glycoside that serves as the iridoid building unit for most terpenoid indole alkaloids.^85,86^ Secologanoside^48^ exhibits structural similarity to oleoside, as both possess dicarboxylic groups at C-7 and C-11. However, they differ in the position of the exocyclic C=C bond, as described previously, akin to the distinction between secoxyloganin^40,42^ and oleoside 11-methyl ester, both of which contain a methoxy group at C-11 (Fig. 13B). Among the identified secologanoside derivatives, *ρ*-coumaroyl-6′-secologanoside (comselogoside)^87^ and caffeoyl-6′-secologanoside (cafselogoside)^88^ are conjugated with a hydroxycinnamoyl moiety, linked to the sugar unit at C-1 of the secoiridoid basic skeleton. Additional secologanosides identified in the olive tree include 6′-*O*-[(2*E*)-2,6-dimethyl-8-hydroxy-2-octenoyloxy]-secologanoside,^13,41^Fig. 13B.

#### 5.7.3 Oleuropein-type secoiridoids and derivatives

**Fig. 14:**
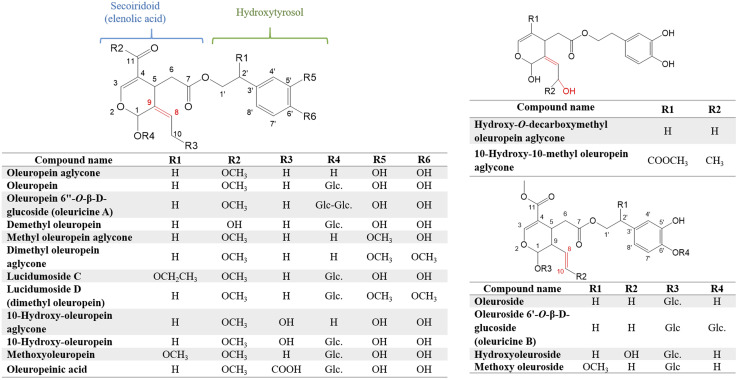
Oleuropein-type secoiridoids in *Olea europaea* L.: core composition with position numbering and representative derivatives.

Oleuropein, responsible for imparting bitterness, is a hallmark compound of *O. europaea*, abundantly present in olive leaves, seeds, oil, roots, stems, and fruit.^10,89^ Its concentration exhibits an inverse relationship with the fruit's maturation degree, meaning higher oleuropein levels are found in unripe olives, whereas its content decreases as the fruit ripens.^90^ Structurally, oleuropein consists of hydroxytyrosol (HTyr; 3,4-DHPEA), linked to oleoside 11-methyl ester (elenolic acid 2-*O*-glucoside) *via* an ester bond, forming the basic skeleton (Fig. 14). Since hydroxytyrosol acts as a precursor in oleuropein biosynthesis, this subclass can be referred to as “hydroxytyrosol-related secoiridoids”.^91^

Additional derivatives of oleuropein-type secoiridoids identified in *O. europaea* include 10-hydroxy-oleuropein aglycone, which features a hydroxyl group at C-10 adjacent to the exocyclic C8=C9 double bond,^92^ and methyl oleuropein aglycone,^34^ along with their glycosylated counterparts.^13,18,43^ Another notable compound is dimethyl oleuropein aglycone, characterized by methoxy groups at positions 5′ and 6′ of the hydroxytyrosol unit,^50^ as well as its glycosylated form, lucidumoside D^48^ (Fig. 14). Further structurally related derivatives include oleuropeinic acid, which bears a carboxylic group at C-10 (Fig. 14),^43^ and oleuropein 6″-*O*-β-d-glucoside, also known as oleuricine A, formed *via* glycosylation of oleuropein^2,34,83^ (Fig. 11A and 13).

Hydroxy-*O*-decarboxymethyl oleuropein aglycone is formed by the removal of the COOCH_3_ group from the pyran ring at position C-4 of oleuropein aglycone, accompanied by the introduction of a hydroxyl group at C-10.^48^ Similarly, 10-hydroxy-10-methyl oleuropein aglycone arises through the addition of a hydroxyl group and a methyl group at position C-10.^1^ In the same context, deacetoxyoleuropein aglycone results from the removal of the acetoxy group (Fig. 14).

Oleuroside is an isomeric form of oleuropein, distinguished by the position of the exocyclic double bond, which is located between C8 and C10 in oleuroside, as opposed to C8 and C9, a defining characteristic of oleoside and oleuropein,^51^Fig. 11A and 13. Upon glucosylation, oleuroside gives rise to oleuricine B (oleuroside 6′-*O*-β-d-glucoside), whereas oleuropein, when glucosylated, forms oleuricine A^88^ (Fig. 11A). Moreover, oleuroside acts as the core structure for several derivatives, including hydroxyoleuroside, which carries a hydroxyl group at C-10, and methoxy oleuroside, featuring a methoxy (OCH_3_) substitution at position C-2′ of the HTyr moiety,^48^Fig. 14.

**Fig. 15:**
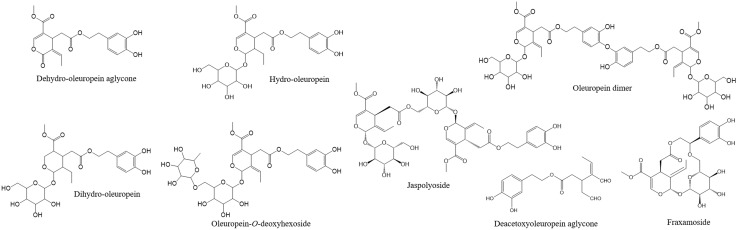
Additional oleuropein-type secoiridoids structures reported in *Olea europaea* L.

From oleuropein, structural modifications lead to the formation of several derivatives. Hydro-oleuropein and dihydro-oleuropein arise from cleavage of the exocyclic double bond between C-8 and C-9, with the latter undergoing an additional cleavage in the pyran ring.^43,93^ Conversely, dehydro-oleuropein aglycone forms through loss of a hydrogen atom at C-1 in the pyran ring, forming a keto group (C=O).^94^ Other notable oleuropein derivatives include oleuropein dimer, oleuropein-*O*-deoxyhexoside, fraxamoside, and jaspolyoside, which have been identified in olive fruit and olive wood extract,^2,94^Fig. 15.

#### 5.7.4 Ligustroside-type secoiridoids

**Fig. 16:**
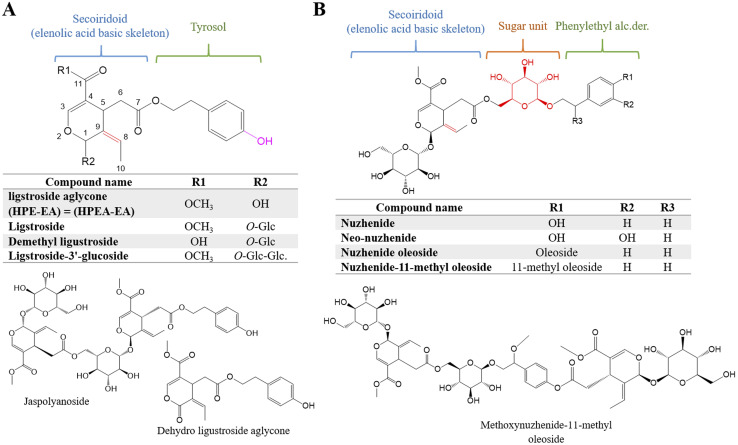
Basic skeletons of ligustroside-type (A) and Nuzhenide-type (B) secoiridoids in *Olea europaea* L., with position numbering and representative derivatives.

Ligustroside, a major secoiridoid compound distributed across all organs of *O. europaea*,^95^ shares close structural similarities with oleuropein. It comprises oleoside 11-methyl ester (elenolic acid glucoside) esterified with tyrosol (Tyr), rather than hydroxytyrosol (HTyr), and carries a sugar moiety at position C-1. Ligustroside also features an exocyclic double bond between C-8 and C-9, which is characteristic of this secoiridoids subclass,^96^Fig. 16A. Because tyrosol serves as the biosynthesis precursor to ligustroside (Fig. 11B), these compounds are often categorized as “tyrosol-related secoiridoids.” Similar to oleuropein, the concentration of ligustroside declines during olive fruit maturation. Meanwhile, ligustroside, though widely expressed in various olive tissues, has been infrequently found in olive seeds.^76^ Likewise, demethyl ligustroside exhibits decreased levels in olive leaves and fruits.^35,82^ Among its known derivatives, ligustroside aglycone (*ρ*-HPEA-EA) represents a mono-aldehydic form.^2,97^ Other notable ligustroside-type secoiridoids include dehydroligustroside aglycone,^50^ ligustroside-3′-*O*-β-d-glucoside,^1^ and jaspolyanoside,^98,99^Fig. 16A. Meanwhile, ligustroside, though widely expressed in various olive tissues, has been infrequently found in olive seeds.^76^

#### 5.7.5 Nuzhenide-type secoiridoids

Nuzhenide, a secoiridoids compound,^100^ features a sugar moiety directly attached to two distinct structural regions of its core skeleton (Fig. 16B). It was initially identified at high concentration in olive seed extract during fruit development, followed by a gradual decline through maturation, a pattern similar to that observed for oleuropein.^47,88^ In a later stage, nuzhenide was also detected in olive paste, albeit in lower amounts. The occurrence of nuzhenide in olive paste contributes to the accumulation of tyrosol, a degradation product released during malaxation, a key step in olive oil production. During this process, neo-nuzhenide, a dihydroxy derivative of nuzhenide, is formed through hydroxylation.^81^ Other notable derivatives include nuzhenide oleoside, detected in olive seed,^51^ and nuzhenide 11-methyl-oleoside, a methyl variant identified in olive fruit, seed, and paste. Additionally, methoxynuzhenide 11-methyl oleoside, an esterified form of nuzhenide, has been reported in both olive oil and seeds.^88,100^

#### 5.7.6 Other secoiridoids

**Fig. 17:**
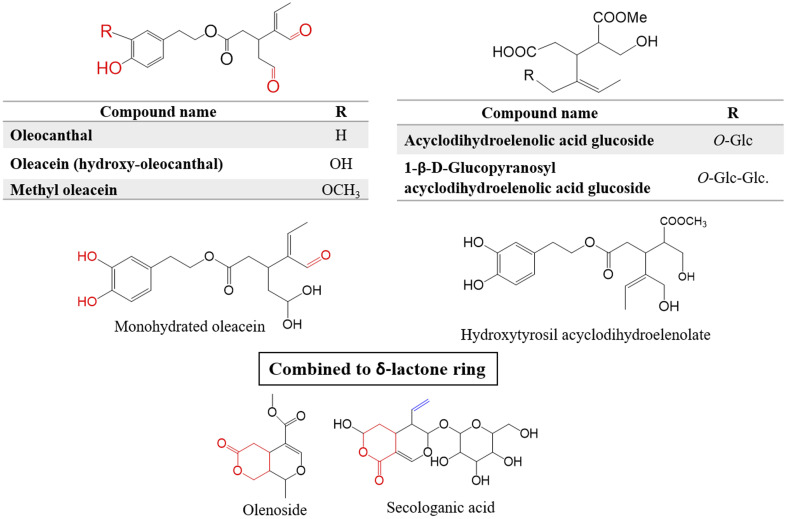
Other secoiridoid derivatives reported in *Olea europaea* L.

Secoiridoids featuring a δ-lactone ring, such as olenoside A, have been identified in olive leaf extracts.^80^ Its C-configuration epimer, olenoside B, has been detected in olive oil mill wastewater.^101^ A structurally related secoiridoid bearing the δ-lactone moiety is secologanic acid (Fig. 17). In addition, acyclic elenolic acid derivatives form a distinct subgroup of secoiridoids. These include acyclodihydroelenolic acid glucoside, 1-β-d-glucopyranosyl acyclodihydroelenolic acid glucoside,^93^ and hydroxytyrosil acyclodihydroelenolate^87^ (Fig. 17).

Within the dialdehydic secoiridoids subclass, oleocanthal (*ρ*-HPEA-EDA) and oleacein (3,4-DHPEA-EDA) are formed through decarboxymethylation of elenolic acid, and are structurally related to tyrosol (Tyr) and hydroxytyrosol (HTyr), respectively^102^ (Fig. 17). The name oleocanthal derives from “oleo-”(olive), “canth-” (stinging sensation), and “-al” (indicating an aldehydic functional group).^103^ Oleacein, first identified in ripe olives,^104^ can undergo methylation to yield methyl oleacein (methyl decarboxymethyl oleuropein aglycone)^50^ (Fig. 11B).

### 5.8. Isochromans

**Fig. 18:**
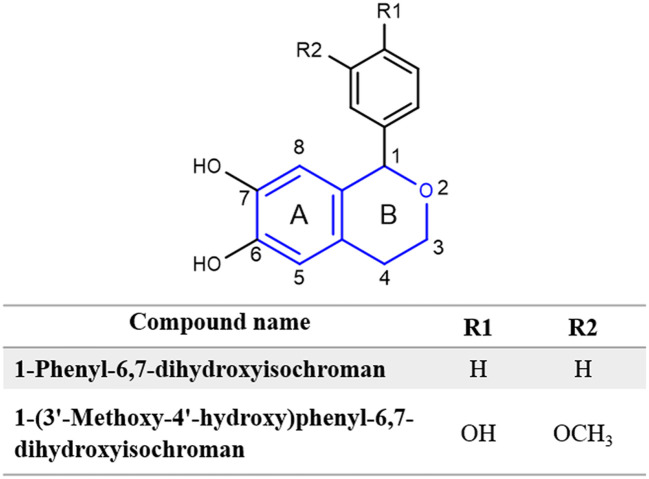
Isochroman derivatives reported in *Olea europaea* L.

Isochromans, derivatives of 3,4-dihydro-1*H*-benzo[*c*]pyran, possess a polyphenolic benzene nucleus (ring A) fused with a heterocyclic dihydropyran ring (ring B), as shown in the core structure (Fig. 18). These fused-ring systems are commonly encountered in nature and have been identified in extra-virgin olive oil.^105–107^ Hydroxy-isochromans are formed through a reaction between hydroxytyrosol and carbonylic compounds, proceeding *via* the oxa-Pictet-Spengler reaction, which occurs during the olive oil extraction process. Notably, these compounds are not naturally present in fresh olive fruits but are instead generated during processing and storage.^108^ During oil preparation, particularly in the kneading step, hydrolysis is triggered by the uncontrolled activity of enzymes such as esterases and glycosidases. This enzymatic activity increases the levels of free hydroxytyrosol and carbonyl compounds, creating favorable conditions for isochroman formation through the convergence of key reactants.^107^ Representative isochroman derivatives include 1-phenyl-6,7-dihydroxyisochroman and 1-(3′-methoxy-4′-hydroxy)phenyl-6,7-dihydroxyisochroman,^20,107^ both of which feature aromatic ring substitutions on the isochroman core (Fig. 18).

### 5.9. Lignans

**Fig. 19:**
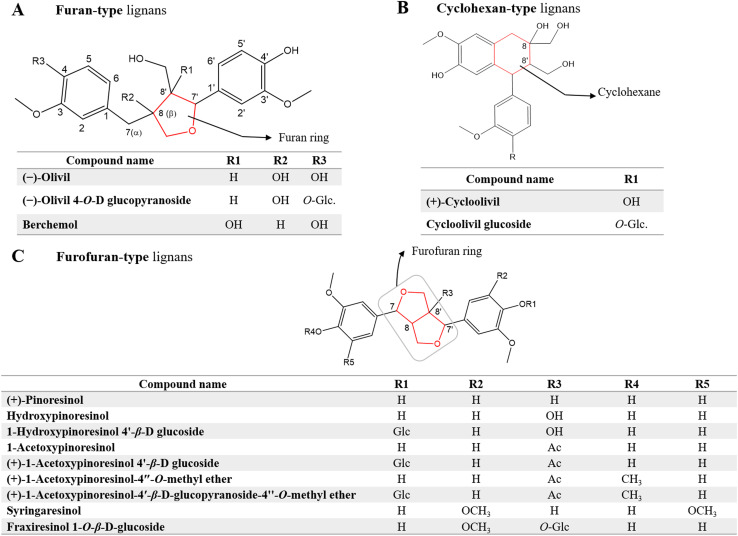
Lignan structures reported in *Olea europaea* L., newly classified by core skeleton: (A) olivil-based with furan ring, (B) cycloolivil-based with cyclohexane ring, and (C) pinoresinol-based with furofuran ring.

Lignans, a structurally significant class of phenolic compounds in the *Olea europaea*, are present in all plant parts, predominantly in the stem and roots, and have been isolated from bark and wood tissues. Chemically, lignans are composed of two phenylpropanoid units, connected *via* a β,β′ (or 8,8′) linkage, forming a basic (C_6_–C_3_)_2_ skeleton in accordance with the IUPAC recommendations.^109,110^ For the first time in this review, lignans are categorized into three distinct subclasses based on their core structural motifs, which determine the mode of linkage between phenylpropanoid units: furan, cyclohexane, and furofuran (Fig. 19).

Among them,*furan-type lignans* “olivil-based structures” include olivil and its glycosylated form olivil 4-*O*-β-d-glucopyranoside,^18,48^ as well as berchemol,^1^Fig. 19A. In contrast, *cyclohexane-type lignans* “cycloolivil-based lignans” are exemplified by cycloolivil and its glucoside,^1,18^Fig. 19B. Meanwhile, *furofuran-type lignans* “pinoresinol-based lignans”, characterized by a fused heterobicyclic ring system consisting of two furan rings, include syringaresinol, (+)-pinoresinol,^14,28^ 1-hydroxypinoresinol,^33^ 1-acetoxypinoresinol, (+)-1-acetoxypinoresinol-4″-*O*-methyl ether and their corresponding glucosides (except pinoresinol itself), along with fraxiresinol 1-*O*-β-d-glucoside,^1,34,111^Fig. 19C. This classification enhances our understanding of lignan diversity in *Olea europaea*, highlighting their structural complexity and potential significance within plant metabolism.

### 5.10. Terpenoids

Terpenoids, consisting of isoprene units of five carbons, are classified based on the number of these units, forming monoterpenes (two isoprene units), sesquiterpenes (three units), diterpenes (four units), and triterpenes (six units).^112^

#### 5.10.1 Monoterpenoids

**Fig. 20:**
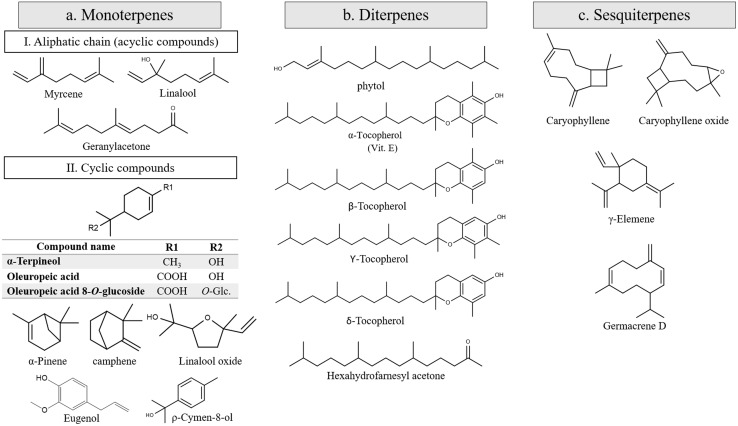
Terpenoids reported in *Olea europaea* L., classified into (a) monoterpenes, (b) diterpenes, and (c) sesquiterpenes.

Monoterpenoids encompass both hydrocarbon monoterpenes and their oxygenated derivatives, representing a wide variety of compounds, including monoterpene alcohols, ketones, aldehydes, acids, esters, iridoids, and glycosides.^113^ These compounds are further classified into acyclic, monocyclic, and bicyclic structures. Among the most abundant acyclic monoterpenes reported in *O. europaea* are myrcene,^114^ linalool (3,7-dimethylocta-1,6-dien-3-ol),^115^ and geranylacetone.^116^ Monocyclic monoterpenes include *ρ*-cymen-8-ol (2-(4-methylphenyl)propan-2-ol) and linalool oxide (furanoid), both found in trace amounts, along with α-terpineol,^116^ oleuropeic acid, and its glucoside.^117^ In contrast, α-pinene^118^ and camphene^114^ represent bicyclic monoterpenes, Fig. 20.

Among *diterpenes*, phytol and hexahydrofarnesyl acetone (6,10,14-trimethylpentadecan-2-one), both aliphatic chain compounds, have been identified in *O. europaea*,^115,119^Fig. 20. Within this category, tocopherols are notable as lipid-soluble natural antioxidants, playing a key role in protecting against lipid peroxidation by scavenging radicals in cellular membranes and lipoprotein particles. In olive oil, four distinct tocopherol isomers have been reported, namely α-, β-, γ-tocopherols, which are also present in olive fruits,^1,50^ and δ-tocopherol,^14^Fig. 20.

Furthermore, caryophyllene, caryophyllene oxide, γ-elemene, and germacrene D were reported in *O. europaea* as sesquiterpenes in leaves and olive oil,^114,120^Fig. 20.

**Fig. 21:**
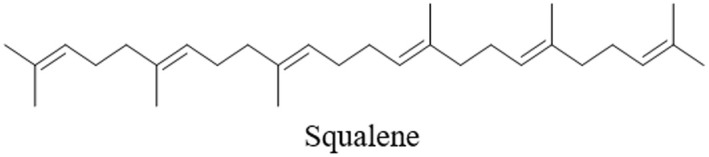
Structure of the acyclic triterpene squalene reported in *Olea europaea* L.

**Fig. 22:**
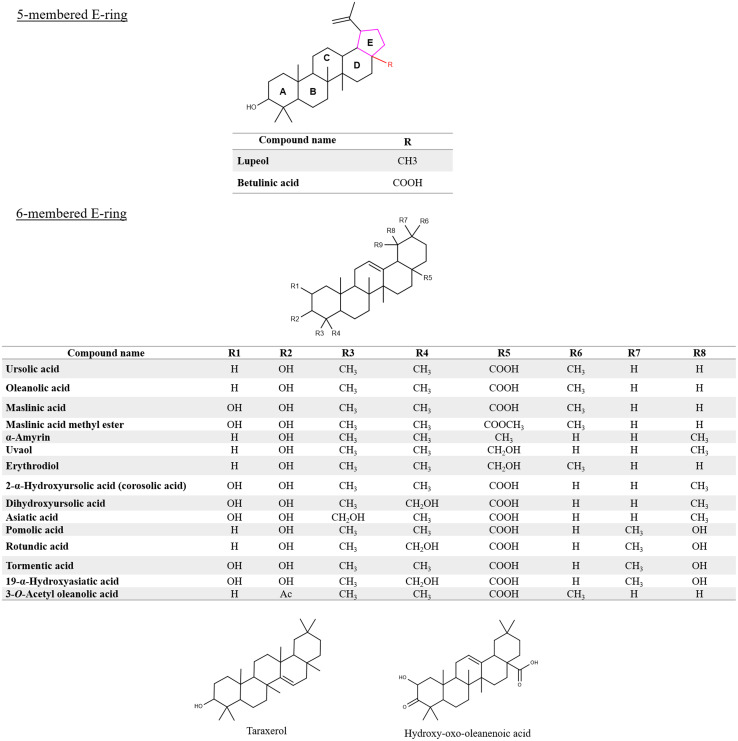
Classification of pentacyclic triterpenes of *Olea europaea* L. based on the E-ring structures.

Triterpene compounds in *Olea europaea* are categorized into pentacyclic triterpenes and other types, such as squalene (Fig. 21 and 22). Squalene, an acyclic polyunsaturated aliphatic triterpene, has been identified in leaves, pomace, fruit, and olive oil and functions as a biosynthetic precursor for phytosterols and steroid compounds,^121,122^Fig. 21. It constitutes a major component of the oil's unsaponifiable fraction, making up approximately 40% of this fraction and serving as its main bioactive constituent.^35,123^ The unsaponifiable fraction refers to the residue remaining after saponification with an alkaline hydroxide and solvent extraction.^85^

Pentacyclic triterpenes, characterized by their five fused rings, can be structurally categorized in *Olea europaea* according to the nature of the E-ring. This classification distinguishes two major groups (Fig. 22). The first group includes compounds such as lupeol and betulinic acid,^1,124,125^ characterized by a tetracyclic backbone of four fused six-membered rings (A, B, C, and D), capped by a five-membered E ring. In contrast, the second group comprises triterpenes with five six-membered rings, such as ursolic acid, oleanolic acid,^42^ maslinic acid and its methyl ester,^34,35^ as well as α-, β-, and δ-amyrin isomers.^119,126,127^ Other examples in this category include uvaol, erythrodiol,^128^ corosolic acid (2-α-hydroxyursolic acid) and its dihydroxy analog,^124^ asiatic acid, pomolic acid, rotundic acid, and tormentic acid.^43,129^ Furthermore, structural derivatives such as 19-α-hydroxyasiatic acid,^124^ 3-*O*-acetyl oleanolic acid,^88^ hydroxy-oxo-oleanolic acid,^43^ and taraxerol^1^ added to the diversity of this group. Notably, erythrodiol and uvaol are particularly abundant in olive oil and serve as biomarkers for distinguishing virgin olive oil from refined oils, based on their relative abundance,^35,88^Fig. 22.

### 5.11. Phytosterols

**Fig. 23:**
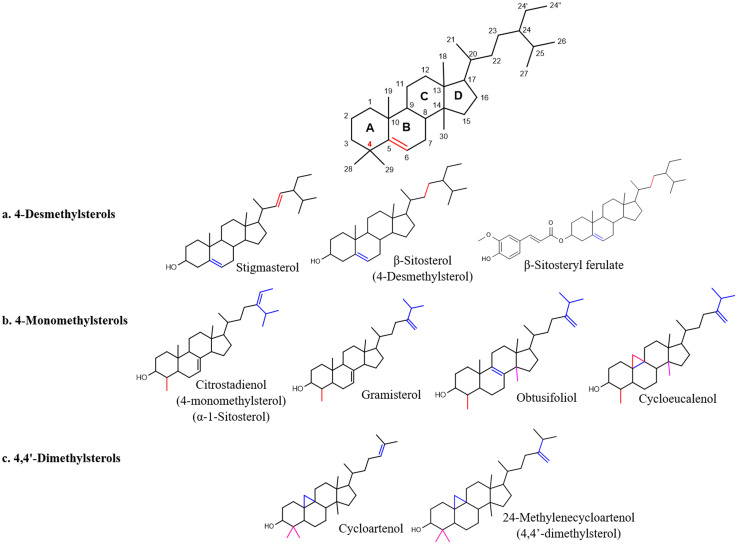
Basic sterol skeleton in *Olea europaea* L., showing carbon atom positions and classification of related compounds into three structural subclasses: (a) 4-desmethylsterols, (b) 4-monomethylsterols, and (c) 4,4′-dimethylsterols.

Phytosterols, triterpene-derived compounds, are classified as a distinct group due to their characteristic tetracyclic structure, comprising three six-membered rings (A, B, and C) and a five-membered D-ring, as defined by IUPAC nomenclature,^130,131^Fig. 23. In *Olea europaea*, several phytosterol structures have been identified, differing primarily in their aliphatic side chains at C-17 of the D-ring and the absence or presence of methyl group at C-4 of the A-ring.^132^ Based on these structural variations, phytosterols are categorized into three subgroups: a. 4-desmethylsterols (no methyl at C-4), referred to as true phytosterols, include stigmasterol, β-sitosterol,^128^ both found in leaves, fruits, and olive oil, and β-sitosteryl ferulate,^53^ present in leaves. b. 4-Monomethylsterols (one methyl group at C-4) include citrostadienol, gramisterol, and obtusifoliol, detected in fruits, seeds, and olive oil, as well as cycloeucalenol, reported in fruit and olive oil.^1,127^ c. 4,4′-dimethylsterols (two methyl groups at C-4), such as 24-methylenecycloartenol and cycloartenol,^125^ are present in seeds, fruits, and olive oil. These compounds feature a cycloartenol moiety, placing them within the cycloartane-type triterpenoid subclass,^1,127^Fig. 23.

### 5.12. Fatty acid composition of olive oil

Fatty acids (FAs) are carboxylic acid compounds (R–COOH) with an aliphatic chain that may be saturated or unsaturated, and are found in various parts of *O. europaea*, predominantly in olive oil (see SI Table S1). Fatty acids are named using two nomenclature systems: the systematic name (IUPAC name) and the more widely used trivial name in practical contexts.^133^ An additional “shorthand nomenclature” simplifies this further by indicating the number of carbon atoms in the aliphatic chain, the number of double bonds, and the position of the first double bond (using the “*n*” notation, counted from the methyl end).^134^

#### 5.12.1 Saturated fatty acids (SFAs)

**Fig. 24:**
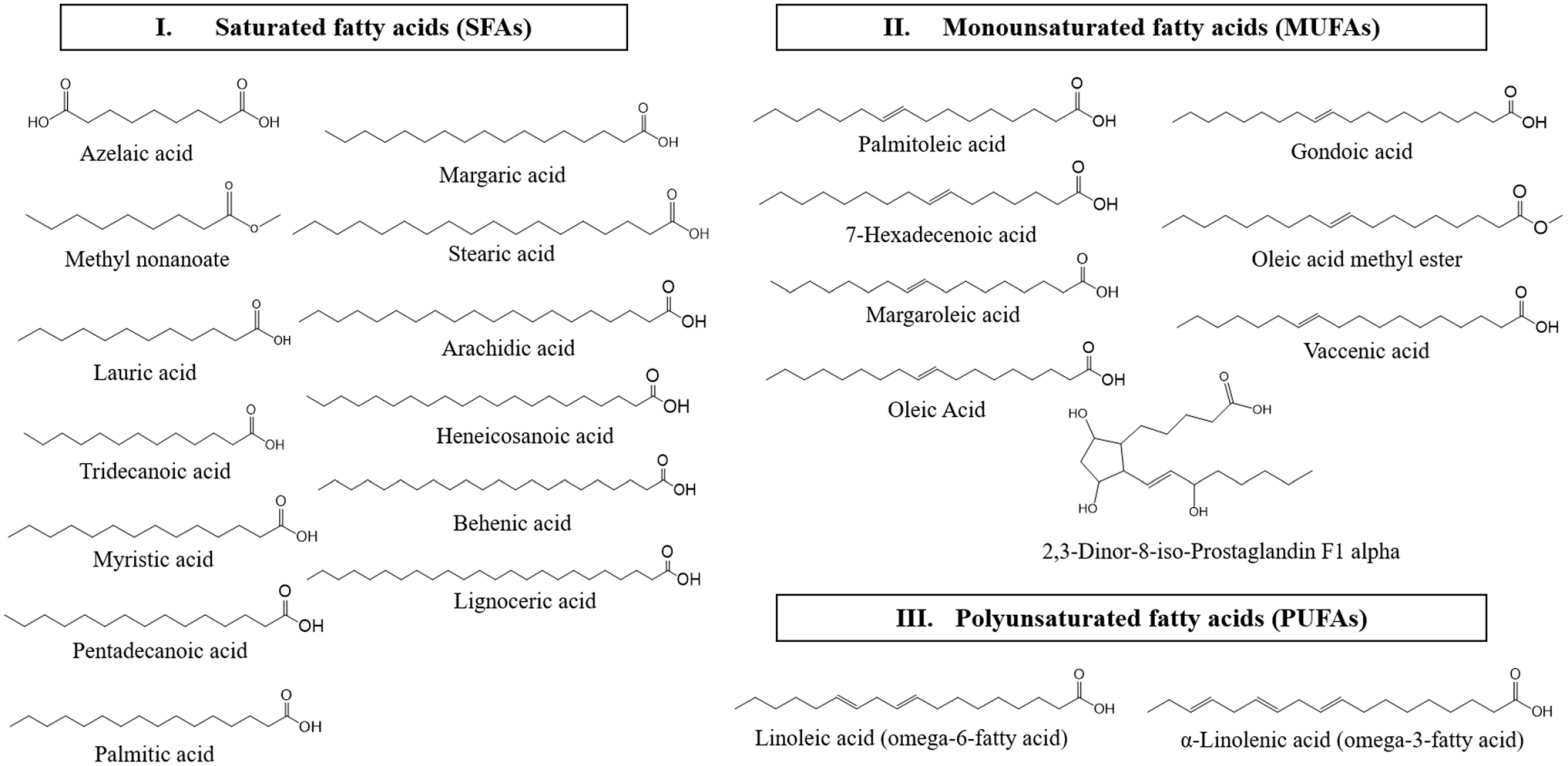
Representative fatty acids in *Olea europaea*, including SFAs(i), MUFAs(ii), and PUFAs(iii).

Saturated fatty acids are essential constituents of olive oil, contributing to its chemical stability and nutritional profile. The most prominent among them is palmitic acid (hexadecanoic acid, C16 : 0), typically present in high concentrations across various samples,^9,133^ followed by stearic acid (octadecanoic acid, C18 : 0),^50^ which is found in lower concentrations, particularly during early fruit stages and varies with cultivar maturation.^128^ Other SFAs include azelaic acid (1,9-nonanedioate, C9 : 0),^96^ methyl pelargonate (methyl nonanoate, C9 : 0 ME),^135^ lauric acid (dodecanoic acid, C12 : 0),^34^ tridecanoic acid (C13 : 0),^136^ myristic acid (tetradecanoic acid, C14 : 0),^137^ pentadecanoic acid (C15 : 0),^136^ margaric acid (heptadecanoic acid, C17 : 0), arachidic acid (eicosanoic acid, C20 : 0), heneicosanoic acid (C21 : 0), behenic acid (docosanoic acid, C22 : 0), and lignoceric acid (tetracosanoic acid, C24 : 0), the latter classified as a very long-chain fatty acid,^9,80,133^Fig. 24I.

#### 5.12.2 Monounsaturated fatty acids (MUFAs)

MUFAs, or monoenoic fatty acids, are the most abundant class of fatty acids in olive oil^138^ and play a central role in its nutritional and oxidative properties. Oleic acid (*cis-*9-octadecenoic acid) (C18 : 1 *n*-9) is the dominant fatty acid in olive oil, consistently found in high concentration and recognized as the primary contributor to its health benefits,^118^ as well as its methyl ester (C18 : 1 *n*-9cME).^139^ Other MUFAs include palmitoleic acid (*cis-*9-hexadecenoic acid, C16 : 1 *n*-7),^140^ margaroleic acid (*cis*-9-heptadecenoic acid, C17 : 1 *n*-8), vaccenic acid (*cis*-11-octadecenoic acid, C18 : 1 *n*-7),^141^ gondoic acid (*cis-*11-eicosenoic acid/omega-9 fatty acid, C20 : 1 *n*-9),^142^ with minor compounds like 2,3-dinor-8-iso-prostaglandin F-1-alpha, also reported in olive oil,^9,143^Fig. 24II. Despite some MUFAs being present in trace amounts, they contribute to the chemical identity of olive oil cultivars.

#### 5.12.3 Polyunsaturated fatty acids (PUFAs)

PUFAs, or polyenoic fatty acids, as described in,^138^ are distinguished by having two or more double bonds in their carbon chain. Linoleic acid (C18 : 2 *n*-6), the principal omega-6 fatty acid, occurs in moderate to low concentrations in olive oil,^123^ particularly during the unripe stage, and varies according to olive cultivar and maturation.^128^ Another key PUFAs is α-Linolenic acid (C18 : 3 *n*-3), an omega-3 fatty acid with three double bonds, also referred to as 9,12,15-octadecatrienoic acid or (9*Z*,12*Z*,15*Z*)-octadeca 9,12,15-trienoic acid,^137^Fig. 24III. Although many other fatty acids appear in minor proportions, their biological relevance is notable, and they can serve as valuable markers for distinguishing between olive cultivars and oil quality profiles.^136,144^

### 5.13. Alkaloids

**Fig. 25:**
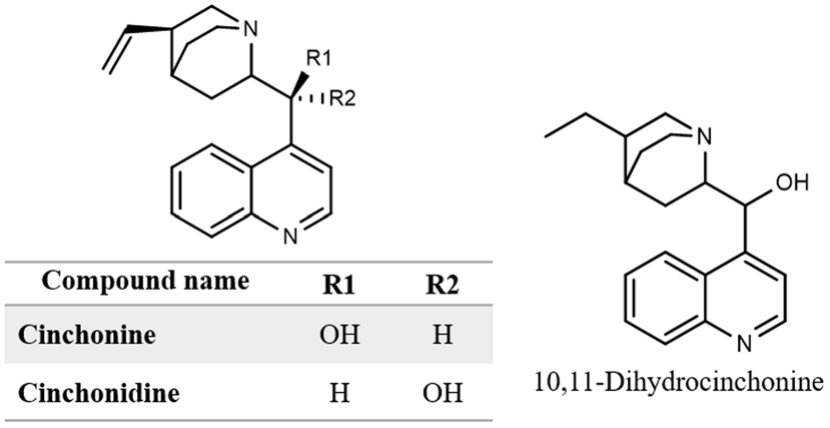
Alkaloids reported in *Olea europaea* L.

Alkaloids, a class of nitrogen-containing organic compounds, have been detected in *O. europaea* extracts, particularly in the herb, bark, and roots. Among them, cinchonine and its isomer cinchonidine have been reported in olive extracts.^146^ Additionally, 10,11-dihydrocinchonine is noted in the Dictionary of Natural Products (DNP), as illustrated in Fig. 25.

### 5.14. Aldehyde and ketone compounds

**Fig. 26:**
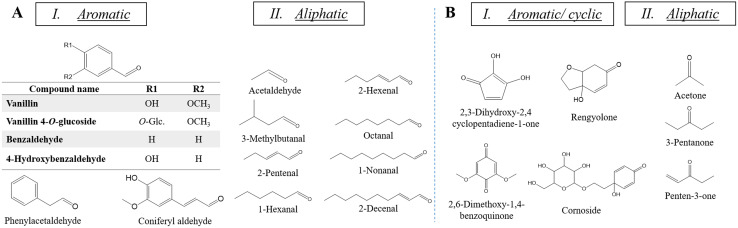
Aldehydic (A) and ketonic (B) compounds reported in *Olea europaea* L., categorized into aromatic and aliphatic subclasses.

Aldehydic and ketonic compounds have been identified as significant volatile components of *Olea europaea* L., contributing to its distinctive aroma and flavor profile. These volatiles have been reported in various parts of the plant, including the fruit, leaves, stem, wood, and pomace. Among the aliphatic *aldehydes*, key compounds detected include acetaldehyde, 3-methylbutanal,^147^ 2-pentenal, 1-hexanal, and 2-hexenal,^148^ as well as octanal, 1-nonanal (nonadienal), and 2-decenal.^116^ These were identified *via* headspace GC-MS analysis.^135^ In contrast, the aromatic aldehydes present include vanillin and glucovanillin (vanillin 4-*O*-glucoside),^14,28^ benzaldehyde,^149^ 4-hydroxybenzaldehyde,^14^ phenylacetaldehyde,^115^ and coniferyl aldehyde (4-hydroxy-3-methoxycinnamaldehyde),^14^ as illustrated in Fig. 26.

Similarly, various ketonic compounds, characterized by the presence of a carbonyl (C=O) group, contribute to the olive's volatile signature. Aliphatic ketones, such as acetone, 3-pentanone,^135^ and penten-3-one (ethyl vinyl ketone),^148^ have been reported. In contrast, aromatic ketones include 2,3-dihydroxy-2,4 cyclopentadien-1-one,^34^ rengyolone,^150^ 2,6-dimethoxy-1,4-benzoquinone,^97^ and cornoside (quinol glucoside).^1,151^ Notably, the latter two compounds are further classified under organic oxides, highlighting their unique structural features and biosynthetic versatility across different plant tissues (Fig. 26).

### 5.15. Other compounds

**Fig. 27:**
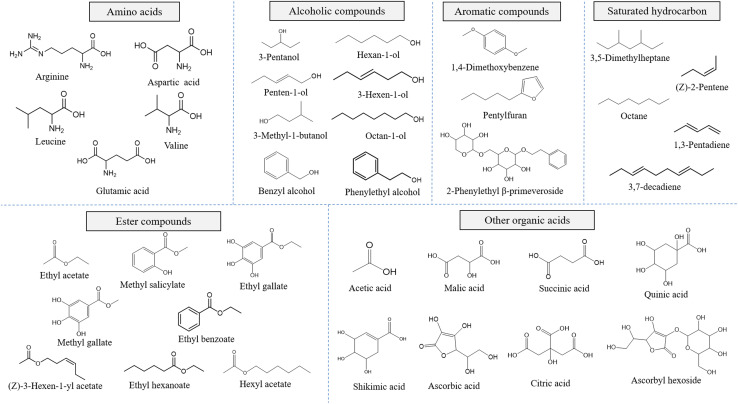
Other phytochemical classes reported in *Olea europaea* L.: amino acids, volatile alcohols, saturated hydrocarbons, aromatic and ester compounds, and other organic acids.

The nutritional value of olive fruit, particularly its protein content, has been widely studied, with notable variations depending on the cultivar and maturation stage. Although protein concentrations are relatively low, olive fruit contains all essential amino acids typically found in plant proteins, which are classified as primary metabolites due to their crucial role in cellular processes.^20^ Among these, aspartic and glutamic acids are the most abundant, followed by leucine and valine; high levels of phenylalanine have also been reported in certain cultivars,^20,33,96^Fig. 27. Additional compounds are documented in.^37^

Beyond proteins, *O. europaea* also contains a wide range of secondary metabolites not classified within the previously discussed classes (Fig. 27). Among saturated hydrocarbons detected in olive fruit extracts are 3,5-dimethylheptane, 2-pentene, 1,3-pentadiene, 3,7-decadiene, and octane.^135,147^ In addition, several aromatic compounds have been reported, including pentylfuran and 1,4-dimethoxybenzene,^116^ as well as β-d-glucopyranoside 2-phenylethyl 6-*O*-β-d-xylopyranosyl, commonly known as 2-phenylethyl β-primeveroside (Difonzo *et al.*, 2022;^41^ Fu *et al.*, 2010 ^143^), Fig. 27.

Similarly, various alcoholic compounds are present in olive extracts, ranging from aliphatic alcohols, such as 3-pentanol,^147^ penten-1-ol, 3-methyl-1-butanol,^148^ hexan-1-ol,^118^ 3-hexen-1-ol, and octan-1-ol^115,135^ to aromatic alcohols like benzyl alcohol and phenylethyl alcohol, which are discussed in greater detail under the simple phenol section,^116,147^Fig. 27.

Olive extracts also contain esters, which can be categorized into aliphatic esters, such as ethyl acetate,^147^ 3-hexenyl acetate, ethyl hexanoate, hexyl acetate, *cis*-3-hexenyl acetate,^114,135^ and aromatic esters, including ethyl benzoate, methyl salicylate, methyl gallate, and ethyl gallate,^43,115,147^Fig. 27.

Finally, olive fruit contains several other organic acids that do not fall under the previously defined classifications. These include acetic acid,^135^ malic acid,^2^ succinic acid,^14^ citric acid,^41^ quinic and shikimic acids,^96^ and ascorbic acid.^68^ Additionally, sugar-conjugated acids such as ascorbyl hexoside,^18^Fig. 27.

## 6. Comparative metabolite profiles of olive source

This section provides a detailed exploration of the diverse metabolite profiles identified across various *Olea europaea* L. organs and their key products. Table 1 serves as a concise summary, outlining the major compound classes and their characteristic metabolites for each source, providing an essential overview.

**Table 1:** Bioactive metabolites in olive tree organs and derived oils: major and some minor components

Source of metabolite	Major classes/metabolites	Reference
Leaves	- Secoiridoids: oleuropein (major compound), ligustroside	39, 50, 152, 158 and 159
	- Flavonoids: luteolin-7-glucoside (major flavonoid), apigenin-7-glucoside	
	- Simple phenols: hydroxytyrosol (major)	
	- Triterpenes: maslinic acid	
	- Phenolic acids: verbascoside, caffeic, and vanillic acids	
Stems	- Secoiridoids: oleuropein	50 and 155
	- Coumarins (major class): aesculetin	
	- Simple phenols: hydroxytyrosol	
	- Flavonoids: luteolin glycosides	
	- Lignans: acetoxypinoresinol	
	- Fatty acids: betulinic acid	
Fruit skin	- Secoiridoids: oleuropein (major in unripe fruit)	39, 47, 50 and 156
	- Triterpenes: oleanolic and maslinic acids	
	- Phenolic acids: *trans*-caffeic acid	
	- Flavonoids: rutin, luteolin-7-*O*-glucoside (major)	
	- Lignans: pinoresinol, hydroxypinoresinol, acetoxypinoresinol, syringaresinol	
Olive pulp	- Secoiridoids: oleuropein (major in unripe fruit), demethyoleuropein, ligustroside	39, 50, 62, 78 and 152
	- Phenolic acids: verbascoside (major)	
	- Simple phenols: tyrosol and hydroxytyrosol (increasing during fruit ripening)	
	- Flavonoids: luteolin and its glucoside, apigenin, rutin	
	- Anthocyanins: cyanidin derivatives	
	- Carbohydrates: glucose, fructose, mannose	
	- Phytosterols: β-sitosterol	
	- Tocopherols: α-tocopherol	
Seeds	- Secoiridoids: nuzhenide (major), nuzhenide-oleoside, salidroside, oleuropein, demethyloleuropein, ligustroside	39, 50, 78, 152 and 157
	- Fatty acids: oleic acid (major), linoleic acid	
	- Phenolic acids: verbascoside (major)	
	- Lignans: acetoxypinoresinol	
	- Flavonoids: luteolin derivatives	
	- Tocopherols: α (major), β, γ, δ-tocopherols	
	- Sterols: β-sitosterol (major)	
Olive oil	- Fatty acids: oleic acid (major MUFA), linoleic acid (major DUFA)	50, 123, 153 and 160
	-Simple phenols: tyrosol, hydroxytyrosol	
	- Secoiridoids: oleuropein aglycone and derivatives, ligustroside aglycone, oleacein, oleocanthal (major)	
	- Lignans: pinoresinol, acetoxypinoresinol	
	- Tocopherols: α-tocopherol	
	- Phytosterols: β-sitosterol, squalene	

Significantly, the metabolites found within the Oleaceae family possess considerable chemotaxonomic value, acting as pivotal markers that effectively distinguish various species and genera. While certain phenolic acids, such as *trans*-caffeic acid, and flavonoids like luteolin are widely distributed across the plant kingdom, a distinctive feature of the Oleaceae family is the unique presence of secoiridoids.^152^ Indeed, a comprehensive survey has identified a total of 232 secoiridoids and their derivatives across nine genera within Oleaceae, including *Olea*.^78^

Among this rich array, oleuropein and ligustroside stand out as particularly prominent conjugated secoiridoids, found abundantly across all parts of *O. europaea*. These compounds have rightfully attracted considerable scientific interest due to their well-established antioxidant, anti-inflammatory, cardioprotective, neuroprotective, and anticancer properties.^152–154^ Beyond these, other secoiridoids, such as oleoside dimethyl ester and dimethyl oleuropein, have been specifically identified in olive leaves and fruits, respectively.^78^ Interestingly, oxidized secologanoside-type secoiridoids, including secologanoside, oleuroside, 6′-*E-ρ*-coumaroyl-secologanoside, and 6′-*O*-[(2*E*)-2,6-dimethyl-8-hydroxy-2-octenoyloxy]-secologanoside, appear to be exclusively confined to olive leaves.^78^ In contrast, coumarins are predominantly associated with olive stems in addition to oleuropein, whereas glycosylated flavonoids, exemplified by luteolin-7-*O*-glucoside, are distributed across both olive leaves and stems, contributing strong anti-inflammatory, anticancer, and antiviral activities Table 1.^152,155^

The chemical profile of the olive fruit is remarkably complex, influenced by ripeness, variety, and geographical origin. For instance, unripe olives are characterized by high concentrations of oleuropein. Conversely, levels of tyrosol (Tyr), hydroxytyrosol (HTyr), and verbascoside exhibit an inverse relationship, decreasing as the fruit ripens. Notably, nuzhenide in the seeds remains unaffected by this ripening process.^47^ Within the olive fruit skin, pinoresinol, hydroxypinoresinol, and syringaresinol are found in significant abundance.^50^ Furthermore, pentacyclic triterpenes, such as oleanolic and maslinic acids, alongside flavonoids like rutin and luteolin-7-*O*-glucoside,^47^ have been reported for their notable antiproliferative activity.^156^ Olive seeds themselves are a rich source of nuzhenide, a compound first documented by.^47^ Other key components of olive seeds include oleic acid (a MUFA), α-tocopherols (triterpenoids), and β-sitosterol (sterols), while verbascoside is also notably present in both the pulp and seed Table 1.^157^

Olive oil is globally celebrated for its exceptional nutritional and medicinal attributes, particularly its pronounced cardiovascular protective effects. These benefits are largely underpinned by its potent antioxidant, anti-inflammatory, and neuroprotective properties. Its unique composition plays a pivotal role, with a high content of monounsaturated fatty acids (MUFAs), notably oleic acid, forming its backbone. Beyond this, a spectrum of secoiridoids, including oleocanthal, oleacein, oleuropein aglycone, and ligustroside aglycone, along with simple phenolic compounds such as tyrosol and hydroxytyrosol, synergistically contribute to its therapeutic efficacy Table 1.^151,155^

## 7. Isolation yields and optimization strategies of *Olea europaea* L. metabolites

Efficient extraction of *Olea europaea* metabolites is a critical step in maximizing the recovery of bioactive compounds and ensuring reproducible quality for analytical and industrial applications. The isolation yields of bioactive metabolites are influenced by multiple factors depending on the plant part used, harvesting season, geographical origin, extraction methodology, solvent polarity, and processing conditions such as temperature and time. Optimization of these parameters not only enhances recovery but also preserves the structural integrity and biological activity of the metabolites. The following subsections provide a detailed overview of the main determinants of extraction efficiency and summarize recent advances in yield-improvement strategies for olive-derived phenolics.

### 7.1. Method of extraction

Isolation yields of *Olea europaea* metabolites vary considerably depending on the extraction methodology. Conventional maceration and Soxhlet extraction typically provide moderate oleuropein recovery, whereas assisted techniques significantly enhance yields. Ultrasound-assisted extraction (UAE) increased oleuropein yield by approximately 30% compared with maceration, due to cavitation-induced cell wall disruption.^161^ Ball milling-assisted extraction (BMAE), optimized through response surface methodology, achieved recoveries up to 79% ± 0.9% under controlled temperature and solvent ratios.^162^ Microwave-assisted extraction (MAE) and high-pressure-assisted extraction (HPAE) approaches further reduced extraction time from hours to minutes compared to conventional heat-reflux extraction.^163^ Supercritical CO_2_ and enzyme-assisted extraction have also been reported to improve purity and release bound phenolics, offering eco-friendly alternatives for industrial scale-up.^164^

### 7.2. Solvent polarity

Solvent polarity was identified as a critical determinant of extraction efficiency. Comparative studies confirmed that ≥75% aqueous methanol provided the highest total phenolic content (TPC) and ferric reducing antioxidant power (FRAP), while ≥75% ethanol yielded the greatest flavonoid content (TFC) and ABTS radical-scavenging activity. In contrast, pure water consistently resulted in the lowest recovery of phenolic compounds.^163,165^ These findings emphasize the importance of selecting mixed aqueous organic solvents to maximize recovery of bioactive metabolites.

### 7.3. Extraction conditions

Processing conditions, particularly temperature and time, exert a strong influence on yield. Extractions performed at >50 °C increased TPC up to fivefold compared with room temperature, although radical-scavenging capacity did not improve proportionally.^163^ Most phenolic compounds were recovered within one hour under optimized heated or assisted conditions, indicating that prolonged extraction times confer no additional benefit. This highlights the need for careful optimization of extraction parameters to balance efficiency with preservation of bioactivity.

### 7.4. Yield and purity optimization

Collectively, the evidence demonstrates that optimization of methodology, solvent polarity, and extraction conditions can substantially improve yields of *Olea europaea* metabolites. Assisted techniques such as UAE, BMAE, MAE, and HPAE provide higher recovery and shorter extraction times compared to conventional methods, while solvent composition and moderate heating further enhance efficiency. Eco-friendly approaches, including supercritical CO_2_ and enzyme-assisted extraction, offer promising strategies for improving purity and scalability. These findings provide a strong basis for future applications in nutraceutical and pharmaceutical development.^161–165^

## 8. Impurity profile of *Olea europaea* L. metabolites

The scientific and regulatory evaluation of natural products requires not only the identification and quantification of the primary bioactive compounds but also a comprehensive assessment of their impurity profiles. This aspect is critical for the characterization and quality assessment of *Olea europaea* L extracts. The presence of impurities can significantly influence the biological activity, safety, stability, and reproducibility of olive-derived phytochemicals. Such impurities may originate from the source material (*e.g.*, co-extracted plant metabolites), the extraction and purification process (*e.g.*, residual solvents or reagents), or environmental contamination (*e.g.*, heavy metals, pesticides, mycotoxins, polycyclic aromatic hydrocarbons).^166^ Furthermore, environmental factors such as temperature, light exposure, oxygen, and pH can promote degradation reactions leading to the formation of additional impurity species.

Several studies have reported the application of advanced analytical techniques for the identification and quantification of impurities associated with olive metabolites, including HPLC, UHPLC, LC-MS/MS, GC-MS, and NMR spectroscopy.^50^ Among the major bioactive compounds, oleuropein, hydroxytyrosol, tyrosol, and verbascoside may be accompanied by co-extracted impurities such as sugars, proteins, organic acids, lipids, and residual solvents that persist after extraction and purification. Olive oil and pomace are rich in phenolic compounds such as oleuropein, tyrosol, and hydroxytyrosol, which contribute to health benefits. Importantly, these phenolics may reduce the toxicity of contaminants (*e.g.*, mycotoxins, pesticides, PAHs), while at the same time, pollutants can diminish the beneficial effects of phenolics.^166^

Advanced purification strategies, including membrane filtration (micro-, ultra-, and nanofiltration), have been shown to selectively enrich oleuropein while reducing non-phenolic impurities.^167^ Recent studies also highlight the use of polymeric resins and natural deep eutectic solvents (NaDES) to improve purity and minimize solvent residues.^164,167^ Furthermore, valorization approaches applied to olive pomace demonstrated that assisted extraction techniques (*e.g.*, UAE, ASE) combined with purification steps can significantly reduce impurity load while enhancing metabolite recovery.^168^ Collectively, these findings emphasize that impurity profiling and targeted purification are essential to guarantee the safety, efficacy, and scalability of olive-derived nutraceuticals. Therefore, systematic evaluation of impurity profiles is essential for quality control and standardization of olive-derived products. In addition, understanding the interaction between bioactive compounds and impurities of toxic compounds is essential to determine the true health impact of olive products.

## 9. Biological and ecological relevance of predominant olive sources (leaves, fruits, and olive oil)

Although this review primarily focuses on the chemical diversity of *Olea europaea* L., it is equally important to highlight the biological implications of the most abundant metabolite sources: leaves, fruits, and olive oil, as mentioned previously in Fig. 2B.

### 9.1. Olive leaves

Olive leaves are a remarkably rich source of bioactive compounds, including oleuropein, hydroxytyrosol, hydroxytyrosol acetate, and various flavonoids, which collectively contribute to their broad pharmacological profile. A key aspect of their therapeutic action is potent antioxidant activity, primarily mediated *via* the activation of the NRF2 pathway, which in turn boosts cellular resilience against oxidative stress, a major contributor to chronic inflammation. Parallel to this, olive leaf extracts consistently demonstrate strong anti-inflammatory and immunomodulatory effects. These include, for instance, reducing neutrophil extracellular trap (NET) formation and inhibiting COX-1 and COX-2 enzymes, often working synergistically with conventional anti-inflammatory drugs.^169–171^ Notably, their efficacy in inhibiting COX-2 is comparable to ibuprofen, and they also offer gastroprotective benefits by preventing ethanol-induced gastric lesions.^172^

These fundamental mechanisms are central to their therapeutic potential in chronic inflammatory conditions, particularly type 2 diabetes. Oleuropein, in particular, plays a crucial role in mitigating diabetic complications by protecting renal, hepatic, muscular, and retinal tissues, restoring antioxidant defenses, and preserving vascular integrity.^173,174^ By targeting both oxidative and inflammatory pathways, olive leaves effectively disrupt the cycle of chronic inflammation that drives metabolic dysfunction. Their antidiabetic benefits also extend to promoting wound healing, inhibiting polyol pathway enzymes, and modulating both insulin sensitivity and secretion.^175^

Beyond metabolic health, olive leaf extracts exhibit valuable lipid-lowering and anti-atherosclerotic properties, supported by clinical observations of improved blood glucose levels and enhanced nitric oxide-mediated vasodilation.^173,174^ Their neuroprotective potential is equally noteworthy; studies reveal their capacity to modulate neurotrophic and endocannabinoid pathways, alleviate excitotoxic stress, and enhance memory formation, especially under oxidative conditions. Additionally, their demonstrated acetylcholinesterase inhibition suggests a potential role in managing neurodegenerative diseases like Alzheimer's, alongside exhibiting cytotoxic effects against various cancer cell lines.^176,177^

Olive leaves further contribute to skin health by promoting dermal fibroblast proliferation, inhibiting melanin synthesis, and protecting against UVB-induced damage, rendering them valuable in dermatological applications.^178,179^ Their broad antimicrobial spectrum encompasses antibacterial, antifungal, and antiviral activities, providing effectiveness against pathogens such as *Staphylococcus aureus*, *Candida albicans*, *Salmonella typhimurium*, *Enterococcus faecalis*, *Salmonella Paratyphi A*, *Escherichia coli*, *Micrococcus luteus*, and *Bacillus cereus*,^180–183^ as well as *Mycobacterium tuberculosis*,^179^ HIV-1,^184^ and SARS-CoV-2.^185^ Additionally, oleuropein has shown antidiarrheal effects through µ-opioid receptor interactions, complementing standard treatments like loperamide.^186^

In addition to their biomedical applications, olive leaves possess significant agronomic relevance. They serve as indicators of soil fertility within intercropping systems and provide natural protection against agricultural pathogens such as *Fusarium oxysporum* and *Alternaria solani*, thereby supporting sustainable farming practices.^187,188^

**Fig. 28:**
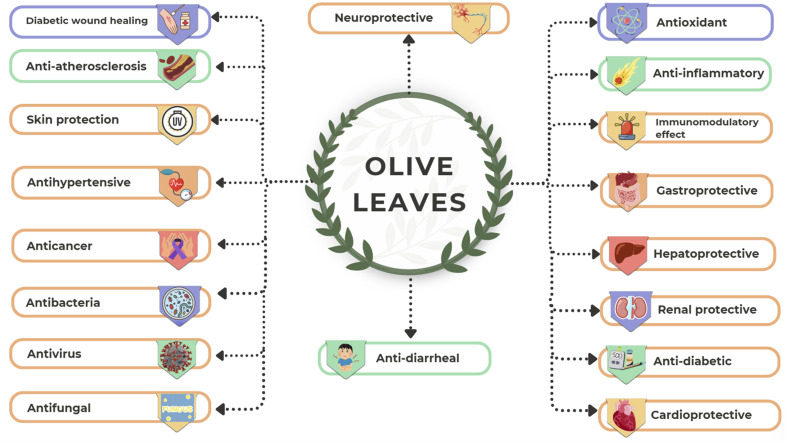
Overview of the therapeutic and biological activities attributed to olive leaves.

While olive leaves represent the richest source of metabolites, making them a primary focus for detailed biological profiling, it is essential to acknowledge that other organs, notably olive fruit and olive oil, also contain bioactive compounds with considerable therapeutic relevance, which deserve further dedicated exploration (Fig. 28).

### 9.2. Olive fruits

Olive fruit extracts exhibit a broad spectrum of biological activities, underscoring their considerable utility in both functional nutrition and therapeutic innovation. Their rich composition, including hydroxytyrosol, tocopherols, carotenoids, phytosterols, and phenolic secoiridoids, confers potent antioxidant, anti-inflammatory, and anti-nociceptive effects through the modulation of key inflammatory mediator pathways such as NF-κB and MAPK.^189,190^ These foundational mechanisms help interrupt the cycle of chronic inflammation that underlies many non-communicable diseases. For instance, olive fruit phenolics have demonstrated neuroprotective effects by reducing oxidative stress and inflammatory burden, while enhancing glutathione levels, thereby mitigating neuronal degeneration in conditions such as stroke, Alzheimer's, and Parkinson's disease.^191^

Within metabolic contexts, comselogoside and 3,4-dihydroxyphenylglycol, two phenolics derived from olive fruit, exhibit inhibitory activity against α-glucosidase and α-amylase. This offers a natural strategy for managing postprandial hyperglycemia and improving glycemic control.^192^ Furthermore, chronic inflammation also contributes to accelerated aging. Here, certain olive cultivars have shown anti-aging potential, evidenced by enhanced stress resilience, improved locomotor function, and reduced cellular aging markers. These effects are mediated through the transcriptional regulation of insulin/IGF-1, DAF-16/FOXO, and SKN-1/Nrf2 184.^193^

Additionally, triterpenoid metabolites, including oleanolic acid and maslinic acid, have demonstrated selective cytotoxic and genotoxic effects against various cancer cell lines, thereby reinforcing their significant potential as natural antitumor agents.^194,195^ Beyond this, olive fruit has been shown to markedly suppress weight gain, reduce epididymal adipose tissue mass, and decrease hepatic lipid accumulation. These beneficial metabolic effects are achieved by modulating the expression of genes crucial for fatty acid transport, lipogenesis, and fatty acid oxidation.^196^ Furthermore, olive extract also possesses antibacterial effects, alongside anti-biofilm potential and minimal hemolytic impact, supporting safe and effective integration into health-promoting formulations.^189,197^

### 9.3. Olive oil

Olive oil, particularly extra virgin olive oil (EVOO), exhibits a wide range of biological activities that extend well beyond its basic nutritional value. These beneficial effects are primarily linked to its abundant phenolic compounds, such as hydroxytyrosol, oleuropein, and oleocanthal, alongside monounsaturated fatty acids like oleic acid. Together, these components play a significant role in modulating lipid metabolism, notably by reducing LDL cholesterol oxidation and enhancing HDL levels, thus improving overall lipid profiles.^198,199^ The European Food Safety Authority^200^ has acknowledged the protective role of olive oil phenolics against oxidative stress in blood lipids, reinforcing its cardiovascular relevance.^201^

Beyond lipid regulation, olive oil exhibits potent anti-inflammatory properties. Oleocanthal, for instance, functions as a natural inhibitor of pro-inflammatory enzymes, demonstrating an effect comparable to that of ibuprofen.^202^ Additionally, it displays antibacterial activity against several pathogenic strains implicated in intestinal and respiratory infections^203^ and may also help prevent cholesterol gallstone formation.^204^ Evidence also suggests that its phenolic compounds stimulate bone formation, highlighting their osteogenic potential for controlling and managing osteoporosis.^205,206^

Moreover, olive oil phenolics exert both anticancer and chemoprotective effects, observed in both *in vitro* and *in vivo* studies across various cancer types. This occurs through mechanisms such as inhibiting cell proliferation, invasion, and oncogene expression, while promoting cancer cell apoptosis.^207–209^ Virgin olive oil also shows neuroprotective and cerebroprotective actions, decreasing infarct size, brain edema, and blood–brain barrier permeability, while improving neurological outcomes and lipid profiles in ischemia-reperfusion models.^210^ Its ability to prevent tau aggregation, counteract β-amyloid toxicity, and reduce brain oxidative stress is relevant to Alzheimer's disease.^145^ Furthermore, olive oil phenolics exhibit antithrombotic activity by inhibiting platelet aggregation, downregulating endothelial adhesion molecules, and decreasing pro-coagulant factors and homocysteine levels.^211,212^ Other noteworthy effects include lowering blood pressure through vasodilation, thereby reducing hypertension risk;^213^ improving glucose metabolism, assisting in the prevention and management of type II diabetes,^214^ and modulating immune responses by decreasing lymphocyte proliferation triggered by B- and T-cell mitogens.^215^

## 10. Toxicological profile, effectiveness, and adverse effects of *Olea europaea* L. extracts and key metabolites

A balanced evaluation of *Olea europaea* metabolites requires integration of three interrelated components: toxicological safety (preclinical and regulatory), clinical effectiveness (human trial outcomes), and adverse effect profiles (observed and potential). This section systematically addresses these aspects for the most relevant olive-derived preparations and compounds.

### 10.1. Olive extract and oleuropein

Olive-derived products are generally recognized as safe, supported by a long history of dietary use. Preclinical studies confirmed the safety of olive leaf extract (OLE), with no mortality or signs of toxicity at a single oral dose of 2000 mg kg^−1^ body weight (bw) of ethanolic OLE in Wistar rats.^216^ A subsequent 28 day subacute study with the same extract at doses up to 400 mg per kg bw per day revealed no biochemical, hematological, or histopathological abnormalities.^216^ Supporting this, a 90 day repeated-dose study of a proprietary OLE (Bonolive™) established a no observed adverse effect level (NOAEL) of 1000 mg per kg per bw per day, with no evidence of genotoxicity in standard assays.^217^ Human clinical studies consistently align with these findings, confirming safety at therapeutic doses. Regarding oleuropein, doses up to 1 g kg^−1^ in albino mice did not cause death.^218^

Clinical evidence highlights the effectiveness of OLE in cardiometabolic health. A meta-analysis of three RCTs (248 participants) showed that supplementation at 1000 mg per day significantly reduced systolic blood pressure^137^ by −11.45 mmHg and diastolic BP by −4.65 mmHg (*p* ≤ 0.001).^219^ Another meta-analysis confirmed mean reductions in systolic BP of −5.78 mmHg over 8 weeks, with efficacy comparable to captopril and no adverse effects on renal or hepatic function.^220^ A larger study (12 RCTs, *n* = 819) reported significant decreases in triglycerides (WMD -9.51 mg dl^−1^; *p* = 0.025) and systolic BP (WMD -3.86 mmHg; *p* = 0.003), with subgroup analyses in hypertensive patients showing improvements in systolic BP (−4.81 mmHg), diastolic BP (−2.45 mmHg), triglycerides (−14.42 mg dl^−1^), total cholesterol (−9.14 mg dl^−1^), and low-density lipoprotein-cholesterol (−4.6 mg dl^−1^),^221^ confirmed by another randomized study^220^ with no evidence of renal or hepatic toxicity. In patients with essential hypertension, aqueous OLE (400 mg × 4 per day for 3 months) significantly reduced BP, with only minor reductions in calcemia and glycemia (*p* < 0.01) and no other side effects.^222^ Beyond blood pressure, a randomized crossover trial in overweight men demonstrated that 12 weeks of OLE supplementation (51.1 mg oleuropein and 9.7 mg hydroxytyrosol per day) improved insulin sensitivity by 15% and β-cell responsiveness by 28% (*p* = 0.013) compared to placebo, and increased fasting interleukin-6, IGFBP-1, and IGFBP-2 concentration, without no significant adverse effects on lipid profile, ambulatory BP, or liver function.^223^ A systematic review of 12 RCTs (683 participants) further highlighted the benefits of oleuropein and hydroxytyrosol supplementation for glucose metabolism, bone health, and cognitive function.^224^

Regarding oleuropein, several biological activities have been documented across *in vitro*, animal, and clinical studies. Anti-infective effects include antiviral activity against multiple viruses such as herpes, hepatitis virus, rotavirus, bovine rhinovirus, canine parvovirus, and feline leukemia virus.^90,225–227^ Anti-inflammatory properties were evaluated in a clinical study of 124 patients with knee osteoarthritis, where daily supplementation with OLE containing 50 mg oleuropein for six months produced clinically meaningful symptom reductions in patients with high baseline pain.^228^ Oleuropein has also demonstrated cardiovascular activity, including vasodilator and hypotensive effects, presumably *via* direct action on vascular smooth muscle.^229^ Central nervous system benefits were observed in Alzheimer's disease model, with reduced amyloid-beta production, enhanced clearance, and NF-κB suppression.^230^ In addition, oleuropein has been implicated in obesity-related mechanisms, upregulating tryptophan metabolism, the precursor of serotonin, and increasing circulating acylcarnitines in serum and urine.^231^

Most clinical trials using standardized OLE extracts at doses of 500–1000 mg per day reported good tolerability, with adverse effects generally mild and infrequent. Side effects such as headache, dizziness, muscle cramps, and mild stomach discomfort occurred in a small proportion of participants, and in a large-scale study (*n* = 663), only 3.2% reported minor events.^219,232^ The Efficacy and Safety of Olive Leaf Extract for Diabetes (ESOLED) trial further confirmed no severe or serious adverse events in adults with type 2 diabetes.^233^ Caution is advised in diabetic patients due to possible hypoglycemic effects, and use is not recommended during pregnancy or lactation. Rare findings include minor reductions in calcemia and glycemia, mood alterations in one elderly patient consuming high-dose OLE (85 mg kg^−1^; hypothesized to be linked to hydroxytyrosol's structural similarity to dopamine), and hepatotoxicity reported in a single animal study.^222,234,235^

Regulatory assessments by the European Food Safety Authority Panel on Additives and Products or Substances used in Animal Feed (FEEDAP) emphasize the importance of standardized extracts, as preparations with ≥20% oleuropein but ∼70% uncharacterized components could not be conclusively evaluated for safety in the context of animal feed.^200^ However, all human clinical trials employed well-characterized preparations (*e.g.*, aqueous decoction, standardized capsules), providing robust evidence for safety at the tested doses.

### 10.2. Hydroxytyrosol (HTyr), tyrosol (Tyr), and verbascoside

Hydroxytyrosol (HTyr), an olive-derived phenol, serves as a precursor of oleuropein, has recognized health benefits, including the EFSA-approved claim that ≥5 mg per day protects LDL from oxidative damage. Long-term toxicological studies established a NOAEL of 500 mg per kg per day for pure HTyr. Consistent *in vitro* evidence from chromosomal aberration and Ames assays confirmed that HTyr and standardized olive extracts (olive juice extract OE20HT, 20% HTyr) are non-genotoxic and non-mutagenic at concentrations far exceeding dietary exposure.^236,237^ Similarly, a standardized aqueous olive pulp extract (HIDROX, containing 50–70% HTyr) demonstrated high oral bioavailability with NOAEL values up to 2000 mg per kg per day and confirmed safety in reproductive and developmental studies, supporting human consumption at doses up to 20 mg per kg per day.^238^ In its assessment of chemically synthesized HTyr as a novel food, the EFSA Panel on Dietetic Products, Nutrition and Allergies (NDA) established a NOAEL of 50 mg per kg per bw per day from a subchronic oral toxicity study. Considering its long history of safe consumption from olives and olive oil, and the absence of adverse effects in both preclinical and human studies, the Panel concluded that HTyr is safe for the general population (excluding young children, pregnant, and breastfeeding women) at the proposed use levels.^239^ These findings are further supported by broader safety assessments from both animal and human studies, which consistently demonstrate low toxicity and favorable metabolic outcomes at physiologically relevant doses. In addition, international regulatory frameworks from the United States, China, and the European Union reinforce the compound's established safe use across food, pharmaceutical, and cosmetic applications.^240^ In contrast, tyrosol has limited toxicological data. Available evidence indicates no genotoxicity in Ames and chromosomal aberration assays, with only weak cytotoxic and pro-apoptotic activity observed at supra-dietary concentrations (≥200 µM). Similarly, verbascoside has shown no genotoxicity but demonstrated cytotoxic and pro-apoptotic effects at higher concentrations (≥100 µM), supporting its safety at dietary levels.^241^

Clinical studies have further evaluated the effectiveness of hydroxytyrosol (HTyr) in humans, complementing the preclinical safety data described above. In a randomized, double-blind, placebo-controlled trial (*n* = 52; 49 completed), adults with overweight and prediabetes received HTyr 15 mg day^−1^ for 16 weeks. Compared with placebo, HTyr significantly reduced oxidized LDL (*p* = 0.045), protein carbonyls (*p* = 0.031), and 8-OHdG (*p* < 0.01), while maintaining total antioxidant status and GPx activity (*p* < 0.01) and lowering IL-6 (*p* = 0.05). These findings suggest that HTyr improves antioxidant, anti-obesity, and anti-inflammatory properties, supporting its potential preventive role against aging-related diseases, with no adverse events reported.^242^ In another clinical study (*n* = 14, mild hyperlipidemia), purified HTyr at 45 mg per day for 8 weeks was safe, with no adverse effects on cardiovascular, hepatic, or renal markers. Significant changes included reduced serum ferritin and folate levels (*p* < 0.05) and a two-fold increase in vitamin C, indicating a physiologically relevant antioxidant effect.^243^ Beyond these trials, HTyr demonstrates a broad spectrum of clinical and experimental activities, including antidiabetic, anti-inflammatory, anti-obesity, and cardiovascular protection, as well as neuroprotective and anticancer properties.^240^ Its strong antioxidant and anti-atherogenic capacity is complemented by antimicrobial, antiviral, and endocrine-modulating effects.^203,244–246^ Evidence also suggests potential benefits in rheumatoid arthritis and autoimmune diseases, with an impact on both chronic and acute inflammatory processes. Furthermore, HTyr exhibits osteoprotective activity, modulates allergen-specific immune responses, and protects keratinocytes against UV-B irradiation, underscoring its broad therapeutic potential.^247^

Unlike hydroxytyrosol, clinical evidence for pure tyrosol remains scarce, with systematic reviews highlighting the absence of dedicated human trials and most data derived from *in vitro* or animal studies.^248^ Preclinical investigations, however, provided valuable insights into tyrosol's biological activity and safety. In a bleomycin-induced lung injury model in rats, oral tyrosol (20–80 mg per kg per day for two weeks) significantly reduced inflammatory cells, IL-6, and oxidative stress markers, while enhancing antioxidant enzyme activity.^249^ Comparable anti-inflammatory effects were observed in beagle dogs with anterior uveitis, where oral tyrosol at 200 mg kg^−1^ twice daily reduced aqueous protein and PGE2 levels, showing efficacy similar to carprofen.^250^ Beyond these findings, tyrosol has been implicated in several cardiometabolic and aging-related pathways.^251^ Experimental evidence suggests potential roles in counteracting hypertension, atherosclerosis, coronary heart disease, chronic heart failure, insulin resistance, and obesity through modulation of CD14 up-regulation.^251^ Tyrosol also reduces oxidative modifications of HDL, preserving its physicochemical integrity, enhancing cholesterol efflux capacity.^252^ Its anti-atherogenic activity has also been linked to inhibition of leukotriene B4 production, improving endothelial function.^253^ Furthermore, tyrosol demonstrates myocardial protection against ischemia-related stress, supporting its potential as an anti-aging therapeutic candidate.^254^

With regard to verbascoside, a randomized, double-blind, phase II study enrolled 100 subjects with cardiovascular risk factors to evaluate supplementation (50 or 100 mg per day for 2 weeks). While the 50 mg dose had no effect, 100 mg per day significantly reduced platelet aggregation induced by arachidonic acid and adenosine diphosphate (from 51 ± 13% to 39 ± 15%; *p* < 0.01), without serious adverse events. These findings support the antiplatelet potential of verbascoside and highlight its possible cardioprotective role, although larger trials are warranted.^255^ In addition to its antiplatelet activity, verbascoside has been reported to exert broad pharmacological effects, including anti-inflammatory, antimicrobial, anticancer, and neuroprotective actions, as well as cardio-, hepato-, nephro-protective and immunomodulatory properties.^256^

In terms of safety and adverse effects, EFSA concluded that hydroxytyrosol is safe for the general population, excluding sensitive groups such as pregnant women and children.^239^ Tyrosol has not been associated with serious adverse events, though systematic reviews emphasize the scarcity of dedicated human safety trials, with most data derived from preclinical models.^248^ Verbascoside, similarly, was well tolerated in a phase II clinical trial at doses of 50–100 mg per day, with no serious adverse events reported, although current evidence is limited to short-term interventions.^255^ Collectively, these findings suggest that all three compounds exhibit promising safety profiles, but further large-scale and long-term studies are warranted to confirm their safety and tolerability.

### 10.3. Oleocanthal (Oc) and oleacein

Oleocanthal, a secoiridoid compound unique to extra virgin olive oil, has a more recently emerging safety profile. An acute oral toxicity study in Swiss albino mice, following OECD Guideline 420, found that a single dose of 10 mg kg^−1^ bw of Oc appeared to be without adverse effects.^257^ Subsequent chronic studies confirmed dose-dependent toxicity, with significant hepatic alterations observed at ≥20 mg kg^−1^ bw, thereby establishing 10 mg kg^−1^ as the non-toxic threshold for preclinical applications.^258^ These findings highlight the necessity of dose optimization for long-term therapeutic use. By contrast, oleacein has no dedicated toxicological studies. Available evidence focuses mainly on its metabolism, tissue distribution, and protective biological effects, underscoring the need for further systematic evaluation.

A single-blind, randomized clinical trial (*n* = 70) evaluated the anti-aging efficacy of a topical formulation containing oleocanthal and oleacein (1% serum applied twice daily for 30 days). Significant wrinkle reductions were observed in women aged 45–79 years (−33.9%), men aged 20–44 years (−51.9%), and men aged 45–79 years (−46.6%), whereas the reduction in younger women (−25.7%) was not statistically significant.^259^ These findings support the dermatological potential of oleocanthal and oleacein, particularly in older adults. In the APRIL study, a randomized, double-blind, controlled crossover clinical trial (*n* = 91 obese, prediabetic adults aged 40–65 years), substitution of dietary fat with extra virgin olive oil enriched in oleocanthal and oleacein for one month significantly reduced interferon-γ (*p* = 0.041), improved antioxidant status, and lowered lipid hydroperoxides compared with common olive oil. Modest reductions in body weight, BMI, and blood glucose were also observed, supporting the metabolic benefits of oleocanthal- and oleacein-rich EVOO.^260^

Beyond their established antioxidant, anti-aging, and antidiabetic properties, oleocanthal and oleacein exhibit additional biological activities. Both compounds demonstrate anti-angiogenic effects, suppressing pathological vascular growth.^261^ Oleocanthal exerts potent anti-inflammatory activity through cytokine modulation, while oleacein displays similar but less extensively studied effects.^262,263^ Oleocanthal has also shown anticancer activity across several tumor models, whereas oleacein contributes to cytokine modulation and apoptosis regulation.^263^ Furthermore, oleocanthal exhibits neuroprotective potential in Alzheimer's disease models.^263^ Recent transcriptomic analyses indicate that both compounds influence immune and metabolic pathways, underscoring their broad pharmacological relevance.^264^

Regarding adverse events, oleocanthal is responsible for the characteristic, temporary, and harmless burning sensation or throat irritation perceived when consuming high-quality extra-virgin olive oil.^265^ Overall, oleocanthal has been reported as well-tolerated in short-term human interventions, with no serious side effects observed. Minor gastrointestinal discomfort has occasionally been mentioned in dietary studies but deemed clinically irrelevant.^263^ Nevertheless, chronic administration at doses ≥20 mg kg^−1^ in mice has been associated with hepatic alterations, highlighting the importance of dose optimization in preclinical settings. In contrast, oleacein has not been associated with specific adverse events in published trials. Available evidence focuses mainly on its metabolism, tissue distribution, and protective outcomes, with short-term studies confirming good tolerability.

Collectively, the totality of evidence from regulatory guidelines, human clinical trials, and compliant animal studies supports the favorable safety profile of *Olea europaea* extracts and their key metabolites. Toxicological data indicate wide margins of safety, with NOAEL values of 1000 mg kg^−1^ bw for OLE, 500 mg kg^−1^ bw for pure HTyr, and up to 10 mg kg^−1^ for oleocanthal in chronic animal studies. Regarding adverse events, oleocanthal is responsible for the characteristic, temporary throat irritation when consuming high-quality extra virgin olive oil, while minor gastrointestinal discomfort has occasionally been reported but deemed clinically irrelevant. Overall, both oleocanthal and oleacein have been well tolerated in short-term human interventions, with no serious side effects observed, although evidence for oleacein remains limited. Nonetheless, high-purity extracts require thorough chemical characterization to ensure long-term safety, particularly for isolated compounds such as tyrosol, oleocanthal, and oleacein.

## 11. Conclusion

*Olea europaea* L. is a very rich source of extremely beneficial phytoconstituents. The biological effects of the plant and its metabolites continue to grab the attention of researchers exploring its activities around different ailments. This review provides a structured, data-driven overview of over 300 metabolites across 15 distinct chemical classes previously reported from various olive sources, including leaves, fruits, olive oil, pomace, flowers, seeds, and stems. Each class is described through its core structural skeletons and position numbering, highlighting structural features that resolve ambiguities between closely related classes (*e.g.*, iridoids *vs.* secoiridoids), subclasses (flavonoids, secoiridoids, and lignans), and structures (oleuricine A *vs.* B and oleocanthal *vs.* oleacein). Secoiridoids, especially oleuropein and ligustroside, represent a predominant class, serving as essential chemotaxonomic indicators and significant contributors to the plant's bioactivity, alongside biosynthetic intermediates such as tyrosol, hydroxytyrosol, and elenolic acid.

Beyond structural annotation, the integration of molecular descriptors (exact mass and molecular formula) and cheminformatics-ready identifiers (InChI, SMILES, PubChem ID, CAS numbers, and ChEMBL) supports downstream computational applications. The five-tier confidence-level scoring system, based on validation methods, shows most compounds clustering around Level 3, reflecting a predominance of moderately validated compounds. Organ-specific profiling reinforces the value of traditionally used sources, leaves, fruits, and olive oil, while drawing attention to the overlooked potential of by-products like pomace. Its notable metabolite content and environmental relevance position it as a promising candidate for sustainable recovery and future therapeutic exploration. Importantly, based on a long history of dietary use of olive fruit, evidence from preclinical and multiple human clinical studies confirms a favorable safety profile for *Olea europaea* leaf extract and its metabolites, reinforcing their suitability for both dietary and therapeutic applications, with no observed hepatotoxicity, nephrotoxicity, or significant adverse effects.

Looking ahead, this resource provides a solid foundation for advancing olive-based therapeutics by integrating detailed phytochemical annotation, biological relevance, and cheminformatic accessibility. It offers a dependable base for pharmacological modeling, virtual screening, and network-based exploration. Future directions may include developing curated molecular fingerprint databases to support *in silico* screening, applying QSAR modeling to examine structure–activity relationships, and expanding experimental validation through NMR and synthesis of reference standards. These efforts will not only accelerate drug discovery and functional food innovation but also enable systems-level exploration of *Olea europaea* metabolites. To improve therapeutic mapping, future studies should prioritize structural confirmation of low-confidence compounds and explore underrepresented plant organs with promising bioactivity. Additionally, long-term human studies on isolated compounds are necessary to ensure safety. Further investigations into lesser-studied olive by-products, such as pomace, will help realize their potential in functional foods or supplements.

## Author contributions

Conceptualization: Seham S. El-Hawary, Mohamed El Raey, and Yasmin Mounir Mohamaden; data curation: Yasmin Mounir Mohamaden; methodology: Yasmin Mounir Mohamaden; visualization: Yasmin Mounir Mohamaden; writing – original draft preparation: Yasmin Mounir Mohamaden; validation: Yasmin Mounir Mohamaden; Reviewing and editing: Seham S. El-Hawary, Mohamed El Raey, Amira Safwat El Senousy, Samar M. Bassam, and Yasmin Mounir Mohamaden; supervision: Seham S. El-Hawary, Mohamed El Raey, Amira Safwat El Senousy, and Samar M. Bassam. All authors have read and agreed to the published version of the manuscript.

## Conflicts of interest

There are no conflicts to declare.

## Abbreviations

bw: Body weight
CAS: Chemical abstracts service
ChEMBL: Chemical database of the european molecular biology laboratory
CI: Confidence interval
CID: PubChem compound identification
3,4-DHPEA: 3,4-Dihydroxyphenyl ethanol (hydroxytyrosol; HTyr)
3,4-DHPEA-EA: 3,4-Dihydroxyphenylethanol elenolic acid (oleuropein aglycone)
3,4-DHPEA-EDA: 3,4-Dihydroxyphenylethanol of decarboxymethyl elenolic acid (oleacein)
DNP: Dictionary of natural products
DUFAs: Diunsaturated fatty acids
EA: Elenolic acid
EDA: Elenolic acid decarboxymethylated
FAs: Fatty acids
GC-MS: Gas chromatography-mass spectrometry
Glc.: Glucoside
Glu.: Glucuronide
*trans*-4-HCA: *trans*-4-Hydroxycinnamic acid
Hex.: Hexoside
*ρ*-HPEA: *ρ*-Hydroxyphenyl ethanol (tyrosol)
*ρ*-HPEA-EA: *ρ*-Hydroxyphenylethanol elenolic acid (ligstroside aglycone)
*ρ*-HPEA-EDA: *ρ*-Hydroxyphenylethanol of decarboxymethyl elenolic acid (oleocanthal)
HPLC-ESI-QTOF-MS: High-performance liquid chromatography coupled to electrospray ionization and quadrupole time-of-flight mass spectrometry
HTyr: Hydroxytyrosol
InChI: IUPAC International chemical identifier
IUPAC: International Union of Pure and Applied Chemistry
KEGG: Kyoto encyclopedia of genes and genomes
MUFAs: Monounsaturated fatty acids
NOAEL: No observed adverse effect level
OECD: Organization for economic co-operation and development
PUFAs: Polyunsaturated fatty acids
QSAR: Quantitative structure–activity relationship
Rha.: Rhamnoside
RP/HPLC-DAD-ESI-QTOF-MS: Reversed-phase high-performance liquid chromatography coupled with diode-array detector, electrospray ionization, and quadrupole-time-of-flight mass spectrometry
RP/HPLC-QTOF-MS: Reversed-phase high-performance liquid chromatography coupled with quadrupole-time-of-flight mass spectrometry
SARS-CoV-2: Severe acute respiratory syndrome – coronavirus 2
SFAs: Saturated fatty acids
SMILES: Simplified molecular input line entry system
Subsp.: Subspecies
TG: Triglyceride
Tyr: Tyrosol
WMD: Weighted mean difference

## Supplementary Material

RA-OLF-D6RA00708B-s001

## Data Availability

No primary research results, software or code have been included and no new data were generated or analysed as part of this review. The data supporting this article, including the comprehensive list of compounds with their chemical identifiers, have been included as part of the supplementary information (SI). Supplementary information: a comprehensive dataset summarizing 355 metabolites across 15 chemical classes. For each compound, key chemical and structural details are included (molecular formula, exact mass, and identifiers such as InChI, SMILES, CID, CAS number, and ChEMBL ID). The file also documents the plant part in which each metabolite is found (leaves, fruits, seeds, flowers, bark, stem, *etc.*). Confidence levels (1–5) are assigned based on the method of validation reported in previous studies. See DOI: https://doi.org/10.1039/d6ra00708b.

## References

[cit1] Hashmi M. A., Khan A., Hanif M., Farooq U., Perveen S. (2015). Traditional Uses, Phytochemistry, and Pharmacology of *Olea europaea* (Olive). Evid. Based Complement. Alternat. Med..

[cit2] Toumi K., Swiatek L., Boguszewska A., Skalicka-Wozniak K., Bouaziz M. (2023). Comprehensive Metabolite Profiling of Chemlali Olive Tree Root Extracts Using LC-ESI-QTOF-MS/MS, Their Cytotoxicity, and Antiviral Assessment. Molecules.

[cit3] Dong W. P., Sun J. H., Liu Y. L., Xu C., Wang Y. H., Suo Z. L., Zhou S. L., Zhang Z. X., Wen J. (2021). Phylogenomic relationships and species identification of the olive genus *Olea* (Oleaceae). J. Systemat. Evol..

[cit4] Rashed S. A., Saad T. I., El-Darier S. M. (2022). Potential aptitude of four olive cultivars as anticancer and antioxidant agents: Oleuropein content. Rendiconti Lincei. Sci. Fis. Nat..

[cit5] A. R. S. United States Department of Agriculture , GRIN-Global: USDA National Plant Germplasm System, https://npgsweb.ars-grin.gov/gringlobal/taxon/taxonomysearch?t=pnlspecies, accessed 2 May 2025

[cit6] Aydin M., Tombuloglu H., Hernandez P., Dorado G., Unver T. (2021). Olive-tree genome sequencing: towards a better understanding of oil biosynthesis. Oil Crop Sci..

[cit7] Kostelenos G. and Kiritsakis A., Olives and olive oil as functional foods: bioactivity, chemistry and processing, 2017, pp. 1–12, 10.1002/9781119135340

[cit8] Wang J., Zhang D., Farooqi T. J. A., Ma L., Deng Y., Jia Z. (2019). The olive (*Olea europaea* L.) industry in China: Its status, opportunities and challenges. Agrofor. Syst..

[cit9] Martínez M., Fuentes M., Franco N., Sánchez J., de Miguel C. (2014). Fatty Acid Profiles of Virgin Olive Oils from the Five Olive‐Growing Zones of Extremadura (Spain). J. Am. Oil Chem. Soc..

[cit10] Batçıoğlu K., Küçükbay F., Alagöz M. A., Günal S., Yilmaztekin Y. (2023). Antioxidant and antithrombotic properties of fruit, leaf, and seed extracts of the Halhalı olive (Olea europaea L.) native to the Hatay region in Turkey. Health.

[cit11] Geana E.-I., Ciucure C. T., Apetrei I. M., Clodoveo M. L., Apetrei C. (2023). Discrimination of olive oil and extra-virgin olive oil from other vegetable oils by targeted and untargeted HRMS profiling of phenolic and triterpenic compounds combined with chemometrics. Int. J. Mol. Sci..

[cit12] Tuck K. L., Hayball P. J. (2002). Major phenolic compounds in olive oil: metabolism and health effects. J. Nutr. Biochem..

[cit13] Quirantes-Pine R., Lozano-Sanchez J., Herrero M., Ibanez E., Segura-Carretero A., Fernandez-Gutierrez A. (2013). HPLC–ESI–QTOF–MS as a powerful analytical tool for characterising phenolic compounds in olive‐leaf extracts. Phytochem. Anal..

[cit14] Dauber C., Carreras T., González L., Gámbaro A., Valdés A., Ibañez E., Vieitez I. (2022). Characterization and incorporation of extracts from olive leaves obtained through maceration and supercritical extraction in Canola oil: Oxidative stability evaluation. Lwt.

[cit15] Tehranizadeh Z. A., Baratian A., Hosseinzadeh H. (2016). Russian olive (Elaeagnus angustifolia) as a herbal healer. Bioimpacts.

[cit16] A. C. C. M. Solution , Search Taxonomy – Arctos, https://arctos.database.museum/taxonomy.cfm, accessed Accessed 4 March 2025

[cit17] Selim S., Albqmi M., Al-Sanea M. M., Alnusaire T. S., Almuhayawi M. S., AbdElgawad H., Al Jaouni S. K., Elkelish A., Hussein S., Warrad M. (2022). Valorizing the usage of olive leaves, bioactive compounds, biological activities, and food applications: A comprehensive review. Front. Nutr..

[cit18] Ammar S., Contreras M. D. M., Gargouri B., Segura-Carretero A., Bouaziz M. (2017). RP-HPLC-DAD-ESI-QTOF-MS based metabolic profiling of the potential *Olea europaea* by-product “wood” and its comparison with leaf counterpart. Phytochem. Anal..

[cit19] Gomez-Gonzalez S., Ruiz-Jimenez J., Priego-Capote F., Luque de Castro M. D. (2010). Qualitative and quantitative sugar profiling in olive fruits, leaves, and stems by gas chromatography-tandem mass spectrometry (GC-MS/MS) after ultrasound-assisted leaching. J. Agric. Food Chem..

[cit20] Paiva-Martins F., Kiritsakis A. (2017). Olive fruit and olive oil composition and their functional compounds, *Olives and Olive Oil as Functional Foods*. Bioactivity, Chemistry and Processing.

[cit21] Mosleh G., Mohagheghzadeh A., Faridi P. (2016). Olive leaf: From tradition to clinic. Trends Pharmacol. Sci..

[cit22] Wodner M., Lavee S., Epstein E. (1988). Identification and seasonal changes of glucose, fructose and mannitol in relation to oil accumulation during fruit development in *Olea europaea* (L.). Sci. Hortic..

[cit23] Attard K. and Lia F., The Antioxidant and Bioactive Potential of Olive Mill Waste, 2024, 10.5772/intechopen.1004127 PMC11049450 38672825

[cit24] Aree T., Jongrungruangchok S. (2018). Structure–antioxidant activity relationship of β-cyclodextrin inclusion complexes with olive tyrosol, hydroxytyrosol and oleuropein: Deep insights from X-ray analysis, DFT calculation and DPPH assay. Carbohydr. Polym..

[cit25] Younis H. E., El Shalakany W. A.-N., Amin S. A. R., Abdel-Reheem M. A. T., Ibrahima H. A. F. (2023). Biological activities and related phenolic compounds content of olive and plum stones ethanolic extract. Egypt. J. Chem..

[cit26] Orak H. H., Karamać M., Amarowicz R., Orak A., Penkacik K. (2019). Genotype-related differences in the phenolic compound profile and antioxidant activity of extracts from olive (*Olea europaea* L.) leaves. Molecules.

[cit27] De La Cruz J. P., Ruiz-Moreno M. I., Guerrero A., Reyes J. J., Benitez-Guerrero A., Espartero J. L., González-Correa J. A. (2015). Differences in the neuroprotective effect of orally administered virgin olive oil (*Olea europaea*) polyphenols tyrosol and hydroxytyrosol in rats. J. Agric. Food Chem..

[cit28] Zhao H., Avena-Bustillos R. J., Wang S. C. (2022). Extraction, Purification and In Vitro Antioxidant Activity Evaluation of Phenolic Compounds in California Olive Pomace. Foods.

[cit29] DellaGreca M., Previtera L., Temussi F., Zarrelli A. (2004). Low‐molecular‐weight components of olive oil mill waste‐waters. Phytochem. Anal..

[cit30] Bianco A., Mazzei R. A., Melchioni C., Romeo G., Scarpati M. L., Soriero A., Uccella N. (1998). Microcomponents of olive oil—III. Glucosides of 2 (3, 4-dihydroxy-phenyl) ethanol. Food Chem..

[cit31] Obied H. K., Bedgood Jr D. R., Prenzler P. D., Robards K. (2007). Chemical screening of olive biophenol extracts by hyphenated liquid chromatography. Anal. Chim. Acta.

[cit32] Pitsillou E., Liang J. J., Beh R. C., Prestedge J., Catak S., Hung A., Karagiannis T. C. (2022). Identification of novel bioactive compounds from *Olea europaea* by evaluation of chemical compounds in the OliveNet™ library: in silico bioactivity and molecular modelling, and in vitro validation of hERG activity. Comput. Biol. Med..

[cit33] Peralbo-Molina A., Priego-Capote F., Luque de Castro M. D. (2012). Tentative identification of phenolic compounds in olive pomace extracts using liquid chromatography-tandem mass spectrometry with a quadrupole-quadrupole-time-of-flight mass detector. J. Agric. Food Chem..

[cit34] Vergine M., Pavan S., Negro C., Nicolì F., Greco D., Sabella E., Aprile A., Ricciardi L., De Bellis L., Luvisi A. (2022). Phenolic characterization of olive genotypes potentially resistant to Xylella. J. Plant Interact..

[cit35] Skaltsounis A.-L., Argyropoulou A., Aligiannis N., Xynos N. (2015). Recovery of high added value compounds from olive tree products and olive processing byproducts, *Olive and Olive*. Oil Bioactive Constituents.

[cit36] Rodriguez-Pérez M. D., Santiago-Corral L., Ortega-Hombrados L., Verdugo C., Arrebola M. M., Martín-Aurioles E., Fernández-Prior M. Á., Bermúdez-Oria A., De La Cruz J. P., González-Correa J. A. (2023). The effect of the extra virgin olive oil minor phenolic compound 3′, 4′-dihydroxyphenylglycol in experimental diabetic kidney disease. Nutrients.

[cit37] Favre H. A. and Powell W. H., Nomenclature of Organic Chemistry: IUPAC Recommendations and Preferred Names 2013, Royal Society of Chemistry, Cambridge, 2014

[cit38] Chemistry, International Union of Pure and Applied Chemistry (IUPAC) , Nomenclature of Organic Chemistry: IUPAC Recommendations and Preferred Names 2013 – Section P‑4, Royal Society of Chemistry/IUPAC, 2013, https://iupac.qmul.ac.uk/BlueBook/PDF/P4.pdf.

[cit39] Ivancic T., Jakopic J., Veberic R., Vesel V., Hudina M. (2022). Effect of Ripening on the Phenolic and Sugar Contents in the Meso- and Epicarp of Olive Fruits (*Olea europaea* L.) Cultivar ‘Leccino’. Agriculture.

[cit40] Silvan J. M., Guerrero-Hurtado E., Gutierrez-Docio A., Alarcon-Cavero T., Prodanov M., Martinez-Rodriguez A. J. (2021). Olive-Leaf Extracts Modulate Inflammation and Oxidative Stress Associated with Human H. pylori Infection. Antioxidants.

[cit41] Difonzo G., Crescenzi M. A., Piacente S., Altamura G., Caponio F., Montoro P. (2022). Metabolomics Approach to Characterize Green Olive Leaf Extracts Classified Based on Variety and Season. Plants.

[cit42] Zhang C., Xin X., Zhang J., Zhu S., Niu E., Zhou Z., Liu D. (2022). Comparative Evaluation of the Phytochemical Profiles and Antioxidant Potentials of Olive Leaves from 32 Cultivars Grown in China. Molecules.

[cit43] Kabbash E. M., Abdel-Shakour Z. T., El-Ahmady S. H., Wink M., Ayoub I. M. (2023). Comparative metabolic profiling of olive leaf extracts from twelve different cultivars collected in both fruiting and flowering seasons. Sci. Rep..

[cit44] Martakos I., Katsianou P., Koulis G., Efstratiou E., Nastou E., Nikas S., Dasenaki M., Pentogennis M., Thomaidis N. (2021). Development of Analytical Strategies for the Determination of Olive Fruit Bioactive Compounds Using UPLC-HRMS and HPLC-DAD. Chemical Characterization of Kolovi Lesvos Variety as a Case Study. Molecules.

[cit45] Zhao Y., Wang S., Pan J., Ma K. (2023). Verbascoside: A neuroprotective phenylethanoid glycosides
with anti-depressive properties. Phytomedicine.

[cit46] Andary C., Wylde R., Laffite C., Privat G., Winternitz F. (1982). Structures of verbascoside and orobanchoside, caffeic acid sugar esters from Orobanche rapum-genistae. Phytochemistry.

[cit47] Servili M., Baldioli M., Selvaggini R., Macchioni A., Montedoro G. (1999). Phenolic compounds of olive fruit: one-and two-dimensional nuclear magnetic resonance characterization of nüzhenide and its distribution in the constitutive parts of fruit. J. Agric. Food Chem..

[cit48] Michel T., Khlif I., Kanakis P., Termentzi A., Allouche N., Halabalaki M., Skaltsounis A.-L. (2015). UHPLC-DAD-FLD and UHPLC-HRMS/MS based metabolic profiling and characterization of different Olea europaea organs of Koroneiki and Chetoui varieties. Phytochem. Lett..

[cit49] Zhu Z., Li X., Zhang Y., Wang J., Dai F., Wang W. (2023). Profiling of phenolic compounds in domestic and imported extra virgin olive oils in China by high performance liquid chromatography-electrochemical detection. Lwt.

[cit50] Olmo-García L., Kessler N., Neuweger H., Wendt K., Olmo-Peinado J. M., Fernández-Gutiérrez A., Baessmann C., Carrasco-Pancorbo A. (2018). Unravelling the distribution of secondary metabolites in Olea europaea L.: exhaustive characterization of eight olive-tree derived matrices by complementary platforms (LC-ESI/APCI-MS and GC-APCI-MS). Molecules.

[cit51] Ryan D., Antolovich M., Prenzler P., Robards K., Lavee S. (2002). Biotransformations of phenolic compounds in *Olea europaea* L. Sci. Hortic..

[cit52] Papageorgiou C. S., Lyri P., Xintaropoulou I., Diamantopoulos I., Zagklis D. P., Paraskeva C. A. (2022). High-Yield Production of a Rich-in-Hydroxytyrosol Extract from Olive (Olea europaea) Leaves. Antioxidants.

[cit53] Ben-Amor I., Musarra-Pizzo M., Smeriglio A., D’Arrigo M., Pennisi R., Attia H., Gargouri B., Trombetta D., Mandalari G., Sciortino M. T. (2021). Phytochemical Characterization of Olea europea Leaf Extracts and Assessment of Their Anti-Microbial and Anti-HSV-1 Activity. Viruses.

[cit54] M Alvarez-Suarez J., Giampieri F., Battino M. (2013). Honey as a source of dietary antioxidants: structures, bioavailability and evidence of protective effects against human chronic diseases. Curr. Med. Chem..

[cit55] Zhu Z., Chen R., Zhang L. (2023). Simple phenylpropanoids: recent advances in biological activities, biosynthetic pathways, and microbial production. Nat. Prod. Rep..

[cit56] Kountouri A. M., Mylona A., Kaliora A. C., Andrikopoulos N. K. (2007). Bioavailability of the phenolic compounds of the fruits (drupes) of Olea europaea (olives): impact on plasma antioxidant status in humans. Phytomedicine.

[cit57] Sharifi-Rad J., Cruz-Martins N., Lopez-Jornet P., Lopez E. P., Harun N., Yeskaliyeva B., Beyatli A., Sytar O., Shaheen S., Sharopov F., Taheri Y., Docea A. O., Calina D., Cho W. C. (2021). Natural Coumarins: Exploring the Pharmacological Complexity and Underlying Molecular Mechanisms. Oxid. Med. Cell. Longev..

[cit58] Moss G. P., Smith P. A. S., Tavernier D. (1995). Glossary of class names of organic compounds and reactivity intermediates based on structure (IUPAC Recommendations 1995). Pure Appl. Chem..

[cit59] Tóth G., Alberti Á., Sólyomváry A., Barabás C., Boldizsár I., Noszál B. (2015). Phenolic profiling of various olive bark-types and leaves: HPLC–ESI/MS study. Ind. Crop. Prod..

[cit60] Hashmi M. A., Khan A., Hanif M., Farooq U., Perveen S. (2015). Traditional Uses, Phytochemistry, and Pharmacology of *Olea europaea* (Olive). Evid. Based Complement. Alternat. Med..

[cit61] Annunziata F., Pinna C., Dallavalle S., Tamborini L., Pinto A. (2020). An overview of coumarin as a versatile and readily accessible scaffold with broad-ranging biological activities. Int. J. Mol. Sci..

[cit62] Dias M. C., Pinto D., Silva A. M. S. (2021). Plant Flavonoids: Chemical Characteristics and Biological Activity. Molecules.

[cit63] I. I. J. C. o. B. Nomenclature , Flavonoid Nomenclature, https://iupac.qmul.ac.uk/flavonoid/, accessed 5 May 2025

[cit64] Çetinkaya S., Akça K. T., Süntar I. (2022). Flavonoids and anticancer activity: Structure–activity relationship. Stud. Nat. Prod. Chem..

[cit65] Kumar S., Pandey A. K. (2013). Chemistry and biological activities of flavonoids: an overview. Sci. World J..

[cit66] Chatzikonstantinou A. V., Giannakopoulou A., Spyrou S., Simos Y. V., Kontogianni V. G., Peschos D., Katapodis P., Polydera A. C., Stamatis H. (2022). Production of hydroxytyrosol rich extract from *Olea europaea* leaf with enhanced biological activity using immobilized enzyme reactors. Environ. Sci. Pollut. Res. Int..

[cit67] Iaria D. L., Chiappetta A., Muzzalupo I. (2015). A De novo Transcriptomic Approach to Identify Flavonoids and Anthocyanins “Switch-Off” in Olive (Olea europaea L.) Drupes at Different Stages of Maturation. Front. Plant Sci..

[cit68] Bensehaila S., Ilias F., Saadi F., Zaouadi N. (2022). Phenolic compounds and antimicrobial activity of olive (*Olea europaea* L.) leaves. Asian J. Dairy Food Res..

[cit69] Angelis A., Mavros P., Nikolaou P. E., Mitakou S., Halabalaki M., Skaltsounis L. (2020). Phytochemical analysis of olive flowers’ hydroalcoholic extract and in vitro evaluation of tyrosinase, elastase and collagenase inhibition activity. Fitoterapia.

[cit70] Martin-Garcia B., Pimentel-Moral S., Gómez-Caravaca A. M., Arráez-Román D., Segura-Carretero A. (2020). Box-Behnken experimental design for a green extraction method of phenolic compounds from olive leaves. Ind. Crop. Prod..

[cit71] Bouaziz M., Chamkha M., Sayadi S. (2004). Comparative study on phenolic content and antioxidant activity during maturation of the olive cultivar Chemlali from Tunisia. J. Agric. Food Chem..

[cit72] Blázovics A., Csorba B., Ferencz A. (2022). The beneficial and adverse effects of phytoestrogens. OBM Integr Compliment Med..

[cit73] Rudrapal M., Khan J., Dukhyil A. A. B., Alarousy R., Attah E. I., Sharma T., Khairnar S. J., Bendale A. R. (2021). Chalcone Scaffolds, Bioprecursors of Flavonoids: Chemistry, Bioactivities, and Pharmacokinetics. Molecules.

[cit74] Baumli J., Antal N., Casoni D., Cimpoiu C. (2023). Use of Secondary Metabolites Profiling and Antioxidant Activity to Unravel the Differences between Two Species of Nettle. Plants.

[cit75] Wang C., Gong X., Bo A., Zhang L., Zhang M., Zang E., Zhang C., Li M. (2020). Iridoids: Research Advances in Their Phytochemistry, Biological Activities, and Pharmacokinetics. Molecules.

[cit76] Castejón M. L., Montoya T., Alarcón-de-la-Lastra C., Sánchez-Hidalgo M. (2020). Potential protective role exerted by secoiridoids from Olea europaea L. in cancer, cardiovascular, neurodegenerative, aging-related, and immunoinflammatory diseases. Antioxidants.

[cit77] Rigane G., Bouaziz M., Sayadi S., Ben Salem R. (2012). Identification and characterization of a new iridoid compound from two-phase Chemlali olive pomace. Eur. Food Res. Technol..

[cit78] Huang Y. L., Oppong M. B., Guo Y., Wang L. Z., Fang S. M., Deng Y. R., Gao X. M. (2019). The Oleaceae family: A source of secoiridoids with multiple biological activities. Fitoterapia.

[cit79] Alu’datt M. H., Rababah T., Alhamad M. N., Gammoh S., Ereifej K., Al-Mahasneh M. A., Naimi O., Hussein N., Kubow S. (2017). Application of olive oil as nutraceutical and pharmaceutical food: composition and biofunctional constituents and their roles in functionality, therapeutic, and nutraceutical properties. Soft Chemistry and Food Fermentation.

[cit80] Alnusaire T. S., Sayed A. M., Elmaidomy A. H., Al-Sanea M. M., Albogami S., Albqmi M., Alowaiesh B. F., Mostafa E. M., Musa A., Youssif K. A., Refaat H., Othman E. M., Dandekar T., Alaaeldin E., Ghoneim M. M., Abdelmohsen U. R. (2021). An In Vitro and In Silico Study of the Enhanced Antiproliferative and Pro-Oxidant Potential of *Olea europaea* L. cv. Arbosana Leaf Extract via Elastic Nanovesicles (Spanlastics). Antioxidants.

[cit81] Termentzi A. , Halabalaki M. and Skaltsounis A. L., UHPLC-DAD-FLD and UHPLC-HRMS/MS based metabolic profiling and characterization of different Olea europaea organs of Koroneiki and Chetoui varieties, Olive and Olive Oil Bioactive Constituents, 2015, pp. 147–177 10.1016/b978-1-63067-041-2.50012-4

[cit82] Abbattista R., Losito I., Calvano C. D., Cataldi T. R. I. (2021). Exploring the Isomeric Precursors of Olive Oil Major Secoiridoids: An Insight into Olive Leaves and Drupes by Liquid-Chromatography and Fourier-Transform Tandem Mass Spectrometry. Foods.

[cit83] Ventura G., Calvano C. D., Abbattista R., Bianco M., De Ceglie C., Losito I., Palmisano F., Cataldi T. R. I. (2019). Characterization of bioactive and nutraceutical compounds occurring in olive oil processing wastes. Rapid Commun. Mass Spectrom..

[cit84] Soler-Rivas C., Espn J. C., Wichers H. J. (2000). Oleuropein and related compounds. J. Sci. Food Agric..

[cit85] Omar S. H. (2010). Oleuropein in olive and its pharmacological effects. Sci. Pharm..

[cit86] Contin A., van der Heijden R., Lefeber A. W. M., Verpoorte R. (1998). The iridoid glucoside secologanin is derived from the novel triose phosphate/pyruvate pathway in a Catharanthus roseus cell culture. FEBS Lett..

[cit87] Obied H. K., Karuso P., Prenzler P. D., Robards K. (2007). Novel secoiNovel secoiridoids with antioxidant activity from Australian olive mill waster. J. Agric. Food Chem..

[cit88] Xie P., Cecchi L., Bellumori M., Balli D., Giovannelli L., Huang L., Mulinacci N. (2021). Phenolic Compounds and Triterpenes in Different Olive Tissues and Olive Oil By-Products, and Cytotoxicity on Human Colorectal Cancer Cells: The Case of Frantoio, Moraiolo and Leccino Cultivars (Olea europaea L.). Foods.

[cit89] Cör Andrejč D., Butinar B., Knez Ž., Tomažič K., Knez Marevci M. (2022). The effect of drying methods and extraction techniques on oleuropein content in olive. Plants.

[cit90] Omar S. H. (2010). Oleuropein in olive and its pharmacological effects. Sci. Pharm..

[cit91] Nikou T., Karampetsou K. V., Koutsoni O. S., Skaltsounis A.-L., Dotsika E., Halabalaki M. (2023). Pharmacokinetics and Metabolism Investigation of Oleocanthal. J. Nat. Prod..

[cit92] Ghorbel A., Wedel S., Kallel I., Cavinato M., Sakavitsi M. E., Fakhfakh J., Halabalaki M., Jansen-Dürr P., Allouche N. (2021). Extraction yield optimization of Oleaster (Olea europaea var. sylvestris) fruits using response surface methodology, LC/MS profiling and evaluation of its effects on antioxidant activity and autophagy in HFF cells. J. Food Meas. Char..

[cit93] Rubio-Senent F., Martos S., Lama-Munoz A., Fernandez-Bolanos J. G., Rodriguez-Gutierrez G., Fernandez-Bolanos J. (2015). Isolation and identification of minor secoiridoids and phenolic components from thermally treated olive oil by-products. Food Chem..

[cit94] Serrano-García I., Olmo-García L., Polo-Megías D., Serrano A., León L., de la Rosa R., Gómez-Caravaca A. M., Carrasco-Pancorbo A. (2022). Fruit phenolic and triterpenic composition of progenies of Olea europaea subsp. cuspidata, an interesting phytochemical source to be included in olive breeding programs. Plants.

[cit95] El Riachy M., Priego-Capote F., León L., Rallo L., Luque de Castro M. D. (2011). Hydrophilic antioxidants of virgin olive oil. Part 2: Biosynthesis and biotransformation of phenolic compounds in virgin olive oil as affected by agronomic and processing factors. Eur. J. Lipid Sci. Technol..

[cit96] Abbattista R., Ventura G., Calvano C. D., Cataldi T. R. I., Losito I. (2021). Bioactive Compounds in Waste By-Products from Olive Oil Production: Applications and Structural Characterization by Mass Spectrometry Techniques. Foods.

[cit97] Melliou E., Zweigenbaum J. A., Mitchell A. E. (2015). Ultrahigh-pressure liquid chromatography triple-quadrupole tandem mass spectrometry quantitation of polyphenols and secoiridoids in california-style black ripe olives and dry salt-cured olives. J. Agric. Food Chem..

[cit98] Panel C. I. R. C. E. , Safety Assessment of Olea Europaea (Olive)-Derived Ingredients as Used in Cosmetics, 2022, https://scholar.googleusercontent.com/scholar?q=cache:PMbvfZ5pK60J:scholar.google.com/+Safety+Assessment+of+Olea+europaea+(Olive)-Derived+Ingredients+as+Used+in+Cosmetics&hl=en&as_sdt=0,5

[cit99] Pérez-Bonilla M., Salido S., van Beek T. A., Waard P. d., Linares-Palomino P. J., Sánchez A., Altarejos J. (2011). Isolation of antioxidative secoiridoids from olive wood (Olea europaea L.) guided by on-line HPLC–DAD–radical scavenging detection. Food Chem..

[cit100] Silva S., Gomes L., Leitão F., Bronze M., Coelho A. V., Boas L. V. (2010). Secoiridoids in olive seed: characterization of nüzhenide and 11-methyl oleosides by liquid chromatography with diode array and mass spectrometry. Grasas Aceites.

[cit101] Damak N., Allouche N., Hamdi B., Litaudon M., Damak M. (2012). New secoiridoid from olive mill wastewater. Nat. Prod. Res..

[cit102] Karković Marković A., Torić J., Barbarić M., Jakobušić Brala C. (2019). Hydroxytyrosol, tyrosol and derivatives and their potential effects on human health. Molecules.

[cit103] Ayoub N. M. , Olive oil oleocanthal and estrogen receptor expression, Olives and Olive Oil in Health and Disease Prevention, 2021, pp. 661–669

[cit104] Montedoro G., Servili M., Baldioli M., Miniati E. (1992). J. Agric. Food Chem..

[cit105] Kamboj S., Singh R. (2022). Arabian J. Sci. Eng..

[cit106] Zeh M., Lorenz P., Kreutzmann P., Schonfeld P. (2008). Redox Rep..

[cit107] Bianco A., Coccioli F., Guiso M., Marra C. (2002). The occurrence in olive oil of a new class of phenolic compounds: hydroxy-isochromans. Food Chem..

[cit108] Togna G. I., Trefiletti G., Guiso M. (2008). 9 Olive Oil Hydroxy-Isochromans, Olive Oil: Minor Constituents and Health.

[cit109] I. I. U. o. P. a. A. Chemistry , Nomenclature of Lignans and Neolignans – Section LG-1.5, https://iupac.qmul.ac.uk/lignan/LG0n1.html#p11, accessed 5 May 2025

[cit110] Zalesak F., Bon D. J. D., Pospisil J. (2019). Pharmacol. Res..

[cit111] Brenes M., Hidalgo F. J., García A., Rios J. J., García P., Zamora R., Garrido A. (2000). J. Am. Oil Chem. Soc..

[cit112] Yang W., Chen X., Li Y., Guo S., Wang Z., Yu X. (2020). Advances in pharmacological activities of terpenoids. Nat. Prod. Commun..

[cit113] Rice P. J., Coats J. R. (1994). Insecticidal properties of several monoterpenoids to the house fly (Diptera: Muscidae), red flour beetle (Coleoptera: Tenebrionidae), and southern corn rootworm (Coleoptera: Chrysomelidae). J. Econ. Entomol..

[cit114] Lukić I., Lukić M., Žanetić M., Krapac M., Godena S., Brkić Bubola K. (2019). Inter-varietal diversity of typical volatile and phenolic profiles of Croatian extra virgin olive oils as revealed by GC-IT-MS and UPLC-DAD analysis. Foods.

[cit115] Boukhebti H., Chaker A. N., Lograda T., Ramdani M. (2015). Chemical and antimicrobial properties of essential oils of *Olea europea*L. Int. J. Toxicol. Pharmacol. Res..

[cit116] Brahmi F., Mechri B., Flamini G., Dhibi M., Hammami M. (2012). Acta Physiol. Plant..

[cit117] Shasha B., Leibowitz J. (1961). On the Oleuropein, the Bitter Principle of Olives1. J. Org. Chem. USSR.

[cit118] Jurisic Grubesic R., Nazlic M., Miletic T., Vuko E., Vuletic N., Ljubenkov I., Dunkic V. (2021). Antioxidants.

[cit119] Suarez Montenegro Z. J., Alvarez-Rivera G., Sanchez-Martinez J. D., Gallego R., Valdes A., Bueno M., Cifuentes A., Ibanez E. (2021). Foods.

[cit120] Campeol E., Flamini G., Cioni P. L., Morelli I., Cremonini R., Ceccarini L. (2003). J. Agric. Food Chem..

[cit121] Mendes A., Azevedo-Silva J., Fernandes J. C. (2022). Pharmaceuticals.

[cit122] Micera M., Botto A., Geddo F., Antoniotti S., Bertea C. M., Levi R., Gallo M. P., Querio G. (2020). Squalene: More than a step toward sterols. Antioxidants.

[cit123] Jimenez-Lopez C., Carpena M., Lourenco-Lopes C., Gallardo-Gomez M., Lorenzo J. M., Barba F. J., Prieto M. A., Simal-Gandara J. (2020). Foods.

[cit124] Stiti N., Hartmann M. A. (2012). J. Lipids.

[cit125] Stiti N., Triki S., Hartmann M. A. (2007). Lipids.

[cit126] Stiti N. , Triki S. and Hartmann M.-A., in Olives and Olive Oil in Health and Disease Prevention, Elsevier, 2010, pp. 211–218

[cit127] Sakouhi F., Absalon C., Sebei K., Fouquet E., Boukhchina S., Kallel H. (2009). Food Chem..

[cit128] Gunduz G., Konuskan D. B. (2023). J. Oleo Sci..

[cit129] Rufino-Palomares E. E., Perez-Jimenez A., Garcia-Salguero L., Mokhtari K., Reyes-Zurita F. J., Peragon-Sanchez J., Lupianez J. A. (2022). Molecules.

[cit130] IUPAC, in Nomenclature of Organic Chemistry: IUPAC Recommendations and Preferred Names 2013 (Blue Book), ed. H. A. Favre and W. H. Powell, RSC Publishing, Cambridge, 2014

[cit131] Moss G. P. (1989). Eur. J. Biochem..

[cit132] Salehi B., Quispe C., Sharifi-Rad J., Cruz-Martins N., Nigam M., Mishra A. P., Konovalov D. A., Orobinskaya V., Abu-Reidah I. M., Zam W., Sharopov F., Venneri T., Capasso R., Kukula-Koch W., Wawruszak A., Koch W. (2020). Front. Pharmacol.

[cit133] Ollivier D., Artaud J., Pinatel C., Durbec J. P., Guerere M. (2003). J. Agric. Food Chem..

[cit134] Rustan A. C. and Drevon C. A., Fatty acids: structures and propertiese LS, 2001, 10.1038/npg.els.0003894

[cit135] Williams M., Morales M. T., Aparicio R., Harwood J. L. (1998). Analysis of volatiles from callus cultures of olive *Olea europaea*. Phytochemistry.

[cit136] Wabaidur S. M., AlAmmari A., Aqel A., Al-Tamrah S. A., Alothman Z. A., Ahmed A. Y. B. H. (2016). Determination of free fatty acids in olive oils by UPHLC–MS. J. Chromatogr. B.

[cit137] Antonia Nunes M., Costa A. S. G., Bessada S., Santos J., Puga H., Alves R. C., Freitas V., Oliveira M. (2018). Sci. Total Environ..

[cit138] Beare-Rogers J. L., Dieffenbacher A., Holm J. V. (2001). Lexicon of lipid nutrition (IUPAC Technical Report). Pure Appl. Chem..

[cit139] Yang Z. , Wang Z., Hollebone B. P., Yang C. and Brown C. E., in Standard Handbook Oil Spill Environmental Forensics, Elsevier, 2016, pp. 565–640

[cit140] Vijakumaran U., Goh N. Y., Razali R. A., Abdullah N. A. H., Yazid M. D., Sulaiman N. (2023). Antioxidants.

[cit141] Choi S.-G. , Won S.-R. and Rhee H.-I., in Olives and Olive Oil in Health and Disease Prevention, Elsevier, 2010, pp. 1375–1383

[cit142] Tarapoulouzi M., Agriopoulou S., Koidis A., Proestos C., Enshasy H. A. E., Varzakas T. (2022). Biomolecules.

[cit143] Fu S., Arraez-Roman D., Segura-Carretero A., Menendez J. A., Menendez-Gutierrez M. P., Micol V., Fernandez-Gutierrez A. (2010). Anal. Bioanal. Chem..

[cit144] Boudour-Benrachou N., Plard J., Pinatel C., Artaud J., Dupuy N. (2017). Adv Food Technol Nutr Sci Open J.

[cit145] Li W., Sperry J. B., Crowe A., Trojanowski J. Q., Smith Iii A. B., Lee V. M. Y. (2009). J. Neurochem..

[cit146] Awwaluddin F., Jamil A. S., Rofida S., Muchlisin M. A. (2023). The Therapeutic Role of Olea europaea in Alcohol Dependence Base in Network. Pharmacology Analysis.

[cit147] Fratianni F., Cozzolino R., Martignetti A., Malorni L., d'Acierno A., De Feo V., da Cruz A. G., Nazzaro F. (2019). Food Nutr. Sci..

[cit148] Masella P., Guerrini L., Angeloni G., Spadi A., Baldi F., Parenti A. (2019). Freezing/storing olives, consequences for extra virgin olive oil quality. Int. J. Refrig..

[cit149] López-López A., Cortés-Delgado A., de Castro A., Sánchez A. H., Montaño A. (2019). Changes in volatile composition during the processing and storage of black ripe olives. Food Res. Int..

[cit150] Mousouri E., Melliou E., Magiatis P. (2014). Isolation of megaritolactones and other bioactive metabolites from ‘megaritiki’table olives and debittering water. J. Agric. Food Chem..

[cit151] Jensen S. R., Franzyk H., Wallander E. (2002). Chemotaxonomy of the Oleaceae: iridoids as taxonomic markers. Phytochemistry.

[cit152] Abdallah M. , Ben Aoun R., Ben Amor M., Touhami I., Habibi M. and Trabelsi N., Agricultural Waste: Environmental Impact, Useful Metabolites and Energy Production, 2023, pp. 331–358

[cit153] Castejón M. L., Montoya T., Ortega-Vidal J., Altarejos J., Alarcón-de-la-Lastra C. (2022). Ligstroside aglycon, an extra virgin olive oil secoiridoid, prevents inflammation by regulation of MAPKs, JAK/STAT, NF-κB, Nrf2/HO-1, and NLRP3 inflammasome signaling pathways in LPS-stimulated murine peritoneal macrophages. Food Funct..

[cit154] Nediani C., Ruzzolini J., Romani A., Calorini L. (2019). Oleuropein, a bioactive compound from Olea europaea L., as a potential preventive and therapeutic agent in non-communicable diseases. Antioxidants.

[cit155] Mohamaden Y. M., El-Hawary S. S., El-Fakharany E. M., El-Maradny Y. A., Raey M. E., El Senousy A. S., Bassam S. M. (2025). Sci. Rep..

[cit156] Juan M. E., Planas J. M., Ruiz-Gutierrez V., Daniel H., Wenzel U. (2008). Antiproliferative and apoptosis-inducing effects of maslinic and oleanolic acids, two pentacyclic triterpenes from olives, on HT-29 colon cancer cells. Br. J. Nutr..

[cit157] Maestri D., Barrionuevo D., Bodoira R., Zafra A., Jiménez-López J., Alché J. d. D. (2019). Nutritional profile and nutraceutical components of olive (Olea europaea L.) seeds. J. Food Sci. Technol..

[cit158] Huang G., Liang J., Chen X., Lin J., Wei J., Huang D., Zhou Y., Sun Z., Zhao L. (2020). . Anal. Methods Chem..

[cit159] Le Tutour B., Guedon D. (1992). Phytochemistry.

[cit160] Papanikolaou C. , Magiatis P. and Melliou E., in Functional Foods, ed. V. S. Lagouri, IntechOpen, Rijeka, 2019, 10.5772/intechopen.81394

[cit161] Cifá D., Skrt M., Pittia P., Di Mattia C., Poklar Ulrih N. (2018). Food Sci. Nutr..

[cit162] Xiang Q., Wang J., Tao K., Huang H., Zhao Y., Jia J., Tan H., Chang H. (2024). Molecules.

[cit163] Dobrinčić A., Repajić M., Garofulić I. E., Tuđen L., Dragović-Uzelac V., Levaj B. (2020). Processes.

[cit164] Fiorito S., Collevecchio C., Spogli R., Epifano F., Genovese S. (2024). Food Chem..

[cit165] Castillo-Correa M., Montalbán-Hernández C., Navarro-Hortal M. D., Peña-Guzmán D., Badillo-Carrasco A., Varela-López A., Hinojosa-Nogueira D., Romero Márquez J. M. (2025). Separations.

[cit166] Schmidt L., Prestes O. D., Augusti P. R., Moreira J. C. F. (2023). Food Biosci..

[cit167] Khemakhem I., Gargouri O. D., Dhouib A., Ayadi M. A., Bouaziz M. (2017). Sep. Purif. Technol..

[cit168] Carlucci V., Ponticelli M., Russo D., Labanca F., Costantino V., Esposito G., Milella L. (2024). Plants.

[cit169] Miyata R., Tabei Y., Abe H., Utsumi K., Fukuzaki S., Azuma S., Takaishi Y., Kato T., Nakajima Y. (2025). Luminescence.

[cit170] Barranco A. M., Arias-de la Rosa I., Cuesta-López L., Martin-Salazar E., Ladehesa-Pineda M. L., Ruiz-Ponce M., Puche-Larrubia M., Llamas-Urbano A., García-Jurado G., Carrasco-Carmonoa Á. (2025). Ann. Rheum. Dis..

[cit171] Jahan N., Mandal M., Rakib I. H., Al Hasan M. S., Mia E., Yana N. T., Alfaifi M., Altemani F. H., Hossan R., Sumaya U. H. (2025). Oleuropein modulates anti-inflammatory activity of celecoxib and ketoprofen through cyclooxygenase pathway: in vivo, in silico and pharmacokinetics approaches. Naunyn-Schmiedeberg’s Arch. Pharmacol..

[cit172] Metlef S., Zidane A., Saadoud M., Gadouche L. (2024). Anti-inflammatory and gastro protective activity of sigoise olive leaves extracts from Algeria: in vivo and in silico evidence. 7th Int. Anatolian Agric. Food Environ. Biol. Congr..

[cit173] Haffani Y. Z., Ben Halim N., Torkhani M., Fouzai C., Rejeb A., Feki M., Zekri S., Boudhrioua N., Darmoul D., Ben Chaouacha-Chekir R. (2025). Targeting Diabetic Complications through Oleuropein-Mediated Enzyme Modulation: A Chemical Biology Study in the Human-Relevant Model Psammomys. ACS Omega.

[cit174] Alsarhan A. A., Khwaldeh A. S., Al-Shawabkeh J. D., Shoiab A. A., Al-Shdefat R., Al-Fawaeir S., Yousef I. (2024). Braz. J. Biol..

[cit175] Al-basher G., Al-otibi F. (2018). Int. J. Pharmacol..

[cit176] Vaccaro F. , Valorisation of medicinal species typical of the biodiversity of the Italian territory for the production of innovative plant products for human health, 2025, 10.25434/vaccaro-federica_phd2025-05-09

[cit177] Ak G., Nilofar N., Bahadirli P., Santanatoglia A., Caprioli G., Sagratini G., Koyuncu I., Kirhan I., Glamočlija J., Stojković D. (2025). Utilizing olive leaves as a rich source of multifunctional bioactive compounds to fight oxidative stress, Alzheimer’s disease, diabetes, and cancer using in vitro, in silico, and bioinformatics techniques. Z. Naturforsch. C Biosci..

[cit178] Shimizu K., Gayatri A., Abdelkarem F. M., Amen Y., Matsumoto M., Nagata M. (2025). Record Nat. Prod..

[cit179] El-Rahmana S. N. A., Abubshaitb S. A., Abubshaitc H. A., Elsharifb A. M., Kamound M. (2023). Braz. J. Biol..

[cit180] Ullah S., Iqbal M. A. (2025). Microchem. J..

[cit181] Salih T. A., Malik S. N., Yaseen S. M., Jameel E. S. (2025). Matrix Sci. Pharma..

[cit182] Tarchi I., Olewnik-Kruszkowska E., Aït-Kaddour A., Bouaziz M. (2025). ACS Omega.

[cit183] Ms H., Fm M., Sm Z. (2025). Mag. Al-Kufa Univ. biol..

[cit184] Lee-Huang S., Zhang L., Huang P. L., Chang Y.-T., Huang P. L. (2003). Biochem. Biophys. Res. Commun..

[cit185] Majrashi T. A., El Hassab M. A., Mahmoud S. H., Mostafa A., Wahsh E. A., Elkaeed E. B., Hassan F. E., Eldehna W. M., Abdelgawad S. M. (2024). PLoS One.

[cit186] Jahan N., Mandal M., Rakib I. H., Al Hasan M. S., Mia E., Hossain M. A., Yana N. T., Ansari S. A., Bappi M. H., Wasaf Hasan A. M. (2025). Drug Dev. Res..

[cit187] Zayani I., Ammari M., Ben Allal L., Daoui K., Bouhafa K. (2025). Vegetos.

[cit188] Cheraghi S., Beiranvand F. (2025). Org. Biomol. Chem..

[cit189] Ali L., Anwar F., Qadir R., Abbas T., Riaz M., Rehman M. F. u. (2025). J. Food Meas. Char..

[cit190] Sahranavard S., Kamalinejad M., Faizi M. (2014). Iran. J. Pharm. Res..

[cit191] Hussain S. Z. , Naseer B., Qadri T., Fatima T. and Bhat T. A., in Fruits Grown in Highland Regions of the Himalayas: Nutritional and Health Benefits, Springer, 2021, pp. 117–129

[cit192] Rubio-Senent F., Bermúdez-Oria A., Lama-Muñoz A., Rodríguez-Gutiérrez G., Fernández-Bolaños J. (2025). Synergistic inhibition of α‐glucosidase and α‐amylase by phenolic compounds isolated from olive oil by‐products. J. Sci. Food Agric..

[cit193] Miao Y., Xu Y., Gao J., Ai X., Duan R., Li R. (2025). Transcriptomic analysis reveals molecular mechanism by which Chinese olive fruit prolongs lifespan of Caenorhabditis. NPJ Sci. Food.

[cit194] Afacan C., Karagoz I. D., Cakir A. (2025). Russ. J. Bioorg. Chem..

[cit195] Ahmad R., Alqathama A., Alam M. M., Riaz M., Abdalla A. N., Aldholmi M., Al− Said H. M., Aljishi F. S., Althomali E. H., Alabdullah M. M. (2023). Chem. Biol. Technol. Agric..

[cit196] Yeh Y.-T., Cho Y.-Y., Hsieh S.-C., Chiang A.-N. (2018). Sci. Rep..

[cit197] Ullah S., Anwar F., ur Rehman M. F., Qadir R., Ansar M. R., Ali H. M., Mustaqeem M., da Silva Dias C. (2023). Arab. J. Chem..

[cit198] Jimenez-Lopez C., Carpena M., Lourenço-Lopes C., Gallardo-Gomez M., Lorenzo J. M., Barba F. J., Prieto M. A., Simal-Gandara J. (2020). Foods.

[cit199] Cicerale S., Lucas L., Keast R. (2010). Int. J. Mol. Sci..

[cit200] Additives E. P. o., Products or Substances used in Animal F., Bampidis V., Azimonti G., Bastos M. d. L., Christensen H., Kos Durjava M., Kouba M., López-Alonso M., López Puente S., Marcon F. (2020). EFSA J..

[cit201] Servili M., Sordini B., Esposto S., Urbani S., Veneziani G., Maio I. D., Selvaggini R., Taticchi A. (2013). Antioxidants.

[cit202] Serra G., Incani A., Serreli G., Porru L., Melis M. P., Tuberoso C. I. G., Rossin D., Biasi F., Deiana M. (2018). Redox Biol..

[cit203] Medina E., De Castro A., Romero C., Brenes M. (2006). J. Agric. Food Chem..

[cit204] Di Ciaula A., Garruti G., Frühbeck G., De Angelis M., De Bari O., Wang D. Q. H., Lammert F., Portincasa P. (2019). Curr. Med. Chem..

[cit205] Chin K.-Y., Ima-Nirwana S. (2016). Int. J. Environ. Res. Public Health.

[cit206] Liu H., Huang H., Li B., Wu D., Wang F., Zheng X. h., Chen Q., Wu B., Fan X. (2014). Clin. Interventions Aging.

[cit207] Song H., Jung J. I., Cho H. J., Park S. Y., Kwon G. T., Kang Y.-H., Lee K. W., Choi M.-S., Park J. H. Y. (2017). Oncotarget.

[cit208] Zubair H., Bhardwaj A., Ahmad A., Srivastava S. K., Khan M. A., Patel G. K., Singh S., Singh A. P. (2017). Nutr. Cancer.

[cit209] Menendez J. A., Vazquez-Martin A., Oliveras-Ferraros C., Garcia-Villalba R., Carrasco-Pancorbo A., Fernandez-Gutierrez A., Segura-Carretero A. (2009). Int. J. Oncol..

[cit210] Mohagheghi F., Bigdeli M. R., Rasoulian B., Zeinanloo A. A., Khoshbaten A. (2010). Sci. World J..

[cit211] Togna G. I., Togna A. R., Franconi M., Marra C., Guiso M. (2003). J. Nutr..

[cit212] Dell Agli M., Maschi O., Galli G. V., Fagnani R., Dal Cero E., Caruso D., Bosisio E. (2008). Br. J. Nutr..

[cit213] Storniolo C. E., Casillas R., Bulló M., Castañer O., Ros E., Sáez G. T., Toledo E., Estruch R., Ruiz-Gutiérrez V., Fitó M. (2017). Eur. J. Nutr..

[cit214] Martinez-Gonzalez M. A., Sayon-Orea C., Bullon-Vela V., Bes-Rastrollo M., Rodriguez-Artalejo F., Yusta-Boyo M. J., Garcia-Solano M. (2022). Clin. Nutr..

[cit215] Aparicio-Soto M., Sánchéz-Hidalgo M., Cárdeno A., Lucena J. M., Gonzáléz-Escribano F., Castillo M. J., Alarcón-de-la-Lastra C. (2017). Mol. Nutr. Food Res..

[cit216] Guex C. G., Reginato F. Z., Figueredo K. C., da da Silva A. R. H., Pires F. B., da Silva Jesus R., Lhamas C. L., Lopes G. H. H., de Freitas Bauermann L. (2018). Regul. Toxicol. Pharmacol..

[cit217] Clewell A. E., Béres E., Vértesi A., Glávits R., Hirka G., Endres J. R., Murbach T. S., Szakonyiné I. P. (2016). Int. J. Toxicol..

[cit218] Petkov V., Manolov P. (1972). Arzneimittelforschung.

[cit219] Lachovicz R., Ferro-Lebres V., Almeida-de-Souza J., Pereira J. A. (2025). Phytother Res..

[cit220] Ismail M. A., Norhayati M. N., Mohamad N. (2021). PeerJ.

[cit221] Razmpoosh E., Abdollahi S., Mousavirad M., Clark C. C. T., Soltani S. (2022). Diabetol. Metab. Syndr..

[cit222] Cherif S., Rahal N., Haouala M., Hizaoui B., Dargouth F., Gueddiche M., Kallel Z., Balansard G., Boukef K. (1996). J. Pharm. Belg..

[cit223] De Bock M., Derraik J. G. B., Brennan C. M., Biggs J. B., Morgan P. E., Hodgkinson S. C., Hofman P. L., Cutfield W. S. (2013). PLoS One.

[cit224] Frumuzachi O., Gavrilaş L. I., Vodnar D. C., Rohn S., Mocan A. (2024). Antioxidants.

[cit225] Toulabi T., Delfan B., Rashidipour M., Yarahmadi S., Ravanshad F., Javanbakht A., Almasian M. (2022). Explore.

[cit226] Salamanca A., Almodóvar P., Jarama I., González-Hedström D., Prodanov M., Inarejos-García A. M. (2021). Antiviral Chem. Chemother..

[cit227] Fredrickson W. R. , Method and composition for antiviral therapy, US Pat., US6117844A, 2000

[cit228] Horcajada M.-N., Beaumont M., Sauvageot N., Poquet L., Saboundjian M., Costes B., Verdonk P., Brands G., Brasseur J., Urbin-Choffray D. (2022). An oleuropein-based dietary supplement may improve joint functional capacity in older people with high knee joint pain: Findings from a multicentre-RCT and post hoc analysis. Ther. Adv. Musculoskelet. Dis..

[cit229] Rabiei Z., Bigdeli M. R., Rasoulian B., Ghassempour A., Mirzajani F. (2012). Phytomedicine.

[cit230] Abdallah I. M., Al-Shami K. M., Yang E., Wang J., Guillaume C., Kaddoumi A. (2022). ACS Chem. Neurosci..

[cit231] Lemonakis N., Mougios V., Halabalaki M., Dagla I., Tsarbopoulos A., Skaltsounis A.-L., Gikas E. (2022). Metabolites.

[cit232] Somerville V., Moore R., Braakhuis A. (2019). Nutrients.

[cit233] Leach M. J., Breakspear I. (2025). Complement. Ther. Clin. Pract..

[cit234] Shaw I. C. (2016). N. Z. Med. J..

[cit235] Arantes-Rodrigues R., Henriques A., Pires M. J., Colaço B., Calado A. M., Rema P., Colaço A., Fernandes T., De la Cruz P. L. F., Lopes C. (2011). Food Chem. Toxicol..

[cit236] Auñon-Calles D., Giordano E., Bohnenberger S., Visioli F. (2013). Pharmacol. Res..

[cit237] Liamin M., Lara M. P., Michelet O., Rouault M., Quintela J. C., Le Bloch J. (2023). Toxicol Rep.

[cit238] Soni M. G., Burdock G. A., Christian M. S., Bitler C. M., Crea R. (2006). Food Chem. Toxicol..

[cit239] Turck D., Bresson J., Burlingame B., Dean T., Fairweather-Tait S., Heinonen M., Hirsch-Ernst K. I., Mangelsdorf I., McArdle H. J., Naska A. (2017). EFSA J..

[cit240] Wang Z., Lei Z., Zhang H., Liu Z., Chen W., Jia Y., Shi R., Wang C. (2025). Int. J. Mol. Sci..

[cit241] Anter J., Tasset I., Demyda-Peyrás S., Ranchal I., Moreno-Millán M., Romero-Jimenez M., Muntané J., de Castro M. D. L., Muñoz-Serrano A., Alonso-Moraga Á. (2014). Mutat. Res. Genet. Toxicol. Environ. Mutagen.

[cit242] Moratilla-Rivera I., Pérez-Jiménez J., Ramos S., Portillo M. P., Martín M. Á., Mateos R. (2025). Hydroxytyrosol supplementation improves antioxidant and anti-inflammatory status in individuals with overweight and prediabetes: A randomized, double-blind, placebo-controlled parallel trial. Clin. Nutr..

[cit243] Lopez-Huertas E., Fonolla J. (2017). Redox Biol..

[cit244] Sogawa K., Kobayashi M., Suzuki J., Sanda A., Kodera Y., Fukuyama M. (2018). Biocontrol Sci..

[cit245] Diallinas G., Rafailidou N., Kalpaktsi I., Komianou A. C., Tsouvali V., Zantza I., Mikros E., Skaltsounis A. L., Kostakis I. K. (2018). Front. Microbiol..

[cit246] Bedoya L. M., Beltrán M., Obregon-Calderon P., García-Pérez J., de la Torre H. E., González N., Pérez-Olmeda M., Auñón D., Capa L., Gómez-Acebo E. (2016). AIDS.

[cit247] Torić J., Barbarić M., Jakobušić Brala C. (2019). Hydroxytyrosol, Tyrosol and Derivatives and Their Potential Effects on Human Health. Molecules.

[cit248] Frumuzachi O., Kieserling H., Rohn S., Mocan A. (2025). CRC Crit. Rev. Food Sci. Nutr..

[cit249] Ekinci E., Karabulut B., Incili C. A., Cankaya E., Seker I., Timurkaan N. (2025). Vet. Sci..

[cit250] Sato K., Kanai K., Ozaki M., Kagawa T., Kita M., Yamashita Y., Nagai N., Tajima K. (2019). J. Vet. Med. Sci..

[cit251] Chang C.-Y., Huang I. T., Shih H.-J., Chang Y.-Y., Kao M.-C., Shih P.-C., Huang C.-J. (2019). J. Funct.Foods.

[cit252] Berrougui H., Ikhlef S., Khalil A. (2015). Extra virgin olive oil polyphenols promote cholesterol efflux and improve HDL functionality. Evid. Based Complement. Alternat. Med..

[cit253] Perona J. S., Cabello-Moruno R., Ruiz-Gutierrez V. (2006). The role of virgin olive oil components in the modulation of endothelial function.. J. Nutr. Biochem..

[cit254] Samuel S. M., Thirunavukkarasu M., Penumathsa S. V., Paul D., Maulik N. (2008). J. Agric. Food Chem..

[cit255] Campo G., Pavasini R., Biscaglia S., Ferri A., Andrenacci E., Tebaldi M., Ferrari R. (2015). Pharmacol. Res..

[cit256] Marčetić M., Bufan B., Drobac M., Antić Stanković J., Arsenović Ranin N., Milenković M. T., Božić D. D. (2025). Antibiotics.

[cit257] Siddique A. B., King J. A., Meyer S. A., Abdelwahed K., Busnena B., El Sayed K. A. (2020). Nutrients.

[cit258] Yang E., Al-Ghraiybah N. F., Alkhalifa A. E., Woodie L. N., Swinford S. P., King J., Greene M. W., Kaddoumi A. (2025). Dose-dependent evaluation of chronic oleocanthal on metabolic phenotypes and organ toxicity in 5xFAD mice. Pharmacol. Res.-Nat. Prod..

[cit259] Nisticò S. P., Greco M. E., Amato S., Bennardo L., Zappia E., Pignataro E., Pellacani G. (2024). Evaluating the impact of oleocanthal and oleacein on skin aging: Results of a randomized clinical study. Medicina.

[cit260] Ruiz-García I., Ortíz-Flores R., Badía R., García-Borrego A., García-Fernández M., Lara E., Martín-Montañez E., García-Serrano S., Valdés S., Gonzalo M. (2023). Clin. Nutr..

[cit261] Marrero A. D., Ortega-Vidal J., Salido S., Castilla L., Vidal I., Quesada A. R., Altarejos J., Martínez-Poveda B., Medina M. A. (2023). Biomed. Pharmacother..

[cit262] Costa V., Costa M., Videira R. A., Andrade P. B., Paiva-Martins F. (2022). Anti-inflammatory activity of olive oil polyphenols—The role of oleacein and its metabolites. Biomedicines.

[cit263] Lozano-Castellón J., López-Yerena A., Rinaldi de Alvarenga J. F., Romero del Castillo-Alba J., Vallverdú-Queralt A., Escribano-Ferrer E., Lamuela-Raventós R. M. (2020). CRC Crit. Rev. Food Sci. Nutr..

[cit264] Wang R., Ganbold M., Ferdousi F., Isoda H. (2025). Comparative transcriptomic and molecular interaction analysis of olive polyphenols: Unraveling their mechanisms in immune regulation and metabolic processes in adipocytes. Comput. Biol. Med..

[cit265] Beauchamp G. K., Keast R. S. J., Morel D., Lin J., Pika J., Han Q., Lee C.-H., Smith A. B., Breslin P. A. S. (2005). Ibuprofen-like activity in extra-virgin olive oil. Nature.

