# Supplementary material for: A comprehensive review of Olea europaea L. metabolites: from structure with confidence scoring to source profiling with biological relevance for therapeutic exploration
Source: RSC Adv. 2026 Jul 7;16(35):34207–46. doi: 10.1039/d6ra00708b (PMC13338935; doi:10.1039/d6ra00708b)
Supplement: RA-016-D6RA00708B-s001 [file RA-016-D6RA00708B-s001.pdf]

This journal is © The Royal Society of Chemistry 2026

The supplementary file accompanying the review article titled **'A Comprehensive Review of *Olea europaea* L. Metabolites: From Structure with Confidence Scoring to Source Profiling with Biological Relevance for Therapeutic Exploration.'**

Yasmin Mounir Mohamaden [a,b *], Seham S. El-Hawary [a, **], Samar M. Bassam [c], Amira Safwat El Senousy [a], Mohamed El Raey [d]

[a] Department of Pharmacognosy, Faculty of Pharmacy, Cairo University, Kasr El Aini, 11562, Cairo, Egypt.

[b] Department of Pharmacognosy, College of Pharmacy, Arab Academy for Science, Technology and Maritime Transport (AASTMT), Alexandria, Egypt

[c] Department of Pharmacognosy and Natural Products, Faculty of Pharmacy, Pharos University in Alexandria, Canal El Mahmoudia street, Beside Green Plaza Complex, 21648, Alexandria, Egypt.

[d] Department of Phytochemistry and Plant Systematics, Pharmaceutical Division, National Research Centre, Dokki, 12622, Cairo, Egypt

[*] Email: corresponding author 1 yasmin.m.mohamaden@pharma.cu.edu.eg; yasmin.m0unir.nm@gmail.com; yasmin.mounir@aast.edu

[**] Email: corresponding author 2 seham.elhawary@pharma.cu.edu.eg

Table S1. Comprehensive profile and identification confidence levels of phytoconstituents reported from *Olea europaea* L.

No.	Compound name	Molecular formula	Exact mass	InChI	SMILES	CID	CAS Number	ChEMBL ID	Part used	References related to the part used	Identification Confidence*	Level 1 ★★★★★<br>Level 2 ★★★★☆<br>Level 3 ★★★☆☆<br>Level 4 ★★☆☆☆<br>Level 5 ★☆☆☆☆	References related to confidence level
**1- Sugars**													
1	D-Mannitol	$C_6H_{14}O_6$	182.07904	InChI=1S/C6H14O6/c7-1-3(9)5(11)6(12)4(10)2-8/h3-12H,1-2H2/t3-,4-,5-,6-/m1/s1	C(C(C(C(C(CO)O)O)O)O)O	6251	69-65-8	CHEMBL689	Roots, wood, fruits, olive oil, leaves, and stems	1-4	2/ Combined NMR 1H NOESY (3.66–3.90 ppm) and MS spectral data HRMS ([M-H]⁻ = 181.073) with literature support.<br><br>3/ Using chromatographic separation [5] combined with MS/MS detection compared to the standard for sugar identification.	Level 2 ★★★★☆<br><br>Level 3 ★★★☆☆	2/ [6]<br><br>3/ [3]
2	D-Sorbitol	$C_6H_{14}O_6$	182.07904	InChI=1S/C6H14O6/c7-1-3(9)5(11)6(12)4(10)2-8/h3-12H,1-2H2/t3-,4+,5-,6-/m1/s1	C(C(C(C(C(CO)O)O)O)O)O	5780	50-70-4	CHEMBL1682	Roots	7	3/ GC-FID matching to standard with no MS validation.	Level 3 ★★★☆☆	3/ [7]
3	D-Gluconic acid	$C_6H_{12}O_7$	196.058305	InChI=1S/C6H12O7/c7-1-2(8)3(9)4(10)5(11)6(12)13/h2-5,7-11H,1H2,(H,12,13)/t2-,3-,4+,5-/m1/s1	C(C(C(C(C(C(=O)O)O)O)O)O)O	10690	526-95-4	CHEMBL464345	Roots, wood, leaves, and fruits	1, 2, 8-10	3/ Identification via HPLC-DAD and LC-ESI-QTOF-MS/MS, relies on chromatographic alignment (Rt) and spectrometric data (HRMS/MSMS) compared to literature data.<br><br>4/ HPLC-MS-based identification without validation.	Level 3 ★★★☆☆<br><br>Level 4 ★★☆☆☆	3/ [1]<br><br>4/ [11]
4	D-Galactonic acid	$C_6H_{12}O_7$	196.058305	InChI=1S/C6H12O7/c7-1-2(8)3(9)4(10)5(11)6(12)13/h2-5,7-11H,1H2,(H,12,13)/t2-,3+,4+,5-/m1/s1	C(C(C(C(C(C(=O)O)O)O)O)O)O	128869	13382-27-9	NF	Wood and flowers	2, 9	4/ HPLC-MS-based identification without validation.	Level 4 ★★☆☆☆	4/ [2]

No.	Compound name	Molecular formula	Exact mass	InChI	SMILES	CID	CAS Number	ChEMBL ID	Part used	References related to the part used	Identification Confidence*	Level 1 ★★★★★<br>Level 2 ★★★★☆<br>Level 3 ★★★☆☆<br>Level 4 ★★☆☆☆<br>Level 5 ★☆☆☆☆	References related to confidence level
**5**	D-(+)-Glucose	$C_6H_{12}O_6$	180.06339	InChI=1S/C6H12O6/c7-1-2-3(8)4(9)5(10)6(11)12-2/h2-11H,1H2/t2-,3-,4+,5-,6?/m1/s1	C(C1C(C(C(C(O1)O)O)O)O)O	5793	2280-44-6	CHEMBL1222250	Fruits, olive oil, leaves, stems, roots, and wood	3, 4, 12-14	3/ Using chromatographic separation [5] combined with MS/MS detection compared to the standard for sugar identification.	Level 3 ★★★☆☆	3/ [3]
**6**	D-Fructose	$C_6H_{12}O_6$	180.06339	InChI=1S/C6H12O6/c7-2-6(11)5(10)4(9)3(8)1-12-6/h3-5,7-11H,1-2H2/t3-,4-,5+,6?/m1/s1	C1[C@H]([C@@H]([C@@H](C(O1)(CO)O)O)O)O	2723872	6347-01-9	CHEMBL2325229	Fruits, olive oil, leaves, stems, and roots	3, 4, 12, 14	3/ Using chromatographic separation [5] combined with MS/MS detection compared to the standard for sugar identification.	Level 3 ★★★☆☆	3/ [3]
**7**	1,5-Anhydroxylitol	$C_5H_{10}O_4$	134.05791	InChI=1S/C5H10O4/c6-3-1-9-2-4(7)5(3)8/h3-8H,1-2H2/t3-,4+,5?	C1[C@H](C([C@H](CO1)O)O)O	446875	39102-78-8	CHEMBL161229	Leaves	15	1/ Isolation, elucidation by 13C NMR spectrum (by DEPT) and 1H NMR, then confirmed by the EI-mass spectrum.	Level 1 ★★★★★	1/ [16]
**8**	D-Sucrose	$C_{12}H_{22}O_{11}$	342.116215	InChI=1S/C12H22O11/c13-1-4-6(16)8(18)9(19)11(21-4)23-12(3-15)10(20)7(17)5(2-14)22-12/h4-11,13-20H,1-3H2/t4-,5-,6-,7-,8+,9-,10+,11-,12+/m1/s1	C([C@@H]1[C@H]([C@@H]([C@H]([C@H](O1)O[C@]2([C@H]([C@@H]([C@H](O2)CO)O)O)CO)O)O)O)O	5988	57-50-1	CHEMBL253582	Fruits, leaves, and roots	3, 12, 17	2/ Combined 1H NOESY NMR (4.07 ppm, 5.40 ppm) and MS spectral data HRMS ($[M-H]^-$ = 341.111) with literature support.	Level 2 ★★★★☆	2/ [6]
**9**	Stachyose	$C_{24}H_{42}O_{21}$	666.221865	InChI=1S/C24H42O21/c25-1-6-10(28)14(32)17(35)21(41-6)39-3-8-11(29)15(33)18(36)22(42-8)40-4-9-12(30)16(34)19(37)23(43-9)45-24(5-27)20(38)13(31)7(2-26)44-24/h6-23,25-38H,1-5H2/t6-,7-,8-,9-,10+,11+,12-,13-,14+,15+,16+,17-,18-,19-,20+,21+,22+,23-,24+/m1/s1	C([C@@H]1[C@@H]([C@@H]([C@H]([C@H](O1)OC[C@@H]2[C@@H]([C@@H]([C@H]([C@H](O2)OC[C@@H]3[C@H]([C@@H]([C@H]([C@H](O3)O[C@]4([C@H]([C@@H]([C@H](O4)CO)O)O)CO)O)O)O)O)O)O)O)O)O)O	439531	470-55-3	CHEMBL1625803	Wood, leaves, and fruits	2, 18	3/ Identification relied on mass spectrometry and MS/MS fragments using RP-HPLC-DAD-ESI-QTOF-MS/MS and UV-Vis data, with literature comparison.	Level 3 ★★★☆☆	3/ [2]

No.	Compound name	Molecular formula	Exact mass	InChI	SMILES	CID	CAS Number	ChEMBL ID	Part used	References related to the part used	Identification Confidence*	Level 1 ★★★★★ Level 2 ★★★★☆ Level 3 ★★★☆☆ Level 4 ★★☆☆☆ Level 5 ★☆☆☆☆	References related to confidence level
**10**	D-Verbascose	$C_{30}H_{52}O_{26}$	828.27469	InChI=1S/C30H52O26/c31-1-7-12(34)17(39)21(43)26(51-7)48-3-9-13(35)18(40)22(44)27(52-9)49-4-10-14(36)19(41)23(45)28(53-10)50-5-11-15(37)20(42)24(46)29(54-11)56-30(6-33)25(47)16(38)8(2-32)55-30/h7-29,31-47H,1-6H2/t7-,8-,9-,10-,11-,12+,13+,14+,15-,16-,17+,18+,19+,20+,21-,22-,23-,24-,25+,26+,27+,28+,29-,30+/m1/s1	C([C@@H]1[C@@H]([C@@H]([C@H]([C@H](O1)OC[C@@H]2[C@@H]([C@@H]([C@H]([C@H](O2)OC[C@@H]3[C@@H]([C@@H]([C@H]([C@H](O3)OC[C@@H]4[C@H]([C@@H]([C@H]([C@H](O4)O[C@]5([C@H]([C@@H]([C@H](O5)CO)O)O)CO)O)O)O)O)O)O)O)O)O)O)O	441434	546-62-3	NF	Wood and leaves	[2, 12, 19]	3/ Identification relied on mass spectrometry and MS/MS fragments using RP-HPLC-DAD-ESI-QTOF-MS/MS and UV-vis data, with literature comparison.	Level 3 ★★★☆☆	3/ [2]

2- Simple phenols

a. Phenylethanols and glycosides (simple phenols)

No.	Compound name	Molecular formula	Exact mass	InChI	SMILES	CID	CAS Number	ChEMBL ID	Part used	References related to the part used	Identification Confidence*	Level	References related to confidence level
**1**	Tyrosol (hydroxyphenylethanol/ HPEA)	$C_8H_{10}O_2$	138.06808	InChI=1S/C8H10O2/c9-6-5-7-1-3-8(10)4-2-7/h1-4,9-10H,5-6H2	C1=CC(=CC=C1CCO)O	10393	501-94-0	CHEMBL53566	Pomace, small branches, leaves, stems, fruits, olive oil (VOO& EVOO), seeds, bark, wood, roots, OOMW, and OOMs	[12, 20-27]	2/ Analysis by LC-SPE-NMR with Rt matching (7.0 min), structural elucidation via 1D (1H and 13C) and 2D NMR (TOCSY) compared with authentic standard.<br>3/ Identification by GC-MS and confirmed by 1H NMR and 1D (COSY).	Level 2 ★★★★☆<br>Level 3 ★★★☆☆	2/ [28]<br>3/ [29]
**2**	Tyrosol 8-*O*-glucoside (salidroside)	$C_{14}H_{20}O_7$	300.120905	InChI=1S/C14H20O7/c15-7-10-11(17)12(18)13(19)14(21-10)20-6-5-8-1-3-9(16)4-2-8/h1-4,10-19H,5-7H2/t10-,11-,12+,13-,14-/m1/s1	C1=CC(=CC=C1CCO[C@H]2[C@@H]([C@H]([C@@H]([C@H](O2)CO)O)O)O)O	159278	10338-51-9	CHEMBL465208	Pomace, leaves, and OOMW	[30-32]	3/ Identification by GC-MS and confirmed by 1H NMR and 1D (COSY).	Level 3 ★★★☆☆	3/ [29]

No.	Compound name	Molecular formula	Exact mass	InChI	SMILES	CID	CAS Number	ChEMBL ID	Part used	References related to the part used	Identification Confidence*	Level 1 ★★★★★ Level 2 ★★★★☆ Level 3 ★★★☆☆ Level 4 ★★☆☆☆ Level 5 ★☆☆☆☆	References related to confidence level
**3**	Tyrosol acetate	$C_{10}H_{12}O_3$	180.078645	InChI=1S/C10H12O3/c1-8(11)13-7-6-9-2-4-10(12)5-3-9/h2-5,12H,6-7H2,1H3	CC(=O)OCCC1=CC=C(O)C=C1	NF	NF	NF	Olive oil (VOO& EVOO)	28, 33	2/ Analysis by LC-SPE-NMR with Rt matching (18.8 min), structural elucidation via 1D (1H and 13C) and 2D NMR (TOCSY) compared with synthesized analogs (hydroxytyrosol acetate).	Level 2 ★★★★☆	2/ [28]
**4**	Hydroxytyrosol (3,4-dihydroxyphenyl ethanol; 3,4-DHPEA)	$C_8H_{10}O_3$	154.062995	InChI=1S/C8H10O3/c9-4-3-6-1-2-7(10)8(11)5-6/h1-2,5,9-11H,3-4H2	C1=CC(=C(C=C1CCO)O)O	82755	10597-60-1	CHEMBL1950045	Roots, flowers, pomace, small branches, stems, leaves, olive oil (VOO& EVOO), fruits, seeds, wood, bark, and OOMW	1, 9, 20-22, 24, 26, 27, 34, 35	2/ Analysis by LC-SPE-NMR with Rt matching (7.0 min), structural elucidation via 1D (1H and 13C) and 2D NMR (TOCSY) compared with authentic standard.	Level 2 ★★★★☆	2/ [28]
**5**	3-β-D-Hydroxytyrosol glucoside (3,4-DHPE glucoside)	$C_{14}H_{20}O_8$	316.11582	InChI=1S/C14H20O8/c15-4-3-7-1-2-8(17)9(5-7)21-14-13(20)12(19)11(18)10(6-16)22-14/h1-2,5,10-20H,3-4,6H2/t10-,11-,12+,13-,14-/m1/s1	OCCC1=CC(OC2C(O)C(O)C(O)C(O2)CO)=C(O)C=C1	5315870	142542-89-0	NF	Olive oil and OOMW	32, 36	3/ Identification via comparing its 1H-NMR and 13C-NMR spectral data to literature values.	Level 3 ★★★☆☆	3/ [36]
**6**	4-β-D-Hydroxytyrosol glucoside (3,4-DHPE glucoside)	$C_{14}H_{20}O_8$	316.11582	InChI=1S/C14H20O8/c15-4-3-7-1-2-9(8(17)5-7)21-14-13(20)12(19)11(18)10(6-16)22-14/h1-2,5,10-20H,3-4,6H2/t10-,11-,12+,13-,14-/m1/s1	C1=CC(=C(C=C1CCO)O)O[C@H]2[C@@H]([C@H]([C@@H]([C@H](O2)CO)O)O)O	6453057	54695-80-6	NF	Roots, pomace, leaves, stems, fruits, wood, bark, seeds, OOMW, and olive oil	1, 2, 8, 17, 30, 34, 37-40	3/ Isolation via PHPLC; structural confirmation via NMR and MS; matched to literature.	Level 3 ★★★☆☆	3/ [41]
**7**	8-β-D-Hydroxytyrosol glucoside (3,4-DHPE glucoside)	$C_{14}H_{20}O_8$	316.11582	InChI=1S/C14H20O8/c15-6-10-11(18)12(19)13(20)14(22-10)21-4-3-7-1-2-8(16)9(17)5-7/h1-2,5,10-20H,3-4,6H2/t10-,11-,12+,13-,14-/m1/s1	C1=CC(=C(C=C1CCO[C@H]2[C@@H]([C@H]([C@@H]([C@H](O2)CO)O)O)O)O)O	5316821	76873-99-9	CHEMBL1689261	Olive oil, leaves, and seeds	32, 42	3/ Identification relies on spectroscopic data (1H-NMR) compared to literature values.	Level 3 ★★★☆☆	3/ [29]

No.	Compound name	Molecular formula	Exact mass	InChI	SMILES	CID	CAS Number	ChEMBL ID	Part used	References related to the part used	Identification Confidence*	Level 1 ★★★★★ Level 2 ★★★★☆ Level 3 ★★★☆☆ Level 4 ★★☆☆☆ Level 5 ★☆☆☆☆	References related to confidence level
**8**	Hydroxytyrosol glucoside derivative	$C_{24}H_{34}O_{10}$	482.2152	—	—	NF	NF	NF	Stems, roots, and pomace	[40, 43]	4/ Identification via UHPLC-HRMS/MS (Rt 14.9 min.), the identification likely depends on hypothetical fragmentation pathways and chemotaxonomic plausibility (e.g., known hydroxytyrosol *m/z* 315.1073 conjugates in *Olea*) and 297.0970 [hydroxytyrosol-H-H_2O]$^-$.	Level 4 ★★☆☆☆	4/ [40]
**9**	Hydroxytyrosol diglucoside	$C_{20}H_{30}O_{13}$	478.168644	InChI=1S/C20H30O13/c21-6-11-13(24)15(26)18(29)20(32-11)31-7-12-14(25)16(27)17(28)19(33-12)30-4-3-8-1-2-9(22)10(23)5-8/h1-2,5,11-29H,3-4,6-7H2	OC1=C(O)C=CC(CCOC2C(O)C(O)C(O)C(COC3C(O)C(O)C(O)C(CO)O3)O2)=C1	NF	NF	NF	Pomace	[25]	4/ Identification based on LC-QqTOF-MS, confirmed by characteristic fragments (e.g., sequential loss of two glucose units [162 Da each], hydroxytyrosol aglycone [*m/z* 153]).	Level 4 ★★☆☆☆	4/ [25]
**10**	Homovanillyl alcohol	$C_9H_{12}O_3$	168.078645	InChI=1S/C9H12O3/c1-12-9-6-7(4-5-10)2-3-8(9)11/h2-3,6,10-11H,4-5H2,1H3	COC1=C(C=CC(=C1)CCO)O	16928	2380-78-1	CHEMBL3747068	Leaves, wood, bark, and olive oil (VOO& EVOO)	[2, 33, 34]	2/ Analysis by LC-SPE-NMR with Rt matching (7.0 min), structural elucidation via 1D (1H and 13C) and 2D NMR (TOCSY) compared with authentic standard.	Level 2 ★★★★☆	2/ [28]
**11**	Hydroxytyrosol acetate	$C_{10}H_{12}O_4$	196.07356	InChI=1S/C10H12O4/c1-7(11)14-5-4-8-2-3-9(12)10(13)6-8/h2-3,6,12-13H,4-5H2,1H3	CC(=O)OCCC1=CC(=C(C=C1)O)O	155240	69039-02-7	CHEMBL3093465	Fruit, pomace, leaves, and olive oil (EVOO)	[11, 25, 44, 45]	2/ Analysis by LC-SPE-NMR with Rt matching (15.0 min), structural elucidation via 1D (1H and 13C) and 2D NMR (TOCSY) compared with synthesized analogs (e.g., acetylation of hydroxytyrosol) and literature data.	Level 2 ★★★★☆	2/ [28]
**12**	Methyl malate-β-hydroxytyrosol ester / (β-hydroxytyrosyl ester of methyl malate)	$C_{13}H_{16}O_7$	284.089605	InChI=1S/C13H16O7/c1-19-13(18)11(16)7-12(17)20-5-4-8-2-3-9(14)10(15)6-8/h2-3,6,11,14-16H,4-5,7H2,1H3	O=C(OCCC1=CC(O)=C(O)C=C1)CC(O)C(OC)=O	NF	NF	NF	Fruits and seeds	[33, 46]	1/ Physically isolated, structural elucidation via 1D (1H and 13C) and 2D (COSY, HSQC, HMBC) NMR, molecular formula verification via HRMS, then comparison to literature [spectral data for related hydroxytyrosol/tyrosol derivatives (e.g., fragmentation patterns, functional groups)].	Level 1 ★★★★★	1/ [46]

b. Other simple phenols

No.	Compound name	Molecular formula	Exact mass	InChI	SMILES	CID	CAS Number	ChEMBL ID	Part used	References related to the part used	Identification Confidence*	Level 1 ★★★★★<br>Level 2 ★★★★☆<br>Level 3 ★★★☆☆<br>Level 4 ★★☆☆☆<br>Level 5 ★☆☆☆☆	References related to confidence level
**1**	Catechol	$C_6H_6O_2$	110.03678	InChI=1S/C6H6O2/c7-5-3-1-2-4-6(5)8/h1-4,7-8H	C1=CC=C(C(=C1)O)O	289	120-80-9	CHEMBL280998	Leaves, stems, branches, fruits, and seeds	35, 47	2/ Analyzed using RPLC-DAD-ESI-MS, confirmed with chemical standard, and supported by Rt, UV spectra, and MS data.<br><br>3/ Identification via GC (Rt = 29.45 min., MS and MS2 compared with the NIST database and literature references), and supported by FTIR/NMR data, but lacks direct structural proof.	Level 2 ★★★★☆<br><br>Level 3 ★★★☆☆	2/ [32]<br><br>3/ [48]
**2**	4-Methylcatechol	$C_7H_8O_2$	124.05243	InChI=1S/C7H8O2/c1-5-2-3-6(8)7(9)4-5/h2-4,8-9H,1H3	CC1=CC(=C(C=C1)O)O	9958	452-86-8	CHEMBL158766	Fruits, and OOMW	49, 50	3/ GC-MS provided HRMS and MS/MS data. Compared with the standard, through calibration with pure compounds, confirmed Rt and UV spectrum, HRMS, and ^{1}H-NMR data.	Level 3 ★★★☆☆	3/ [51]
**3**	3,4-Dihydroxyphenylgl-ycol (DHPG) / 1-(3,4-Dihydroxyphenyl)-1,2-ethanediol	$C_8H_{10}O_4$	170.05791	InChI=1S/C8H10O4/c9-4-8(12)5-1-2-6(10)7(11)3-5/h1-3,8-12H,4H2	C1=CC(=C(C=C1C(CO)O)O)O	5324679	55254-51-8	NF	Flowers, fruit, OOMW, and olive oil (EVOO)	9, 52-54	2/Co-chromatography (HPLC/UV) compared with commercial DHPG standard, spectroscopic confirmation via 1D (1H and 13C) and 2D NMR was carried out.	Level 1 ★★★★★	2/ [55]
					**3- Phenolic acids**								
	**a. Cinnamic acids and derivatives**												
**1**	*trans*-Cinnamic acid	$C_9H_8O_2$	148.0527	InChI=1S/C9H8O2/c10-9(11)7-6-8-4-2-1-3-5-8/h1-7H,(H,10,11)/b7-6+	C1=CC=C(C=C1)C=CC(=O)O	444539	140-10-3	CHEMBL27246	Pomace, fruits, seeds, leaves, stems, branches, olive oil (VOO& EVOO), and OOMW	30, 33, 35, 47, 56-58	2/ Identification via GC-MS matched a commercial standard after derivatization, confirmed by 1H and 13C NMR, and structural validation via DEPT-135 and HRMS.	Level 2 ★★★★☆	2/ [59]
**2**	*trans*-Caffeic acid (trans-3,4-DHCA)	$C_9H_8O_4$	180.04226	InChI=1S/C9H8O4/c10-7-3-1-6(5-8(7)11)2-4-9(12)13/h1-5,10-11H,(H,12,13)/b4-2+	C1=CC(=C(C=C1C=CC(=O)O)O)O	689043	501-16-6	CHEMBL145	Pomace, leaves, branches, fruits, seeds, olive oil, wood, bark, and OOMW	60 2, 23, 30, 33, 34, 58	2/ Identified by HPLC (Rt 25.8 min.) matched to an authentic standard; UV detection at 335 nm.	Level 2 ★★★★☆	2/ [61]

No.	Compound name	Molecular formula	Exact mass	InChI	SMILES	CID	CAS Number	ChEMBL ID	Part used	References related to the part used	Identification Confidence*	Level 1 ★★★★★<br>Level 2 ★★★★☆<br>Level 3 ★★★☆☆<br>Level 4 ★★☆☆☆<br>Level 5 ★☆☆☆☆	References related to confidence level
**3**	Caffeoyl-β-D-glucose (caffeic acid 3-*O*-glucoside)	$C_{15}H_{18}O_9$	342.095085	InChI=1S/C15H18O9/c16-6-10-12(20)13(21)14(22)15(24-10)23-9-5-7(1-3-8(9)17)2-4-11(18)19/h1-5,10,12-17,20-22H,6H2,(H,18,19)/b4-2+/t10-,12-,13+,14-,15-/m1/s1	C1=CC(=C(C=C1C=CC(=O)O)O)OC2C(C(C(C(O2)CO)O)O)O)O	5281759	24959-81-7	NF	Pomace and OOMW	8, 62	3/ Identification based on RP-HPLC-ESI-TOF/MS, and comparison with literature data.	Level 3 ★★★☆☆	3/ [62]
**4**	*ρ*-4-Coumaric acid	$C_9H_8O_3$	164.047345	InChI=1S/C9H8O3/c10-8-4-1-7(2-5-8)3-6-9(11)12/h1-6,10H,(H,11,12)/b6-3+	C1=CC(=CC=C1C=CC(=O)O)O	637542	501-98-4	CHEMBL66879	Pomace, leaves, stems, branches, fruits, bark, wood, seeds, olive oil (VOO& EVOO), and OOMW	12, 21, 23, 30, 35, 47, 58, 63, 64	2/ Analysis by LC-SPE-NMR with Rt matching (12.7 min), structural elucidation via 1D (1H and 13C) and 2D NMR (TOCSY) compared with authentic standard.	Level 2 ★★★★☆	2/ [28]
**5**	Coumaric acid-4-*O*- glucoside	$C_{15}H_{18}O_8$	326.10017	InChI=1S/C15H18O8/c16-7-10-12(19)13(20)14(21)15(23-10)22-9-4-1-8(2-5-9)3-6-11(17)18/h1-6,10,12-16,19-21H,7H2,(H,17,18)/b6-3+/t10-,12-,13+,14-,15-/m1/s1	C1=CC(=CC=C1C=CC(=O)O)OC2C(C(C(C(O2)CO)O)O)O	9840292	14364-05-7	CHEMBL3978496	Fruit and leaves	62, 65	3/ Detection via HPLC-DAD/MS-MS.	Level 4 ★★☆☆☆	3/ [65]
**6**	Ferulic acid (trans-3-M,4-HCA)	$C_{10}H_{10}O_4$	194.05791	InChI=1S/C10H10O4/c1-14-9-6-7(2-4-8(9)11)3-5-10(12)13/h2-6,11H,1H3,(H,12,13)/b5-3+	COC1=C(C=CC(=C1)C=CC(=O)O)O	445858	537-98-4	CHEMBL32749	Pomace, leaves, branches, bark, fruits, seeds, olive oil (VOO& EVOO), and OOMW	25, 33, 34, 47, 57, 58, 60, 63, 64	2/ Identification by HPLC (Rt 36.8 min.) matched to an authentic standard; UV detection at 335 nm.	Level 2 ★★★★☆	2/ [61]
**7**	Feruloyl-glucose	$C_{16}H_{20}O_9$	356.110735	InChI=1S/C16H20O9/c1-25-12-6-8(2-4-9(12)18)3-5-10(19)13(21)15(23)16(24)14(22)11(20)7-17/h2-6,11,14-18,20,22-24H,7H2,1H3/b5-3+/t11-,14-,15+,16+/m1/s1	COC1=C(C=CC(=C1)C=CC(=O)C(=O)C(C(C(C(CO)O)O)O)O)O	22842356	NF	NF	Leaves and pomace	66, 67	4/ Identification via HPLC-DAD ESI/MS-TOF.	Level 4 ★★☆☆☆	4/ [66]
**8**	Sinapic acid	$C_{11}H_{12}O_5$	224.068475	InChI=1S/C11H12O5/c1-15-8-5-7(3-4-10(12)13)6-9(16-2)11(8)14/h3-6,14H,1-2H3,(H,12,13)/b4-3+	COC1=CC(=CC(=C1O)OC)C=CC(=O)O	637775	530-59-6	CHEMBL109341	Olive oil (VOO), fruits, pomace, and OOMW	58, 60	2/ Identification based on chromatography RPLC-DAD-ESI-MS with a chemical standard, supported by retention time and UV data, but incomplete MS validation.<br><br>3/ Identification via 1D (1H and 13C) and 2D NMR (gCOSY,	Level 2 ★★★★☆<br><br>Level 3 ★★★☆☆	2/ [32]<br><br>3/ [68]

No.	Compound name	Molecular formula	Exact mass	InChI	SMILES	CID	CAS Number	ChEMBL ID	Part used	References related to the part used	Identification Confidence*	Level 1 ★★★★★<br>Level 2 ★★★★☆<br>Level 3 ★★★☆☆<br>Level 4 ★★☆☆☆<br>Level 5 ★☆☆☆☆	References related to confidence level
											gNOESY, gHMQC, and gHMBC) compared to literature.		
**9**	Sinapic acid 4-*O*-glucoside	$C_{17}H_{22}O_{10}$	386.1213	InChI=1S/C17H22O10/c1-24-9-5-8(3-4-12(19)20)6-10(25-2)16(9)27-17-15(23)14(22)13(21)11(7-18)26-17/h3-6,11,13-15,17-18,21-23H,7H2,1-2H3,(H,19,20)/b4-3+/t11-,13-,14+,15-,17+/m1/s1	COC1=CC(=CC(=C1OC2C(C(C(C(O2)CO)O)O)O)OC)C=CC(=O)O	5280550	117405-52-4	CHEMBL3916405	Leaves	[66]	4/ Identification via HPLC-DAD ESI/MS-TOF.	Level 4 ★★☆☆☆	4/ [66]
**10**	*trans*-4,5-Dicaffeoylquinic acid (isochlorogenic acid C)	$C_{25}H_{24}O_{12}$	516.12678	InChI=1S/C25H24O12/c26-15-5-1-13(9-17(15)28)3-7-21(31)36-20-12-25(35,24(33)34)11-19(30)23(20)37-22(32)8-4-14-2-6-16(27)18(29)10-14/h1-10,19-20,23,26-30,35H,11-12H2,(H,33,34)/b7-3+,8-4+/t19-,20-,23+,25-/m1/s1	C1C(C(C(CC1(C(=O)O)O)OC(=O)C=CC2=CC(=C(C=C2)O)O)OC(=O)C=CC3=CC(=C(C=C3)O)O)O	6474309	89886-30-6	CHEMBL177126	Leaves	[69]	2/ Analyzed via RP-HPLC-PAD-MS[70], was identified by coelution with a pure reference standard. Retention time, UV spectra, and pseudomolecular/fragment ion masses matched the reference standard.<br><br>3/ Analysis was performed using LC-DAD-ESI-MS, identification based on Rt, UV-vis spectra, MS, and M.wt. compared with literature data.	Level 2 ★★★★☆<br><br>Level 3 ★★★☆☆	2/ [23]<br><br>3/ [71]
**11**	Chlorogenic acid	$C_{16}H_{18}O_{9}$	354.095085	InChI=1S/C16H18O9/c17-9-3-1-8(5-10(9)18)2-4-13(20)25-12-7-16(24,15(22)23)6-11(19)14(12)21/h1-5,11-12,14,17-19,21,24H,6-7H2,(H,22,23)/b4-2+/t11-,12-,14-,16+/m1/s1	C1C(C(C(CC1(C(=O)O)O)OC(=O)C=CC2=CC(=C(C=C2)O)O)O)O	1794427	202650-88-2	CHEMBL284616	Leaves, stems, branches, fruits, seeds, pomace, olive oil (EVOO), OOMW, and OLS	[2, 21, 35, 39, 64]	2/ Analyzed using RPLC-DAD-ESI-MS, confirmed with chemical standard, and supported by Rt, UV spectra, and MS data.	Level 2 ★★★★☆	2/ [32]
**12**	Decaffeoylverbascoside (verbasoside)	$C_{20}H_{30}O_{12}$	462.17373	InChI=1S/C20H30O12/c1-8-13(24)15(26)16(27)20(30-8)32-18-14(25)12(7-21)31-19(17(18)28)29-5-4-9-2-3-10(22)11(23)6-9/h2-3,6,8,12-28H,4-5,7H2,1H3/t8-,12+,13-,14+,15+,16+,17+,18-,19+,20-/m0/s1	CC1C(C(C(C(O1)OC2C(C(OC(C2O)OCCC3=CC(=C(C=C3)O)O)CO)O)O)O)O	11754080	61548-34-3	CHEMBL1779146	Leaves, wood, and OLS	[2, 69]	4/ Identification relied on mass spectrometry and MS/MS fragmentation using UPLC-Q-TOF/MS, and Literature comparison for spectral data.	Level 4 ★★☆☆☆	4/ [72]

No.	Compound name	Molecular formula	Exact mass	InChI	SMILES	CID	CAS Number	ChEMBL ID	Part used	References related to the part used	Identification Confidence*	Level 1 ★★★★★<br>Level 2 ★★★★☆<br>Level 3 ★★★☆☆<br>Level 4 ★★☆☆☆<br>Level 5 ★☆☆☆☆	References related to confidence level
**13**	Rosmarinic acid	$C_{18}H_{16}O_8$	360.08452	InChI=1S/C18H16O8/c19-12-4-1-10(7-14(12)21)3-6-17(23)26-16(18(24)25)9-11-2-5-13(20)15(22)8-11/h1-8,16,19-22H,9H2,(H,24,25)/b6-3+/t16-/m1/s1	C1=CC(=C(C=C1CC(C(=O)O)OC(=O)C=CC2=CC(=C(C=C2)O)O)O)O	5281792	20283-92-5	CHEMBL324842	Leaves, branches, fruits, and seeds	33, 47	4/ Identification via UPLC-ESI-Q-TOF based on RT and HRMS (m/z), but no MS/MS, UV, or NMR data provided to confirm structural elucidation.<br><br>4/ Analyzed using RP/HPLC-Q-TOF MS/MS analysis, and the identification based on precursor ion mass, Rt, and MS/MS fragmentation pattern matched against spectral libraries (NIST20, LipidBlast, MoNA).	Level 4 ★★☆☆☆	4/ [47]<br><br>4/ [64]
**14**	Calceolarioside	$C_{23}H_{26}O_{11}$	478.147515	InChI=1S/C23H26O11/c24-11-18-22(34-19(29)6-3-12-1-4-14(25)16(27)9-12)20(30)21(31)23(33-18)32-8-7-13-2-5-15(26)17(28)10-13/h1-6,9-10,18,20-28,30-31H,7-8,11H2/b6-3+/t18-,20-,21-,22-,23-/m1/s1	C1=CC(=C(C=C1CCOC2C(C(C(C(O2)CO)OC(=O)C=CC3=CC(=C(C=C3)O)O)O)O)O)O	5273566	84744-28-5	CHEMBL481635	Leaves, stems, OLS	2, 40	3/ Identification via HRMS/MS (Rt 11.0 min.) matched to an authentic standard.	Level 3 ★★★☆☆	3/ [40]
**15**	Verbascoside (acteoside)	$C_{29}H_{36}O_{15}$	624.205425	InChI=1S/C29H36O15/c1-13-22(36)23(37)24(38)29(41-13)44-27-25(39)28(40-9-8-15-3-6-17(32)19(34)11-15)42-20(12-30)26(27)43-21(35)7-4-14-2-5-16(31)18(33)10-14/h2-7,10-11,13,20,22-34,36-39H,8-9,12H2,1H3/b7-4+/t13-,20+,22-,23+,24+,25+,26+,27+,28+,29-/m0/s1	CC1C(C(C(C(O1)OC2C(C(OC(C2OC(=O)C=CC3=CC(=C(C=C3)O)O)CO)OCCC4=CC(=C(C=C4)O)O)O)O)O)O	5281800	61276-17-3	CHEMBL444478	Roots, flowers, fruits, leaves, stems, pomace, small branches, virgin olive oil, seeds, wood, bark, OOMW, and OLS	1, 2, 9, 25-27, 30, 40, 63, 73, 74	1/ 1D (1H/13C), 2D NMR (HMBC, HSQC, TOCSY), literature validation	Level 1 ★★★★★	1/ [75]
**16**	β-Hydroxyacteoside (β-hydroxyverbascoside)	$C_{29}H_{36}O_{16}$	640.20034	InChI=1S/C29H36O16/c1-12-22(37)23(38)24(39)29(42-12)45-27-25(40)28(41-11-19(35)14-4-6-16(32)18(34)9-14)43-20(10-30)26(27)44-21(36)7-3-13-2-5-15(31)17(33)8-13/h2-9,12,19-20,22-35,37-40H,10-11H2,1H3/b7-3+/t12-,19?,20+,22-,23+,24+,25+,26+,27+,28+,29-/m0/s1	CC1C(C(C(C(O1)OC2C(C(OC(C2OC(=O)C=CC3=CC(=C(C=C3)O)O)CO)OCC(C4=CC(=C(C=C4)O)O)O)O)O)O)O	10009317	95587-86-3	NF	Leaves, stems, roots, flowers, fruits, pomace, wood, and OLS	2, 9, 40, 76, 77	3/ Identification was based on chromatographic (Rt), spectroscopic (HRMS/MS/MS) data, and UV (via DAD) compared to literature.<br><br>3/ Identification relied on mass spectrometry and MS/MS fragments using RP-HPLC-DAD-ESI-QTOF-MS/MS and UV-Vis data, with literature comparison.	Level 3 ★★★☆☆	3/ [76]<br><br>3/ [2]

No.	Compound name	Molecular formula	Exact mass	InChI	SMILES	CID	CAS Number	ChEMBL ID	Part used	References related to the part used	Identification Confidence*	Level 1 ★★★★★ Level 2 ★★★★☆ Level 3 ★★★☆☆ Level 4 ★★☆☆☆ Level 5 ★☆☆☆☆	References related to confidence level
**17**	β-Methoxyverbascosi-de (β-methyl-OH verbascoside)	$C_{30}H_{38}O_{16}$	654.21599	InChI=1S/C30H38O16/c1-13-23(37)24(38)25(39)30(43-13)46-28-26(40)29(42-12-21(41-2)15-5-7-17(33)19(35)10-15)44-20(11-31)27(28)45-22(36)8-4-14-3-6-16(32)18(34)9-14/h3-10,13,20-21,23-35,37-40H,11-12H2,1-2H3/b8-4+	CC1C(O)C(O)C(O)C(OC2C(O)C(OCC(OC)C3=CC(O)=C(O)C=C3)OC(CO)C2OC(/C=C/C4=CC(O)=C(O)C=C4)=O)O1	NF	NF	NF	Leaves, stems, roots, flowers, and fruit	9, 40, 76	3/ Identification was based on chromatographic (Rt), spectroscopic (HRMS/MS/MS) data, and UV (via DAD) compared to literature.	Level 3 ★★★☆☆	3/ [76]
**18**	Plantamajoside	$C_{29}H_{36}O_{16}$	640.20034	InChI=1S/C29H36O16/c30-11-19-22(37)23(38)24(39)29(42-19)45-27-25(40)28(41-8-7-14-2-5-16(33)18(35)10-14)43-20(12-31)26(27)44-21(36)6-3-13-1-4-15(32)17(34)9-13/h1-6,9-10,19-20,22-35,37-40H,7-8,11-12H2/b6-3+/t19-,20-,22-,23+,24-,25-,26-,27-,28-,29+/m1/s1	C1=CC(=C(C=C1CCOC2C(C(C(C(O2)CO)OC(=O)C=CC3=CC(=C(C=C3)O)O)OC4C(C(C(C(O4)CO)O)O)O)O)O)O	5281788	104777-68-6	NF	Leaves	78	2/ Identification via HPLC-ESI-MS/MS matched (Rt) and MS/MS fragmentation data, and compared to the standard compound.	Level 2 ★★★★☆	2/ [79]
**19**	Hellicoside	$C_{29}H_{36}O_{17}$	656.195255	InChI=1S/C29H36O17/c30-9-19-22(38)23(39)24(40)29(43-19)46-27-25(41)28(42-11-18(36)13-3-5-15(33)17(35)8-13)44-20(10-31)26(27)45-21(37)7-2-12-1-4-14(32)16(34)7-12/h1-8,18-20,22-36,38-41H,9-11H2/b6-2+/t18-,19-,20-,22-,23+,24-,25-,26-,27-,28-,29+/m1/s1	C1=CC(=C(C=C1C=CC(=O)OC2C(OC(C(C2OC3C(C(C(C(O3)CO)O)O)O)O)OCC(C4=CC(=C(C=C4)O)O)O)CO)O)O	5281778	132278-04-7	NF	Olive oil (EVOO)	33	5/ It is not identified or analyzed, no data provided, included only as a reference structure, and shown only to illustrate structural similarities to DHPG precursors.	Level 5 ★☆☆☆☆	5/ [55]
**20**	Suspensaside	$C_{29}H_{36}O_{16}$	640.20034	InChI=1S/C29H36O16/c1-12-22(36)23(37)25(39)28(43-12)42-11-20-27(45-21(35)7-3-13-2-5-15(30)17(32)8-13)24(38)26(40)29(44-20)41-10-19(34)14-4-6-16(31)18(33)9-14/h2-9,12,19-20,22-34,36-40H,10-11H2,1H3/b7-3+/t12-,19?,20+,22-,23+,24+,25+,26+,27+,28+,29+/m0/s1	CC1C(C(C(C(O1)OCC2C(C(C(C(O2)OCC(C3=CC(=C(C=C3)O)O)O)O)O)OC(=O)C=CC4=CC(=C(C=C4)O)O)O)O)O	5281798	84213-44-5	NF	Leaves, fruits, and olive oil (EVOO)	33, 80	5/ It is not identified or analyzed, no data provided, included only as a reference structure, and shown only to illustrate structural similarities to DHPG precursors.	Level 5 ★☆☆☆☆	5/ [55]
**21**	Orobanchoside	$C_{29}H_{34}O_{15}$	622.189775	InChI=1S/C29H34O15/c1-12-22(36)23(37)24(38)28(40-12)44-26-25(43-21(35)7-3-13-2-5-15(31)17(33)8-13)19(10-30)42-29-27(26)41-20(11-39-29)14-4-6-16(32)18(34)9-14/h2-9,12,19-20,22-34,36-38H,10-11H2,1H3/b7-3+/t12-,19+,20+,22-,23+,24+,25+,26-,27+,28-,29+/m0/s1	CC1C(C(C(C(O1)OC2C3C(OCC(O3)C4=CC(=C(C=C4)O)O)OC(C2OC(=O)C=CC5=CC(=C(C=C5)O)O)CO)O)O)O	44593361	61276-16-2	CHEMBL451273	Olive oil (EVOO)	33	5/ It is not identified or analyzed, no data provided, included only as a reference structure, and shown only to illustrate structural similarities to DHPG precursors.	Level 5 ★☆☆☆☆	5/ [55]

No.	Compound name	Molecular formula	Exact mass	InChI	SMILES	CID	CAS Number	ChEMBL ID	Part used	References related to the part used	Identification Confidence*	Level 1 ★★★★★<br>Level 2 ★★★★☆<br>Level 3 ★★★☆☆<br>Level 4 ★★☆☆☆<br>Level 5 ★☆☆☆☆	References related to confidence level
**22**	Wedelosin	$C_{34}H_{60}O_{20}$	788.3678	InChI=1S/C34H60O20/c1-14-24(43)25(44)29(54-33-30(46)34(47,12-36)13-49-33)32(50-14)53-28-26(45)31(48-11-21(41)16-4-6-18(38)20(40)9-16)51-22(10-35)27(28)52-23(42)7-3-15-2-5-17(37)19(39)8-15/h14-33,35-47H,2-13H2,1H3	OC(CC1)C(CC1CCC(O)OC2C(C(C(OC2CO)OCC(C3CCC(C(C3)O)O)O)O)OC4C(C(C(C(C)O4)O)O)OC5C(C(CO5)(CO)O)O)O	NF	NF	NF	Olive oil (EVOO) and OOMW	33, 55	5/ It is not identified or analyzed, no data provided, included only as a reference structure, and shown only to illustrate structural similarities to DHPG precursors.	Level 5 ★☆☆☆☆	5/ [55]
**b. Benzoic acids and derivatives**													
**1**	Benzoic acid	$C_7H_6O_2$	122.03678	InChI=1S/C7H6O2/c8-7(9)6-4-2-1-3-5-6/h1-5H,(H,8,9)	C1=CC=C(C=C1)C(=O)O	243	65-85-0	CHEMBL541	Fruits, branches, leaves, wood, and olive oil (VOO)	12, 47, 56, 81	3/ Identification via GC (Rt = 26.67 min., MS and MS2 compared with the NIST database and literature references), and supported by FTIR/1D NMR (1H and 13C) data, but lacks direct structural proof.	Level 3 ★★★☆☆	3/ [48]
**2**	*ρ*-Hydroxybenzoic acid	$C_7H_6O_3$	138.031695	InChI=1S/C7H6O3/c8-6-3-1-5(2-4-6)7(9)10/h1-4,8H,(H,9,10)	C1=CC(=CC=C1C(=O)O)O	135	99-96-7	CHEMBL441343	Flowers, fruits, seeds, olive oil (VOO), leaves, stems, branches, wood, bark, and OOMW	2, 9, 34, 35, 47, 63, 82	2/ Identification by HPLC (Rt 19.9 min.) matched to an authentic standard; UV detection at 335 nm.	Level 2 ★★★★☆	2/ [61]
**3**	Salicylic acid	$C_7H_6O_3$	138.031695	InChI=1S/C7H6O3/c8-6-4-2-1-3-5(6)7(9)10/h1-4,8H,(H,9,10)	C1=CC=C(C(=C1)C(=O)O)O	338	69-72-7	CHEMBL424	Leaves, branches, fruits, and olive oil	47, 83	4/ Identification via UPLC-ESI-Q-TOF based on RT and HRMS (m/z), but no MS/MS, UV, or NMR data provided to confirm structural elucidation.	Level 4 ★★☆☆☆	4/ [47]
**4**	3,4-Dihydroxybenzoic acid hexoside pentoside	$C_{18}H_{24}O_{13}$	448.121695	InChI=1S/C18H24O13/c19-4-9-11(21)13(23)15(25)18(30-9)29-8-3-6(16(26)27)1-2-7(8)28-17-14(24)12(22)10(5-20)31-17/h1-3,9-15,17-25H,4-5H2,(H,26,27)	O=C(O)C1=CC=C(OC2C(C(C(CO)O2)O)O)C(OC3C(C(C(C(CO)O3)O)O)O)=C1	NF	NF	NF	Roots, olive oil (VOO), wood, and OLS	1, 2, 63	3/ Identification via HPLC-DAD and LC-ESI-QTOF-MS/MS, relies on chromatographic alignment (Rt) and spectrometric data (HRMS/MSMS) compared to literature data.	Level 3 ★★★☆☆	3/ [1]
**5**	Gallic acid	$C_7H_6O_5$	170.021525	InChI=1S/C7H6O5/c8-4-1-3(7(11)12)2-5(9)6(4)10/h1-2,8-10H,(H,11,12)	C1=C(C=C(C(=C1O)O)O)C(=O)O	370	149-91-7	CHEMBL288114	Pomace, fruits, seeds, olive oil (VOO& EVOO),	21, 25, 33, 35, 58, 63, 84	2/ Analyzed using RPLC-DAD-ESI-MS, confirmed with chemical standard, and supported by Rt, UV spectra, and MS data.	Level 2 ★★★★☆	2/ [32]

No.	Compound name	Molecular formula	Exact mass	InChI	SMILES	CID	CAS Number	ChEMBL ID	Part used	References related to the part used	Identification Confidence*	Level 1 ★★★★★<br>Level 2 ★★★★☆<br>Level 3 ★★★☆☆<br>Level 4 ★★☆☆☆<br>Level 5 ★☆☆☆☆	References related to confidence level
									leaves, stems, fruits, OOMW, and OLS				
**6**	Protocatechuic acid (dihydroxybenzoic acid/ DHBA)	$C_7H_6O_4$	154.02661	InChI=1S/C7H6O4/c8-4-1-2-6(9)5(3-4)7(10)11/h1-3,8-9H,(H,10,11)	C1=CC(=C(C=C1O)C(=O)O)O	72	99-50-3	CHEMBL37537	Pomace, leaves, stems, branches, fruits, seeds, OOMW, and olive oil (EVOO)	21, 25, 33, 35, 47	2/ Analyzed via RP-HPLC-PAD-MS[70] was identified by coelution with a pure reference standard, matching Rt, UV spectra, and mass spectrometry data (pseudomolecular/ fragment ions).<br><br>3/ Identification via 1D (1H and 13C) and 2D NMR (gCOSY, gNOESY, gHMQC, and gHMBC) compared to literature.	Level 2 ★★★★☆<br><br>Level 3 ★★★☆☆	2/ [23]<br>3/ [68]
**7**	Protocatechuic acid 4-*O*-glucoside (dihydroxybenzoic acid glucoside/ DHBA glucoside)	$C_{13}H_{16}O_9$	316.079435	InChI=1S/C13H16O9/c14-4-8-9(16)10(17)11(18)13(22-8)21-7-2-1-5(12(19)20)3-6(7)15/h1-3,8-11,13-18H,4H2,(H,19,20)	C1=CC(=C(C=C1C(=O)O)O)OC2C(C(C(C(O2)CO)O)O)O	157010113	99-50-3	NF	Leaves and stems	2	3/ Identification relied on mass spectrometry and MS/MS fragments using RP-HPLC-DAD-ESI-QTOF-MS/MS and UV-Vis data, with literature comparison.	Level 3 ★★★☆☆	3/ [2]
**8**	Vanillic acid	$C_8H_8O_4$	168.04226	InChI=1S/C8H8O4/c1-12-7-4-5(8(10)11)2-3-6(7)9/h2-4,9H,1H3,(H,10,11)	COC1=C(C=CC(=C1)C(=O)O)O	8468	121-34-6	CHEMBL120568	Pomace, fruits, seeds, olive oil (VOO& EVOO), leaves, stems, branches, bark, and OOMW	8, 12, 21, 25, 35, 47, 58, 63	2/ Analysis by LC-SPE-NMR with Rt matching (10.5 min), structural elucidation via 1D (1H and 13C) and 2D NMR (TOCSY) compared with authentic standard.	Level 2 ★★★★☆	2/ [28]
**9**	Syringic acid (4-hydroxy-3,5-dimethoxybenzoic acid)	$C_9H_{10}O_5$	198.052824	InChI=1S/C9H10O5/c1-13-6-3-5(9(11)12)4-7(14-2)8(6)10/h3-4,10H,1-2H3,(H,11,12)	COC1=CC(=CC(=C1O)OC)C(=O)O	10742	530-57-4	CHEMBL1414	Olive oil, fruits, pomace, and OOMW	58, 60	2/ Analyzed using RPLC-DAD-ESI-MS, confirmed with chemical standard, and supported by Rt, UV spectra, and MS data.	Level 2 ★★★★☆	2/ [32]
**10**	Eudesmic acid (3,4,5-trimethoxybenzoic acid)	$C_{10}H_{12}O_5$	212.068475	InChI=1S/C10H12O5/c1-13-7-4-6(10(11)12)5-8(14-2)9(7)15-3/h4-5H,1-3H3,(H,11,12)	COC1=CC(=CC(=C1OC)OC)C(=O)O	8357	118-41-2	CHEMBL377172	Olive oil (VOO) and leaves	85	4/ Identification based only on literature comparison using LC-QTOF-MS (high-resolution MS) with ESI and APCI sources.	Level 4 ★★☆☆☆	4/ [85]
**11**	Ellagic acid (heterotetracyclic organic compound)	$C_{14}H_6O_8$	302.00627	InChI=1S/C14H6O8/c15-5-1-3-7-8-4(14(20)22-11(7)9(5)17)2-6(16)10(18)12(8)21-13(3)19/h1-2,15-18H	C1=C2C3=C(C(=C1O)O)OC(=O)C4=CC(=C(C(=C43)OC2=O)O)O	5281855	476-66-4	CHEMBL6246	Leaves and fruits	73, 86	3/ Identification based on chromatography RPLC-DAD-ESI-MS and UV data compared to literature, with incomplete validation.	Level 3 ★★★☆☆	3/ [32]

No.	Compound name	Molecular formula	Exact mass	InChI	SMILES	CID	CAS Number	ChEMBL ID	Part used	References related to the part used	Identification Confidence*	Level 1 ★★★★★<br>Level 2 ★★★★☆<br>Level 3 ★★★☆☆<br>Level 4 ★★☆☆☆<br>Level 5 ★☆☆☆☆	References related to confidence level
**12**	Valoneic acid dilactone	$C_{21}H_{10}O_{13}$	470.012145	InChI=1S/C21H10O13/c22-7-2-6(19(28)29)16(15(27)12(7)24)32-9-3-5-11-10-4(20(30)34-18(11)14(9)26)1-8(23)13(25)17(10)33-21(5)31/h1-3,22-27H,(H,28,29)	C1=C2C3=C(C(=C1O)O)OC(=O)C4=CC(=C(C(=C43)OC2=O)O)OC5=C(C(=C(C=C5C(=O)O)O)O)O	10151874	60202-70-2	CHEMBL5079864	Leaves	[69]	3/ Analysis was performed using LC-DAD-ESI-MS, identification based on Rt, UV-vis spectra, MS, and M.wt. compared with literature data.	Level 3 ★★★☆☆	3/ [71]
**13**	Gallagic acid	$C_{28}H_{14}O_{18}$	638.01802	InChI=1S/C28H14O18/c29-5-1-3(25(39)40)7(17(33)15(5)31)9-13-11-12-14(28(44)46-23(11)21(37)19(9)35)10(20(36)22(38)24(12)45-27(13)43)8-4(26(41)42)2-6(30)16(32)18(8)34/h1-2,29-38H,(H,39,40)(H,41,42)	C1=C(C(=C(C(=C1O)O)O)C2=C(C(=C3C4=C2C(=O)OC5=C(C(=C(C(=C45)C(=O)O3)C6=C(C(=C(C=C6C(=O)O)O)O)O)O)O)O)O)C(=O)O	14754405	65995-62-2	NF	Leaves	[69]	3/ Analysis was performed using LC-DAD-ESI-MS, identification based on Rt, UV-Vis spectra, MS, and M.wt. compared with literature data.	Level 3 ★★★☆☆	3/ [71]
**14**	Diellagilactone	$C_{28}H_{10}O_{16}$	601.99689	InChI=1S/C28H10O16/c29-5-1-3-7-11-13(27(39)43-21(7)15(5)31)9(17(33)19(35)23(11)41-25(3)37)10-14-12-8-4(26(38)42-24(12)20(36)18(10)34)2-6(30)16(32)22(8)44-28(14)40/h1-2,29-36H	C1=C2C3=C(C(=C1O)O)OC(=O)C4=C3C(=C(C(=C4C5=C(C(=C6C7=C5C(=O)OC8=C7C(=CC(=C8O)O)C(=O)O6)O)O)O)O)OC2=O	131752354	153816-55-8	NF	Leaves	[69]	3/ Analysis was performed using LC-DAD-ESI-MS, identification based on Rt, UV-Vis spectra, MS, and M.wt. compared with literature data.	Level 3 ★★★☆☆	3/ [71]
**c. Phenylacetic acids**													
**1**	4-Hydroxyphenyl acetic acid (4-HPA)	$C_8H_8O_3$	152.047345	InChI=1S/C8H8O3/c9-7-3-1-6(2-4-7)5-8(10)11/h1-4,9H,5H2,(H,10,11)	C1=CC(=CC=C1CC(=O)O)O	11970	614-75-5	CHEMBL240714	Leaves, stems, fruits, pomace, bark, olive oil (EVOO), and OOMW	[12, 21, 30, 35, 58, 87, 88]	2/ Analyzed using RPLC-DAD-ESI-MS, confirmed with chemical standard, and supported by Rt, UV spectra, and MS data.	Level 2 ★★★★☆	2/ [32]
**2**	Homovanillic acid	$C_9H_{10}O_4$	182.05791	InChI=1S/C9H10O4/c1-13-8-4-6(5-9(11)12)2-3-7(8)10/h2-4,10H,5H2,1H3,(H,11,12)	COC1=C(C=CC(=C1)CC(=O)O)O	1738	306-08-1	CHEMBL1562	fruits, pomace, olive oil, and OOMW	[58, 88]	2/ Analyzed using RPLC-DAD-ESI-MS, confirmed with chemical standard, and supported by Rt, UV spectra, and MS data.<br><br>3/ Identification via 1H and 31P NMR combined with chemometrics that relied on matching spectroscopic data to literature values.	Level 2 ★★★★☆<br><br>Level 3 ★★★☆☆	2/ [32]<br><br>3/ [89]
**d. Phenylpropanoic acids**													

No.	Compound name	Molecular formula	Exact mass	InChI	SMILES	CID	CAS Number	ChEMBL ID	Part used	References related to the part used	Identification Confidence*	Level 1 ★★★★★<br>Level 2 ★★★★☆<br>Level 3 ★★★☆☆<br>Level 4 ★★☆☆☆<br>Level 5 ★☆☆☆☆	References related to confidence level
**1**	Phloretic acid	$C_9H_{10}O_3$	166.062995	InChI=1S/C9H10O3/c10-8-4-1-7(2-5-8)3-6-9(11)12/h1-2,4-5,10H,3,6H2,(H,11,12)	C1=CC(=CC=C1CCC(=O)O)O	10394	501-97-3	CHEMBL1172560	Fruits	[74]	3/ Analyzed using GC/MS analysis in Single Ion Monitoring (SIM) mode after derivatization with BSTFA/TMCS, and the identification based on Rt, fragment ions, and external standard.	Level 3 ★★★☆☆	3/ [90]
**2**	3-Hydroxyphloretin 2'-*O*-xylosyl glucoside	$C_{26}H_{32}O_{15}$	584.174125	InChI=1S/C26H32O15/c27-11-6-15(31)19(13(29)4-2-10-1-3-12(28)14(30)5-10)17(7-11)40-26-24(37)22(35)21(34)18(41-26)9-39-25-23(36)20(33)16(32)8-38-25/h1,3,5-7,16,18,20-28,30-37H,2,4,8-9H2/t16-,18-,20+,21-,22+,23-,24-,25+,26-/m1/s1	C1C(C(C(C(O1)OCC2C(C(C(C(O2)OC3=CC(=CC(=C3C(=O)CCC4=CC(=C(C=C4)O)O)O)O)O)O)O)O)O)O	121225502	NF	NF	Leaves	[69]	3/ Analysis was performed using LC-DAD-ESI-MS, identification based on Rt, UV-Vis spectra, MS, and M.wt. compared with literature data.	Level 3 ★★★☆☆	3/ [71]
	**4- Coumarins and hydroxycoumarin derivatives**												
**1**	Esculetin (aesculetin/ dihydroxycoumarin)	$C_9H_6O_4$	178.02661	InChI=1S/C9H6O4/c10-6-3-5-1-2-9(12)13-8(5)4-7(6)11/h1-4,10-11H	C1=CC(=O)OC2=CC(=C(C=C21)O)O	5281416	305-01-1	CHEMBL244743	Leaves, stems, branches, wood, bark, flowers, fruits, and roots	[9, 12, 33, 40, 47, 86]	2/ Analyzed using HPLC-ESI-TOF/MS and HPLC-ESI/MS/MS analysis, and the identification based on direct comparison of Rt and MS/MS fragmentation to an authentic esculetin standard. Quantitative validation via external calibration curve.<br><br>3/ Identification relied on mass spectrometry and MS/MS fragments using RP-HPLC-DAD-ESI-QTOF-MS/MS and UV-vis data, with literature comparison.	Level 2 ★★★★☆<br><br>Level 3 ★★★☆☆	2/ [34]<br><br>3/ [2]
**2**	Esculin (aesculin or esculetin glucoside)	$C_{15}H_{16}O_9$	340.079435	InChI=1S/C15H16O9/c16-5-10-12(19)13(20)14(21)15(24-10)23-9-3-6-1-2-11(18)22-8(6)4-7(9)17/h1-4,10,12-17,19-21H,5H2/t10-,12-,13+,14-,15-/m1/s1	C1=CC(=O)OC2=CC(=C(C=C21)OC3C(C(C(C(O3)CO)O)O)O)O	5281417	531-75-9	CHEMBL482581	Leaves, stems, roots, wood, bark, and OLS	[2, 33, 40, 79]	2/ Identification via HPLC-ESI-MS/MS matched (Rt) and MS/MS fragmentation data, and compared to the standard compound.<br><br>3/ Identification relied on mass spectrometry and MS/MS fragments using RP-HPLC-DAD-ESI-QTOF-MS/MS and UV/HRMS/MSMS, with literature validation for Rt and spectral data.	Level 2 ★★★★☆	2/ [79]<br><br>3/ [2]

No.	Compound name	Molecular formula	Exact mass	InChI	SMILES	CID	CAS Number	ChEMBL ID	Part used	References related to the part used	Identification Confidence*	Level 1 ★★★★★<br>Level 2 ★★★★☆<br>Level 3 ★★★☆☆<br>Level 4 ★★☆☆☆<br>Level 5 ★☆☆☆☆	References related to confidence level
**3**	Cichoriin (isomer of esculin)	$C_{15}H_{16}O_9$	340.079435	InChI=1S/C15H16O9/c16-5-10-12(19)13(20)14(21)15(24-10)23-9-4-8-6(3-7(9)17)1-2-11(18)22-8/h1-4,10,12-17,19-21H,5H2/t10-,12-,13+,14-,15-/m1/s1	C1=CC(=O)OC2=CC(=C(C=C21)O)OC3C(C(C(C(O3)CO)O)O)O	442101	531-58-8	NF	Bark	[12]	3/ Analyzed using HPLC-ESI-TOF/MS and HPLC-ESI/MS/MS analysis, and the identification based on identical molecular formula and fragmentation to esculin (authentic standard).	Level 3 ★★★☆☆	3/ [34]
**4**	Scopoletin (6-methylesculetin)	$C_{10}H_8O_4$	192.04226	InChI=1S/C10H8O4/c1-13-9-4-6-2-3-10(12)14-8(6)5-7(9)11/h2-5,11H,1H3	COC1=C(C=C2C(=C1)C=CC(=O)O2)O	5280460	92-61-5	CHEMBL71851	Leaves, branches, fruits, seeds, wood, and bark	[2, 33, 47]	3/ Analyzed using HPLC-ESI-TOF/MS and HPLC-ESI/MS/MS analysis, and the identification was based on comparing its fragmentation and methyl-group difference to esculetin.<br><br>3/ Identification relied on mass spectrometry and MS/MS fragments using RP-HPLC-DAD-ESI-QTOF-MS/MS and UV/HRMS/MSMS, with literature validation for Rt and spectral data.	Level 3 ★★★☆☆	3/ [34]<br><br>3/ [2]
**5**	Scopolin (scopoletin 7-*O*-glucoside)	$C_{16}H_{18}O_9$	354.095085	InChI=1S/C16H18O9/c1-22-9-4-7-2-3-12(18)23-8(7)5-10(9)24-16-15(21)14(20)13(19)11(6-17)25-16/h2-5,11,13-17,19-21H,6H2,1H3/t11-,13-,14+,15-,16-/m1/s1	COC1=C(C=C2C(=C1)C=CC(=O)O2)OC3C(C(C(C(O3)CO)O)O)O	439514	531-44-2	CHEMBL225024	Bark and leaves	[33, 91]	2/ Analyzed using HPLC-UV, and co-chromatography with an authentic scopolin standard.	Level 2 ★★★★☆	2/ [92]
**6**	Dimeresculetin	$C_{18}H_{10}O_8$	354.03757	InChI=1S/C18H10O8/c19-10-6-12-8(1-3-15(21)24-12)5-14(10)26-18-9-2-4-16(22)25-13(9)7-11(20)17(18)23/h1-7,19-20,23H	OC(C=C1O2)=C(OC3=C(O)C(O)=CC(O4)=C3C=CC4=O)C=C1C=CC2=O	NF	NF	NF	Bark	[34]	3/ Analyzed using HPLC-ESI-TOF/MS and HPLC-ESI/MS/MS analysis, and spectral data were compared to esculetin (authentic standard).	Level 3 ★★★☆☆	3/ [34]
**5- Flavonoids**													
**a. Flavanols**													
**1**	(+)-Catechin	$C_{15}H_{14}O_6$	290.07904	InChI=1S/C15H14O6/c16-8-4-11(18)9-6-13(20)15(21-14(9)5-8)7-1-2-10(17)12(19)3-7/h1-5,13,15-20H,6H2/t13-,15+/m0/s1	C1C(C(OC2=CC(=CC(=C21)O)O)C3=CC(=C(C=C3)O)O)O	9064	154-23-4	CHEMBL311498	Leaves, fruits, wood, and olive oil (EVOO)	[12, 21, 93, 94]	4/ Analyzed using HPLC-ECD/UV analysis, and the identification relies only on Rt compared to catechin commercially purchased chemical standard, and UV spectra.	Level 4 ★★☆☆☆	4/ [21]
**2**	Gallocatechin	$C_{15}H_{14}O_7$	306.073955	InChI=1S/C15H14O7/c16-7-3-9(17)8-5-12(20)15(22-13(8)4-7)6-1-10(18)14(21)11(19)2-6/h1-4,12,15-21H,5H2/t12-,15+/m0/s1	C1C(C(OC2=CC(=CC(=C21)O)O)C3=CC(=C(C(=C3)O)O)O)O	65084	970-73-0	CHEMBL125743	Seeds, flowers, and leaves	[9, 38, 73]	3/ Identification relies on chromatographic behavior (Rt), UV spectrum, and MS/MS fragmentation pattern using LC-ESI-QTOF-MS, with Literature comparison.	Level 3 ★★★☆☆	3/ [73]

No.	Compound name	Molecular formula	Exact mass	InChI	SMILES	CID	CAS Number	ChEMBL ID	Part used	References related to the part used	Identification Confidence*	Level 1 ★★★★★<br>Level 2 ★★★★☆<br>Level 3 ★★★☆☆<br>Level 4 ★★☆☆☆<br>Level 5 ★☆☆☆☆	References related to confidence level
**3**	Epicatechin 3-*O*-ρ-hydroxybenzoate	$C_{22}H_{18}O_8$	410.10017	InChI=1S/C22H18O8/c23-13-4-1-11(2-5-13)22(28)30-20-10-15-17(26)8-14(24)9-19(15)29-21(20)12-3-6-16(25)18(27)7-12/h1-9,20-21,23-27H,10H2/t20-,21-/m1/s1	C1C(C(OC2=CC(=CC(=C21)O)O)C3=CC(=C(C=C3)O)O)OC(=O)C4=CC=C(C=C4)O	10764037	108907-45-5	NF	Leaves	69	3/ Analysis was performed using LC-DAD-ESI-MS, identification based on Rt, UV-vis spectra, MS, and M.wt. compared with literature data.	Level 3 ★★★☆☆	3/ 71
**b. Flavanones**													
**1**	Naringenin	$C_{15}H_{12}O_5$	272.068475	InChI=1S/C15H12O5/c16-9-3-1-8(2-4-9)13-7-12(19)15-11(18)5-10(17)6-14(15)20-13/h1-6,13,16-18H,7H2/t13-/m0/s1	C1C(OC2=CC(=CC(=C2C1=O)O)O)C3=CC=C(C=C3)O	439246	480-41-1	CHEMBL9352	Leaves, branches, fruit, seeds, olive pomace, bark, wood, and olive oil (EVOO)	34, 47, 83, 85	2/ 3/ Analyzed using HPLC-ESI/MS/MS in MRM mode and the identification based on (Rt) and spectral data compared to an authentic standard.<br>3/ Identification via LC-ESI-MS and LC-APCI-MS compared to spectral library.	Level 2 ★★★★☆<br>Level 3 ★★★☆☆	2/ 34<br>3/ 85
**2**	Naringenin 7-*O*-glucoside	$C_{21}H_{22}O_{10}$	434.1213	InChI=1S/C21H22O10/c22-8-16-18(26)19(27)20(28)21(31-16)29-11-5-12(24)17-13(25)7-14(30-15(17)6-11)9-1-3-10(23)4-2-9/h1-6,14,16,18-24,26-28H,7-8H2/t14-,16+,18+,19-,20+,21+/m0/s1	C1C(OC2=CC(=CC(=C2C1=O)O)OC3C(C(C(C(O3)CO)O)O)O)C4=CC=C(C=C4)O	92794	529-55-5	CHEMBL469654	Bark	34	3/ 3/ Analyzed using HPLC-ESI-TOF/MS and HPLC-ESI/MS/MS analysis, and product ions matching naringenin aglycone.	Level 3 ★★★☆☆	3/ 34
**3**	Eriodictyol (eriodyctiol)	$C_{15}H_{12}O_6$	288.06339	InChI=1S/C15H12O6/c16-8-4-11(19)15-12(20)6-13(21-14(15)5-8)7-1-2-9(17)10(18)3-7/h1-5,13,16-19H,6H2/t13-/m0/s1	C1C(OC2=CC(=CC(=C2C1=O)O)O)C3=CC(=C(C=C3)O)O	440735	552-58-9	CHEMBL8996	Bark, wood, branches, leaves, fruits, and seeds	24, 34, 47, 77	2/ Identification via HPLC-ESI-MS/MS matched (Rt) and MS/MS fragmentation data, and compared to the standard compound.	Level 2 ★★★★☆	2/ 79
**4**	Eriodictyol 7-*O*-glucoside	$C_{21}H_{22}O_{11}$	450.116215	InChI=1S/C21H22O11/c22-7-16-18(27)19(28)20(29)21(32-16)30-9-4-12(25)17-13(26)6-14(31-15(17)5-9)8-1-2-10(23)11(24)3-8/h1-5,14,16,18-25,27-29H,6-7H2/t14-,16+,18+,19-,20+,21+/m0/s1	C1C(OC2=CC(=CC(=C2C1=O)O)OC3C(C(C(C(O3)CO)O)O)O)C4=CC(=C(C=C4)O)O	13254473	38965-51-4	CHEMBL509624	Leaves and branches	64, 77	2/ Analyzed via RP-HPLC-PAD-MS[70], was identified by coelution with a pure reference standard. Retention time, UV spectra, and pseudomolecular/fragment ion masses matched the reference standard.	Level 2 ★★★★☆	2/ 23
**5**	Eriodictyol 7-*O*-rutinoside (eriocitrin)	$C_{27}H_{32}O_{15}$	596.174125	InChI=1S/C27H32O15/c1-9-20(32)22(34)24(36)26(39-9)38-8-18-21(33)23(35)25(37)27(42-18)40-11-5-14(30)19-15(31)7-16(41-17(19)6-11)10-2-3-12(28)13(29)4-10/h2-6,9,16,18,20-30,32-37H,7-8H2,1H3/t9-,16-,18+,20-,21+,22+,23-,24+,25+,26+,27+/m0/s1	CC1C(C(C(C(O1)OCC2C(C(C(C(O2)OC3=CC(=C4C(=O)CC(OC4=C3)C5=CC(=C(C=C5)O)O)O)O)O)O)O)O)O	83489	13463-28-0	CHEMBL2165586	Leaves	23	2/ Analyzed via RP-HPLC-PAD-MS[70], was identified by coelution with a pure reference standard. Rt, UV spectra, and pseudomolecular/fragment ion masses were matched to the reference standard.	Level 2 ★★★★☆	2/ 23

No.	Compound name	Molecular formula	Exact mass	InChI	SMILES	CID	CAS Number	ChEMBL ID	Part used	References related to the part used	Identification Confidence*	Level 1 ★★★★★<br>Level 2 ★★★★☆<br>Level 3 ★★★☆☆<br>Level 4 ★★☆☆☆<br>Level 5 ★☆☆☆☆	References related to confidence level
**6**	Hesperitin	$C_{16}H_{14}O_6$	302.07904	InChI=1S/C16H14O6/c1-21-13-3-2-8(4-10(13)18)14-7-12(20)16-11(19)5-9(17)6-15(16)22-14/h2-6,14,17-19H,7H2,1H3/t14-/m0/s1	COC1=C(C=C(C=C1)C2CC(=O)C3=C(C=C(C=C3O2)O)O)O	72281	520-33-2	CHEMBL399121	Leaves and olive oil (EVOO)	[81, 95]	5/ No experimental details or analytical proof provided.	Level 5 ★☆☆☆☆	5/ [32]
**7**	Hesperidin (hesperetin-7-*O*-rutinoside)	$C_{28}H_{34}O_{15}$	610.189775	InChI=1S/C28H34O15/c1-10-21(32)23(34)25(36)27(40-10)39-9-19-22(33)24(35)26(37)28(43-19)41-12-6-14(30)20-15(31)8-17(42-18(20)7-12)11-3-4-16(38-2)13(29)5-11/h3-7,10,17,19,21-30,32-37H,8-9H2,1-2H3/t10-,17-,19+,21-,22+,23+,24-,25+,26+,27+,28+/m0/s1	CC1C(C(C(C(O1)OCC2C(C(C(C(O2)OC3=CC(=C4C(=O)CC(OC4=C3)C5=CC(=C(C=C5)OC)O)O)O)O)O)O)O)O	10621	520-26-3	CHEMBL449317	Fruits, pomace, and leaves	[58, 88]	3/ Identification based on chromatography RPLC-DAD-ESI-MS and UV data compared to literature, but lacks confirmation with a reference standard or full structural validation.	Level 3 ★★★☆☆	3/ [32]
**c. Flavanonols**													
**1**	Dihydrokaempferol	$C_{15}H_{12}O_6$	288.06339	InChI=1S/C15H12O6/c16-8-3-1-7(2-4-8)15-14(20)13(19)12-10(18)5-9(17)6-11(12)21-15/h1-6,14-18,20H/t14-,15+/m0/s1	C1=CC(=CC=C1C2C(C(=O)C3=C(C=C(C=C3O2)O)O)O)O	122850	480-20-6	CHEMBL9323	Fruits, branches, and stems	[47, 96]	4/ Identification via UPLC-ESI-Q-TOF based on RT and HRMS (m/z), but no MS/MS, UV, or NMR data provided to confirm structural elucidation.	Level 4 ★★☆☆☆	4/ [47]
**2**	Fustin	$C_{15}H_{12}O_6$	288.06339	InChI=1S/C15H12O6/c16-8-2-3-9-12(6-8)21-15(14(20)13(9)19)7-1-4-10(17)11(18)5-7/h1-6,14-18,20H/t14-,15+/m0/s1	C1=CC(=C(C=C1C2C(C(=O)C3=C(O2)C=C(C=C3)O)O)O)O	5317435	20725-03-5	CHEMBL470267	Bark	[34]	3/ Analyzed using HPLC-ESI-TOF/MS and HPLC-ESI/MS/MS analysis, and the identification was based solely on literature data for fragmentation patterns.	Level 3 ★★★☆☆	3/ [34]
**3**	Taxifolin	$C_{15}H_{12}O_7$	304.058305	InChI=1S/C15H12O7/c16-7-4-10(19)12-11(5-7)22-15(14(21)13(12)20)6-1-2-8(17)9(18)3-6/h1-5,14-19,21H/t14-,15+/m0/s1	C1=CC(=C(C=C1C2C(C(=O)C3=C(C=C(C=C3O2)O)O)O)O)O	439533	480-18-2	CHEMBL66	Leaves, stems, flowers, fruits, seeds, pomace, small branches, olive oil (VOO), bark, and OLS	[2, 9, 25, 27, 33, 34, 47, 63, 79]	3/ Analyzed using HPLC-ESI-TOF/MS and HPLC-ESI/MS/MS analysis, and the identification was based solely on literature data for fragmentation patterns.<br><br>3/ Identification relied on mass spectrometry and MS/MS fragments using RP-HPLC-DAD-ESI-QTOF-MS/MS and UV/HRMS/MSMS, with literature validation for Rt and spectral data.	Level 3 ★★★☆☆	3/ [34]<br><br>3/ [2]
**4**	Taxifolin-3-*O*-glucoside	$C_{21}H_{22}O_{12}$	466.11113	InChI=1S/C21H22O12/c22-6-13-15(27)17(29)18(30)21(32-13)33-20-16(28)14-11(26)4-8(23)5-12(14)31-19(20)7-1-2-9(24)10(25)3-7/h1-5,13,15,17-27,29-30H,6H2/t13-,15-,17+,18-,19-,20+,21+/m1/s1	C1=CC(=C(C=C1C2C(C(=O)C3=C(C=C(C=C3O2)O)O)OC4C(C(C(C(O4)CO)O)O)O)O)O	14187089	27297-45-6	CHEMBL2332678	Leaves and branches	[77, 79]	3/ The identification based on Chromatography (Rt) + UV/HRMS/MSMS using HPLC-ESI-MS/MS compared to literature data.	Level 3 ★★★☆☆	3/ [79]
**d. Flavones**													

No.	Compound name	Molecular formula	Exact mass	InChI	SMILES	CID	CAS Number	ChEMBL ID	Part used	References related to the part used	Identification Confidence*	Level 1 ★★★★★<br>Level 2 ★★★★☆<br>Level 3 ★★★☆☆<br>Level 4 ★★☆☆☆<br>Level 5 ★☆☆☆☆	References related to confidence level
**1**	Luteolin	$C_{15}H_{10}O_6$	286.04774	InChI=1S/C15H10O6/c16-8-4-11(19)15-12(20)6-13(21-14(15)5-8)7-1-2-9(17)10(18)3-7/h1-6,16-19H	C1=CC(=C(C=C1C2=CC(=O)C3=C(C=C(C=C3O2)O)O)O)O	5280445	491-70-3	CHEMBL151	Roots, fruits, seeds, flowers, pomace, leaves, stems, branches, olive oil (VOO& EVOO), bark, and OOMW	1, 9, 25, 34, 38, 40, 47, 63, 73, 83	2/ Analysis by LC-SPE-NMR with Rt matching (18.2 min), structural elucidation via 1D (1H and 13C) and 2D NMR (TOCSY) compared with authentic standard.	Level 2 ★★★★☆	2/ [28]
**2**	Hispidulin	$C_{16}H_{12}O_6$	300.06339	InChI=1S/C16H12O6/c1-21-16-11(19)7-13-14(15(16)20)10(18)6-12(22-13)8-2-4-9(17)5-3-8/h2-7,17,19-20H,1H3	COC1=C(C2=C(C=C1O)OC(=CC2=O)C3=CC=C(C=C3)O)O	5281628	1447-88-7	CHEMBL293776	Leaves, branches, fruits, seeds, and olive oil	47	3/ The identification based on Chromatography (Rt) + UV/HRMS/MSMS using HPLC-ESI-MS/MS compared to literature data.	Level 3 ★★★☆☆	3/ [79]
**3**	Chrysoeriol	$C_{16}H_{12}O_6$	300.06339	InChI=1S/C16H12O6/c1-21-14-4-8(2-3-10(14)18)13-7-12(20)16-11(19)5-9(17)6-15(16)22-13/h2-7,17-19H,1H3	COC1=C(C=CC(=C1)C2=CC(=O)C3=C(C=C(C=C3O2)O)O)O	5280666	491-71-4	CHEMBL214321	Leaves	97	3/ Isolation by TLC and column chromatography, confirmed its structure by matching NMR (1H, 13C) chemical shifts and splitting patterns with a reference study.	Level 3 ★★★☆☆	3/ [97]
**4**	Chrysoeriol-7-*O*-glucoside	$C_{22}H_{22}O_{11}$	462.116215	InChI=1S/C22H22O11/c1-30-15-4-9(2-3-11(15)24)14-7-13(26)18-12(25)5-10(6-16(18)32-14)31-22-21(29)20(28)19(27)17(8-23)33-22/h2-7,17,19-25,27-29H,8H2,1H3/t17-,19-,20+,21-,22-/m1/s1	COC1=C(C=CC(=C1)C2=CC(=O)C3=C(C=C(C=C3O2)OC4C(C(C(C(O4)CO)O)O)O)O)O	11294177	19993-32-9	CHEMBL486391	Leaves, stems, fruits, seeds, and OLS	2, 33, 87	3/ Identification relied on mass spectrometry and MS/MS fragments using RP-HPLC-DAD-ESI-QTOF-MS/MS and UV-vis data, with literature comparison.	Level 3 ★★★☆☆	3/ [2]
**5**	Luteolin-7-*O*-glucoside	$C_{21}H_{20}O_{11}$	448.100564	InChI=1S/C21H20O11/c22-7-16-18(27)19(28)20(29)21(32-16)30-9-4-12(25)17-13(26)6-14(31-15(17)5-9)8-1-2-10(23)11(24)3-8/h1-6,16,18-25,27-29H,7H2/t16-,18-,19+,20-,21-/m1/s1	C1=CC(=C(C=C1C2=CC(=O)C3=C(C=C(C=C3O2)OC4C(C(C(C(O4)CO)O)O)O)O)O)O	5280637	5373-11 -5	CHEMBL233929	Fruits, fruit peel, leaves, stems, branches, seeds, olive oil, flowers, pomace, wood, bark, OOMW, and OLS	2, 9, 25, 33, 34, 47, 74, 77, 98	3/ The identification relies on chromatographic (RT) and spectrometric (HRMS/MSMS and NMR) parameters, cross-referenced with literature data.	Level 3 ★★★☆☆	3/ [99]

No.	Compound name	Molecular formula	Exact mass	InChI	SMILES	CID	CAS Number	ChEMBL ID	Part used	References related to the part used	Identification Confidence*	Level 1 ★★★★★<br>Level 2 ★★★★☆<br>Level 3 ★★★☆☆<br>Level 4 ★★☆☆☆<br>Level 5 ★☆☆☆☆	References related to confidence level
**6**	Luteolin 3',7-di-*O*-glucoside	$C_{27}H_{30}O_{16}$	610.15339	InChI=1S/C27H30O16/c28-7-17-20(33)22(35)24(37)26(42-17)39-10-4-12(31)19-13(32)6-14(40-16(19)5-10)9-1-2-11(30)15(3-9)41-27-25(38)23(36)21(34)18(8-29)43-27/h1-6,17-18,20-31,33-38H,7-8H2/t17-,18-,20-,21-,22+,23+,24-,25-,26-,27-/m1/s1	O=C(C1=C(O)C=C(OC2C(O)C(O)C(O)C(CO)O2)C=C1O3)C=C3C4=CC(OC5C(O)C(O)C(O)C(CO)O5)=C(O)C=C4	5490298	52187-80-1	CHEMBL1744031	Leaves, branches, stems, fruits, seeds, olive oil, and OLS	2, 47, 64	2/ HPLC-DAD (Rt + UV); ESI-MS ($[M–H]^-$ m/z 447, fragments) with literature comparison.	Level 2 ★★★★☆	2/ [100]
**7**	Diosmetin (luteolin 4'-methyl ether)	$C_{16}H_{12}O_6$	300.06339	InChI=1S/C16H12O6/c1-21-13-3-2-8(4-10(13)18)14-7-12(20)16-11(19)5-9(17)6-15(16)22-14/h2-7,17-19H,1H3	COC1=C(C=C(C=C1)C2=CC(=O)C3=C(C=C(C=C3O2)O)O)O	5281612	520-34-3	CHEMBL90568	Flowers, pomace, leaves	9, 43, 101	4/ Identification via LC-MS (ESI/APCI, negative/positive polarity) and reliance on literature-reported MS data without experimental spectral validation.	Level 4 ★★☆☆☆	4/ [85]
**8**	Diosmetin-7-*O*-glucoside (luteolin 4'-methyl ether 7-*O*-glucoside)	$C_{22}H_{22}O_{11}$	462.116215	InChI=1S/C22H22O11/c1-30-14-3-2-9(4-11(14)24)15-7-13(26)18-12(25)5-10(6-16(18)32-15)31-22-21(29)20(28)19(27)17(8-23)33-22/h2-7,17,19-25,27-29H,8H2,1H3/t17-,19-,20+,21-,22-/m1/s1	COC1=C(C=C(C=C1)C2=CC(=O)C3=C(C=C(C=C3O2)OC4C(C(C(C(O4)CO)O)O)O)O)O	11016019	20126-59-4	CHEMBL488001	Leaves	73	2/ Analyzed via RP-HPLC-PAD-MS[70], was identified by coelution with a pure reference standard. Retention time, UV spectra, and pseudomolecular/ fragment ion masses matched the reference standard.	Level 2 ★★★★☆	2/ [23]
**9**	Diosmin (luteolin 4′-methyl ether 7-*O*-rutinoside/ diosmetin 7-*O*-rutionoside)	$C_{28}H_{32}O_{15}$	608.174125	InChI=1S/C28H32O15/c1-10-21(32)23(34)25(36)27(40-10)39-9-19-22(33)24(35)26(37)28(43-19)41-12-6-14(30)20-15(31)8-17(42-18(20)7-12)11-3-4-16(38-2)13(29)5-11/h3-8,10,19,21-30,32-37H,9H2,1-2H3/t10-,19+,21-,22+,23+,24-,25+,26+,27+,28+/m0/s1	CC1C(C(C(C(O1)OCC2C(C(C(C(O2)OC3=CC(=C4C(=C3)OC(=CC4=O)C5=CC(=C(C=C5)OC)O)O)O)O)O)O)O)O	5281613	520-27-4	CHEMBL231884	Leaves and OLS	2, 23	2/ Analyzed via RP-HPLC-PAD-MS[70], was identified by coelution with a pure reference standard. Rt, UV spectra, and pseudomolecular/fragment ion masses matched the reference standard.	Level 2 ★★★★☆	2/ [23]
**10**	Luteolin-7-O-rutinoside	$C_{27}H_{30}O_{15}$	594.158475	InChI=1S/C27H30O15/c1-9-20(32)22(34)24(36)26(39-9)38-8-18-21(33)23(35)25(37)27(42-18)40-11-5-14(30)19-15(31)7-16(41-17(19)6-11)10-2-3-12(28)13(29)4-10/h2-7,9,18,20-30,32-37H,8H2,1H3/t9-,18+,20-,21+,22+,23-,24+,25+,26+,27+/m0/s1	CC1C(C(C(C(O1)OCC2C(C(C(C(O2)OC3=CC(=C4C(=C3)OC(=CC4=O)C5=CC(=C(C=C5)O)O)O)O)O)O)O)O)O	14032966	20633-84-5	CHEMBL1714943	Flowers, leaves, stems, fruits, pomace, OOMW, and OLS	9, 93, 101 2	3/ The identification based on Chromatography (Rt) + UV/HRMS/MSMS using HPLC-ESI-MS/MS compared to literature data.<br><br>4/ Identification relied on mass spectrometry, MS/MS fragmentation using UPLC-Q-TOF/MS, and Literature comparison for spectral data.	Level 3 ★★★☆☆<br><br>Level 4 ★★☆☆☆	3/ [79]<br><br>4/ [72]
**11**	Apigenin	$C_{15}H_{10}O_5$	270.052825	InChI=1S/C15H10O5/c16-9-3-1-8(2-4-9)13-7-12(19)15-11(18)5-10(17)6-14(15)20-13/h1-7,16-18H	C1=CC(=CC=C1C2=CC(=O)C3=C(C=C(C=C3O2)O)O)O	5280443	520-36-5	CHEMBL28	Seed, flowers, pomace, olive oil (VOO& EVOO),	9, 33, 60, 83 40	2/ Analysis by LC-SPE-NMR with Rt matching (21.1 min), structural elucidation via 1D (1H and 13C) and 2D NMR (TOCSY) compared with authentic standard.	Level 2 ★★★★☆	2/ [28]

No.	Compound name	Molecular formula	Exact mass	InChI	SMILES	CID	CAS Number	ChEMBL ID	Part used	References related to the part used	Identification Confidence*	Level 1 ★★★★★<br>Level 2 ★★★★☆<br>Level 3 ★★★☆☆<br>Level 4 ★★☆☆☆<br>Level 5 ★☆☆☆☆	References related to confidence level
									leaves, stems, and OOMW				
**12**	Apigenin 7-*O*-glucoside	$C_{21}H_{20}O_{10}$	432.105649	InChI=1S/C21H20O10/c22-8-16-18(26)19(27)20(28)21(31-16)29-11-5-12(24)17-13(25)7-14(30-15(17)6-11)9-1-3-10(23)4-2-9/h1-7,16,18-24,26-28H,8H2/t16-,18-,19+,20-,21-/m1/s1	C1=CC(=CC=C1C2=CC(=O)C3=C(C=C(C=C3O2)OC4C(C(C(C(O4)CO)O)O)O)O)O	5280704	578-74-5	CHEMBL487017	Leaves, fruits, pomace, wood, and OLS	2, 25, 58, 79, 98	3/ Identification based on chromatography RPLC-DAD-ESI-MS and UV data compared to literature, but lacks confirmation with a reference standard or full structural validation.	Level 3 ★★★☆☆	3/ [32]
**13**	Apigenin-7-*O*-glucuronide	$C_{21}H_{18}O_{11}$	446.084915	InChI=1S/C21H18O11/c22-9-3-1-8(2-4-9)13-7-12(24)15-11(23)5-10(6-14(15)31-13)30-21-18(27)16(25)17(26)19(32-21)20(28)29/h1-7,16-19,21-23,25-27H,(H,28,29)/t16-,17-,18+,19-,21+/m0/s1	C1=CC(=CC=C1C2=CC(=O)C3=C(C=C(C=C3O2)OC4C(C(C(C(O4)C(=O)O)O)O)O)O)O	5319484	29741-09-1	CHEMBL254213	Leaves	23	2/ Analyzed via RP-HPLC-PAD-MS[70] was identified by coelution with a pure reference standard, matching Rt, UV spectra, and mass spectrometry data (pseudomolecular/ fragment ions).	Level 2 ★★★★☆	2/ [23]
**14**	Apigenin 7-*O*-rutinoside (rhoifolin/ apigenin-*O*-hexosyl deoxyhexoside)	$C_{27}H_{30}O_{14}$	578.16356	InChI=1S/C27H30O14/c1-10-20(31)22(33)24(35)26(38-10)37-9-18-21(32)23(34)25(36)27(41-18)39-13-6-14(29)19-15(30)8-16(40-17(19)7-13)11-2-4-12(28)5-3-11/h2-8,10,18,20-29,31-36H,9H2,1H3/t10-,18+,20-,21+,22+,23-,24+,25+,26+,27+/m0/s1	CC1C(C(C(C(O1)OCC2C(C(C(C(O2)OC3=CC(=C4C(=C3)OC(=CC4=O)C5=CC=C(C=C5)O)O)O)O)O)O)O)O	9851181	552-57-8	CHEMBL454778	Flowers, fruits, leaves, seeds, OOMW, and OLS	2, 9, 58, 79	2/ Analyzed via RP-HPLC-PAD-MS[70] was identified by coelution with a pure reference standard, matching Rt, UV spectra, and mass spectrometry data (pseudomolecular/ fragment ions).	Level 2 ★★★★☆	2/ [23]
**15**	Apigenin-6,8-C-dihexoside (vicenin)	$C_{27}H_{30}O_{15}$	594.158475	InChI=1S/C27H30O15/c28-6-12-17(32)21(36)23(38)26(41-12)15-19(34)14-10(31)5-11(8-1-3-9(30)4-2-8)40-25(14)16(20(15)35)27-24(39)22(37)18(33)13(7-29)42-27/h1-5,12-13,17-18,21-24,26-30,32-39H,6-7H2/t12-,13-,17-,18-,21+,22+,23-,24-,26+,27+/m1/s1	C1=CC(=CC=C1C2=CC(=O)C3=C(C(=C(C(=C3O2)C4C(C(C(C(O4)CO)O)O)O)O)C5C(C(C(C(O5)CO)O)O)O)O)O	442664	23666-13-9	CHEMBL1442950	Fruit, seed, pomace, OOMW, and OLS	2, 17, 33	2/ Analyzed via RP-HPLC-PAD-MS[70] was identified by coelution with a pure reference standard, matching Rt, UV spectra, and mass spectrometry data (pseudomolecular/ fragment ions).	Level 2 ★★★★☆	2/ [23]
**16**	Apigenin-*O*-dideoxyhexoside-hexoside	$C_{33}H_{40}O_{18}$	724.22147	—	—	NF	NF	NF	Leaves	73	4/ Analyzed using HPLC-PDA-ESI-MS/MS analysis, and the identification relies only on Rt, UV, and mass spectra data.	Level 4 ★★☆☆☆	4/ [73]
**e. Flavonols**													
**1**	Quercetin	$C_{15}H_{10}O_7$	302.042654	InChI=1S/C15H10O7/c16-7-4-10(19)12-11(5-7)22-15(14(21)13(12)20)6-1-2-8(17)9(18)3-6/h1-5,16-19,21H	C1=CC(=C(C=C1C2=C(C(=O)C3=C(C=C(C=C3O2)O)O)O)O)O	5280343	117-39-5	CHEMBL50	Pomace, leaves, stems, branches, bark, fruits, seeds, peel, olive oil (EVOO),	25, 33, 34, 40, 47, 74, 83	2/ Identification by HPLC (Rt 18.3 min.) matched to an authentic standard; UV detection at 335 nm.	Level 2 ★★★★☆	2/ [61]

No.	Compound name	Molecular formula	Exact mass	InChI	SMILES	CID	CAS Number	ChEMBL ID	Part used	References related to the part used	Identification Confidence*	Level 1 ★★★★★<br>Level 2 ★★★★☆<br>Level 3 ★★★☆☆<br>Level 4 ★★☆☆☆<br>Level 5 ★☆☆☆☆	References related to confidence level
									and OOMW				
**2**	Isoquercitrin (quercetin 3-*O*-glucoside)	$C_{21}H_{20}O_{12}$	464.09548	InChI=1S/C21H20O12/c22-6-13-15(27)17(29)18(30)21(32-13)33-20-16(28)14-11(26)4-8(23)5-12(14)31-19(20)7-1-2-9(24)10(25)3-7/h1-5,13,15,17-18,21-27,29-30H,6H2/t13-,15-,17+,18-,21+/m1/s1	C1=CC(=C(C=C1C2=C(C(=O)C3=C(C=C(C=C3O2)O)O)OC4C(C(C(C(O4)CO)O)O)O)O)O	5280804	482-35-9	CHEMBL250450	Flowers, leaves, stems, fruits, and roots	9, 40, 58	2/ Analyzed via RP-HPLC-PAD-MS[70] was identified by coelution with a pure reference standard, matching Rt, UV spectra, and mass spectrometry data (pseudomolecular/ fragment ions).	Level 2 ★★★★☆	2/ [23]
**3**	Rutin (quercetin-3-*O*-rutinoside)	$C_{27}H_{30}O_{16}$	610.15339	InChI=1S/C27H30O16/c1-8-17(32)20(35)22(37)26(40-8)39-7-15-18(33)21(36)23(38)27(42-15)43-25-19(34)16-13(31)5-10(28)6-14(16)41-24(25)9-2-3-11(29)12(30)4-9/h2-6,8,15,17-18,20-23,26-33,35-38H,7H2,1H3/t8-,15+,17-,18+,20+,21-,22+,23+,26+,27-/m0/s1	CC1C(C(C(C(O1)OCC2C(C(C(C(O2)OC3=C(OC4=CC(=CC(=C4C3=O)O)O)C5=CC(=C(C=C5)O)O)O)O)O)O)O)O	5280805	153-18-4	CHEMBL226335	Fruits, fruit peel, seeds, flowers, pomace, leaves, stems, roots, olive oil (VOO), OOMW, and OLS	2, 9, 25, 40, 47, 56, 58, 74, 85, 96	2/ Identification by HPLC (Rt 8.4 min.) matched to an authentic standard; UV detection at 335 nm.	Level 2 ★★★★☆	2/ [61]
**4**	Quercetin 3-*O*-hexuronide (quercetin 3-*O*-glucuronide/ querciturone)	$C_{21}H_{18}O_{13}$	478.074745	InChI=1S/C21H18O13/c22-7-4-10(25)12-11(5-7)32-17(6-1-2-8(23)9(24)3-6)18(13(12)26)33-21-16(29)14(27)15(28)19(34-21)20(30)31/h1-5,14-16,19,21-25,27-29H,(H,30,31)/t14-,15-,16+,19-,21+/m0/s1	C1=CC(=C(C=C1C2=C(C(=O)C3=C(C=C(C=C3O2)O)O)OC4C(C(C(C(O4)C(=O)O)O)O)O)O)O	5274585	22688-79-5	CHEMBL520546	Leaves	66	4/ Identification via HPLC-DAD ESI/MS-TOF.	Level 4 ★★☆☆☆	4/ [66]
**5**	Quercitrin (quercetin-3-*O*-rhamnoside)	$C_{21}H_{20}O_{11}$	448.100564	InChI=1S/C21H20O11/c1-7-15(26)17(28)18(29)21(30-7)32-20-16(27)14-12(25)5-9(22)6-13(14)31-19(20)8-2-3-10(23)11(24)4-8/h2-7,15,17-18,21-26,28-29H,1H3/t7-,15-,17+,18+,21-/m0/s1	CC1C(C(C(C(O1)OC2=C(OC3=CC(=CC(=C3C2=O)O)O)C4=CC(=C(C=C4)O)O)O)O)O	5280459	522-12-3	CHEMBL82242	Flowers, leaves, fruits, seeds, and OOMW	9, 33, 58, 79	2/ Analyzed via RP-HPLC-PAD-MS[70] was identified by coelution with a pure reference standard, matching Rt, UV spectra, and mass spectrometry data (pseudomolecular/ fragment ions).	Level 2 ★★★★☆	2/ [23]
**6**	Quercetin 3-*O*-arabino-glucoside	$C_{26}H_{28}O_{16}$	596.13774	InChI=1S/C26H28O16/c27-9-4-12(30)16-14(5-9)40-23(8-1-2-10(28)11(29)3-8)24(19(16)34)42-26-22(37)20(35)18(33)15(41-26)7-39-25-21(36)17(32)13(31)6-38-25/h1-5,13,15,17-18,20-22,25-33,35-37H,6-7H2/t13-,15+,17-,18+,20-,21+,22+,25-,26-/m0/s1	C1[C@@H]([C@@H]([C@H]([C@@H](O1)OC[C@@H]2[C@H]([C@@H]([C@H]([C@@H](O2)OC3=C(OC4=CC(=CC(=C4C3=O)O)O)C5=CC(=C(C=C5)O)	5484066	23284-18-6	NF	Fruit and olive oil	102	2/ The identification via HPLC (Rt 9.7 min. matched to an authentic standard); UV detection at 335 nm.	Level 2 ★★★★☆	2/ [61]

No.	Compound name	Molecular formula	Exact mass	InChI	SMILES	CID	CAS Number	ChEMBL ID	Part used	References related to the part used	Identification Confidence*	Level 1 ★★★★★<br>Level 2 ★★★★☆<br>Level 3 ★★★☆☆<br>Level 4 ★★☆☆☆<br>Level 5 ★☆☆☆☆	References related to confidence level
					O)O)O)O)O)O)O								
**7**	Quercetin 3,4'-*O*-diglucoside	$C_{27}H_{30}O_{17}$	626.148305	InChI=1S/C27H30O17/c28-6-14-17(33)20(36)22(38)26(42-14)41-12-2-1-8(3-10(12)31)24-25(19(35)16-11(32)4-9(30)5-13(16)40-24)44-27-23(39)21(37)18(34)15(7-29)43-27/h1-5,14-15,17-18,20-23,26-34,36-39H,6-7H2/t14-,15-,17-,18-,20+,21+,22-,23-,26-,27+/m1/s1	C1=CC(=C(C=C1C2=C(C(=O)C3=C(C=C(C=C3O2)O)O)OC4C(C(C(C(O4)CO)O)O)O)OC5C(C(C(C(O5)CO)O)O)O	5320835	29125-80-2	CHEMBL1098352	Leaves	[73]	4/ Analyzed using HPLC-PDA-ESI-MS/MS analysis, and the identification based on Rt + UV spectrum, and MS/MS fragmentation pattern matched to MassBank database.	Level 4 ★★☆☆☆	4/ [73]
**8**	Quercetin-*O*-hexoside-*O*-deoxyhexoside	$C_{27}H_{30}O_{16}$	610.15339	—	—	NF	NF	NF	Leaves	[73]	4/ Analyzed using HPLC-PDA-ESI-MS/MS analysis, and the identification based on Rt + UV spectrum, and MS/MS fragmentation pattern matched to MassBank database.	Level 4 ★★☆☆☆	4/ [73]
**9**	Kampferol	$C_{15}H_{10}O_6$	286.04774	InChI=1S/C15H10O6/c16-8-3-1-7(2-4-8)15-14(20)13(19)12-10(18)5-9(17)6-11(12)21-15/h1-6,16-18,20H	C1=CC(=CC=C1C2=C(C(=O)C3=C(C=C(C=C3O2)O)O)O)O	5280863	520-18-3	CHEMBL150	Leaves and OOMW	[60]	2/ Identification via HPLC-ESI-MS/MS matched (Rt) and MS/MS fragmentation data, and compared to the standard compound.<br><br>4/ Identification via UPLC-ESI-Q-TOF based on RT and HRMS (m/z), but no MS/MS, UV, or NMR data provided to confirm structural elucidation.	Level 2 ★★★★☆<br><br>Level 4 ★★☆☆☆	2/ [79]<br><br>4/ [47]
**10**	Kaempferol-7-*O*-glucoside	$C_{21}H_{20}O_{11}$	448.100564	InChI=1S/C21H20O11/c22-7-13-15(25)17(27)19(29)21(32-13)30-10-5-11(24)14-12(6-10)31-20(18(28)16(14)26)8-1-3-9(23)4-2-8/h1-6,13,15,17,19,21-25,27-29H,7H2/t13-,15-,17+,19-,21-/m1/s1	C1=CC(=CC=C1C2=C(C(=O)C3=C(C=C(C=C3O2)OC4C(C(C(C(O4)CO)O)O)O)O)O)O	10095180	16290-07-6	CHEMBL469441	Leaves, seeds, branches, and fruits	[47, 79]	2/ Identification via HPLC-ESI-MS/MS matched (Rt) and MS/MS fragmentation data, and compared to the standard compound.	Level 2 ★★★★☆	2/ [79]
**11**	Syringetin *O*-di-glucoside	$C_{29}H_{34}O_{18}$	670.17452	InChI=1S/C29H34O18/c1-41-13-3-9(4-14(42-2)18(13)33)26-27(21(36)17-11(32)5-10(31)6-12(17)44-26)47-29-25(40)23(38)20(35)16(46-29)8-43-28-24(39)22(37)19(34)15(7-30)45-28/h3-6,15-16,19-20,22-25,28-35,37-40H,7-8H2,1-2H3/t15-,16-,19-,20-,22+,23+,24-,25-,28-,29+/m1/s1	COC1=C(O)C(OC)=CC(C2=C(OC3C(O)C(O)C(O)C(O3)COC4C(O)C(O)C(O)C(O4)CO)C(C5=C(O)C=C(O)C=C5O2)=O)=C1	NF	NF	NF	Leaves	[66]	4/ Identification via HPLC-DAD ESI/MS-TOF.	Level 4 ★★☆☆☆	4/ [66]

No.	Compound name	Molecular formula	Exact mass	InChI	SMILES	CID	CAS Number	ChEMBL ID	Part used	References related to the part used	Identification Confidence*	Level 1 ★★★★★<br>Level 2 ★★★★☆<br>Level 3 ★★★☆☆<br>Level 4 ★★☆☆☆<br>Level 5 ★☆☆☆☆	References related to confidence level
**12**	Quercetin dimer	$C_{30}H_{18}O_{14}$	602.06966	InChI=1S/C30H18O14/c31-13-7-17(35)23-21(9-13)41-27(26(38)25(23)37)11-1-4-19-20(5-11)42-29(40)28(39)24-18(36)8-14(32)10-22(24)44-30(29,43-19)12-2-3-15(33)16(34)6-12/h1-10,31-36,38,40H	C1=CC2=C(C=C1C3=C(C(=O)C4=C(C=C(C=C4O3)O)O)O)OC5(C(=O)C6=C(C=C(C=C6OC5(O2)C7=CC(=C(C=C7)O)O)O)O)O	71534128	167276-19-9	NF	Leaves	[66]	4/ Analyzed using HPLC-DAD-ESI/MS-TOF analysis, and identification based on Rt, MS spectra, and compared to reference literature.	Level 4 ★★☆☆☆	4/ [66]
**13**	Resinoside	$C_{31}H_{34}O_{13}$	614.199945	InChI=1S/C31H34O13/c1-31(2,40)16-7-3-15(4-8-16)29(39)41-13-21-23(35)25(37)26(38)30(43-21)44-28-24(36)22-19(34)11-18(33)12-20(22)42-27(28)14-5-9-17(32)10-6-14/h3,5-6,9-12,16,21,23,25-26,30,32-35,37-38,40H,4,7-8,13H2,1-2H3/t16?,21-,23-,25+,26-,30+/m1/s1	CC(C)(C1CCC(=CC1)C(=O)OC[C@@H]2[C@H]([C@@H]([C@H]([C@@H](O2)OC3=C(OC4=CC(=CC(=C4C3=O)O)O)C5=CC=C(C=C5)O)O)O)O)O	15172373	NF	NF	Leaves	[103]	4/ Analyzed using HPLC-ESI-TOF-MS analysis, and identification based on RT + HRMS match to literature.	Level 4 ★★☆☆☆	4/ [104]
**f. Isoflavones**													
**1**	Spherobioside	$C_{27}H_{30}O_{14}$	578.16356	InChI=1S/C27H30O14/c1-10-19(30)22(33)24(35)26(39-10)38-9-17-21(32)23(34)25(36)27(41-17)40-13-6-15(29)18-16(7-13)37-8-14(20(18)31)11-2-4-12(28)5-3-11/h2-8,10,17,19,21-30,32-36H,9H2,1H3/t10-,17+,19-,21+,22+,23-,24+,25+,26+,27+/m0/s1	C[C@H]1[C@@H]([C@H]([C@H]([C@@H](O1)OC[C@@H]2[C@H]([C@@H]([C@H]([C@@H](O2)OC3=CC(=C4C(=C3)OC=C(C4=O)C5=CC=C(C=C5)O)O)O)O)O)O)O	76315025	14988-20-6	CHEMBL2227762	Leaves	[64]	4/ Analyzed using RP/HPLC-Q-TOF MS/MS analysis, and the identification based on precursor ion mass, Rt, and MS/MS fragmentation pattern matched against spectral libraries (NIST20, LipidBlast, MoNA).	Level 4 ★★☆☆☆	4/ [64]
**g. Anthocyanins**													
**1**	Cyanidin 3-*O*-glucoside	$C_{21}H_{21}O_{11}^{+}$	449.10839	InChI=1S/C21H20O11/c22-7-16-17(27)18(28)19(29)21(32-16)31-15-6-10-12(25)4-9(23)5-14(10)30-20(15)8-1-2-11(24)13(26)3-8/h1-6,16-19,21-22,27-29H,7H2,(H3-,23,24,25,26)/p+1/t16-,17-,18+,19-,21-/m1/s1	C1=CC(=C(C=C1C2=[O+]C3=CC(=CC(=C3C=C2OC4C(C(C(C(O4)CO)O)O)O)O)O)O)O.[Cl-]	12303220	7084-24-4	NF	Fruits, pomace, skin, and leaves	[58, 88]	1/ Isolated by different chromatographic techniques (TLC, HPLC), elucidated through 1D NMR (1H and 13C NMR), 2D NMR (COSY), then identified by MS was used.	Level 1 ★★★★★	1/ [105]

No.	Compound name	Molecular formula	Exact mass	InChI	SMILES	CID	CAS Number	ChEMBL ID	Part used	References related to the part used	Identification Confidence*	Level 1 ★★★★★<br>Level 2 ★★★★☆<br>Level 3 ★★★☆☆<br>Level 4 ★★☆☆☆<br>Level 5 ★☆☆☆☆	References related to confidence level
2	Cyanidin 3-*O*-diglucoside	$C_{27}H_{31}O_{16}^{+}$	611.161215	InChI=1S/C27H30O16/c28-7-17-19(33)21(35)23(37)26(42-17)39-8-18-20(34)22(36)24(38)27(43-18)41-16-6-11-13(31)4-10(29)5-15(11)40-25(16)9-1-2-12(30)14(32)3-9/h1-6,17-24,26-28,33-38H,7-8H2,(H3-,29,30,31,32)/p+1	OC(C(COC1C(O)C(O)C(O)C(CO)O1)O2)C(O)C(O)C2OC3=CC4=C(O)C=C(O)C=C4[O+]=C3C5=CC(O)=C(O)C=C5	NF	NF	NF	Olive skin	[65]	4/ The Identification via MS/MS; LTQXL; Thermo Scientific, Waltham, MA, USA with heated electrospray ionization (HESI) using external standards [cyanidin-3-O-glucoside] as equivalents of related substances.	Level 4 ★★☆☆☆	4/ [65]
3	Cyanidin 3,5-*O*-sophoroside	$C_{39}H_{51}O_{26}^{+}$	935.266865	InChI=1S/C39H50O26/c40-7-19-23(45)27(49)31(53)36(62-19)57-9-21-25(47)29(51)33(55)38(64-21)60-17-5-12(42)4-16-13(17)6-18(35(59-16)11-1-2-14(43)15(44)3-11)61-39-34(56)30(52)26(48)22(65-39)10-58-37-32(54)28(50)24(46)20(8-41)63-37/h1-6,19-34,36-41,45-56H,7-10H2,(H2-,42,43,44)/p+1	OC1=CC(OC2C(O)C(O)C(O)C(COC3C(O)C(O)C(O)C(CO)O3)O2)=C(C=C4OC5OC(C(C(C5O)O)O)COC6OC(C(C(C6O)O)O)CO)C([O+]=C4C7=CC(O)=C(O)C=C7)=C1	NF	NF	NF	Olive skin	[65]	4/ The Identification via MS/MS; LTQXL; Thermo Scientific, Waltham, MA, USA with heated electrospray ionization (HESI) using external standards [cyanidin-3-O-glucoside] as equivalents of related substances.	Level 4 ★★☆☆☆	4/ [65]
4	Cyanidin 3-*O*-rutinoside	$C_{27}H_{31}O_{15}^{+}$	595.1663	InChI=1S/C27H30O15/c1-9-19(32)21(34)23(36)26(39-9)38-8-18-20(33)22(35)24(37)27(42-18)41-17-7-12-14(30)5-11(28)6-16(12)40-25(17)10-2-3-13(29)15(31)4-10/h2-7,9,18-24,26-27,32-37H,8H2,1H3,(H3-,28,29,30,31)/p+1/t9-,18+,19-,20+,21+,22-,23+,24+,26+,27+/m0/s1	CC1C(C(C(C(O1)OCC2C(C(C(C(O2)OC3=CC4=C(C=C(C=C4[O+]=C3C5=CC(=C(C=C5)O)O)O)O)O)O)O)O)O	441674	28338-59-2	CHEMBL505251	Fruits and pomace	[50, 106]	3/ Identification based on chromatography (RPLC-DAD-ESI-MS), UV, and MS data compared to literature, without a reference standard or structural validation.	Level 3 ★★★☆☆	3/ [32]
5	Delphinidin 3-*O*-glucoside	$C_{21}H_{21}O_{12}^{+}$	465.103305	InChI=1S/C21H20O12/c22-6-15-17(28)18(29)19(30)21(33-15)32-14-5-9-10(24)3-8(23)4-13(9)31-20(14)7-1-11(25)16(27)12(26)2-7/h1-5,15,17-19,21-22,28-30H,6H2,(H4-,23,24,25,26,27)/p+1/t15-,17-,18+,19-,21-/m1/s1	C1=C(C=C(C(=C1O)O)O)C2=[O+]C3=CC(=CC(=C3C=C2OC4C(C(C(C(O4)CO)O)O)O)O)O	443650	50986-17-9	CHEMBL518846	Fruits	[65]	3/ Identification based on chromatography (RPLC-DAD-ESI-MS), UV, and MS data compared to literature, without a reference standard or structural validation.	Level 3 ★★★☆☆	3/ [32]
**h. Chalcones**													
1	Olivin	$C_{17}H_{16}O_6$	316.09469	InChI=1S/C17H16O6/c1-9(5-10-3-4-12(19)15(6-10)23-2)17(22)16-13(20)7-11(18)8-14(16)21/h3-8,18-21H,1-2H3/b9-5+	COC1=C(O)C=CC(\C=C(/C)C(=O)C2=C(O)C=C(O)C=C2O)=C1	NF	NF	NF	Leaves	[107]	5/ No techniques (e.g., NMR, HPLC-MS, co-chromatography) are mentioned to validate olivin's presence in the extracted samples.	Level 5 ★☆☆☆☆	5/ [108]

No.	Compound name	Molecular formula	Exact mass	InChI	SMILES	CID	CAS Number	ChEMBL ID	Part used	References related to the part used	Identification Confidence*	Level 1 ★★★★★ Level 2 ★★★★☆ Level 3 ★★★☆☆ Level 4 ★★☆☆☆ Level 5 ★☆☆☆☆	References related to confidence level
**2**	Olivine diglucoside	$C_{29}H_{36}O_{17}$	656.195255	—	—	NF	NF	NF	Leaves	[107]	5/ No techniques (e.g., NMR, HPLC-MS, co-chromatography) are mentioned to validate olivin's presence in the extracted samples.	Level 5 ★☆☆☆☆	5/ [108]
**6- Iridoids, glycosides, and derivatives**													
**1**	Loganin (loganoside)	$C_{17}H_{26}O_{10}$	390.1526	InChI=1S/C17H26O10/c1-6-9(19)3-7-8(15(23)24-2)5-25-16(11(6)7)27-17-14(22)13(21)12(20)10(4-18)26-17/h5-7,9-14,16-22H,3-4H2,1-2H3/t6-,7+,9-,10+,11+,12+,13-,14+,16-,17-/m0/s1	CC1C(CC2C1C(OC=C2C(=O)OC)OC3C(C(C(C(O3)CO)O)O)O)O	87691	18524-94-2	CHEMBL1081584	Leaves, stems, roots, fruits, seeds, pomace, wood, and OLS	[1, 2, 25, 33, 40]	3/ Identification based on chromatography RPLC-DAD-ESI-MS and UV/MS data compared to literature, but lacking confirmation with a reference standard	Level 3 ★★★☆☆	3/ [32]
**2**	Loganin glucoside	$C_{23}H_{36}O_{15}$	552.205425	InChI=1S/C23H36O15/c1-7-10(25)3-8-9(20(32)33-2)5-34-21(13(7)8)38-23-19(31)17(29)15(27)12(37-23)6-35-22-18(30)16(28)14(26)11(4-24)36-22/h5,7-8,10-19,21-31H,3-4,6H2,1-2H3	CC1C(O)CC2C1C(OC3C(O)C(O)C(O)C(COC4C(O)C(O)C(O)C(CO)O4)O3)OC=C2C(OC)=O	NF	NF	NF	Pomace and wood	[2, 25]	4/ Identification relied on mass spectrometry, MS/MS fragmentation using UPLC-Q-TOF/MS, and Literature comparison for spectral data.	Level 4 ★★☆☆☆	4/ [72]
**3**	Loganic acid	$C_{16}H_{24}O_{10}$	376.13695	InChI=1S/C16H24O10/c1-5-8(18)2-6-7(14(22)23)4-24-15(10(5)6)26-16-13(21)12(20)11(19)9(3-17)25-16/h4-6,8-13,15-21H,2-3H2,1H3,(H,22,23)/t5-,6+,8-,9+,10+,11+,12-,13+,15-,16-/m0/s1	CC1C(CC2C1C(OC=C2C(=O)O)OC3C(C(C(C(O3)CO)O)O)O)O	89640	22255-40-9	CHEMBL1081585	Roots, flowers, fruits, seeds, pomace, leaves, and OLS	[1, 2, 9, 25, 33, 73]	3/ The identification based on Chromatography (Rt) + UV/HRMS/MSMS using HPLC-ESI-MS/MS compared to literature data.<br><br>3/ Identification relied on mass spectrometry and MS/MS fragments using RP-HPLC-DAD-ESI-QTOF-MS/MS and UV/HRMS/MSMS, with literature validation for Rt and spectral data.	Level 3 ★★★☆☆	3/ [79]<br><br>3/ [2]
**4**	Loganic acid glucoside	$C_{22}H_{34}O_{15}$	538.189775	InChI=1S/C22H34O15/c1-6-9(24)2-7-8(19(31)32)4-33-20(12(6)7)37-22-18(30)16(28)14(26)11(36-22)5-34-21-17(29)15(27)13(25)10(3-23)35-21/h4,6-7,9-18,20-30H,2-3,5H2,1H3,(H,31,32)/t6-,7+,9-,10+,11+,12+,13+,14+,15-,16-,17+,18+,20-,21+,22-/m0/s1	CC1C(CC2C1C(OC=C2C(=O)O)OC3C(C(C(C(O3)COC4C(C(C(C(O4)CO)O)O)O)O)O)O)O	101696514	176226-39-4	NF	Stems, roots, fruits, pomace, wood, and OLS	[2, 25, 40, 76]	3/ Identification was based on chromatographic (Rt) and spectroscopic (HRMS/MS/MS) data and UV (via DAD) compared to literature.<br><br>3/ Identification relied on mass spectrometry and MS/MS fragments using RP-HPLC-DAD-ESI-QTOF-MS/MS and UV/HRMS/MSMS, with literature validation for Rt and spectral data.	Level 3 ★★★☆☆	3/ [76]<br><br>3/ [2]

No.	Compound name	Molecular formula	Exact mass	InChI	SMILES	CID	CAS Number	ChEMBL ID	Part used	References related to the part used	Identification Confidence*	Level 1 ★★★★★<br>Level 2 ★★★★☆<br>Level 3 ★★★☆☆<br>Level 4 ★★☆☆☆<br>Level 5 ★☆☆☆☆	References related to confidence level
**5**	7-Deoxyloganic acid	$C_{16}H_{24}O_9$	360.142035	InChI=1S/C16H24O9/c1-6-2-3-7-8(14(21)22)5-23-15(10(6)7)25-16-13(20)12(19)11(18)9(4-17)24-16/h5-7,9-13,15-20H,2-4H2,1H3,(H,21,22)/t6-,7+,9+,10+,11+,12-,13+,15-,16-/m0/s1	CC1CCC2C1C(OC=C2C(=O)O)OC3C(C(C(C(O3)CO)O)O)O	443322	22487-36-1	NF	Roots, flowers, fruit, seed, pomace, wood, and OLS	[1, 2, 9, 25, 33, 40]	3/ Identification via HPLC-DAD and LC-ESI-QTOF-MS/MS, relies on chromatographic alignment (Rt) and spectrometric data (HRMS/MSMS) compared to literature data.<br><br>3/ Identification relied on mass spectrometry and MS/MS fragments using RP-HPLC-DAD-ESI-QTOF-MS/MS and UV/HRMS/MSMS, with literature validation for Rt and spectral data.	Level 3 ★★★☆☆	3/ [1]<br><br>3/ [2]
**6**	Deoxyloganic acid lauryl ester	C23H38O5	394.27192	InChI=1S/C23H38O5/c1-3-4-5-6-7-8-9-10-11-14-27-22(24)15-20-18(2)12-13-19(20)21-16-26-17-28-23(21)25/h15-16,18-19,23,25H,3-14,17H2,1-2H3/b20-15+	OC1C(C2CCC(C)/C2=C\C(OCCCCCCCCCCC)=O)=COCO1	NF	NF	NF	Pomace	[109]	1/ 2D NMR Spectroscopy (HMQC, HMBC, COSY, DEPT-90), ESI-MS, IR Spectroscopy, and UV Spectroscopy techniques were used, and NMR data compared to literature iridoids (known deoxyloganic acid isomers).	Level 1 ★★★★★	1/ [109]

7- Secoiridoids, glycosides, and derivatives

a. Elenolic acid/ elenolate-type secoiridoids

No.	Compound name	Molecular formula	Exact mass	InChI	SMILES	CID	CAS Number	ChEMBL ID	Part used	References related to the part used	Identification Confidence*	Level	References related to confidence level
**1**	Deoxyelenolic acid (desoxyelenolic acid)	$C_{11}H_{14}O_5$	226.084125	InChI=1S/C11H14O5/c1-7-9(5-13)8(3-4-12)10(6-16-7)11(14)15-2/h4-9H,3H2,1-2H3	CC1C(C=O)C(CC([H])=O)C(C(OC)=O)=CO1	NF	NF	NF	Leaves, seeds, and fruit	[85]	4/ Identification based only on literature comparison using LC-ESI-MS and LC-APCI-MS.	Level 4 ★★☆☆☆	4/ [85]
**2**	Elenolic acid (elenaic acid/ EA)	$C_{11}H_{14}O_6$	242.07904	InChI=1S/C11H14O6/c1-6-8(4-12)7(3-10(13)14)9(5-17-6)11(15)16-2/h4-8H,3H2,1-2H3,(H,13,14)/t6-,7-,8+/m0/s1	CC1C(C(C(=CO1)C(=O)OC)CC(=O)O)C=O	169607	34422-12-3	NF	Pomace, leaves, olive oil (EVOO), and OOMW	[25, 73, 110]	2/ Analysis by LC-SPE-NMR with Rt matching (15.4 min for isomer 5S,8R,9S; 16.5 min for 5S,8S,9S), structural elucidation via 1D (1H and 13C) and 2D NMR (TOCSY) compared with prior literature for structural validation rather than direct comparison with a standard.	Level 2 ★★★★☆	2/ [28]
**3**	Elenolic acid methylester	$C_{12}H_{16}O_6$	256.09469	InChI=1S/C12H16O6/c1-7-9(5-13)8(4-11(14)16-2)10(6-18-7)12(15)17-3/h5-9H,4H2,1-3H3	CC1C(C=O)C(CC(OC)=O)C(C(OC)=O)=CO1	NF	NF	NF	Fruit, skin, leaves, olive oil (VOO)	[85]	4/ Identification based only on literature comparison using LC-ESI-MS and LC-APCI-MS.	Level 4 ★★☆☆☆	4/ [85]
**4**	Hydroxyelenolic acid (hydroxy-EA)	$C_{11}H_{14}O_7$	258.073955	InChI=1S/C11H14O7/c1-17-11(16)8-5-18-9(4-13)7(3-12)6(8)2-10(14)15/h3,5-7,9,13H,2,4H2,1H3,(H,14,15)	O=C(OC)C1=COC(C(C=O)C1CC(O)=O)CO	NF	NF	NF	Fruit, skin, leaves, and olive oil (VOO)	[85]	4/ Identification based only on literature comparison using LC-ESI-MS and LC-APCI-MS.	Level 4 ★★☆☆☆	4/ [85]

No.	Compound name	Molecular formula	Exact mass	InChI	SMILES	CID	CAS Number	ChEMBL ID	Part used	References related to the part used	Identification Confidence*	Level 1 ★★★★★<br>Level 2 ★★★★☆<br>Level 3 ★★★☆☆<br>Level 4 ★★☆☆☆<br>Level 5 ★☆☆☆☆	References related to confidence level
**5**	Decarboxymethyl elenolic acid (EDA)	$C_9H_{12}O_4$	184.07356	InChI=1S/C9H12O4/c1-2-7(6-11)8(3-4-10)5-9(12)13/h2,4,6,8H,3,5H2,1H3,(H,12,13)/b7-2-	O=CCC(CC(O)=O)/C(C=O)=C\C	NF	NF	NF	Leaves, wood, skin, flowers, fruit, olive oil (VOO), seeds, and OOMW	9, 85, 111	2/ Analysis by LC-SPE-NMR with Rt matching (8.4 min), structural elucidation via 1D (1H and 13C) and 2D NMR (TOCSY) compared with prior literature for spectral assignment.	Level 2 ★★★★☆	2/ [28]
**6**	Hydroxy-EDA	$C_9H_{12}O_5$	200.068475	InChI=1S/C9H12O5/c1-2-6(5-10)7(3-8(11)12)4-9(13)14/h2,5,7H,3-4H2,1H3,(H,11,12)(H,13,14)/b6-2-	O=C(CC(CC(O)=O)/C(C=O)=C\C)O	NF	NF	NF	Fruit, flowers, leaves, skin, pomace, and OOMW	9, 85	2/ The structure elucidation of purified compounds was based on NMR (1D; 1H and 13C and 2D; COSY, HSQC-DEPT, and HMBC). Their presence in diverse EVOOs was verified by UHPLC-HRMS.	Level 2 ★★★★☆	2/ [112]
**7**	Hydroxytyrosil-elenolate (hydroxytyrosol-elenolate)	$C_{20}H_{24}O_8$	392.14712	InChI=1S/C20H24O8/c1-12-16(10-21)15(17(11-27-12)20(25)26-2)9-19(24)28-18(23)8-5-13-3-6-14(22)7-4-13/h3-4,6-7,10-12,15-16,18,22-23H,5,8-9H2,1-2H3	CC1C(C=O)C(CC(OC(O)CCC2=CC=C(O)C=C2)=O)C(C(OC)=O)=CO1	NF	NF	NF	Leaves, fruits, seeds, and olive oil (EVOO)	33	4/ Identification based on HPLC-UV Rt matching in HPLC chromatograms, using indirect reference via comparison with authentic samples.	Level 4 ★★☆☆☆	4/ [113]
**8**	Hydrogenated EA	$C_{11}H_{16}O_6$	244.09469	InChI=1S/C11H16O6/c1-6-8(4-12)7(3-10(13)14)9(5-17-6)11(15)16-2/h5-8,12H,3-4H2,1-2H3,(H,13,14)	CC1C(CO)C(CC(O)=O)C(C(OC)=O)=CO1	NF	NF	NF	Pomace and leaves	43, 103	4/ Identification via LC-ESI-QToF-MS that based on chromatographic Rt and HR-MS/MS spectral data.	Level 4 ★★☆☆☆	4/ [43]
**9**	Hydrogenated Elenolic acid decarboxymethylated (Hydrogenated EDA)	$C_9H_{14}O_4$	186.08921	InChI=1S/C9H14O4/c1-2-7(6-11)8(3-4-10)5-9(12)13/h2,4,8,11H,3,5-6H2,1H3,(H,12,13)/b7-2-	O=CCC(CC(O)=O)/C(CO)=C\C	NF	NF	NF	OOMW	111	4/ Identification via MALDI-TOF/TOF MS and MS/MS fragmentation was performed to confirm the structure.	Level 4 ★★☆☆☆	4/ [111]
**10**	Monohydrated-EDA	$C_9H_{14}O_5$	202.084125	InChI=1S/C9H14O5/c1-5-8(9(12)13)6(2-3-14-5)4-7(10)11/h2-3,5-6,8-9,12-13H,4H2,1H3,(H,10,11)	CC1C(C(O)O)C(CC(O)=O)C=CO1	NF	NF	NF	OOMW	111	4/ Identification via MALDI-TOF/TOF MS and MS/MS fragmentation was performed to confirm the structure.	Level 4 ★★☆☆☆	4/ [111]
**b. Oleoside and secologanoside-types secoiridoids and derivatives**													
**1**	Oleoside	$C_{16}H_{22}O_{11}$	390.116215	InChI=1S/C16H22O11/c1-2-6-7(3-10(18)19)8(14(23)24)5-25-15(6)27-16-13(22)12(21)11(20)9(4-17)26-16/h2,5,7,9,11-13,15-17,20-22H,3-4H2,1H3,(H,18,19)(H,23,24)/b6-2+/t7-,9+,11+,12-,13+,15-,16-/m0/s1	C/C=C/1\[C@@H](C(=CO[C@H]1O[C@H]2[C@@H]([C@H]([C@@H]([C@H](O2)CO)O)O)O)C(=O)O)CC(=O)O	101042548	178600-68-5	CHEMBL4174698	Roots, flowers, fruits, seeds, pomace, leaves, stems, wood, bark, OOMW, and OLS	1, 2, 9, 25, 34, 40	3/ Identification based on chromatography RPLC–DAD–ESI-MS, UV, and MS data compared to literature, without a reference standard or structural validation	Level 3 ★★★☆☆	3/ [32]

No.	Compound name	Molecular formula	Exact mass	InChI	SMILES	CID	CAS Number	ChEMBL ID	Part used	References related to the part used	Identification Confidence*	Level 1 ★★★★★<br>Level 2 ★★★★☆<br>Level 3 ★★★☆☆<br>Level 4 ★★☆☆☆<br>Level 5 ★☆☆☆☆	References related to confidence level
**2**	Elenolic acid 2-*O*-glucoside (oleoside 11-methyl ester)	$C_{17}H_{24}O_{11}$	404.131865	InChI=1S/C17H24O11/c1-3-7-8(4-11(19)20)9(15(24)25-2)6-26-16(7)28-17-14(23)13(22)12(21)10(5-18)27-17/h3,6,8,10,12-14,16-18,21-23H,4-5H2,1-2H3,(H,19,20)/b7-3+/t8-,10+,12+,13-,14+,16-,17-/m0/s1	CC=C1C(C(=COC1OC2C(C(C(C(O2)CO)O)O)O)C(=O)OC)CC(=O)O	10692563	60539-23-3	NF	Roots, flowers, fruit, pomace, seed, leaves, stems, wood, bark, and OOMW	[1 9, 17, 24, 25, 34, 40, 101]	1/ Isolated by different chromatographic techniques (column chromatography, TLC, HPLC, PHPLC), elucidated through 1D NMR (1H and 13C NMR), 2D NMR (COSY), identified by MS and electrospray mass spectrometry.	Level 1 ★★★★★	1/ [105]
**3**	Elenolic acid diglucoside (7-β-1-D-glucopyranosyl-11-methyloleoside/ glucosyl-methyloleoside)	$C_{23}H_{34}O_{16}$	566.18469	InChI=1S/C23H34O16/c1-3-8-9(4-13(26)38-22-18(31)16(29)14(27)11(5-24)36-22)10(20(33)34-2)7-35-21(8)39-23-19(32)17(30)15(28)12(6-25)37-23/h3,7,9,11-12,14-19,21-25,27-32H,4-6H2,1-2H3/b8-3-/t9-,11+,12+,14+,15+,16-,17-,18+,19+,21-,22-,23-/m0/s1	CC=C1C(C(=COC1OC2C(C(C(C(O2)CO)O)O)O)C(=O)OC)CC(=O)OC3C(C(C(C(O3)CO)O)O)O	14080694	NF	NF	Flowers, fruits, leaves, seeds, and roots	[9, 40, 64, 77, 114]	4/ Identification relied on mass spectrometry, MS/MS fragmentation using UPLC-Q-TOF/MS, and Literature comparison for spectral data.<br><br>4/ Analyzed using RP/HPLC-Q-TOF MS/MS analysis, and the identification based on precursor ion mass, Rt, and MS/MS fragmentation pattern matched against spectral libraries (NIST20, LipidBlast, MoNA).	Level 4 ★★☆☆☆	4/ [72]<br><br>4/ [64]
**4**	Elenolic acid hexoside derivative	$C_{19}H_{26}O_{13}$	462.137345	—	—	NF	NF	NF	Roots, leaves, wood, and OLS	[1, 2, 9]	3/ Identification relied on mass spectrometry and MS/MS fragments using RP-HPLC-DAD-ESI-QTOF-MS/MS and UV-vis data, with literature comparison.	Level 3 ★★★☆☆	3/ [2]
**5**	Oleoside methyl ester derivative	$C_{18}H_{30}O_{11}$	422.178815	—	—	NF	NF	NF	Roots, leaves, wood, and OLS	[1, 2]	3/ Identification via HPLC-DAD and LC-ESI-QTOF-MS/MS, relies on chromatographic alignment (Rt) and spectrometric data (HRMS/MSMS) compared to literature data.<br><br>3/ Identification relied on mass spectrometry and MS/MS fragments using RP-HPLC-DAD-ESI-QTOF-MS/MS and UV/HRMS/MSMS, with literature validation for Rt and spectral data.	Level 3 ★★★☆☆	3/ [1]<br><br>3/ [2]
**6**	Oleoside dimethyl ester	$C_{18}H_{26}O_{11}$	418.147515	InChI=1S/C18H26O11/c1-4-8-9(5-12(20)25-2)10(16(24)26-3)7-27-17(8)29-18-15(23)14(22)13(21)11(6-19)28-18/h4,7,9,11,13-15,17-19,21-23H,5-6H2,1-3H3/b8-4-	CC=C1C(C(=COC1OC2C(C(C(C(O2)CO)O)O)O)C(=O)OC)CC(=O)OC	10788149	30164-95-5	CHEMBL4162902	Fruits and pomace	[25, 114]	3/ The Identification via chromatographic and spectroscopic data compared to literature.	Level 3 ★★★☆☆	3/ [115]

No.	Compound name	Molecular formula	Exact mass	InChI	SMILES	CID	CAS Number	ChEMBL ID	Part used	References related to the part used	Identification Confidence*	Level 1 ★★★★★<br>Level 2 ★★★★☆<br>Level 3 ★★★☆☆<br>Level 4 ★★☆☆☆<br>Level 5 ★☆☆☆☆	References related to confidence level
**7**	Oleoside glucoside (6'-β-glucopyranosyl-oleoside)	$C_{22}H_{32}O_{16}$	552.16904	InChI=1S/C22H32O16/c1-2-7-8(3-12(25)37-21-17(30)15(28)13(26)10(4-23)35-21)9(19(32)33)6-34-20(7)38-22-18(31)16(29)14(27)11(5-24)36-22/h2,6,8,10-11,13-18,20-24,26-31H,3-5H2,1H3,(H,32,33)/b7-2+	OCC1C(O)C(O)C(O)C(OC(CC(C(C(O)=O)=COC/2OC3C(O)C(O)C(O)C(CO)O3)C2=C/C)=O)O1	NF	NF	NF	Fruits, pomace, leaves, and seeds	[25, 33, 116]	4/ Identification relied on mass spectrometry and MS/MS fragmentation using UPLC-Q-TOF/MS, and Literature comparison for spectral data.	Level 4 ★★☆☆☆	4/ [72]
**8**	Oleoside diglucoside	$C_{28}H_{42}O_{21}$	714.221865	InChI=1S/C28H42O21/c1-2-8-9(3-14(32)47-26-21(39)18(36)15(33)11(4-29)44-26)10(24(42)48-27-22(40)19(37)16(34)12(5-30)45-27)7-43-25(8)49-28-23(41)20(38)17(35)13(6-31)46-28/h2,7,9,11-13,15-23,25-31,33-41H,3-6H2,1H3/b8-2+	OCC1C(O)C(O)C(O)C(OC(CC(C(C(OC2C(O)C(O)C(O)C(CO)O2)=O)=COC/3OC4C(O)C(O)C(O)C(CO)O4)C3=C/C)=O)O1	NF	NF	NF	Fruits and pomace	[117, 118]	4/ Identification via LC−QqTOF MS and MS/MS fragmentation was performed to confirm the structure.	Level 4 ★★☆☆☆	4/ [25]
**9**	Oleoside rhamnoside (rhamnopyranosyl oleoside)	$C_{22}H_{32}O_{15}$	536.174125	InChI=1S/C22H32O15/c1-3-8-9(4-12(24)36-21-17(29)15(27)13(25)7(2)34-21)10(19(31)32)6-33-20(8)37-22-18(30)16(28)14(26)11(5-23)35-22/h3,6-7,9,11,13-18,20-23,25-30H,4-5H2,1-2H3,(H,31,32)/b8-3+	OC(C(C(C(C)O1)O)O)C1OC(CC(C(C(O)=O)=COC/2OC3C(O)C(O)C(O)C(CO)O3)C2=C/C)=O	NF	NF	NF	Fruits and seeds	[33]	4/ Identification relied solely on LC-ESI-MS/MS and the validation via fragmentation pattern comparison with literature only.	Level 4 ★★☆☆☆	4/ [119]
**10**	Frameroside	$C_{27}H_{38}O_{15}$	602.221075	InChI=1S/C27H38O15/c1-4-12-14(7-18(29)39-9-15(23(33)34)13-6-5-11(2)19(13)24(35)36)16(25(37)38-3)10-40-26(12)42-27-22(32)21(31)20(30)17(8-28)41-27/h4,10-11,13-15,17,19-22,26-28,30-32H,5-9H2,1-3H3,(H,33,34)(H,35,36)/b12-4+/t11-,13+,14-,15+,17+,19+,20-,21+,22+,26-,27-/m0/s1	C/C=C/1\[C@@H](C(=CO[C@H]1O[C@H]2[C@@H]([C@@H]([C@H]([C@H](O2)CO)O)O)O)C(=O)OC)CC(=O)OC[C@H]([C@@H]3CC[C@@H]([C@H]3C(=O)O)C)C(=O)O	12047161	326594-35-8	NF	Leaves	[103]	4/ Identification via HPLC-ESI-TOF-MS and formula matched to literature.	Level 4 ★★☆☆☆	4/ [103]
**11**	Secologanin	$C_{17}H_{24}O_{10}$	388.13695	InChI=1S/C17H24O10/c1-3-8-9(4-5-18)10(15(23)24-2)7-25-16(8)27-17-14(22)13(21)12(20)11(6-19)26-17/h3,5,7-9,11-14,16-17,19-22H,1,4,6H2,2H3/t8-,9+,11-,12-,13+,14-,16+,17+/m1/s1	COC(=O)C1=COC(C(C1CC=O)C=C)OC2C(C(C(C(O2)CO)O)O)O	161276	19351-63-4	CHEMBL1235867	Fruits, seeds, and pomace	[25, 33]	4/ Identification relied on LC-QqTOF MS and MS/MS fragmentation for structural confirmation. MS/MS fragmentation patterns were compared with accurate masses to databases (METLIN, PlantCyc) and literature.	Level 4 ★★☆☆☆	4/ [25]

No.	Compound name	Molecular formula	Exact mass	InChI	SMILES	CID	CAS Number	ChEMBL ID	Part used	References related to the part used	Identification Confidence*	Level 1 ★★★★★<br>Level 2 ★★★★☆<br>Level 3 ★★★☆☆<br>Level 4 ★★☆☆☆<br>Level 5 ★☆☆☆☆	References related to confidence level
**12**	Secoxyloganin	$C_{17}H_{24}O_{11}$	404.131865	InChI=1S/C17H24O11/c1-3-7-8(4-11(19)20)9(15(24)25-2)6-26-16(7)28-17-14(23)13(22)12(21)10(5-18)27-17/h3,6-8,10,12-14,16-18,21-23H,1,4-5H2,2H3,(H,19,20)/t7-,8+,10-,12-,13+,14-,16+,17+/m1/s1	COC(=O)C1=COC(C(C1CC(=O)O)C=C)OC2C(C(C(C(O2)CO)O)O)O	162868	58822-47-2	NF	Leaves and fruits	8, 23	2/ Analyzed via RP-HPLC-PAD-MS[70] was identified by coelution with a pure reference standard, matching Rt, UV spectra, and mass spectrometry data (pseudomolecular/ fragment ions).	Level 2 ★★★★☆	2/ [23]
**13**	Secologanoside	$C_{16}H_{22}O_{11}$	390.116215	InChI=1S/C16H22O11/c1-2-6-7(3-10(18)19)8(14(23)24)5-25-15(6)27-16-13(22)12(21)11(20)9(4-17)26-16/h2,5-7,9,11-13,15-17,20-22H,1,3-4H2,(H,18,19)(H,23,24)/t6-,7+,9-,11-,12+,13-,15+,16+/m1/s1	C=CC1C(C(=COC1OC2C(C(C(C(O2)CO)O)O)O)C(=O)O)CC(=O)O	14136854	59472-23-0	NF	Roots, flowers, fruits, seeds, pomace, leaves, stems, wood, bark, OOMW, and OLS	1, 2, 9, 96 25, 33, 34	3/ The Identification via chromatographic and spectroscopic data compared to literature.	Level 3 ★★★☆☆	3/ [115]
**14**	ρ-Coumaroyl-6`-secologanoside (comselogoside)	$C_{25}H_{28}O_{13}$	536.152995	InChI=1S/C25H28O13/c1-2-14-15(9-18(27)28)16(23(33)34)10-36-24(14)38-25-22(32)21(31)20(30)17(37-25)11-35-19(29)8-5-12-3-6-13(26)7-4-12/h2-8,10,14-15,17,20-22,24-26,30-32H,1,9,11H2,(H,27,28)(H,33,34)/b8-5+	OC(C=C1)=CC=C1/C=C/C(OCC2C(O)C(O)C(O)C(OC3OC=C(C(O)=O)C(CC(O)=O)C3C=C)O2)=O	NF	NF	NF	Leaves, flowers, fruit, pomace, roots, and OOMW	9, 33, 40, 77, 118, 120	1/ Isolated by preparative HPLC, identified by 2D NMR (COSY, HMBC, HSQC, NOESY), MS (ESI-MS, HRMS), and confirmed by co-chromatography with standards (HPLC-UV)<br><br>3/ Isolation via PHPLC; structural confirmation via 1D (1H and 13C) and 2D NMR (COSY, TOCSY, HMBC) and ESI-MS; matched to literature (no co-injection with standard).	Level 1 ★★★★★<br><br>Level 3 ★★★☆☆	1/ [115]<br><br>3/ [120]
**15**	caffeoyl-6`-secologanoside (cafselogoside)	$C_{25}H_{28}O_{14}$	552.14791	InChI=1S/C25H28O14/c1-2-12-13(8-18(28)29)14(23(34)35)9-37-24(12)39-25-22(33)21(32)20(31)17(38-25)10-36-19(30)6-4-11-3-5-15(26)16(27)7-11/h2-7,9,12-13,17,20-22,24-27,31-33H,1,8,10H2,(H,28,29)(H,34,35)/t12-,13+,17-,20-,21+,22-,24+,25+/m1/s1	C=CC1C(C(C(O)=O)=COC1OC2C(O)C(O)C(O)C(O2)COC(/C=C/C3=CC(O)=C(O)C=C3)=O)CC(O)=O	NF	NF	NF	Leaves, fruits, seeds, pomace, and OOMW	33, 118, 121	2/ Identified via LC-MS/MS analysis, with detailed spectral data and characteristic fragments (*m/z* 507, 489, 393, 323, 161). These fragments align with known secoiridoid breakdown patterns.<br><br>2/ Tentatively identified by HPLC-DAD-FLDMS/MS and comparing its Rt, UV spectra, and MS/MS fragmentation to published data.	Level 2 ★★★★☆	2/ [122]<br><br>2/ [123]

No.	Compound name	Molecular formula	Exact mass	InChI	SMILES	CID	CAS Number	ChEMBL ID	Part used	References related to the part used	Identification Confidence*	Level 1 ★★★★★<br>Level 2 ★★★★☆<br>Level 3 ★★★☆☆<br>Level 4 ★★☆☆☆<br>Level 5 ★☆☆☆☆	References related to confidence level
**16**	6ʽ*O*-[(2E)-2,6-dimethyl-8-hydroxy-2-octenoyloxy]-secologanoside / Dimethyl hydroxy octenoyloxy secologanoside	$C_{26}H_{38}O_{13}$	558.231245	InChI=1S/C26H38O13/c1-4-15-16(10-19(28)29)17(23(33)34)11-37-25(15)39-26-22(32)21(31)20(30)18(38-26)12-36-24(35)14(3)7-5-6-13(2)8-9-27/h4,7,11,13,15-16,18,20-22,25-27,30-32H,1,5-6,8-10,12H2,2-3H3,(H,28,29)(H,33,34)/b14-7+	C=CC1C(CC(O)=O)C(C(O)=O)=COC1OC2C(O)C(O)C(O)C(COC(/C(C)=C/CCC(C)CCO)=O)O2	NF	NF	NF	Roots, leaves, wood, and OLS	[1, 2, 17]	1/ Isolated by preparative HPLC, identified by 2D NMR (COSY, HMBC, HSQC, NOESY), MS (ESI-MS, HRMS), and confirmed by co-chromatography with standards (HPLC-UV).	Level 1 ★★★★★	1/ [115]
**c. Oleuropein-type secoiridoids and derivatives**													
**1**	Oleuropein aglycone (3,4-DHPEA-EA)	$C_{19}H_{22}O_{8}$	378.13147	InChI=1S/C19H22O8/c1-3-12-13(14(18(23)25-2)10-27-19(12)24)9-17(22)26-7-6-11-4-5-15(20)16(21)8-11/h3-5,8,10,13,19-21,24H,6-7,9H2,1-2H3/b12-3-/t13-,19+/m0/s1	CC=C1C(C(=COC1O)C(=O)OC)CC(=O)OCCC2=CC(=C(C=C2)O)O	124202093	NF	NF	Fruits, seeds, pomace, olive oil (VOO& EVOO), leaves, stems, wood, and OOMW	[2, 25, 38-40, 57, 63]	1/ For separation and detection HPLC-DAD-SPE-NMR/MS, for structure elucidation 1D (1H, 13C) and 2D (COSY, HSQC, HMBC, NOESY) NMR, for validation, Monte Carlo conformational search.	Level 1 ★★★★★	1/ [45]
**2**	Oleuropein	$C_{25}H_{32}O_{13}$	540.184295	InChI=1S/C25H32O13/c1-3-13-14(9-19(29)35-7-6-12-4-5-16(27)17(28)8-12)15(23(33)34-2)11-36-24(13)38-25-22(32)21(31)20(30)18(10-26)37-25/h3-5,8,11,14,18,20-22,24-28,30-32H,6-7,9-10H2,1-2H3/b13-3+/t14-,18+,20+,21-,22+,24-,25-/m0/s1	CC=C1C(C(=COC1OC2C(C(C(C(O2)CO)O)O)O)C(=O)OC)CC(=O)OCCC3=CC(=C(C=C3)O)O	5281544	32619-42-4	CHEMBL1911053	Roots, flowers, fruits, pomace, small branches, leaves, stems, olive oil (VOO& EVOO), seeds, wood, bark, OOMW, and OLS	[1, 2, 9, 26, 27, 30, 34, 56, 74, 83, 94, 96]	1/ Isolated by different chromatographic techniques (column chromatography, TLC, HPLC, PHPLC), elucidated through 1D NMR (1H and 13C NMR), 2D NMR (COSY), identified by MS and electrospray mass spectrometry.<br><br>3/ chromatographic and spectroscopic data compared to literature.	Level 1 ★★★★★<br><br>Level 3 ★★★☆☆	1/ [105]<br><br>3/ [115]
**3**	Oleuropein derivative	$C_{25}H_{36}O_{12}$	528.22068	—	—	NF	NF	NF	Pomace	[25]	4/ Identification relied on LC-QqTOF MS and MS/MS fragmentation for structural confirmation. MS/MS fragmentation patterns showed ions common to those provided by oleuropein, but lacked direct evidence (e.g., standards, NMR) to confirm the modified structure.	Level 4 ★★☆☆☆	4/ [25]

No.	Compound name	Molecular formula	Exact mass	InChI	SMILES	CID	CAS Number	ChEMBL ID	Part used	References related to the part used	Identification Confidence*	Level 1 ★★★★★<br>Level 2 ★★★★☆<br>Level 3 ★★★☆☆<br>Level 4 ★★☆☆☆<br>Level 5 ★☆☆☆☆	References related to confidence level
**4**	Oleuropein derivative	$C_{25}H_{36}O_{13}$	544.215595	—	—	NF	NF	NF	Pomace and leaves	[17, 25]	4/ Identification relied on LC-QqTOF MS and MS/MS fragmentation for structural confirmation. MS/MS fragmentation patterns showed ions common to those provided by oleuropein, but lacked direct evidence (e.g., standards, NMR) to confirm the modified structure.	Level 4 ★★☆☆☆	4/ [25]
**5**	Oleuropein derivative	$C_{35}H_{46}O_{15}$	706.283675	—	—	NF	NF	NF	Roots	[1]	4/ Identification via LC-ESI-QTOF-MS/MS and based on fragmentation pattern matched to literature.	Level 4 ★★☆☆☆	4/ [1]
**6**	Oleuropein derivative	$C_{30}H_{38}O_{18}$	686.20582	—	—	NF	NF	NF	Leaves	[124]	4/ HPLC/ESI-TOF-MS and HPLC/ESI-IT-MS² techniques were used for identification. It is based on Rt, and MS/MS fragments were matched to literature data.	Level 4 ★★☆☆☆	4/ [124]
**7**	Oleuropein 6"-*O*-β-D-glucoside (oleuricine A)	$C_{31}H_{42}O_{18}$	702.23712	InChI=1S/C31H42O18/c1-3-14-15(9-21(36)44-7-6-13-4-5-17(34)18(35)8-13)16(28(42)43-2)12-45-29(14)49-31-26(41)27(23(38)20(11-33)47-31)48-30-25(40)24(39)22(37)19(10-32)46-30/h3-5,8,12,15,19-20,22-27,29-35,37-41H,6-7,9-11H2,1-2H3/b14-3+/t15-,19+,20+,22+,23+,24-,25+,26+,27-,29-,30-,31-/m0/s1	CC=C1C(C(=COC1OC2C(C(C(C(O2)CO)O)OC3C(C(C(C(O3)CO)O)O)O)O)C(=O)OC)CC(=O)OCCC4=CC(=C(C=C4)O)O	NF	NF	NF	Roots, branches, fruit, pomace, wood, bark, OOMW, and OLS	[1, 2, 34, 40, 77, 125]	1/ Isolation by column chromatography (silica gel, C18RP), identified throgh 1D, 2D (HMBC, HSQC) NMR, and HR-ESI-MS, IR, UV techniques, and confirmed by acid hydrolysis and sugar analysis.	Level 1 ★★★★★	1/ [126]
**8**	Demethyloleuropein	$C_{24}H_{30}O_{13}$	526.168645	InChI=1S/C24H30O13/c1-2-12-13(8-18(28)34-6-5-11-3-4-15(26)16(27)7-11)14(22(32)33)10-35-23(12)37-24-21(31)20(30)19(29)17(9-25)36-24/h2-4,7,10,13,17,19-21,23-27,29-31H,5-6,8-9H2,1H3,(H,32,33)/b12-2+/t13-,17+,19+,20-,21+,23-,24-/m0/s1	CC=C1C(C(=COC1OC2C(C(C(C(O2)CO)O)O)O)C(=O)O)CC(=O)OCCC3=CC(=C(C=C3)O)O	6450302	52077-55-1	NF	Roots, flowers, fruits, seeds, pomace, leaves, stems, wood, OOMW, and OLS	[1, 2, 9, 33, 40, 58, 74]	1/ Isolation by preparative HPLC, identified by 2D NMR (COSY, HMBC, HSQC, NOESY), MS (ESI-MS, HRMS), and confirmed by co-chromatography with standards (HPLC-UV).	Level 1 ★★★★★	1/ [115]
**9**	Methyl oleuropein aglycone	$C_{20}H_{24}O_8$	392.14712	InChI=1S/C20H24O8/c1-4-13-14(15(19(23)26-3)11-28-20(13)24)10-18(22)27-8-7-12-5-6-16(21)17(9-12)25-2/h4-6,9,11,14,20-21,24H,7-8,10H2,1-3H3/b13-4-	COC(C1=COC(O)/C(C1CC(OCCC2=CC(OC)=C(O)C=C2)=O)=C\C)=O	NF	NF	NF	Roots, leaves, and olive oil (EVOO)	[1, 110]	3/ Identification via HPLC-DAD and LC-ESI-QTOF-MS/MS, relies on chromatographic alignment (Rt) and spectrometric data (HRMS/MSMS) compared to literature data.	Level 3 ★★★☆☆	3/ [1]

No.	Compound name	Molecular formula	Exact mass	InChI	SMILES	CID	CAS Number	ChEMBL ID	Part used	References related to the part used	Identification Confidence*	Level 1 ★★★★★<br>Level 2 ★★★★☆<br>Level 3 ★★★☆☆<br>Level 4 ★★☆☆☆<br>Level 5 ★☆☆☆☆	References related to confidence level
**10**	Dimethyl oleuropein aglycone	$C_{21}H_{26}O_8$	406.16277	InChI=1S/C21H26O8/c1-5-14-15(16(20(23)27-4)12-29-21(14)24)11-19(22)28-9-8-13-6-7-17(25-2)18(10-13)26-3/h5-7,10,12,15,21,24H,8-9,11H2,1-4H3/b14-5-	C/C=C1C(CC(OCCC2=CC(OC)=C(OC)C=C2)=O)C(C(OC)=O)=COC/1O	NF	NF	NF	Fruit, wood, and leaves	85	4/ Identification via LC-MS (ESI/APCI, negative/positive polarity) and reliance on literature-reported MS data without experimental spectral validation.	Level 4 ★★☆☆☆	4/ [85]
**11**	Lucidumoside C	$C_{27}H_{36}O_{14}$	584.21051	InChI=1S/C27H36O14/c1-4-14-15(9-21(31)38-12-20(37-5-2)13-6-7-17(29)18(30)8-13)16(25(35)36-3)11-39-26(14)41-27-24(34)23(33)22(32)19(10-28)40-27/h4,6-8,11,15,19-20,22-24,26-30,32-34H,5,9-10,12H2,1-3H3/b14-4+/t15-,19+,20?,22+,23-,24+,26-,27-/m0/s1	CCOC(COC(=O)CC1C(=COC(C1=CC)OC2C(C(C(C(O2)CO)O)O)O)C(=O)OC)C3=CC(=C(C=C3)O)O	10793430	354553-73-4	NF	Roots, leaves, stems, fruits, and wood	1, 2, 73, 96, 127	3/ Identification via HPLC-DAD and LC-ESI-QTOF-MS/MS, relies on chromatographic alignment (Rt) and spectrometric data (HRMS/MSMS) compared to literature data.<br>3/ Identification relied on mass spectrometry and MS/MS fragments using RP-HPLC-DAD-ESI-QTOF-MS/MS and UV/HRMS/MSMS, with literature validation for Rt and spectral data.	Level 3 ★★★☆☆	3/ [1]<br>3/ [2]
**12**	Lucidumoside D (dimethyl oleuropein)	$C_{27}H_{36}O_{13}$	568.215595	InChI=1S/C27H36O13/c1-5-15-16(11-21(29)37-9-8-14-6-7-18(34-2)19(10-14)35-3)17(25(33)36-4)13-38-26(15)40-27-24(32)23(31)22(30)20(12-28)39-27/h5-7,10,13,16,20,22-24,26-28,30-32H,8-9,11-12H2,1-4H3/b15-5+/t16-,20+,22+,23-,24+,26-,27-/m0/s1	CC=C1C(C(=COC1OC2C(C(C(C(O2)CO)O)O)O)C(=O)OC)CC(=O)OCCC3=CC(=C(C=C3)OC)OC	10531060	NF	CHEMBL4167894	Leaves and stems	40, 60	4/ Identification via UHPLC-ESI-HRMS/MS relies on chromatographic RT, HRMS/MS, adduct ions, and formula (no literature match).	Level 4 ★★☆☆☆	4/ [40]
**13**	10-Hydroxy-oleuropein aglycone	$C_{19}H_{22}O_9$	394.126385	InChI=1S/C19H22O9/c1-26-18(24)14-10-28-19(25)12(4-6-20)13(14)9-17(23)27-7-5-11-2-3-15(21)16(22)8-11/h2-4,8,10,13,19-22,25H,5-7,9H2,1H3/b12-4-	OC1OC=C(C(OC)=O)C(CC(OCCC2=CC(O)=C(O)C=C2)=O)/C1=C/CO	NF	NF	NF	Roots, fruits, pomace, leaves, and olive oil (EVOO)	1, 25, 33, 40, 76	2/ Analysis by HPLC-DAD-SPE-NMR/MS with Rt matching (42.3 and 44.6 min), structural validation via 1D (1H and 13C) and 2D NMR (COSY, HSQC, HMBC), the data (MS, Rt) aligned with literature.	Level 2 ★★★★☆	2/ [45]
**14**	10-Hydroxy-oleuropein	$C_{25}H_{32}O_{14}$	556.17921	InChI=1S/C25H32O14/c1-35-23(34)15-11-37-24(39-25-22(33)21(32)20(31)18(10-27)38-25)13(4-6-26)14(15)9-19(30)36-7-5-12-2-3-16(28)17(29)8-12/h2-4,8,11,14,18,20-22,24-29,31-33H,5-7,9-10H2,1H3/b13-4+/t14-,18+,20+,21-,22+,24-,25-/m0/s1	COC(=O)C1=COC(C(=CCO)C1CC(=O)OCCC2=CC(=C(C=C2)O)O)OC3C(C(C(C(O3)CO)O)O)O	6440747	84638-44-8	NF	Roots, leaves, stems, fruits, pomace, flowers, wood, bark, and OLS	1, 2, 9, 40, 127<br>34, 128	4/ Identification relied on mass spectrometry, MS/MS fragmentation using UPLC-Q-TOF/MS, and Literature comparison for spectral data.<br>3/ Identification via HPLC-DAD and LC-ESI-QTOF-MS/MS, relies on chromatographic alignment (Rt) and spectrometric data (HRMS/MSMS) compared to prior works.	Level 4 ★★☆☆☆<br>Level 3 ★★★☆☆	4/ [72]<br>3/ [1]

No.	Compound name	Molecular formula	Exact mass	InChI	SMILES	CID	CAS Number	ChEMBL ID	Part used	References related to the part used	Identification Confidence*	Level 1 ★★★★★<br>Level 2 ★★★★☆<br>Level 3 ★★★☆☆<br>Level 4 ★★☆☆☆<br>Level 5 ★☆☆☆☆	References related to confidence level
**15**	Methoxyoleuropein	$C_{26}H_{34}O_{14}$	570.19486	InChI=1S/C26H34O14/c1-4-13-14(8-20(30)37-11-19(35-2)12-5-6-16(28)17(29)7-12)15(24(34)36-3)10-38-25(13)40-26-23(33)22(32)21(31)18(9-27)39-26/h4-7,10,14,18-19,21-23,25-29,31-33H,8-9,11H2,1-3H3/b13-4-	CC=C1C(C(=COC1OC2C(C(C(C(O2)CO)O)O)O)C(=O)OC)CC(=O)OCC(C3=CC(=C(C=C3)O)O)OC	131751756	256498-10-9	NF	Roots, flowers, fruits, leaves, stems, wood, and bark	1, 2, 9, 34, 40, 127	3/ Analyzed using HPLC-ESI-TOF/MS and HPLC-ESI/MS/MS analysis, and the identification was based solely on literature data for fragmentation patterns.<br><br>3/ Identification relied on mass spectrometry and MS/MS fragments using RP-HPLC-DAD-ESI-QTOF-MS/MS and UV/HRMS/MSMS, with literature validation for Rt and spectral data.	Level 3 ★★★☆☆	3/ [34]<br><br>3/ [2]
**16**	Oleuropeinic acid	$C_{25}H_{30}O_{15}$	570.158475	InChI=1S/C25H30O15/c1-36-23(35)14-10-38-24(40-25-22(34)21(33)20(32)17(9-26)39-25)13(7-18(29)30)12(14)8-19(31)37-5-4-11-2-3-15(27)16(28)6-11/h2-3,6-7,10,12,17,20-22,24-28,32-34H,4-5,8-9H2,1H3,(H,29,30)/b13-7+/t12?,17-,20-,21+,22-,24?,25+/m1/s1	COC(=O)C1=COC(C(=CC(=O)O)C1CC(=O)OCCC2=CC(=C(C=C2)O)O)OC3C(C(C(C(O3)CO)O)O)O	146014677	NF	NF	Leaves and OOMW	73, 129	3/ Identification relies on Chromatographic behavior (Rt), UV spectrum, and MS/MS fragmentation pattern using LC-ESI-QTOF-MS, with Literature comparison.	Level 3 ★★★☆☆	3/ [73]
**17**	Hydroxy-*o*-decarboxymethyl oleuropein aglycone	$C_{17}H_{20}O_7$	336.120905	InChI=1S/C17H20O7/c18-6-3-13-12(5-8-24-17(13)22)10-16(21)23-7-4-11-1-2-14(19)15(20)9-11/h1-3,5,8-9,12,17-20,22H,4,6-7,10H2/b13-3-	OC1OC=CC(CC(OCCC2=CC(O)=C(O)C=C2)=O)/C1=C/CO	NF	NF	NF	Roots, fruits, and olive oil (EVOO)	40, 57, 76	2/ Analysis by HPLC-DAD-SPE-NMR/MS with Rt matching (23.2 and 33.4 min), structural validation via 1D (1H and 13C) and 2D NMR (COSY, HSQC, HMBC), the data (MS, Rt) aligned with literature.	Level 2 ★★★★☆	2/ [45]
**18**	10-Hydroxy-10-methyl oleuropein aglycone	$C_{20}H_{24}O_9$	408.142035	InChI=1S/C20H24O9/c1-11(21)7-14-13(15(19(25)27-2)10-29-20(14)26)9-18(24)28-6-5-12-3-4-16(22)17(23)8-12/h3-4,7-8,10-11,13,20-23,26H,5-6,9H2,1-2H3/b14-7-	COC(C1=COC(O)/C(C1CC(OCCC2=CC(O)=C(O)C=C2)=O)=C\C(O)C)=O	NF	NF	NF	Olive oil (EVOO)	33	3/ Identification relies on Chromatographic Rt and MS/MS fragmentation pattern using UHPLC-QTOF-MS, with Literature comparison.	Level 3 ★★★☆☆	3/ [130]
**19**	Oleuroside	$C_{25}H_{32}O_{13}$	540.184295	InChI=1S/C25H32O13/c1-3-13-14(9-19(29)35-7-6-12-4-5-16(27)17(28)8-12)15(23(33)34-2)11-36-24(13)38-25-22(32)21(31)20(30)18(10-26)37-25/h3-5,8,11,13-14,18,20-22,24-28,30-32H,1,6-7,9-10H2,2H3	COC(=O)C1=COC(C(C1CC(=O)OCCC2=CC(=C(C=C2)O)O)C=C)OC3C(C(C(C(O3)CO)O)O)O	14136851	116383-31-4	NF	Leaves, fruit, stems, roots, and bark	8, 34, 40	3/ Identification based on chromatography RPLC–DAD–ESI-MS, UV, and MS data compared to literature, without a reference standard or structural validation.	Level 3 ★★★☆☆	3/ [32]

No.	Compound name	Molecular formula	Exact mass	InChI	SMILES	CID	CAS Number	ChEMBL ID	Part used	References related to the part used	Identification Confidence*	Level 1 ★★★★★<br>Level 2 ★★★★☆<br>Level 3 ★★★☆☆<br>Level 4 ★★☆☆☆<br>Level 5 ★☆☆☆☆	References related to confidence level
**20**	Oleuricine B (oleuroside 6'-*O*-β-D-glucoside)	$C_{31}H_{42}O_{18}$	702.23712	InChI=1S/C31H42O18/c1-3-14-15(16(28(42)43-2)12-45-29(14)49-31-27(41)25(39)23(37)20(11-33)48-31)9-21(35)44-7-6-13-4-5-18(17(34)8-13)46-30-26(40)24(38)22(36)19(10-32)47-30/h3-5,8,12,14-15,19-20,22-27,29-34,36-41H,1,6-7,9-11H2,2H3	COC(C1=COC(OC2C(O)C(O)C(O)C(CO)O2)C(C=C)C1CC(OCCC3=CC(O)=C(OC4C(O)C(O)C(O)C(CO)O4)C=C3)=O)=O	NF	NF	NF	Leaves	[111]	1/ Isolation by column chromatography (silica gel, C18RP), identified throgh 1D, 2D (HMBC, HSQC) NMR, and HR-ESI-MS, IR, UV techniques, and confirmed by acid hydrolysis and sugar analysis.	Level 1 ★★★★★	1/ [126]
**21**	Hydroxyoleuroside	$C_{25}H_{32}O_{14}$	556.17921	InChI=1S/C25H32O14/c1-35-23(34)15-11-37-24(39-25-22(33)21(32)20(31)18(10-27)38-25)13(4-6-26)14(15)9-19(30)36-7-5-12-2-3-16(28)17(29)8-12/h2-4,6,8,11,13-14,18,20-22,24-29,31-33H,5,7,9-10H2,1H3/b6-4+	COC(C1=COC(OC2C(O)C(O)C(O)C(CO)O2)C(/C=C/O)C1CC(OCCC3=CC(O)=C(O)C=C3)=O)=O	NF	NF	NF	Stems, leaves, and roots	[40, 62]	4/ Identification via UHPLC-HRMS/MS and reliance on Rt, Elemental composition, and fragmentation patterns, without spectral matching to a known reference.	Level 4 ★★☆☆☆	4/ [40]
**22**	Methoxy oleuroside	$C_{26}H_{34}O_{14}$	570.19486	InChI=1S/C26H34O14/c1-4-13-14(8-20(30)37-11-19(35-2)12-5-6-16(28)17(29)7-12)15(24(34)36-3)10-38-25(13)40-26-23(33)22(32)21(31)18(9-27)39-26/h4-7,10,13-14,18-19,21-23,25-29,31-33H,1,8-9,11H2,2-3H3	COC(C1=COC(OC2C(O)C(O)C(O)C(CO)O2)C(C=C)C1CC(OCC(OC)C3=CC(O)=C(O)C=C3)=O)=O	NF	NF	NF	Leaves, stems, and roots	[40]	4/ Identification via UHPLC-HRMS/MS and reliance on Rt, Elemental composition, and fragmentation patterns, without spectral matching to a known reference.	Level 4 ★★☆☆☆	4/ [40]
**23**	Dehydro-oleuropein aglycone	$C_{19}H_{20}O_8$	376.11582	InChI=1S/C19H20O8/c1-3-12-13(14(18(23)25-2)10-27-19(12)24)9-17(22)26-7-6-11-4-5-15(20)16(21)8-11/h3-5,8,10,13,20-21H,6-7,9H2,1-2H3/b12-3-	C/C=C1C(CC(OCCC2=CC(O)=C(O)C=C2)=O)C(C(OC)=O)=COC/1=O	NF	NF	NF	Leaves, wood, fruit, and olive oil (EVOO)	[85, 110, 127]	4/ Identification via LC-MS (ESI/APCI, negative/positive polarity) and reliance on literature-reported MS data without experimental spectral validation.	Level 4 ★★☆☆☆	4/ [85]
**24**	Hydro-oleuropein	$C_{25}H_{34}O_{13}$	542.199945	InChI=1S/C25H34O13/c1-3-13-14(9-19(29)35-7-6-12-4-5-16(27)17(28)8-12)15(23(33)34-2)11-36-24(13)38-25-22(32)21(31)20(30)18(10-26)37-25/h4-5,8,11,13-14,18,20-22,24-28,30-32H,3,6-7,9-10H2,1-2H3	COC(C1=COC(OC2C(O)C(O)C(O)C(CO)O2)C(CC)C1CC(OCCC3=CC(O)=C(O)C=C3)=O)=O	NF	NF	NF	Fruits, leaves, and olive oil	[62, 73]	3/ Identification relies on Chromatographic behavior (Rt), UV spectrum, and MS/MS fragmentation pattern using LC-ESI-QTOF-MS, with Literature comparison.	Level 3 ★★★☆☆	3/ [73]
**25**	Dihydro-oleuropein	$C_{25}H_{36}O_{13}$	544.215595	InChI=1S/C25H36O13/c1-3-13-14(9-19(29)35-7-6-12-4-5-16(27)17(28)8-12)15(23(33)34-2)11-36-24(13)38-25-22(32)21(31)20(30)18(10-26)37-25/h4-5,8,13-15,18,20-22,24-28,30-32H,3,6-7,9-11H2,1-2H3	COC(C1COC(OC2C(O)C(O)C(O)C(CO)O2)C(CC)C1CC(OCCC3=CC(O)=C(O)C=C3)=O)=O	NF	NF	NF	Roots, fruit, wood, OLS, and OOMW	[1, 2, 40, 66, 85]	4/ Identification relied on mass spectrometry, MS/MS fragmentation using UPLC-Q-TOF/MS, and Literature comparison for spectral data.	Level 4 ★★☆☆☆	4/ [72]

No.	Compound name	Molecular formula	Exact mass	InChI	SMILES	CID	CAS Number	ChEMBL ID	Part used	References related to the part used	Identification Confidence*	Level 1 ★★★★★<br>Level 2 ★★★★☆<br>Level 3 ★★★☆☆<br>Level 4 ★★☆☆☆<br>Level 5 ★☆☆☆☆	References related to confidence level
**26**	Oleuropein-*O*-deoxyhexoside	$C_{31}H_{42}O_{17}$	686.242205	InChI=1S/C31H42O17/c1-4-15-16(10-21(34)43-8-7-14-5-6-18(32)19(33)9-14)17(28(41)42-3)11-44-29(15)48-31-27(40)25(38)23(36)20(47-31)12-45-30-26(39)24(37)22(35)13(2)46-30/h4-6,9,11,13,16,20,22-27,29-33,35-40H,7-8,10,12H2,1-3H3/b15-4-	CC1C(O)C(O)C(O)C(OCC(O2)C(O)C(O)C(O)C2OC3OC=C(C(OC)=O)C(CC(OCCC4=CC(O)=C(O)C=C4)=O)/C3=C/C)O1	NF	NF	NF	Leaves	[73]	3/ Identification relies on Chromatographic behavior (Rt), UV spectrum, and MS/MS fragmentation pattern using LC-ESI-QTOF-MS, with Literature comparison.	Level 3 ★★★☆☆	3/ [73]
**27**	Oleuropein dimer	$C_{50}H_{62}O_{25}$	1062.358025	InChI=1S/C50H62O25/c1-5-25-27(29(45(63)65-3)21-69-47(25)74-49-43(61)41(59)39(57)35(19-51)72-49)17-37(55)67-13-11-23-8-10-33(32(54)15-23)71-34-16-24(7-9-31(34)53)12-14-68-38(56)18-28-26(6-2)48(70-22-30(28)46(64)66-4)75-50-44(62)42(60)40(58)36(20-52)73-50/h5-10,15-16,21-22,27-28,35-36,39-44,47-54,57-62H,11-14,17-20H2,1-4H3/b25-5-,26-6-	C/C=C1C(CC(OCCC2=CC(O)=C(OC3=C(O)C=CC(CCOC(CC(C(C(OC)=O)=COC/4OC5C(O)C(O)C(O)C(CO)O5)C4=C\C)=O)=C3)C=C2)=O)C(C(OC)=O)=COC/1OC6C(O)C(O)C(O)C(CO)O6	NF	NF	NF	Fruit and pomace	[131]	3/ Identified via ESI-MS/MS + NMR (indirect) based on MS/MS fragmentation logic (neutral losses matching oleuropein mass), general NMR evidence for oligomers, and literature comparison.	Level 3 ★★★☆☆	3/ [131]
**28**	Deacetoxy-oleuropein aglycone	$C_{17}H_{20}O_6$	320.12599	InChI=1S/C17H20O6/c1-2-13(11-19)14(5-7-18)10-17(22)23-8-6-12-3-4-15(20)16(21)9-12/h2-4,7,9,11,14,20-21H,5-6,8,10H2,1H3/b13-2-	C/C=C(C(CC=O)CC(OCCC1=CC=C(C(O)=C1)O)=O)/C=O	NF	NF	NF	Olive oil (EVOO)	[132, 133]	2/ Identification via APCI-MS, compared to fragmentation patterns with prior literature.	Level 2 ★★★★☆	2/ [133]
**29**	Jaspolyoside	$C_{42}H_{54}O_{23}$	926.305595	InChI=1S/C42H54O23/c1-5-19-21(12-29(46)58-10-9-18-7-8-25(44)26(45)11-18)23(37(54)56-3)15-61-40(19)65-42-36(53)34(51)32(49)28(63-42)17-59-30(47)13-22-20(6-2)39(60-16-24(22)38(55)57-4)64-41-35(52)33(50)31(48)27(14-43)62-41/h5-8,11,15-16,21-22,27-28,31-36,39-45,48-53H,9-10,12-14,17H2,1-4H3/b19-5-,20-6-/t21-,22+,27+,28+,31+,32+,33-,34-,35+,36+,39+,40-,41-,42-/m1/s1	C/C=C1[C@@H](OC=C([C@H]/1CC(OC[C@H]2O[C@H]([C@@H]([C@H]([C@@H]2O)O)O)O[C@@H]3OC=C([C@H](/C3=C\C)CC(OCCC4=CC(O)=C(O)C=C4)=O)C(OC)=O)=O)C(OC)=O)O[C@@H]5O[C@@H]([C@H]([C@@H]([C@H]5O)O)O)CO	57395550	NF	CHEMBL1911052	Roots, flowers, leaves, stems, wood, and OLS	[1, 2, 9, 40, 73]	1/ Isolation via HPLC, full structural elucidation: 1D/2D NMR (including 1H, 13C, COSY, HMQC, HMBC), and HMBC correlation confirmed glucoside linkage at C-3' of ligustroside, was used.<br><br>3/ Identification via HPLC-DAD and LC-ESI-QTOF-MS/MS, relies on chromatographic alignment (Rt) and spectrometric data (HRMS/MSMS) compared to prior works.<br><br>3/ Identification relied on mass spectrometry and MS/MS fragments using RP-HPLC-DAD-ESI-QTOF-MS/MS and UV/HRMS/MSMS, with literature validation for Rt and spectral data.	Level 1 ★★★★★<br><br>Level 3 ★★★☆☆<br><br>Level 3 ★★★☆☆	1/ [134]<br><br>3/ [1]<br><br>3/ [2]

No.	Compound name	Molecular formula	Exact mass	InChI	SMILES	CID	CAS Number	ChEMBL ID	Part used	References related to the part used	Identification Confidence*	Level 1 ★★★★★<br>Level 2 ★★★★☆<br>Level 3 ★★★☆☆<br>Level 4 ★★☆☆☆<br>Level 5 ★☆☆☆☆	References related to confidence level
**30**	Fraxamoside	$C_{25}H_{30}O_{13}$	538.168645	InChI=1S/C25H30O13/c1-3-12-13-7-19(28)35-9-17(11-4-5-15(26)16(27)6-11)34-10-18-20(29)21(30)22(31)25(37-18)38-24(12)36-8-14(13)23(32)33-2/h3-6,8,13,17-18,20-22,24-27,29-31H,7,9-10H2,1-2H3/b12-3+/t13-,17-,18+,20+,21-,22+,24-,25-/m0/s1	C/C=C/1\[C@@H]2CC(=O)OC[C@H](OC[C@@H]3[C@H]([C@@H]([C@H]([C@@H](O3)O[C@@H]1OC=C2C(=O)OC)O)O)O)C4=CC(=C(C=C4)O)O	5323574	NF	CHEMBL4228021	Flowers, fruits, leaves, stems, roots, wood, and OLS	2, 9, 40, 127	3/ Identification relied on mass spectrometry and MS/MS fragments using RP-HPLC-DAD-ESI-QTOF-MS/MS and UV-vis data, with literature comparison.	Level 3 ★★★☆☆	3/ [2]
**31**	Lucidumoside derivative	$C_{26}H_{38}O_{12}$	542.23633	—	—	NF	NF	NF	Roots, fruits, and leaves	40, 66, 76	3/ Identification was based on chromatographic (Rt), spectroscopic (HRMS/MS/MS) data, and UV (via DAD) compared to literature.	Level 3 ★★★☆☆	3/ [76]
**d. Ligstroside-type secoiridoids**													
**1**	Ligustroside aglycone (HPE-EA/ HPEA-EA)	$C_{19}H_{22}O_7$	362.136555	InChI=1S/C19H22O7/c1-3-14-15(16(18(22)24-2)11-26-19(14)23)10-17(21)25-9-8-12-4-6-13(20)7-5-12/h3-7,11,15,19-20,23H,8-10H2,1-2H3/b14-3+/t15-,19+/m0/s1	CC=C1C(C(=COC1O)C(=O)OC)CC(=O)OCCC2=CC=C(C=C2)O	102252771	NF	NF	Roots, fruits, pomace, leaves, olive oil (VOO& EVOO), and OOMW	1, 25, 39, 57, 63, 135	2/ Analysis by LC-SPE-NMR with Rt matching (26.0 min), structural elucidation via 1D (1H and 13C) and 2D NMR (TOCSY) compared with prior literature.	Level 2 ★★★★☆	2/ [28]
**2**	Ligustroside (4-HPE-EA-glucoside)	$C_{25}H_{32}O_{12}$	524.18938	InChI=1S/C25H32O12/c1-3-15-16(10-19(28)34-9-8-13-4-6-14(27)7-5-13)17(23(32)33-2)12-35-24(15)37-25-22(31)21(30)20(29)18(11-26)36-25/h3-7,12,16,18,20-22,24-27,29-31H,8-11H2,1-2H3/b15-3+/t16-,18+,20+,21-,22+,24-,25-/m0/s1	CC=C1C(C(=COC1OC2C(C(C(C(O2)CO)O)O)O)C(=O)OC)CC(=O)OCCC3=CC=C(C=C3)O	14136859	35897-92-8	CHEMBL1086877	Roots, flowers, fruits, seeds, leaves, stems, pomace, olive oil (VOO), wood, bark, OOMW, and OLS	1, 2, 8, 9, 34, 40, 56, 58, 74	1/ Isolated by different chromatographic techniques (column chromatography, TLC, HPLC, PHPLC), elucidated through 1D NMR (1H and 13C NMR), 2D NMR (COSY), identified by MS and electrospray mass spectrometry.	Level 1 ★★★★★	1/ [105]
**3**	Demethyl ligustroside	$C_{24}H_{30}O_{12}$	510.17373	InChI=1S/C24H30O12/c1-2-14-15(9-18(27)33-8-7-12-3-5-13(26)6-4-12)16(22(31)32)11-34-23(14)36-24-21(30)20(29)19(28)17(10-25)35-24/h2-6,11,15,17,19-21,23-26,28-30H,7-10H2,1H3,(H,31,32)/b14-2-	C/C=C1C(CC(OCCC2=CC=C(O)C=C2)=O)C(C(O)=O)=COC/1OC3C(O)C(O)C(O)C(CO)O3	NF	NF	NF	Leaves, flowers, fruits, pomace, and OOMW	8, 9, 50	5/ No experimental details or analytical proof provided.	Level 5 ★☆☆☆☆	5/ [32]

No.	Compound name	Molecular formula	Exact mass	InChI	SMILES	CID	CAS Number	ChEMBL ID	Part used	References related to the part used	Identification Confidence*	Level 1 ★★★★★<br>Level 2 ★★★★☆<br>Level 3 ★★★☆☆<br>Level 4 ★★☆☆☆<br>Level 5 ★☆☆☆☆	References related to confidence level
**4**	Ligustroside-3'-*O*-β-D-glucoside	$C_{31}H_{42}O_{17}$	686.242205	InChI=1S/C31H42O17/c1-3-16-17(10-21(35)43-9-8-14-4-6-15(34)7-5-14)18(28(41)42-2)13-44-29(16)48-31-26(40)27(23(37)20(12-33)46-31)47-30-25(39)24(38)22(36)19(11-32)45-30/h3-7,13,17,19-20,22-27,29-34,36-40H,8-12H2,1-2H3/b16-3+/t17-,19+,20+,22+,23+,24-,25+,26+,27-,29-,30-,31-/m0/s1	CC=C1C(C(=COC1OC2C(C(C(C(O2)CO)O)OC3C(C(C(C(O3)CO)O)O)O)O)C(=O)OC)CC(=O)OCCC4=CC=C(C=C4)O	102031345	NF	NF	Wood	[12]	1/ Isolation via HPLC, full structural elucidation: 1D/2D NMR (including 1H, 13C, COSY, HMQC, HMBC), and HMBC correlation confirmed glucoside linkage at C-3' of ligustroside, was used.	Level 1 ★★★★★	1/ [134]
**5**	Dehydroligustroside aglycone	$C_{19}H_{20}O_{7}$	360.120905	InChI=1S/C19H20O7/c1-3-14-15(16(18(22)24-2)11-26-19(14)23)10-17(21)25-9-8-12-4-6-13(20)7-5-12/h3-7,11,15,20H,8-10H2,1-2H3/b14-3-	C/C=C1C(CC(OCCC2=CC=C(O)C=C2)=O)C(C(OC)=O)=COC/1=O	NF	NF	NF	Olive oil (VOO) and fruit	[85]	4/ Identification via LC-MS (ESI/APCI, negative/positive polarity) and reliance on literature-reported MS data without experimental spectral validation.	Level 4 ★★☆☆☆	4/ [85]
**6**	Jaspolyanoside	$C_{42}H_{54}O_{22}$	910.31068	InChI=1S/C42H54O22/c1-5-21-23(13-29(45)57-12-11-19-7-9-20(44)10-8-19)25(37(53)55-3)16-60-40(21)64-42-36(52)34(50)32(48)28(62-42)18-58-30(46)14-24-22(6-2)39(59-17-26(24)38(54)56-4)63-41-35(51)33(49)31(47)27(15-43)61-41/h5-10,16-17,23-24,27-28,31-36,39-44,47-52H,11-15,18H2,1-4H3/b21-5+,22-6+	C/C=C1[C@@H](OC=C([C@H]/1CC(OC[C@H]2O[C@H]([C@@H]([C@H]([C@@H]2O)O)O)O[C@@H]3OC=C([C@H](/C3=C\C)CC(OCCC4=CC=C(O)C=C4)=O)C(OC)=O)=O)C(OC)=O)O[C@@H]5O[C@@H]([C@H]([C@@H]([C@H]5O)O)O)CO	NF	NF	NF	Wood, seeds, leaves, stems, and roots	[24, 38, 40]	1/ Isolation via HPLC, full structural elucidation: 1D/2D NMR (including 1H, 13C, COSY, HMQC, HMBC), and HMBC correlation confirmed glucoside linkage at C-3' of ligustroside, was used.	Level 1 ★★★★★	1/ [134]
**e. Nuzhenide-type secoiridoids**													
**1**	Nuzhenide (nüzhenide)	$C_{31}H_{42}O_{17}$	686.242205	InChI=1S/C31H42O17/c1-3-16-17(18(28(41)42-2)12-45-29(16)48-31-27(40)24(37)22(35)19(11-32)46-31)10-21(34)44-13-20-23(36)25(38)26(39)30(47-20)43-9-8-14-4-6-15(33)7-5-14/h3-7,12,17,19-20,22-27,29-33,35-40H,8-11,13H2,1-2H3/b16-3+/t17?,19-,20-,22-,23-,24+,25+,26-,27-,29+,30-,31+/m1/s1	C/C=C\1/[C@@H](OC=C(C1CC(=O)OC[C@@H]2[C@H]([C@@H]([C@H]([C@@H](O2)OCCC3=CC=C(C=C3)O)O)O)O)C(=O)OC)O[C@H]4[C@@H]([C@H]([C@@H]([	6440999	39011-92-2	CHEMBL1076818	Roots, seeds, pomace, leaves, fruits, bark, olive oil (VOO), and OOMW	[1, 33, 34, 56, 111, 136]	1/ Isolated by HPLC, elucidated by 1H and 13C NMR data, and confirmed by 2D NOESY technique.	Level 1 ★★★★★	1/ [137]

No.	Compound name	Molecular formula	Exact mass	InChI	SMILES	CID	CAS Number	ChEMBL ID	Part used	References related to the part used	Identification Confidence*	Level 1 ★★★★★<br>Level 2 ★★★★☆<br>Level 3 ★★★☆☆<br>Level 4 ★★☆☆☆<br>Level 5 ★☆☆☆☆	References related to confidence level
					C@H](O4)CO)O)O)O								
**2**	Neo-nüzhenide	$C_{31}H_{42}O_{18}$	702.23712	InChI=1S/C31H42O18/c1-3-14-15(16(28(42)43-2)11-46-29(14)49-31-27(41)24(38)22(36)19(10-32)47-31)9-21(35)45-12-20-23(37)25(39)26(40)30(48-20)44-7-6-13-4-5-17(33)18(34)8-13/h3-5,8,11,15,19-20,22-27,29-34,36-41H,6-7,9-10,12H2,1-2H3/b14-3+/t15-,19+,20+,22+,23+,24-,25-,26+,27+,29-,30+,31-/m0/s1	C/C=C/1\[C@@H](C(=CO[C@H]1O[C@H]2[C@@H]([C@H]([C@@H]([C@H](O2)CO)O)O)O)C(=O)OC)CC(=O)OC[C@@H]3[C@H]([C@@H]([C@H]([C@@H](O3)OCCC4=CC(=C(C=C4)O)O)O)O)O	101720830	NF	CHEMBL4744112	Leaves, flowers, seeds, and fruits	9, 38, 40, 127	4/ Identification via LC-HRMS and reliance on accurate mass and comparison with literature data (indirect way).	Level 4 ★★☆☆☆	4/ 127
**3**	Nuzhenide oleoside (nüzhenide oleoside)	$C_{47}H_{62}O_{27}$	1058.34785	InChI=1S/C47H62O27/c1-4-21-23(25(41(61)62)16-67-43(21)73-46-39(59)35(55)32(52)27(14-48)70-46)13-31(51)69-20-8-6-19(7-9-20)10-11-65-45-38(58)37(57)34(54)29(72-45)18-66-30(50)12-24-22(5-2)44(68-17-26(24)42(63)64-3)74-47-40(60)36(56)33(53)28(15-49)71-47/h4-9,17,24,27-29,32-40,43-49,52-60H,10-16,18H2,1-3H3,(H,61,62)/b21-4-,22-5-	C/C=C1[C@H](O[C@H]2[C@H](O)[C@@H](O)[C@H](O)[C@@H](CO)O2)OC=C(C(OC)=O)C/1CC(OC[C@@H]3[C@@H](O)[C@H](O)[C@@H](O)[C@H](OCCC4=CC=C(OC(C[C@H](C(C(O)=O)=CO[C@H]/5O[C@H]6[C@H](O)[C@@H](O)[C@H](O)[C@@H](CO)O6)C5=C/C)=O)C=C4)O3)=O	NF	NF	NF	Fruits and seeds	33	3/ The techniques were used TLC, CC, IR, UV, Hydrolysis, and MW estimation. Both chromatographic and spectroscopic parameters compared with literature data.	Level 3 ★★★☆☆	3/ 138

No.	Compound name	Molecular formula	Exact mass	InChI	SMILES	CID	CAS Number	ChEMBL ID	Part used	References related to the part used	Identification Confidence*	Level 1 ★★★★★ Level 2 ★★★★☆ Level 3 ★★★☆☆ Level 4 ★★☆☆☆ Level 5 ★☆☆☆☆	References related to confidence level
**4**	Nuzhenide-11-methyl oleoside	$C_{48}H_{64}O_{27}$	1072.3635	InChI=1S/C48H64O27/c1-5-22-24(26(42(62)64-3)17-68-44(22)74-47-40(60)36(56)33(53)28(15-49)71-47)13-31(51)67-19-30-35(55)38(58)39(59)46(73-30)66-12-11-20-7-9-21(10-8-20)70-32(52)14-25-23(6-2)45(69-18-27(25)43(63)65-4)75-48-41(61)37(57)34(54)29(16-50)72-48/h5-10,17,24,28-30,33-41,44-50,53-61H,11-16,18-19H2,1-4H3/b22-5-,23-6-	C/C=C1[C@H](O[C@H]2[C@H](O)[C@@H](O)[C@H](O)[C@@H](CO)O2)OC=C(C(OC)=O)C/1CC(OC[C@@H]3[C@@H](O)[C@H](O)[C@@H](O)[C@H](OCCC4=CC=C(OC(C[C@H](C(C(OC)=O)=CO[C@H]/5O[C@H]6[C@H](O)[C@@H](O)[C@H](O)[C@@H](CO)O6)C5=C/C)=O)C=C4)O3)=O	NF	NF	NF	Fruits and seeds	[136, 139]	4/ The olive seeds were analyzed by RP-HPLC-DAD, ESI-MS/MS (negative/positive polarity), and compared to literature data only.	Level 3 ★★★☆☆	3/ [136]
**5**	Methoxynüzhenide 11-methyl oleoside	$C_{49}H_{66}O_{28}$	1102.374069	InChI=1S/C49H66O28/c1-6-22-24(26(43(63)66-4)16-69-45(22)76-48-41(61)37(57)34(54)28(14-50)73-48)12-32(52)68-19-31-36(56)39(59)40(60)47(75-31)71-18-30(65-3)20-8-10-21(11-9-20)72-33(53)13-25-23(7-2)46(70-17-27(25)44(64)67-5)77-49-42(62)38(58)35(55)29(15-51)74-49/h6-11,16,24,28-31,34-42,45-51,54-62H,12-15,17-19H2,1-5H3/b22-6-,23-7-	C/C=C1[C@H](O[C@H]2[C@H](O)[C@@H](O)[C@H](O)[C@@H](CO)O2)OC=C(C(OC)=O)C/1CC(OC[C@@H]3[C@@H](O)[C@H](O)[C@@H](O)[C@H](OCC(OC)C4=CC=C(OC(C[C@H](C(C(OC)=O)=CO[C@H]/5O[C@H]6[C@H](O)[C@@H](O)[C@H](O)[C@@H](CO)O6)C5=C/C)=O)C=C4)O3)=O	NF	NF	NF	seeds	[140]	3/ Identification via UPLC-DAD-ESI-QTOF-HRMS (Rt, UV, MS/MS) and compared to literature data only.	Level 3 ★★★☆☆	3/ [135]

f. Other secoiridoids

No.	Compound name	Molecular formula	Exact mass	InChI	SMILES	CID	CAS Number	ChEMBL ID	Part used	References related to the part used	Identification Confidence*	Level 1 ★★★★★<br>Level 2 ★★★★☆<br>Level 3 ★★★☆☆<br>Level 4 ★★☆☆☆<br>Level 5 ★☆☆☆☆	References related to confidence level
**1**	Oleocanthal (ρ-HPEA-EDA/ ρ-Hydroxy decarboxymethyl-ligustroside aglycone)	$C_{17}H_{20}O_5$	304.131075	InChI=1S/C17H20O5/c1-2-14(12-19)15(7-9-18)11-17(21)22-10-8-13-3-5-16(20)6-4-13/h2-6,9,12,15,20H,7-8,10-11H2,1H3/b14-2-/t15-/m0/s1	CC=C(C=O)C(CC=O)CC(=O)OCCC1=CC=C(C=C1)O	11652416	289030-99-5	CHEMBL2172394	Olive oil (VOO& EVOO), pomace, leaves, and OOMW	25, 110, 141	1/ 1D (1H/13C), 2D NMR (HMBC, HSQC, TOCSY), literature validation<br>2/ Analysis by LC-SPE-NMR with Rt matching (18.0 min), structural elucidation via 1D (1H and 13C) and 2D NMR (TOCSY) compared with prior literature for spectral assignment.	Level 1 ★★★★★<br>Level 2 ★★★★☆	1/ [75]<br>2/ [28]
**2**	Oleacein (hydroxy-oleocanthal/ 3,4-DHPEA-EDA)	$C_{17}H_{20}O_6$	320.12599	InChI=1S/C17H20O6/c1-2-13(11-19)14(5-7-18)10-17(22)23-8-6-12-3-4-15(20)16(21)9-12/h2-4,7,9,11,14,20-21H,5-6,8,10H2,1H3/b13-2+	CC=C(C=O)C(CC=O)CC(=O)OCCC1=CC(=C(C=C1)O)O	18684078	149183-75-5	NF	Pomace, olive oil (VOO& EVOO), leaves, fruits, OOMW, and roots	30, 40, 63, 74, 110	1/ 1D (1H/13C), 2D NMR (HMBC, HSQC, TOCSY), literature validation<br>2/ Analysis by LC-SPE-NMR with Rt matching (18.0 min), structural elucidation via 1D (1H and 13C) and 2D NMR (TOCSY) compared with prior literature for spectral assignment.	Level 1 ★★★★★<br>Level 2 ★★★★☆	1/ [75]<br>2/ [28]
**3**	Methyl oleacein (methyldecarboxymethyloleuropein aglycone)	$C_{18}H_{22}O_6$	334.14164	InChI=1S/C18H22O6/c1-3-14(12-20)15(6-8-19)11-18(22)24-9-7-13-4-5-16(21)17(10-13)23-2/h3-5,8,10,12,15,21H,6-7,9,11H2,1-2H3/b14-3+	C/C=C(C(CC(OCCC1=CC(OC)=C(O)C=C1)=O)CC=O)\C=O	NF	NF	NF	Fruit, olive oil (VOO), leaves, and wood	85	4/ Identification via LC-MS (ESI/APCI, negative/positive polarity) and reliance on literature-reported MS data without experimental spectral validation.	Level 4 ★★☆☆☆	4/ [85]
**4**	Monohydrated oleacein (geminal diol)	$C_{17}H_{22}O_7$	338.136555	InChI=1S/C17H22O7/c1-2-12(10-18)13(8-16(21)22)9-17(23)24-6-5-11-3-4-14(19)15(20)7-11/h2-4,7,10,13,16,19-22H,5-6,8-9H2,1H3/b12-2+	C/C=C(C(CC(OCCC1=CC(O)=C(O)C=C1)=O)CC(O)O)\C=O	NF	NF	NF	Leaves and OOMW	66, 111	4/ Identification via MALDI-TOF/TOF MS and MS/MS fragmentation was performed to confirm the structure.	Level 4 ★★☆☆☆	4/ [111]
**5**	Hydroxytyrosil acyclodihydroelenolate (HT-ACDE)	$C_{19}H_{26}O_8$	382.16277	InChI=1S/C19H26O8/c1-3-13(10-20)14(15(11-21)19(25)26-2)9-18(24)27-7-6-12-4-5-16(22)17(23)8-12/h3-5,8,14-15,20-23H,6-7,9-11H2,1-2H3/b13-3+	OC/C(C(CC(OCCC1=CC(O)=C(O)C=C1)=O)C(CO)C(OC)=O)=C\C	NF	NF	NF	OOMW	120	3/ Isolation via PHPLC; structural confirmation via 1D (1H and 13C) and 2D NMR (COSY, TOCSY, HMBC) and ESI-MS; matched to literature (no co-injection with standard).	Level 3 ★★★☆☆	3/ [120]
**6**	Acyclodihydroelenolic acid glucoside	$C_{17}H_{28}O_{11}$	408.163165	InChI=1S/C17H28O11/c1-3-8(9(4-12(20)21)10(5-18)16(25)26-2)7-27-17-15(24)14(23)13(22)11(6-19)28-17/h3,9-11,13-15,17-19,22-24H,4-7H2,1-2H3,(H,20,21)/b8-3-	OCC(C(OC)=O)C(/C(COC1C(O)C(O)C(O)C(CO)O1)=C\C)CC(O)=O	NF	NF	NF	Fruit, roots, wood, and OLS	1, 2, 127	1/ Full structural elucidation via 1D (1H, 13C) and 2D (COSY, HSQC, NOESY, and DEPT-45), HR-ESI-MS, and IR spectroscopy after isolating the compound using HPLC/column chromatography. Acetylation to confirm free hydroxyl groups through chemical derivatization.	Level 1 ★★★★★	1/ [100]

No.	Compound name	Molecular formula	Exact mass	InChI	SMILES	CID	CAS Number	ChEMBL ID	Part used	References related to the part used	Identification Confidence*	Level 1 ★★★★★<br>Level 2 ★★★★☆<br>Level 3 ★★★☆☆<br>Level 4 ★★☆☆☆<br>Level 5 ★☆☆☆☆	References related to confidence level
**7**	1-β-D-glucopyranosyl acyclodihydroelenol-ic acid glucoside	$C_{23}H_{38}O_{16}$	570.21599	InChI=1S/C23H38O16/c1-3-9(10(4-14(27)28)11(5-24)21(34)35-2)8-36-23-20(18(32)16(30)13(7-26)38-23)39-22-19(33)17(31)15(29)12(6-25)37-22/h3,10-13,15-20,22-26,29-33H,4-8H2,1-2H3,(H,27,28)/b9-3-	OCC(C(OC)=O)C(/C(COC1C(OC2C(O)C(O)C(O)C(CO)O2)C(O)C(O)C(CO)O1)=C\C)CC(O)=O	NF	NF	NF	Pomace	[100]	1/ Full structural elucidation via 1D (1H, 13C) and 2D (COSY, HSQC, NOESY) and HR-ESI-MS.	Level 1 ★★★★★	1/ [100]
**8**	Olenoside	$C_{11}H_{14}O_{5}$	226.084125	InChI=1S/C11H14O5/c1-6-8-4-16-10(12)3-7(8)9(5-15-6)11(13)14-2/h5-8H,3-4H2,1-2H3	O=C(OC1)CC2C1C(C)OC=C2C(OC)=O	NF	NF	NF	OOMW	[142]	1/ Isolation was achieved by liq.-liq. Extraction C18-RP, elucidated through 2D NMR (COSY, HMQC, HMBC, and NOESY), FT-IR, UV, MS (HR-ESI-MS), and confirmed by X-ray diffraction.	Level 1 ★★★★★	1/ [142]
**9**	Secologanic acid	$C_{16}H_{22}O_{10}$	374.1213	InChI=1S/C16H22O10/c1-2-6-7-3-10(18)25-14(22)8(7)5-23-15(6)26-16-13(21)12(20)11(19)9(4-17)24-16/h2,5-7,9-13,15-21H,1,3-4H2/t6-,7+,9-,10?,11-,12+,13-,15+,16+/m1/s1	C=CC1C2CC(OC(=O)C2=COC1OC3C(C(C(C(O3)CO)O)O)O)O	71607801	60077-46-5	NF	Pomace	[25]	3/ Identification via LC-HRMS, NMR (HMBC). The validation method through the fragmentation pattern of comselogoside compared to the literature.	Level 3 ★★★☆☆	3/ [120]
					**8- Isochromans**								
**1**	1-Phenyl-6,7-dihydroxyisochroman	$C_{15}H_{14}O_{3}$	242.094295	InChI=1S/C15H14O3/c16-13-8-11-6-7-18-15(12(11)9-14(13)17)10-4-2-1-3-5-10/h1-5,8-9,15-17H,6-7H2	OC1=CC2=C(C=C1O)CCOC2C3=CC=CC=C3	131750844	NF	NF	Fruits and olive oil	[143]	3/ Identification via HPLC-MS/MS (Multiple Reaction Monitoring, MRM mode) and MS/MS spectra matched to a synthesized chemical standard.	Level 3 ★★★☆☆	3/ [144]
**2**	1-(3'-Methoxy-4'-hydroxy)phenyl-6,7-dihydroxyisochroman	$C_{16}H_{16}O_{5}$	288.099775	InChI=1S/C16H16O5/c1-20-15-7-10(2-3-12(15)17)16-11-8-14(19)13(18)6-9(11)4-5-21-16/h2-3,6-8,16-19H,4-5H2,1H3	OC1=CC2=C(C=C1O)CCOC2C3=CC=C(O)C(OC)=C3	NF	NF	NF	Olive oil (VOO) and fruits	[145, 146]	3/ Identification via HPLC-MS/MS (Multiple Reaction Monitoring, MRM mode) and MS/MS spectra matched to a synthesized chemical standard.	Level 3 ★★★☆☆	3/ [144]
					**9- Lignans, glycosides, and derivatives**								
**a. Furan-type lignans**													
**1**	[147]-Olivil	$C_{20}H_{24}O_{7}$	376.152205	InChI=1S/C20H24O7/c1-25-17-7-12(3-5-15(17)22)9-20(24)11-27-19(14(20)10-21)13-4-6-16(23)18(8-13)26-2/h3-8,14,19,21-24H,9-11H2,1-2H3/t14-,19-,20-/m1/s1	COC1=C(C=CC(=C1)CC2(COC(C2CO)C3=CC(=C(C=C3)O)OC)O)O	5273570	2955-23-9	NF	Stems, leaves, roots, wood, and bark	[1, 2, 33, 40, 62]	1/ isolated then purified by column chromatography and PTLC, identified through 1D (1H and 13C) and 2D (COSY, HMBC) NMR, HREIMS, IR, UV spectrophotometer techniques, and confirmed by literature.	Level 1 ★★★★★	1/ [148]

No.	Compound name	Molecular formula	Exact mass	InChI	SMILES	CID	CAS Number	ChEMBL ID	Part used	References related to the part used	Identification Confidence*	Level 1 ★★★★★<br>Level 2 ★★★★☆<br>Level 3 ★★★☆☆<br>Level 4 ★★☆☆☆<br>Level 5 ★☆☆☆☆	References related to confidence level
**2**	[147]-Olivil 4-*O*-β-D-glucopyranoside	$C_{26}H_{34}O_{12}$	538.20503	InChI=1S/C26H34O12/c1-34-18-8-14(4-5-16(18)29)24-15(10-27)26(33,12-36-24)9-13-3-6-17(19(7-13)35-2)37-25-23(32)22(31)21(30)20(11-28)38-25/h3-8,15,20-25,27-33H,9-12H2,1-2H3/t15-,20-,21-,22+,23-,24-,25-,26-/m1/s1	COC1=C(C=CC(=C1)CC2(COC(C2CO)C3=CC(=C(C=C3)O)OC)O)OC4C(C(C(C(O4)CO)O)O)O	14033815	76880-93-8	NF	Roots, stems, wood, and OLS	[1, 24, 62]	3/ Identification via HPLC-DAD and LC-ESI-QTOF-MS/MS, relies on chromatographic alignment (Rt) and spectrometric data (HRMS/MSMS) compared to literature data.<br><br>3/ Identification relied on mass spectrometry and MS/MS fragments using RP-HPLC-DAD-ESI-QTOF-MS/MS and UV/HRMS/MSMS, with literature validation for Rt and spectral data.	Level 3 ★★★☆☆	3/ [1]<br><br>3/ [2]
**3**	Berchemol	$C_{20}H_{24}O_7$	376.152205	InChI=1S/C20H24O7/c1-25-17-8-12(3-5-15(17)22)7-14-10-27-19(20(14,24)11-21)13-4-6-16(23)18(9-13)26-2/h3-6,8-9,14,19,21-24H,7,10-11H2,1-2H3	COC1=C(C=CC(=C1)CC2COC(C2(CO)O)C3=CC(=C(C=C3)O)OC)O	14521044	NF	NF	Olive oil (EVOO)	[33]	4/ Analysis by LC-SPE-NMR (1H only). No standard/literature comparison or 2D NMR was used.	Level 4 ★★☆☆☆	4/ [28]
**b. Cyclohexane-type lignans**													
**1**	(+)-Cycloolivil	$C_{20}H_{24}O_7$	376.152205	InChI=1S/C20H24O7/c1-26-17-5-11(3-4-15(17)23)19-13-7-16(24)18(27-2)6-12(13)8-20(25,10-22)14(19)9-21/h3-7,14,19,21-25H,8-10H2,1-2H3/t14-,19+,20-/m1/s1	COC1=C(C=C2C(C(C(CC2=C1)(CO)O)CO)C3=CC(=C(C=C3)O)OC)O	5316262	3064- 05-9	CHEMBL516536	Wood, bark, and stems	[2, 33, 96]	3/ Identification relied on mass spectrometry and MS/MS fragments using RP-HPLC-DAD-ESI-QTOF-MS/MS and UV-vis data, with literature comparison.	Level 3 ★★★☆☆	3/ [2]
**2**	Cycloolivil glucoside	$C_{26}H_{34}O_{12}$	538.20503	InChI=1S/C26H34O12/c1-35-18-6-13-8-26(34,11-29)15(9-27)21(14(13)7-16(18)30)12-3-4-17(19(5-12)36-2)37-25-24(33)23(32)22(31)20(10-28)38-25/h3-7,15,20-25,27-34H,8-11H2,1-2H3	COC1=C(O)C=C2C(C3=CC(OC)=C(OC4C(O)C(O)C(O)C(CO)O4)C=C3)C(CO)C(CO)(O)CC2=C1	NF	NF	NF	Stems, roots, wood, and OLS	[2, 40, 96]	3/ Identification relied on mass spectrometry and MS/MS fragments using RP-HPLC-DAD-ESI-QTOF-MS/MS and UV-vis data, with literature comparison.	Level 3 ★★★☆☆	3/ [2]
**c. Furofuran-type lignans**													
**1**	(+)-Pinoresinol	$C_{20}H_{22}O_6$	358.14164	InChI=1S/C20H22O6/c1-23-17-7-11(3-5-15(17)21)19-13-9-26-20(14(13)10-25-19)12-4-6-16(22)18(8-12)24-2/h3-8,13-14,19-22H,9-10H2,1-2H3/t13-,14-,19+,20+/m0/s1	COC1=C(C=CC(=C1)C2C3COC(C3CO2)C4=CC(=C(C=C4)O)OC)O	73399	487-36-5	CHEMBL260183	Leaves, skin, pomace, and olive oil (VOO& EVOO)	[30, 33, 63, 73, 85, 149]	1/ Isolation by preparative HPLC, full structural elucidation (1D; 1H and 13C and 2D; COSY, HMQC) NMR, and NMR/MS matched literature.	Level 1 ★★★★★	1/ [150]

No.	Compound name	Molecular formula	Exact mass	InChI	SMILES	CID	CAS Number	ChEMBL ID	Part used	References related to the part used	Identification Confidence*	Level 1 ★★★★★<br>Level 2 ★★★★☆<br>Level 3 ★★★☆☆<br>Level 4 ★★☆☆☆<br>Level 5 ★☆☆☆☆	References related to confidence level
**2**	Hydroxypinoresinol	$C_{20}H_{22}O_7$	374.136555	InChI=1S/C20H22O7/c1-24-16-7-11(3-5-14(16)21)18-13-9-26-19(20(13,23)10-27-18)12-4-6-15(22)17(8-12)25-2/h3-8,13,18-19,21-23H,9-10H2,1-2H3/t13-,18-,19-,20-/m1/s1	COC1=C(C=CC(=C1)C2C3COC(C3(CO2)O)C4=CC(=C(C=C4)O)OC)O	3010930	81426-17-7	CHEMBL471274	Pomace, olive oil (VOO), leaves, and bark	25, 33, 63	1/ Isolation via column chromatography and preparative TLC, full structural elucidation (1H, 13C), then spectral data (IR, UV), chemical derivatization.<br><br>3/ Identification based on chromatography RPLC–DAD–ESI-MS, UV, and MS data compared to literature, without a reference standard or structural validation	Level 1 ★★★★★<br><br>Level 3 ★★★☆☆	1/ 151<br><br>3/ 32
**3**	1-Hydroxypinoresinol 4'-β-D glucoside	$C_{26}H_{32}O_{12}$	536.18938	InChI=1S/C26H32O12/c1-33-17-8-13(3-5-15(17)28)24-26(32)11-36-23(14(26)10-35-24)12-4-6-16(18(7-12)34-2)37-25-22(31)21(30)20(29)19(9-27)38-25/h3-8,14,19-25,27-32H,9-11H2,1-2H3/t14-,19-,20-,21+,22-,23-,24-,25-,26-/m1/s1	COC1=C(C=CC(=C1)C2C3COC(C3(CO2)O)C4=CC(=C(C=C4)O)OC)OC5C(C(C(C(O5)CO)O)O)O	21591950	102582-69-4	CHEMBL479730	Stems, leaves, bark, wood, roots, and OLS	2, 12, 33, 40, 66	3/ Identification relied on mass spectrometry and MS/MS fragments using RP-HPLC-DAD-ESI-QTOF-MS/MS and UV-vis data, with literature comparison.	Level 3 ★★★☆☆	3/ 2
**4**	(+)-1-Acetoxypinoresinol	$C_{22}H_{24}O_8$	416.14712	InChI=1S/C22H24O8/c1-12(23)30-22-11-29-20(13-4-6-16(24)18(8-13)26-2)15(22)10-28-21(22)14-5-7-17(25)19(9-14)27-3/h4-9,15,20-21,24-25H,10-11H2,1-3H3/t15-,20-,21-,22-/m1/s1	CC(=O)OC12COC(C1COC2C3=CC(=C(C=C3)O)OC)C4=CC(=C(C=C4)O)OC	442831	81426-14-4	NF	Roots, pomace, leaves, stems, bark, and olive oil (VOO& EVOO)	25, 33, 63, 96	1/ isolated then purified by column chromatography and PTLC, identified through 1D (1H and 13C) and 2D (COSY, HMBC) NMR, HREIMS, IR, UV spectrophotometer techniques, and confirmed by literature.	Level 1 ★★★★★	1/ 148
**5**	(+) 1-Acetoxypinoresinol 4'-β-D glucoside	$C_{28}H_{34}O_{13}$	578.199945	InChI=1S/C28H34O13/c1-13(30)41-28-12-38-25(14-4-6-17(31)19(8-14)35-2)16(28)11-37-26(28)15-5-7-18(20(9-15)36-3)39-27-24(34)23(33)22(32)21(10-29)40-27/h4-9,16,21-27,29,31-34H,10-12H2,1-3H3	CC(=O)OC12COC(C1COC2C3=CC(=C(C=C3)OC4C(C(C(C(O4)CO)O)O)O)OC)C5=CC(=C(C=C5)O)OC	14756309	NF	NF	Stems, branches, leaves, bark, roots, and olive oil (EVOO)	33, 40, 77, 96	4/ Identification based on MS/MS and UV data without structural confirmation.	Level 4 ★★☆☆☆	4/ 77
**6**	(+)-1-Acetoxypinoresinol-4″-*O*-methyl ether	$C_{23}H_{26}O_8$	430.16277	InChI=1S/C23H26O8/c1-13(24)31-23-12-30-21(14-6-8-18(26-2)20(9-14)28-4)16(23)11-29-22(23)15-5-7-17(25)19(10-15)27-3/h5-10,16,21-22,25H,11-12H2,1-4H3	CC(OC12COC(C3=CC(OC)=C(OC)C=C3)C1COC2C4=CC(OC)=C(O)C=C4)=O	NF	NF	NF	Bark	33	1/ Isolation via column chromatography and preparative TLC, full structural elucidation (1H, 13C), then spectral data (IR, UV), chemical derivatization.	Level 1 ★★★★★	1/ 151

No.	Compound name	Molecular formula	Exact mass	InChI	SMILES	CID	CAS Number	ChEMBL ID	Part used	References related to the part used	Identification Confidence*	Level 1 ★★★★★<br>Level 2 ★★★★☆<br>Level 3 ★★★☆☆<br>Level 4 ★★☆☆☆<br>Level 5 ★☆☆☆☆	References related to confidence level
**7**	(+)-1-Acetoxypinoresinol-4′-β-D-glucopyranoside-4″-*O*-methyl ether	$C_{29}H_{36}O_{13}$	592.215595	InChI=1S/C29H36O13/c1-14(31)42-29-13-39-26(15-5-7-18(35-2)20(9-15)37-4)17(29)12-38-27(29)16-6-8-19(36-3)21(10-16)40-28-25(34)24(33)23(32)22(11-30)41-28/h5-10,17,22-28,30,32-34H,11-13H2,1-4H3	CC(OC12COC(C1COC2C3=CC(OC4C(C(C(C(O4)CO)O)O)O)=C(C=C3)OC)C5=CC(OC)=C(C=C5)OC)=O	NF	NF	NF	Bark and skin	33, 105	1/ Isolated by different chromatographic techniques (TLC, HPLC), elucidated through 1D NMR (1H and 13C NMR), 2D NMR (COSY), and then identified by MS was used.	Level 1 ★★★★★	1/ [105]
**8**	Syringaresinol	$C_{22}H_{26}O_8$	418.16277	InChI=1S/C22H26O8/c1-25-15-5-11(6-16(26-2)19(15)23)21-13-9-30-22(14(13)10-29-21)12-7-17(27-3)20(24)18(8-12)28-4/h5-8,13-14,21-24H,9-10H2,1-4H3	COC1=CC(=CC(=C1O)OC)C2C3COC(C3CO2)C4=CC(=C(C(=C4)OC)O)OC	100067	1177-14-6	CHEMBL4469429	Leaves and olive oil (EVOO)	152, 153	2/ Analysis by LC-SPE-NMR with Rt matching (18.2 min), structural elucidation via 1D (1H and 13C) and 2D NMR (TOCSY) compared with prior literature (isolation studies).	Level 2 ★★★★☆	2/ [28]
**9**	Fraxiresinol 1-*O*-β-D-glucoside (methoxypinoresinol glucoside)	$C_{27}H_{34}O_{13}$	566.199945	InChI=1S/C27H34O13/c1-34-16-6-12(4-5-15(16)29)24-14-10-37-25(13-7-17(35-2)20(30)18(8-13)36-3)27(14,11-38-24)40-26-23(33)22(32)21(31)19(9-28)39-26/h4-8,14,19,21-26,28-33H,9-11H2,1-3H3/t14-,19-,21-,22+,23-,24-,25-,26+,27-/m1/s1	COC1=CC(=CC(=C1O)OC)C2C3(COC(C3CO2)C4=CC(=C(C=C4)O)OC)OC5C(C(C(C(O5)CO)O)O)O	21632948	89199-94-0	NF	Bark, stems, and roots	33, 96	4/ Identification relied on mass spectrometry and MS/MS fragmentation using UPLC-Q-TOF/MS, and Literature comparison for spectral data.	Level 4 ★★☆☆☆	4/ [72]
					**10- Terpenes**								
**a. Monoterpenes**													
**1**	Myrcene (7-methyl-3-methyleneocta-1,6-diene)	$C_{10}H_{16}$	136.1252	InChI=1S/C10H16/c1-5-10(4)8-6-7-9(2)3/h5,7H,1,4,6,8H2,2-3H3	CC(=CCCC(=C)C=C)C	31253	123-35-3	CHEMBL455491	Leaves, stems, and olive oil (EVOO)	35, 154, 155	2/ Identification via GC-IT-MS with HS-SPME and LRI matched to the NIST05 mass spectral library (Rt+MS), confirmed via pure standard.<br><br>4/ Identification based on comparison of Rt, linear retention indices relative to n-hydrocarbons, and computer matching against commercial [NIST 98, ADAMS] and homemade library mass spectra... Molecular weights confirmed by GC-CIMS.	Level 2 ★★★★☆<br><br>Level 4 ★★☆☆☆	2/ [155]<br><br>4/ [35]

No.	Compound name	Molecular formula	Exact mass	InChI	SMILES	CID	CAS Number	ChEMBL ID	Part used	References related to the part used	Identification Confidence*	Level 1 ★★★★★<br>Level 2 ★★★★☆<br>Level 3 ★★★☆☆<br>Level 4 ★★☆☆☆<br>Level 5 ★☆☆☆☆	References related to confidence level
**2**	Linalool (3,7-dimethylocta-1,6-dien-3-ol)	$C_{10}H_{18}O$	154.135765	InChI=1S/C10H18O/c1-5-10(4,11)8-6-7-9(2)3/h5,7,11H,1,6,8H2,2-4H3	CC(=CCCC(C)(C=C)O)C	6549	78-70-6	CHEMBL25306	Leaves, stems, and olive oil (EVOO)	[35, 70]	2/ Identification via GC-IT-MS with HS-SPME and LRI matched to the NIST05 mass spectral library (Rt+MS), confirmed via pure standard.<br><br>4/ Identification based on comparison of Rt, linear retention indices relative to n-hydrocarbons, and computer matching against commercial [NIST 98, ADAMS] and homemade library mass spectra... Molecular weights confirmed by GC-CIMS.	Level 2 ★★★★☆<br><br>Level 4 ★★☆☆☆	2/ [155]<br><br>4/ [35]
**3**	Geranylacetone	$C_{13}H_{22}O$	194.167065	InChI=1S/C13H22O/c1-11(2)7-5-8-12(3)9-6-10-13(4)14/h7,9H,5-6,8,10H2,1-4H3/b12-9+	CC(=CCCC(=CCCC(=O)C)C)C	1549778	3796-70-1	NF	Stems and olive oil	[35, 156]	4/ Identification based on comparison of Rt, linear retention indices relative to n-hydrocarbons, and computer matching against commercial [NIST 98, ADAMS] and homemade library mass spectra... Molecular weights confirmed by GC-CIMS.	Level 4 ★★☆☆☆	4/ [35]
**4**	α-Terpineol	$C_{10}H_{18}O$	154.135765	InChI=1S/C10H18O/c1-8-4-6-9(7-5-8)10(2,3)11/h4,9,11H,5-7H2,1-3H3	CC1=CCC(CC1)C(C)(C)O	17100	98-55-5	CHEMBL449810	Stems, leaves, and olive oil	[35, 70]	2/ Identification via GC-IT-MS with HS-SPME matched a pure standard and the NIST05 mass spectral library (Rt+MS), confirmed via pure standard.<br><br>4/ Identification based on comparison of Rt, linear retention indices relative to n-hydrocarbons, and computer matching against commercial [NIST 98, ADAMS] and homemade library mass spectra... Molecular weights confirmed by GC-CIMS.	Level 2 ★★★★☆<br><br>Level 4 ★★☆☆☆	2/ [155]<br><br>4/ [35]
**5**	Oleuropeic acid	$C_{10}H_{16}O_3$	184.109945	InChI=1S/C10H16O3/c1-10(2,13)8-5-3-7(4-6-8)9(11)12/h3,8,13H,4-6H2,1-2H3,(H,11,12)/t8-/m1/s1	CC(C)(C1CCC(=CC1)C(=O)O)O	188320	5027-76-9	CHEMBL4796626	Wood and fruits	[12, 157]	3/ Identification via chromatography with UV/IR + multi-technique validation (titration, degradation chemistry; H_2/Pd, acid cleavage, dehydrogenation, and optical activity), but no co-chromatography with a physical standard due to novelty.	Level 3 ★★★☆☆	3/ [157]

No.	Compound name	Molecular formula	Exact mass	InChI	SMILES	CID	CAS Number	ChEMBL ID	Part used	References related to the part used	Identification Confidence*	Level 1 ★★★★★<br>Level 2 ★★★★☆<br>Level 3 ★★★☆☆<br>Level 4 ★★☆☆☆<br>Level 5 ★☆☆☆☆	References related to confidence level
**6**	Oleuropeic acid 8-*O*-glucoside	$C_{16}H_{26}O_8$	346.16277	InChI=1S/C16H26O8/c1-16(2,9-5-3-8(4-6-9)14(21)22)24-15-13(20)12(19)11(18)10(7-17)23-15/h3,9-13,15,17-20H,4-7H2,1-2H3,(H,21,22)/t9-,10-,11-,12+,13-,15+/m1/s1	CC(C1CCC(C(O)=O)=CC1)(OC2C(O)C(O)C(O)C(CO)O2)C	11152176	865887-46-3	CHEMBL1478642	Wood	[158]	3/ Identification via HPLC-DAD technique compared to oleuropeic acid as reference standard.	Level 3 ★★★☆☆	3/ [158]
**7**	α-Pinene	$C_{10}H_{16}$	136.1252	InChI=1S/C10H16/c1-7-4-5-8-6-9(7)10(8,2)3/h4,8-9H,5-6H2,1-3H3	CC1=CCC2CC1C2(C)C	6654	80-56-8	CHEMBL442565	Leaves, stems, and olive oil (EVOO)	[35, 70, 154, 155]	2/ Identification via GC-IT-MS with HS-SPME and LRI matched to the NIST05 mass spectral library (Rt+MS), confirmed via pure standard.<br><br>4/ Identification based on comparison of Rt, linear retention indices relative to n-hydrocarbons, and computer matching against commercial [NIST 98, ADAMS] and homemade library mass spectra... Molecular weights confirmed by GC-CIMS.	Level 2 ★★★★☆<br><br>Level 4 ★★☆☆☆	2/ [155]<br><br>4/ [35]
**8**	Camphene	$C_{10}H_{16}$	136.1252	InChI=1S/C10H16/c1-7-8-4-5-9(6-8)10(7,2)3/h8-9H,1,4-6H2,2-3H3	CC1(C2CCC(C2)C1=C)C	6616	79-92-5	CHEMBL2268550	Leaves and olive oil (EVOO)	[155, 159]	2/ Identification via GC-IT-MS with HS-SPME matched a pure chemical standard and the NIST05 mass spectral library (Rt+MS).	Level 2 ★★★★☆	2/ [155]
**9**	Linalool oxide (furanoid)	$C_{10}H_{18}O_2$	170.130679	InChI=1S/C10H18O2/c1-5-10(4)7-6-8(12-10)9(2,3)11/h5,8,11H,1,6-7H2,2-4H3/t8-,10+/m0/s1	CC1(CCC(O1)C(C)(C)O)C=C	6432254	34995-77-2	CHEMBL2252947	Stems	[35]	4/ Identification based on comparison of Rt, linear retention indices relative to n-hydrocarbons, and computer matching against commercial [NIST 98, ADAMS] and homemade library mass spectra... Molecular weights confirmed by GC-CIMS.	Level 4 ★★☆☆☆	4/ [35]
**10**	Eugenol	$C_{10}H_{12}O_2$	164.08373	InChI=1S/C10H12O2/c1-3-4-8-5-6-9(11)10(7-8)12-2/h3,5-7,11H,1,4H2,2H3	COC1=C(C=CC(=C1)CC=C)O	3314	97-53-0	CHEMBL42710	Leaves and stems	[35, 70]	3/ identified using GC-MS with UV, and its Rt was compared with literature.	Level 3 ★★★☆☆	3/ [35]

No.	Compound name	Molecular formula	Exact mass	InChI	SMILES	CID	CAS Number	ChEMBL ID	Part used	References related to the part used	Identification Confidence*	Level 1 ★★★★★<br>Level 2 ★★★★☆<br>Level 3 ★★★☆☆<br>Level 4 ★★☆☆☆<br>Level 5 ★☆☆☆☆	References related to confidence level
**11**	ρ-Cymen-8-ol / (2-(4-methylphenyl)propan-2-ol)	$C_{10}H_{14}O$	150.104465	InChI=1S/C10H14O/c1-8-4-6-9(7-5-8)10(2,3)11/h4-7,11H,1-3H3	CC1=CC=C(C=C1)C(C)(C)O	14529	1197-01-9	CHEMBL3186822	Stems and leaves	35, 159	3/ Identification via GC-MS, retention index (KI = 9.90), mass spectral libraries, and comparison with literature retention indices and chemical family classification (monoterpenoid, C10)<br><br>4/ Identification based on comparison of Rt, linear retention indices relative to n-hydrocarbons, and computer matching against commercial [NIST 98, ADAMS] and homemade library mass spectra... Molecular weights confirmed by GC-CIMS.	Level 3 ★★★☆☆<br><br>Level 4 ★★☆☆☆	3/ [159]<br><br>4/ [35]
**b. Diterpenes**													
**1**	Phytol	$C_{20}H_{40}O$	296.307915	InChI=1S/C20H40O/c1-17(2)9-6-10-18(3)11-7-12-19(4)13-8-14-20(5)15-16-21/h15,17-19,21H,6-14,16H2,1-5H3/b20-15+/t18-,19-/m1/s1	CC(C)CCCC(C)CCCC(C)CCCC(=CCO)C	5280435	150-86-7	CHEMBL1644111	Leaves, fruits, and olive oil (EVOO)	160-162	3/ Identification via GC-MS, retention index (n-alkanes), mass spectral libraries (NIST14, Wiley Registry), and comparison with Pure compounds, literature retention indices.	Level 3 ★★★☆☆	3/ [161]
**2**	α-Tocopherol (Vit. E)	$C_{29}H_{50}O_2$	430.38108	InChI=1S/C29H50O2/c1-20(2)12-9-13-21(3)14-10-15-22(4)16-11-18-29(8)19-17-26-25(7)27(30)23(5)24(6)28(26)31-29/h20-22,30H,9-19H2,1-8H3/t21-,22-,29-/m1/s1	CC1=C(C2=C(CCC(O2)(C)CCCC(C)CCCC(C)CCCC(C)C)C(=C1O)C)C	14985	59-02-9	CHEMBL47	Leaves, fruits, pomace, and olive oil (VOO)	85, 118	3/ Identification via GC-MS, retention index (n-alkanes), mass spectral libraries (NIST14, Wiley Registry), and comparison with Pure compounds, literature retention indices.	Level 3 ★★★☆☆	3/ [161]
**3**	β-Tocopherol	$C_{28}H_{48}O_2$	416.36543	InChI=1S/C28H48O2/c1-20(2)11-8-12-21(3)13-9-14-22(4)15-10-17-28(7)18-16-25-24(6)26(29)19-23(5)27(25)30-28/h19-22,29H,8-18H2,1-7H3/t21-,22-,28-/m1/s1	CC1=CC(=C(C2=C1OC(CC2)(C)CCCC(C)CCCC(C)CCCC(C)C)C)O	6857447	148-03-8	NF	Leaves, fruit, pomace, seed oil, and olive oil (VOO)	85, 118	2/ Identification via GC-APCI-MS (Rt + HRMS/MSMS) and comparison with authentic chemical standard	Level 2 ★★★★☆	2/ [85]
**4**	γ-Tocopherol	$C_{28}H_{48}O_2$	416.36543	InChI=1S/C28H48O2/c1-20(2)11-8-12-21(3)13-9-14-22(4)15-10-17-28(7)18-16-25-19-26(29)23(5)24(6)27(25)30-28/h19-22,29H,8-18H2,1-7H3/t21-,22-,28-/m1/s1	CC1=C(C=C2CCC(OC2=C1C)(C)CCCC(C)CCCC(C)CCCC(C)C)O	92729	54-28-4	CHEMBL2151591	Leaves, fruit, pomace, seed oil, and olive oil (VOO&EVOO)	85, 118, 159, 163	2/ Identification via GC-APCI-MS (Rt + HRMS/MSMS) and comparison with authentic chemical standard	Level 2 ★★★★☆	2/ [85]

No.	Compound name	Molecular formula	Exact mass	InChI	SMILES	CID	CAS Number	ChEMBL ID	Part used	References related to the part used	Identification Confidence*	Level 1 ★★★★★<br>Level 2 ★★★★☆<br>Level 3 ★★★☆☆<br>Level 4 ★★☆☆☆<br>Level 5 ★☆☆☆☆	References related to confidence level
**5**	δ-Tocopherol	$C_{27}H_{46}O_2$	402.34978	InChI=1S/C27H46O2/c1-20(2)10-7-11-21(3)12-8-13-22(4)14-9-16-27(6)17-15-24-19-25(28)18-23(5)26(24)29-27/h18-22,28H,7-17H2,1-6H3/t21-,22-,27-/m1/s1	CC1=CC(=CC2=C1OC(CC2)(C)CCCC(C)CCCC(C)CCCC(C)C)O	92094	119-13-1	CHEMBL1451395	Olive oil (VOO)	[164]	2/ Identification via GC-APCI-MS (Rt + HRMS/MSMS) and comparison with authentic chemical standard	Level 2 ★★★★☆	2/ [85]
**6**	Hexahydrofarnesyl acetone (6,10,14-trimethylpentadecan-2-one)	$C_{18}H_{36}O$	268.276615	InChI=1S/C18H36O/c1-15(2)9-6-10-16(3)11-7-12-17(4)13-8-14-18(5)19/h15-17H,6-14H2,1-5H3	CC(C)CCCC(C)CCCC(C)CCCC(=O)C	10408	502-69-2	NF	Leaves	[159]	3/ Identification via GC-QTOF-MS (retention time, MS spectra) and comparison with literature retention indices and chemical family classification (sesquiterpenoids, C15).	Level 3 ★★★☆☆	3/ [159]
**c. Sesquiterpenes**													
**1**	Caryophyllene	$C_{15}H_{24}$	204.1878	InChI=1S/C15H24/c1-11-6-5-7-12(2)13-10-15(3,4)14(13)9-8-11/h6,13-14H,2,5,7-10H2,1,3-4H3/b11-6+/t13-,14-/m1/s1	CC1=CCCC(=C)C2CC(C2CC1)(C)C	5281515	87-44-5	CHEMBL445740	Leaves and olive oil	[154, 156]	3/ Identification via GC-MS, retention index (KI = 1424), mass spectral libraries, and comparison with literature retention indices and spectral databases (NIST).	Level 3 ★★★☆☆	3/ [156]
**2**	Caryophyllene oxide	$C_{15}H_{24}O$	220.182715	InChI=1S/C15H24O/c1-10-5-6-13-15(4,16-13)8-7-12-11(10)9-14(12,2)3/h11-13H,1,5-9H2,2-4H3/t11-,12-,13-,15-/m1/s1	CC1(CC2C1CCC3(C(O3)CCC2=C)C)C	1742210	1139-30-6	CHEMBL508894	Leaves and olive oil	[70, 156]	3/ Identification via GC-MS, retention index (KI = 1589), mass spectral libraries, and comparison with literature retention indices and spectral databases (NIST).	Level 3 ★★★☆☆	3/ [156]
**3**	γ-Elemene	$C_{15}H_{24}$	204.1878	InChI=1S/C15H24/c1-7-15(6)9-8-13(11(2)3)10-14(15)12(4)5/h7,14H,1,4,8-10H2,2-3,5-6H3/t14-,15+/m0/s1	CC(=C1CCC(C(C1)C(=C)C)(C)C=C)C	6432312	30824-67-0	NF	Leaves and olive oil (EVOO)	[155, 159]	3/ Identification via GC-IT-MS with comparison of mass spectra to NIST05 library and literature data.	Level 3 ★★★☆☆	3/ [155]
**4**	Germacrene D	$C_{15}H_{24}$	204.1878	InChI=1S/C15H24/c1-12(2)15-10-8-13(3)6-5-7-14(4)9-11-15/h7-8,10,12,15H,3,5-6,9,11H2,1-2,4H3/b10-8+,14-7+/t15-/m0/s1	CC1=CCCC(=C)C=CC(CC1)C(C)C	5317570	23986-74-5	NF	Leaves and olive oil	[70, 156]	3/ Identification via GC-MS, retention index (KI = 1485), mass spectral libraries, and comparison with literature retention indices and spectral databases (NIST).	Level 3 ★★★☆☆	3/ [156]
**d. Triterpenes**													
**I. Aliphatic acyclic compound**													
**1**	Squalene	$C_{30}H_{50}$	410.39125	InChI=1S/C30H50/c1-25(2)15-11-19-29(7)23-13-21-27(5)17-9-10-18-28(6)22-14-24-30(8)20-12-16-26(3)4/h15-18,23-24H,9-14,19-22H2,1-8H3/b27-17+,28-18+,29-23+,30-24+	CC(=CCCC(=CCCC(=CCCC=C(C)CCC=C(C)CCC=C(C)C)C)C)C	638072	111-02-4	CHEMBL458402	Leaves, pomace, fruits, and olive oil (VOO)	[50, 165, 166]	3/ 13C NMR spectra compared with literature (Farines and Soulier, 1988). Stigmatadienes were interpreted but not isolated.	Level 3 ★★★☆☆	3/ [167]
**II. Pentacyclic triterpenes**													
**5-membered E-ring**													

No.	Compound name	Molecular formula	Exact mass	InChI	SMILES	CID	CAS Number	ChEMBL ID	Part used	References related to the part used	Identification Confidence*	Level 1 ★★★★★<br>Level 2 ★★★★☆<br>Level 3 ★★★☆☆<br>Level 4 ★★☆☆☆<br>Level 5 ★☆☆☆☆	References related to confidence level
**1**	Lupenol (lupeol)	$C_{30}H_{50}O$	426.386165	InChI=1S/C30H50O/c1-19(2)20-11-14-27(5)17-18-29(7)21(25(20)27)9-10-23-28(6)15-13-24(31)26(3,4)22(28)12-16-30(23,29)8/h20-25,31H,1,9-18H2,2-8H3/t20-,21+,22-,23+,24-,25+,27+,28-,29+,30+/m0/s1	CC(=C)C1CCC2(C1C3CCC4C5(CCC(C(C5CCC4(C3(CC2)C)C)(C)C)O)C)C	259846	545-47-1	CHEMBL289191	Fruits	168	3 /Identification via GC-MS (retention time, MS spectra) and comparison with literature MS fragmentation patterns and retention indices	Level 3 ★★★☆☆	3/ 168
**2**	Betulinic acid	$C_{30}H_{48}O_3$	456.360345	InChI=1S/C30H48O3/c1-18(2)19-10-15-30(25(32)33)17-16-28(6)20(24(19)30)8-9-22-27(5)13-12-23(31)26(3,4)21(27)11-14-29(22,28)7/h19-24,31H,1,8-17H2,2-7H3,(H,32,33)/t19-,20+,21-,22+,23-,24+,27-,28+,29+,30-/m0/s1	CC(=C)C1CCC2(C1C3CCC4C5(CCC(C(C5CCC4(C3(CC2)C)C)(C)C)O)C)C(=O)O	64971	472-15-1	CHEMBL269277	Leaves, stems, roots, fruits, pomace, and seeds	33, 43, 96	3/ Identification via LC-MS (ESI/APCI, negative/positive polarity) and compared to reference standard.	Level 3 ★★★☆☆	3/ 85
**6-membered E-ring**													
**1**	Ursolic acid	$C_{30}H_{48}O_3$	456.360345	InChI=1S/C30H48O3/c1-25(2)14-16-30(24(32)33)17-15-28(6)19(20(30)18-25)8-9-22-27(5)12-11-23(31)26(3,4)21(27)10-13-29(22,28)7/h8,20-23,31H,9-18H2,1-7H3,(H,32,33)/t20-,21-,22+,23-,27-,28+,29+,30-/m0/s1	CC1(CCC2(CCC3(C(=CCC4C3(CCC5C4(CCC(C5(C)C)O)C)C)C2C1)C)C(=O)O)C	64945	77-52-1	CHEMBL168	Leaves, branches, and fruits	33, 47	1/ Isolation and structural elucidation via NMR spectroscopy (1H, 13C) and comparison with literature data. The authors assert that the structure was "easily identified" using 1D NMR.<br><br>2/ Identification via HPLC-ESI-MS/MS matched (Rt) and MS/MS fragmentation data, and compared to the standard compound.<br><br>4/ Identification via UPLC-ESI-Q-TOF based on RT and HRMS (m/z), but no MS/MS, UV, or NMR data provided to confirm structural elucidation.	Level 1 ★★★★★<br><br>Level 2 ★★★★☆<br><br>Level 4 ★★☆☆☆	1/ 169<br><br>2/ 79<br><br>4/ 47
**2**	Oleanolic acid	$C_{30}H_{48}O_3$	456.360345	InChI=1S/C30H48O3/c1-25(2)14-16-30(24(32)33)17-15-28(6)19(20(30)18-25)8-9-22-27(5)12-11-23(31)26(3,4)21(27)10-13-29(22,28)7/h8,20-23,31H,9-18H2,1-7H3,(H,32,33)/t20-,21-,22+,23-,27-,28+,29+,30-/m0/s1	CC1(CCC2(CCC3(C(=CCC4C3(CCC5C4(CCC(C5(C)C)O)C)C)C2C1)C)C(=O)O)C	10494	508-02-1	NF	Flowers, fruits, pomace, olive oil, leaves, stems, branches, and roots	9, 40, 73, 76, 77, 96	1/ Isolated by different chromatographic techniques (e.g., PTLC; preparative thin layer chromatography), elucidated through 1D NMR (1H and 13C NMR), 2D NMR (COSY), identified by Fast atom bombardment - mass spectrometry (FAB-MS).<br><br>1/ Isolated by GC and GC-MS, elucidated through 1D and 2D NMR (COSY, HSQC, HETCOR).	Level 1 ★★★★★	1/ 105<br><br>1/ 170

No.	Compound name	Molecular formula	Exact mass	InChI	SMILES	CID	CAS Number	ChEMBL ID	Part used	References related to the part used	Identification Confidence*	Level 1 ★★★★★<br>Level 2 ★★★★☆<br>Level 3 ★★★☆☆<br>Level 4 ★★☆☆☆<br>Level 5 ★☆☆☆☆	References related to confidence level
**3**	Maslinic acid	$C_{30}H_{48}O_4$	472.35526	InChI=1S/C30H48O4/c1-25(2)12-14-30(24(33)34)15-13-28(6)18(19(30)16-25)8-9-22-27(5)17-20(31)23(32)26(3,4)21(27)10-11-29(22,28)7/h8,19-23,31-32H,9-17H2,1-7H3,(H,33,34)/t19-,20+,21-,22+,23-,27-,28+,29+,30-/m0/s1	CC1(CCC2(CCC3(C(=CCC4C3(CCC5C4(CC(C(C5(C)C)O)O)C)C)C2C1)C)C(=O)O)C	73659	4373-41-5	CHEMBL201515	Flowers, fruits, pomace, olive oil, seeds, leaves, stems, branches, and roots	9, 33, 40, 43, 77	1/ Isolated by different chromatographic techniques (e.g., PTLC; preparative thin layer chromatography), elucidated through 1D NMR (1H and 13C NMR), 2D NMR (COSY), identified by Fast atom bombardment - mass spectrometry (FAB-MS).<br>1/ Isolated by GC and GC-MS, elucidated through 1D and 2D NMR (COSY, HSQC, HETCOR).	Level 1 ★★★★★	1/ [105]<br>1/ [170]
**4**	Maslinic acid methyl ester	$C_{31}H_{50}O_4$	486.37091	InChI=1S/C31H50O4/c1-26(2)13-15-31(25(34)35-8)16-14-29(6)19(20(31)17-26)9-10-23-28(5)18-21(32)24(33)27(3,4)22(28)11-12-30(23,29)7/h9,20-24,32-33H,10-18H2,1-8H3/t20-,21+,22-,23+,24-,28-,29+,30+,31-/m0/s1	CC1(CCC2(CCC3(C(=CCC4C3(CCC5C4(CC(C(C5(C)C)O)O)C)C)C2C1)C)C(=O)OC)C	13653327	22425-82-7	CHEMBL202346	Leaves	66	4/ Identification via HPLC-DAD ESI/MS-TOF.	Level 4 ★★☆☆☆	4/ [66]
**5**	α-Amyrin	$C_{30}H_{50}O$	426.386165	InChI=1S/C30H50O/c1-19-11-14-27(5)17-18-29(7)21(25(27)20(19)2)9-10-23-28(6)15-13-24(31)26(3,4)22(28)12-16-30(23,29)8/h9,19-20,22-25,31H,10-18H2,1-8H3/t19-,20+,22+,23-,24+,25+,27-,28+,29-,30-/m1/s1	CC1CCC2(CCC3(C(=CCC4C3(CCC5C4(CCC(C5(C)C)O)C)C)C2C1C)C)C	73170	638-95-9	CHEMBL455357	* **α and β:** fruits and leaves<br>* **δ:** fruits, seeds	33, 50, 166	3/ Identification via GC-MS, retention index (n-alkanes), mass spectral libraries (NIST14, Wiley Registry), and comparison with Pure compounds, literature retention indices.	Level 3 ★★★☆☆	3/ [161]
**6**	Uvaol	$C_{30}H_{50}O_2$	442.38108	InChI=1S/C30H50O2/c1-19-10-15-30(18-31)17-16-28(6)21(25(30)20(19)2)8-9-23-27(5)13-12-24(32)26(3,4)22(27)11-14-29(23,28)7/h8,19-20,22-25,31-32H,9-18H2,1-7H3/t19-,20+,22+,23-,24+,25+,27+,28-,29-,30-/m1/s1	CC1CCC2(CCC3(C(=CCC4C3(CCC5C4(CCC(C5(C)C)O)C)C)C2C1C)C)CO	92802	545-46-0	CHEMBL399873	Fruits, olive oil, seeds, and leaves	33, 50, 171	3/ Identified via 1D (13C) NMR, only matching with purchased standards and literature.	Level 3 ★★★☆☆	3/ [172]
**7**	Erythrodiol	$C_{30}H_{50}O_2$	442.38108	InChI=1S/C30H50O2/c1-25(2)14-16-30(19-31)17-15-28(6)20(21(30)18-25)8-9-23-27(5)12-11-24(32)26(3,4)22(27)10-13-29(23,28)7/h8,21-24,31-32H,9-19H2,1-7H3/t21-,22-,23+,24-,27-,28+,29+,30+/m0/s1	CC1(CCC2(CCC3(C(=CCC4C3(CCC5C4(CCC(C5(C)C)O)C)C)C2C1)C)CO)C	101761	545-48-2	CHEMBL400074	Fruits, skin, olive oil, seeds, branches, and leaves	33, 50, 77	1/ Isolated by different chromatographic techniques (TLC, HPLC), elucidated through 1D NMR (1H and 13C NMR), 2D NMR (COSY), and then identified by MS was used.	Level 1 ★★★★★	1/ [105]

No.	Compound name	Molecular formula	Exact mass	InChI	SMILES	CID	CAS Number	ChEMBL ID	Part used	References related to the part used	Identification Confidence*	Level 1 ★★★★★<br>Level 2 ★★★★☆<br>Level 3 ★★★☆☆<br>Level 4 ★★☆☆☆<br>Level 5 ★☆☆☆☆	References related to confidence level
**8**	2-α-Hydroxyursolic acid (corosolic acid)	$C_{30}H_{48}O_4$	472.35526	InChI=1S/C30H48O4/c1-17-10-13-30(25(33)34)15-14-28(6)19(23(30)18(17)2)8-9-22-27(5)16-20(31)24(32)26(3,4)21(27)11-12-29(22,28)7/h8,17-18,20-24,31-32H,9-16H2,1-7H3,(H,33,34)/t17-,18+,20-,21+,22-,23+,24+,27+,28-,29-,30+/m1/s1	CC1CCC2(CCC3(C(=CCC4C3(CCC5C4(CC(C(C5(C)C)O)O)C)C)C2C1C)C)C(=O)O	6918774	4547-24-4	CHEMBL391533	Leaves and fruits	[79, 173]	1/ Isolation and structural elucidation via NMR spectroscopy (1H, 13C) and comparison with literature data. The authors assert that the structure was "easily identified" using 1D NMR.<br><br>2/ Identification via HPLC-ESI-MS/MS matched (Rt) and MS/MS fragmentation data, and compared to the standard compound.	Level 1 ★★★★★<br><br>Level 2 ★★★★☆	1/ [169]<br><br>2/ [79]
**9**	Dihydroxyursolic acid	$C_{30}H_{48}O_5$	488.350175	InChI=1S/C30H48O5/c1-17-9-12-30(25(34)35)14-13-28(5)19(23(30)18(17)2)7-8-22-26(3)15-20(32)24(33)27(4,16-31)21(26)10-11-29(22,28)6/h7,17-18,20-24,31-33H,8-16H2,1-6H3,(H,34,35)/t17-,18+,20-,21-,22-,23+,24+,26+,27-,28-,29-,30+/m1/s1	CC1CCC2(CCC3(C(=CCC4C3(CCC5C4(CC(C(C5(C)CO)O)O)C)C)C2C1C)C)C(=O)O	69570833	143839-02-5	NF	Leaves	[73]	3/ Identification via HPLC–PDA–ESI–MS/MS (Rt, UV, HRMS/MSMS) and comparison with Spectral libraries [174] and literature.	Level 3 ★★★☆☆	3/ [73]
**10**	Asiatic acid	$C_{30}H_{48}O_5$	488.350175	InChI=1S/C30H48O5/c1-17-9-12-30(25(34)35)14-13-28(5)19(23(30)18(17)2)7-8-22-26(3)15-20(32)24(33)27(4,16-31)21(26)10-11-29(22,28)6/h7,17-18,20-24,31-33H,8-16H2,1-6H3,(H,34,35)/t17-,18+,20-,21-,22-,23+,24+,26+,27+,28-,29-,30+/m1/s1	CC1CCC2(CCC3(C(=CCC4C3(CCC5C4(CC(C(C5(C)CO)O)O)C)C)C2C1C)C)C(=O)O	119034	464-92-6	CHEMBL404313	Leaves	[175]	2/ Identification via HPLC-ESI-MS/MS matched (Rt) and MS/MS fragmentation data, and compared to the standard compound.	Level 2 ★★★★☆	2/ [79]
**11**	Pomolic acid	$C_{30}H_{48}O_4$	472.35526	InChI=1S/C30H48O4/c1-18-10-15-30(24(32)33)17-16-27(5)19(23(30)29(18,7)34)8-9-21-26(4)13-12-22(31)25(2,3)20(26)11-14-28(21,27)6/h8,18,20-23,31,34H,9-17H2,1-7H3,(H,32,33)/t18-,20+,21-,22+,23-,26+,27-,28-,29-,30+/m1/s1	CC1CCC2(CCC3(C(=CCC4C3(CCC5C4(CCC(C5(C)C)O)C)C)C2C1(C)O)C)C(=O)O	382831	13849-91-7	CHEMBL486986	Leaves	[176]	1/ Isolation and structural elucidation via NMR spectroscopy (1H, 13C) and comparison with literature data. The authors assert that the structure was "easily identified" using 1D NMR.<br><br>4/ Analyzed using RP/HPLC-Q-TOF MS/MS analysis, and the identification based on precursor ion mass, Rt, and MS/MS fragmentation pattern matched against spectral libraries (NIST20, LipidBlast, MoNA).	Level 1 ★★★★★<br><br>Level 4 ★★☆☆☆	1/ [169]<br><br>4/ [64]

No.	Compound name	Molecular formula	Exact mass	InChI	SMILES	CID	CAS Number	ChEMBL ID	Part used	References related to the part used	Identification Confidence*	Level 1 ★★★★★<br>Level 2 ★★★★☆<br>Level 3 ★★★☆☆<br>Level 4 ★★☆☆☆<br>Level 5 ★☆☆☆☆	References related to confidence level
**12**	Rotundic acid	$C_{30}H_{48}O_5$	488.350175	InChI=1S/C30H48O5/c1-18-9-14-30(24(33)34)16-15-27(4)19(23(30)29(18,6)35)7-8-21-25(2)12-11-22(32)26(3,17-31)20(25)10-13-28(21,27)5/h7,18,20-23,31-32,35H,8-17H2,1-6H3,(H,33,34)/t18-,20-,21-,22+,23-,25+,26+,27-,28-,29-,30+/m1/s1	CC1CCC2(CCC3(C(=CCC4C3(CCC5C4(CCC(C5(C)CO)O)C)C)C2C1(C)O)C)C(=O)O	12315075	20137-37-5	CHEMBL1271052	Olive cell suspension	[169, 176]	1/ Isolation and structural elucidation via NMR spectroscopy (1H, 13C) and comparison with literature data. The authors assert that the structure was "easily identified" using 1D NMR.	Level 1 ★★★★★	1/ [169]
**13**	Tormentic acid	$C_{30}H_{48}O_5$	488.350175	InChI=1S/C30H48O5/c1-17-10-13-30(24(33)34)15-14-27(5)18(22(30)29(17,7)35)8-9-21-26(4)16-19(31)23(32)25(2,3)20(26)11-12-28(21,27)6/h8,17,19-23,31-32,35H,9-16H2,1-7H3,(H,33,34)/t17-,19-,20+,21-,22-,23+,26+,27-,28-,29-,30+/m1/s1	CC1CCC2(CCC3(C(=CCC4C3(CCC5C4(CC(C(C5(C)C)O)O)C)C)C2C1(C)O)C)C(=O)O	73193	13850-16-3	CHEMBL239077	Fruits and leaves	[177]	1/ Isolation and structural elucidation via NMR spectroscopy (1H, 13C) and comparison with literature data. The authors assert that the structure was "easily identified" using 1D NMR.	Level 1 ★★★★★	1/ [169]
**14**	19-α-Hydroxyasiatic acid	$C_{30}H_{48}O_6$	504.34509	InChI=1S/C30H48O6/c1-17-9-12-30(24(34)35)14-13-27(4)18(22(30)29(17,6)36)7-8-21-25(2)15-19(32)23(33)26(3,16-31)20(25)10-11-28(21,27)5/h7,17,19-23,31-33,36H,8-16H2,1-6H3,(H,34,35)/t17-,19-,20-,21-,22-,23+,25+,26+,27-,28-,29-,30+/m1/s1	CC1CCC2(CCC3(C(=CCC4C3(CCC5C4(CC(C(C5(C)CO)O)O)C)C)C2C1(C)O)C)C(=O)O	490367	70868-78-9	CHEMBL515920	Olive cell suspension	[169, 176]	1/ Isolation and structural elucidation via NMR spectroscopy (1H, 13C) and comparison with literature data. The authors assert that the structure was "easily identified" using 1D NMR.	Level 1 ★★★★★	1/ [169]
**15**	3-*O*-Acetyl oleanolic acid	$C_{32}H_{50}O_4$	498.37091	InChI=1S/C32H50O4/c1-20(33)36-25-12-13-29(6)23(28(25,4)5)11-14-31(8)24(29)10-9-21-22-19-27(2,3)15-17-32(22,26(34)35)18-16-30(21,31)7/h9,22-25H,10-19H2,1-8H3,(H,34,35)/t22-,23-,24+,25-,29-,30+,31+,32-/m0/s1	CC(=O)OC1CCC2(C(C1(C)C)CCC3(C2CC=C4C3(CCC5(C4CC(CC5)(C)C)C(=O)O)C)C)C	151202	4339-72-4	CHEMBL486822	Olive tissue	[77]	3/ Identification via HRMS/MSMS (TOF-MS/MS) and comparison with theoretical m/z fragmentation.	Level 3 ★★★☆☆	3/ [178]
**16**	Hydroxy-oxo-oleanenoic acid	$C_{30}H_{46}O_4$	470.33961	InChI=1S/C30H46O4/c1-25(2)12-14-30(24(33)34)15-13-28(6)18(19(30)16-25)8-9-22-27(5)17-20(31)23(32)26(3,4)21(27)10-11-29(22,28)7/h8,19-22,31H,9-17H2,1-7H3,(H,33,34)	CC1(CCC2(CCC3(C(=CCC4C3(CCC5C4(CC(C(=O)C5(C)C)O)C)C)C2C1)C)C(=O)O)C	77503897	73584-62-0	NF	Leaves	[73]	3/ Identification relies on Chromatographic behavior (Rt), UV spectrum, and MS/MS fragmentation pattern using LC-ESI-QTOF-MS, with Literature comparison.	Level 3 ★★★☆☆	3/ [73]

No.	Compound name	Molecular formula	Exact mass	InChI	SMILES	CID	CAS Number	ChEMBL ID	Part used	References related to the part used	Identification Confidence*	Level 1 ★★★★★<br>Level 2 ★★★★☆<br>Level 3 ★★★☆☆<br>Level 4 ★★☆☆☆<br>Level 5 ★☆☆☆☆	References related to confidence level
**17**	Taraxerol	$C_{30}H_{50}O$	426.386165	InChI=1S/C30H50O/c1-25(2)17-18-27(5)13-9-21-29(7)14-10-20-26(3,4)24(31)12-16-28(20,6)22(29)11-15-30(21,8)23(27)19-25/h9,20,22-24,31H,10-19H2,1-8H3/t20-,22+,23+,24-,27-,28-,29-,30+/m0/s1	CC1(CCC2(CC=C3C4(CCC5C(C(CCC5(C4CCC3(C2C1C)C)C)O)(C)C)C)C)C	92097	127-22-0	CHEMBL511822	Fruits and seeds	[33]	3/ Identification via GC–MS (retention time, MS spectra) and comparison with literature data on triterpene alcohols.	Level 3 ★★★☆☆	3/ [179]
							**11. Phytosterols**						
**1**	Stigmasterol	$C_{29}H_{48}O$	412.370515	InChI=1S/C29H48O/c1-7-21(19(2)3)9-8-20(4)25-12-13-26-24-11-10-22-18-23(30)14-16-28(22,5)27(24)15-17-29(25,26)6/h8-10,19-21,23-27,30H,7,11-18H2,1-6H3/b9-8+/t20-,21-,23+,24+,25-,26+,27+,28+,29-/m1/s1	CCC(C=CC(C)C1CCC2C1(CCC3C2CC=C4C3(CCC(C4)O)C)C)C(C)C	5280794	83-48-7	CHEMBL400247	Fruits, olive oil and leaves	[159, 166, 180]	3/ Identification via 1H-NMR, implying reliance on literature-based spectroscopic parameters.	Level 3 ★★★☆☆	3/ [181]
**2**	β-Sitosterol (4-desmethylsterol)	$C_{29}H_{50}O$	414.386165	InChI=1S/C29H50O/c1-7-21(19(2)3)9-8-20(4)25-12-13-26-24-11-10-22-18-23(30)14-16-28(22,5)27(24)15-17-29(25,26)6/h10,19-21,23-27,30H,7-9,11-18H2,1-6H3/t20-,21-,23+,24+,25-,26+,27+,28+,29-/m1/s1	CCC(CCC(C)C1CCC2C1(CCC3C2CC=C4C3(CCC(C4)O)C)C)C(C)C	222284	83-46-5	CHEMBL221542	Leaves, fruits, and olive oil (VOO& EVOO)	[33, 160, 166, 180]	3/ 13C NMR spectra compared with literature (Farines and Soulier, 1988). Stigmatadienes were interpreted but not isolated.	Level 3 ★★★☆☆	3/ [167]
**3**	β-Sitosteryl ferulate	$C_{39}H_{58}O_4$	590.43351	InChI=1S/C39H58O4/c1-8-28(25(2)3)12-9-26(4)32-15-16-33-31-14-13-29-24-30(19-21-38(29,5)34(31)20-22-39(32,33)6)43-37(41)18-11-27-10-17-35(40)36(23-27)42-7/h10-11,13,17-18,23,25-26,28,30-34,40H,8-9,12,14-16,19-22,24H2,1-7H3/b18-11+/t26-,28-,30+,31+,32-,33+,34+,38+,39-/m1/s1	CCC(CCC(C)C1CCC2C1(CCC3C2CC=C4C3(CCC(C4)OC(=O)C=CC5=CC(=C(C=C5)O)OC)C)C)C(C)C	9938436	286011-30-1	CHEMBL3799667	Leaves	[69]	3/ Analysis was performed using LC-DAD-ESI-MS, identification based on Rt, UV-Vis spectra, MS, and M.wt., compared with literature data.	Level 3 ★★★☆☆	3/ [71]
**4**	Citrostadienol (4-monomethylsterol/ α-1-sitosterol)	$C_{30}H_{50}O$	426.386165	InChI=1S/C30H50O/c1-8-22(19(2)3)10-9-20(4)24-13-14-26-23-11-12-25-21(5)28(31)16-18-30(25,7)27(23)15-17-29(24,26)6/h8,11,19-21,24-28,31H,9-10,12-18H2,1-7H3/b22-8-/t20-,21+,24-,25+,26+,27+,28+,29-,30+/m1/s1	CC=C(CCC(C)C1CCC2C1(CCC3C2=CCC4C3(CCC(C4C)O)C)C)C(C)C	9548595	474-40-8	CHEMBL3184315	Fruits, seeds, and olive oil (EVOO)	[33, 160]	3/ The tentative identification relies on literature-reported NMR data (chemical shifts, multiplicities) rather than direct structural proof.	Level 3 ★★★☆☆	3/ [179]

No.	Compound name	Molecular formula	Exact mass	InChI	SMILES	CID	CAS Number	ChEMBL ID	Part used	References related to the part used	Identification Confidence*	Level 1 ★★★★★<br>Level 2 ★★★★☆<br>Level 3 ★★★☆☆<br>Level 4 ★★☆☆☆<br>Level 5 ★☆☆☆☆	References related to confidence level
**5**	Gramisterol	$C_{29}H_{48}O$	412.370515	InChI=1S/C29H48O/c1-18(2)19(3)8-9-20(4)23-12-13-25-22-10-11-24-21(5)27(30)15-17-29(24,7)26(22)14-16-28(23,25)6/h10,18,20-21,23-27,30H,3,8-9,11-17H2,1-2,4-7H3/t20-,21+,23-,24+,25+,26+,27+,28-,29+/m1/s1	CC1C(CCC2(C1CC=C3C2CCC4(C3CCC4C(C)CCC(=C)C(C)C)C)C)O	5283640	1176-52-9	NF	Fruits, seeds, and olive oil (EVOO)	[33, 160]	3/ The tentative identification relies on literature-reported NMR data (chemical shifts, multiplicities) rather than direct structural proof.	Level 3 ★★★☆☆	3/ [179]
**6**	Obtusifoliol	$C_{30}H_{50}O$	426.386165	InChI=1S/C30H50O/c1-19(2)20(3)9-10-21(4)23-13-17-30(8)26-12-11-24-22(5)27(31)15-16-28(24,6)25(26)14-18-29(23,30)7/h19,21-24,27,31H,3,9-18H2,1-2,4-8H3/t21-,22+,23-,24+,27+,28+,29-,30+/m1/s1	CC1C2CCC3=C(C2(CCC1O)C)CCC4(C3(CCC4C(C)CCC(=C)C(C)C)C)C	65252	16910-32-0	CHEMBL481434	Fruits, seeds, and olive oil (EVOO)	[33, 160]	3/ The tentative identification relies on literature-reported NMR data (chemical shifts, multiplicities) rather than direct structural proof.	Level 3 ★★★☆☆	3/ [179]
**7**	Cycloeucalenol (3β-sterol)	$C_{30}H_{50}O$	426.386165	InChI=1S/C30H50O/c1-19(2)20(3)8-9-21(4)23-12-14-28(7)26-11-10-24-22(5)25(31)13-15-29(24)18-30(26,29)17-16-27(23,28)6/h19,21-26,31H,3,8-18H2,1-2,4-7H3/t21-,22+,23-,24+,25+,26+,27-,28+,29-,30+/m1/s1	CC1C2CCC3C4(CCC(C4(CCC35C2(C5)CCC1O)C)C(C)CCC(=C)C(C)C)C	101690	469-39-6	CHEMBL225634	Fruits and olive oil (EVOO)	[160, 179]	3/ Identification via GC–MS (Rt, MS spectra) and comparison with literature data on sterol derivatives.	Level 3 ★★★☆☆	3/ [179]
**8**	Cycloartenol (9-β,19-cyclo-24-lanosten-3β-ol)	$C_{30}H_{50}O$	426.386165	InChI=1S/C30H50O/c1-20(2)9-8-10-21(3)22-13-15-28(7)24-12-11-23-26(4,5)25(31)14-16-29(23)19-30(24,29)18-17-27(22,28)6/h9,21-25,31H,8,10-19H2,1-7H3/t21-,22-,23+,24+,25+,27-,28+,29-,30+/m1/s1	CC(CCC=C(C)C)C1CCC2(C1(CCC34C2CCC5C3(C4)CCC(C5(C)C)O)C)C	92110	469-38-5	CHEMBL225815	Fruits, seeds, and olive oil (EVOO)	[33, 160]	3/ Identification via 1H-NMR, implying reliance on literature-based spectroscopic parameters.	Level 3 ★★★☆☆	3/ [181]
**9**	24-Methylene cycloartenol (4,4'-dimethylsterol)	$C_{31}H_{52}O$	440.401815	InChI=1S/C31H52O/c1-20(2)21(3)9-10-22(4)23-13-15-29(8)25-12-11-24-27(5,6)26(32)14-16-30(24)19-31(25,30)18-17-28(23,29)7/h20,22-26,32H,3,9-19H2,1-2,4-8H3/t22-,23-,24+,25?,26+,28-,29+,30-,31+/m1/s1	CC(C)C(=C)CCC(C)C1CCC2(C1(CCC34C2CCC5C3(C4)CCC(C5(C)C)O)C)C	9547213	NF	NF	Fruits, seeds, and olive oil (EVOO)	[33, 160]	3/ Identification via GC–MS (Rt, MS spectra) and comparison with literature data on sterol derivatives.	Level 3 ★★★☆☆	3/ [179]

12- Fatty acids

a. Saturated fatty acids (SFAs)

No.	Compound name	Molecular formula	Exact mass	InChI	SMILES	CID	CAS Number	ChEMBL ID	Part used	References related to the part used	Identification Confidence*	Level 1 ★★★★★<br>Level 2 ★★★★☆<br>Level 3 ★★★☆☆<br>Level 4 ★★☆☆☆<br>Level 5 ★☆☆☆☆	References related to confidence level
**1**	Azelaic acid (1,9-nonanedioate)	$C_9H_{16}O_4$	188.10486	InChI=1S/C9H16O4/c10-8(11)6-4-2-1-3-5-7-9(12)13/h1-7H2,(H,10,11)(H,12,13)	C(CCCC(=O)O)CCCC(=O)O	2266	123-99-9	CHEMBL1238	Leaves	[182]	3/ Identification via HRMS (accurate mass, ppm error) and comparison with theoretical molecular formula ($C_9H_{16}O_4$)<br><br>5/ The identification of a tentative compound relies solely on mass accuracy, Rt, and HRMS without UV, MS/MS, or validation against a standard or literature.	Level 3 ★★★☆☆<br><br>Level 5 ★☆☆☆☆	3/ [182]<br><br>5/ [85]
**2**	Methyl pelargonate (methyl nonanoate)	$C_{10}H_{20}O_2$	172.14633	InChI=1S/C10H20O2/c1-3-4-5-6-7-8-9-10(11)12-2/h3-9H2,1-2H3	CCCCCCCCC(=O)OC	15606	1731-84-6	CHEMBL1234788	Olive oil (VOO), pomace, and olive callus	[183]	2/ Identification via GC-MS/MS (Rt + MS/MS) and direct comparison with external standards (Sigma-Aldrich reference compounds).	Level 2 ★★★★☆	2/ [184]
**3**	Lauric acid (dodecanoic acid)	$C_{12}H_{24}O_2$	200.17763	InChI=1S/C12H24O2/c1-2-3-4-5-6-7-8-9-10-11-12(13)14/h2-11H2,1H3,(H,13,14)	CCCCCCCCCCCC(=O)O	3893	143-07-7	CHEMBL108766	Olive oil (VOO), pomace, and leaves	[63, 66, 185]	3/ Identification via gas chromatography [5] and comparison with literature data on fatty acid profiles.	Level 3 ★★★☆☆	3/ [186]
**4**	Tridecanoic acid	$C_{13}H_{26}O_2$	214.19328	InChI=1S/C13H26O2/c1-2-3-4-5-6-7-8-9-10-11-12-13(14)15/h2-12H2,1H3,(H,14,15)	CCCCCCCCCCCCC(=O)O	12530	638-53-9	CHEMBL107874	Fruits and olive oil	[187, 188]	2/ Identification via UPLC–MS (Rt, HRMS) using reference standards matched to the theoretical molecular formula ($C_{13}H_{26}O_2$) and mass spectral data.	Level 2 ★★★★☆	2/ [188]
**5**	Myristic acid (tetradecanoic acid)	$C_{14}H_{28}O_2$	228.20893	InChI=1S/C14H28O2/c1-2-3-4-5-6-7-8-9-10-11-12-13-14(15)16/h2-13H2,1H3,(H,15,16)	CCCCCCCCCCCCCC(=O)O	11005	544-63-8	CHEMBL111077	Leaves, pomace, and olive oil (VOO& EVOO)	[63, 118, 189]	2/ Identification via UPLC–MS (Rt, HRMS) using reference standards matched to the theoretical molecular formula ($C_{14}H_{28}O_2$) and mass spectral data.	Level 2 ★★★★☆	2/ [188]
**6**	Pentadecanoic acid	$C_{15}H_{30}O_2$	242.22458	InChI=1S/C15H30O2/c1-2-3-4-5-6-7-8-9-10-11-12-13-14-15(16)17/h2-14H2,1H3,(H,16,17)	CCCCCCCCCCCCCCC(=O)O	13849	1002-84-2	CHEMBL460025	Olive oil	[188]	2/ Identification via UPLC–MS (Rt, HRMS) using reference standards matched to the theoretical molecular formula ($C_{15}H_{30}O_2$) and mass spectral data.	Level 2 ★★★★☆	2/ [188]
**7**	Palmitic acid (hexadecanoic acid)	$C_{16}H_{32}O_2$	256.24023	InChI=1S/C16H32O2/c1-2-3-4-5-6-7-8-9-10-11-12-13-14-15-16(17)18/h2-15H2,1H3,(H,17,18)	CCCCCCCCCCCCCCCC(=O)O	985	57-10-3	CHEMBL82293	Leaves, branches, fruit, pomace, seed, wood, and olive oil (VOO& EVOO)	[47, 85, 143, 190]	3/ HPLC (UV detection) and 13C NMR for regiospecific analysis, validated via literature<br><br>4/ Detection by GC-MS without validation against standards or literature.	Level 3 ★★★☆☆<br><br>Level 4 ★★☆☆☆	3/ [191]<br><br>4/ [105]
**8**	Margaric acid (heptadecanoic acid)	$C_{17}H_{34}O_2$	270.25588	InChI=1S/C17H34O2/c1-2-3-4-5-6-7-8-9-10-11-12-13-14-15-16-17(18)19/h2-16H2,1H3,(H,18,19)	CCCCCCCCCCCCCCCCC(=O)O	10465	506-12-7	CHEMBL1172910	Pomace and olive oil (EVOO)	[118, 143]	2/ Identification via UPLC–MS (Rt, HRMS) using reference standards matched to the theoretical molecular formula ($C_{17}H_{34}O_2$) and mass spectral data.	Level 2 ★★★★☆	2/ [188]

No.	Compound name	Molecular formula	Exact mass	InChI	SMILES	CID	CAS Number	ChEMBL ID	Part used	References related to the part used	Identification Confidence*	Level 1 ★★★★★<br>Level 2 ★★★★☆<br>Level 3 ★★★☆☆<br>Level 4 ★★☆☆☆<br>Level 5 ★☆☆☆☆	References related to confidence level
**9**	Stearic acid (octadecanoic acid)	$C_{18}H_{36}O_2$	284.27153	InChI=1S/C18H36O2/c1-2-3-4-5-6-7-8-9-10-11-12-13-14-15-16-17-18(19)20/h2-17H2,1H3,(H,19,20)	CCCCCCCCCCCCCCCCCC(=O)O	5281	57-11-4	CHEMBL46403	Fruits, pomace, seeds, wood, and olive oil (VOO& EVOO)	85, 118, 143	3/ HPLC (UV detection) and 13C NMR for regiospecific analysis, validated via literature.<br>4/ Detection by GC-MS without validation against standards or literature.	Level 3 ★★★☆☆<br>Level 4 ★★☆☆☆	3/ [191]<br>4/ [105]
**10**	Arachidic acid (eicosanoic acid)	$C_{20}H_{40}O_2$	312.30283	InChI=1S/C20H40O2/c1-2-3-4-5-6-7-8-9-10-11-12-13-14-15-16-17-18-19-20(21)22/h2-19H2,1H3,(H,21,22)	CCCCCCCCCCCCCCCCCCCC(=O)O	10467	506-30-9	CHEMBL1173381	Pomace, fruit, wood, and olive oil (EVOO)	98, 118, 192	3/ Identification via gas chromatography [5] and comparison with legislative directives and fatty acid literature.<br>4/ Detection by GC-MS without validation against standards or literature.	Level 3 ★★★☆☆<br>Level 4 ★★☆☆☆	3/ [186]<br>4/ [105]
**11**	Heneicosanoic acid	$C_{21}H_{42}O_2$	326.31848	InChI=1S/C21H42O2/c1-2-3-4-5-6-7-8-9-10-11-12-13-14-15-16-17-18-19-20-21(22)23/h2-20H2,1H3,(H,22,23)	CCCCCCCCCCCCCCCCCCCCC(=O)O	16898	2363-71-5	CHEMBL1172909	Leaves and olive oil (EVOO)	192, 193	4/ Identification via LC-HRESIMS without using a reference standard.	Level 4 ★★☆☆☆	4/ [193]
**12**	Behenic acid (docosanoic acid)	$C_{22}H_{44}O_2$	340.33413	InChI=1S/C22H44O2/c1-2-3-4-5-6-7-8-9-10-11-12-13-14-15-16-17-18-19-20-21-22(23)24/h2-21H2,1H3,(H,23,24)	CCCCCCCCCCCCCCCCCCCCCC(=O)O	8215	112-85-6	CHEMBL1173474	Pomace, fruit, seeds, wood, and olive oil (VOO& EVOO)	98, 118, 143, 190	3/ Identification via gas chromatography [5] and comparison with legislative directives and fatty acid literature.<br>4/ Detection by GC-MS without validation against standards or literature.	Level 3 ★★★☆☆<br>Level 4 ★★☆☆☆	3/ [186]<br>4/ [105]
**13**	Lignoceric acid (tetracosanoic acid)	$C_{24}H_{48}O_2$	368.36543	InChI=1S/C24H48O2/c1-2-3-4-5-6-7-8-9-10-11-12-13-14-15-16-17-18-19-20-21-22-23-24(25)26/h2-23H2,1H3,(H,25,26)	CCCCCCCCCCCCCCCCCCCCCCCC(=O)O	11197	557-59-5	CHEMBL1173620	Pomace, fruit, leaves, and olive oil (VOO& EVOO)	63, 118, 143, 194	4/ Detection by GC-MS without validation against standards or literature.	Level 4 ★★☆☆☆	4/ [105]
**b. Monounsaturated fatty acids (MUSFAs)**													
**1**	Palmitoleic acid (cis-9-hexadecenoic acid)	$C_{16}H_{30}O_2$	254.22458	InChI=1S/C16H30O2/c1-2-3-4-5-6-7-8-9-10-11-12-13-14-15-16(17)18/h7-8H,2-6,9-15H2,1H3,(H,17,18)/b8-7-	CCCCCCC=CCCCCCCCC(=O)O	445638	373-49-9	CHEMBL453509	Pomace, fruit, seed oil, and olive oil (VOO& EVOO)	63, 85, 118, 143	3/ Identification via gas chromatography [5] and comparison with literature data on fatty acid profiles.<br>4/ Detection by GC-MS without validation against standards or literature.	Level 3 ★★★☆☆<br>Level 4 ★★☆☆☆	3/ [186]<br>4/ [105]

No.	Compound name	Molecular formula	Exact mass	InChI	SMILES	CID	CAS Number	ChEMBL ID	Part used	References related to the part used	Identification Confidence*	Level 1 ★★★★★<br>Level 2 ★★★★☆<br>Level 3 ★★★☆☆<br>Level 4 ★★☆☆☆<br>Level 5 ★☆☆☆☆	References related to confidence level
**2**	7-Hexadecenoic acid	$C_{16}H_{30}O_2$	254.22458	InChI=1S/C16H30O2/c1-2-3-4-5-6-7-8-9-10-11-12-13-14-15-16(17)18/h9-10H,2-8,11-15H2,1H3,(H,17,18)	CCCCCCCCC=CCCCCCC(=O)O	543268	NF	NF	Leaves and olive oil (VOO)	195-197	3/ Identification via GC-FID + GC-MS (DMDS adducts), comparison with international standards (IOOC, EC, Codex), and validation via Rt matching with standards + MS fragmentation vs. literature.	Level 3 ★★★☆☆	3/ [197]
**3**	Margaroleic acid (cis-9-heptadecenoic acid)	$C_{17}H_{32}O_2$	268.24023	InChI=1S/C17H32O2/c1-2-3-4-5-6-7-8-9-10-11-12-13-14-15-16-17(18)19/h8-9H,2-7,10-16H2,1H3,(H,18,19)/b9-8-	CCCCCCCC=CCCCCCCCC(=O)O	5282748	1981-50-6	NF	Fruits and olive oil (VOO& EVOO)	143, 190, 198	3/ Identification via GC-FID + GC-MS (DMDS adducts), comparison with international standards (IOOC, EC, Codex), and validation via Rt matching with standards + MS fragmentation vs. literature.	Level 3 ★★★☆☆	3/ [197]
**4**	Oleic Acid (*cis*-9-octadecenoic acid)	$C_{18}H_{34}O_2$	282.25588	InChI=1S/C18H34O2/c1-2-3-4-5-6-7-8-9-10-11-12-13-14-15-16-17-18(19)20/h9-10H,2-8,11-17H2,1H3,(H,19,20)/b10-9-	CCCCCCCCC=CCCCCCCCC(=O)O	445639	112-80-1	CHEMBL8659	Fruits, pomace, leaves, seeds, wood, and olive oil (VOO& EVOO)	63, 85, 98, 118, 143	3/ HPLC (UV detection) and 13C NMR for regiospecific analysis, validated via literature<br><br>4/ Detection by GC-MS without validation against standards or literature	Level 3 ★★★☆☆<br><br>Level 4 ★★☆☆☆	3/ [191]<br><br>4/ [105]
**5**	Oleic acid methyl ester (methyl oleate)	$C_{19}H_{36}O_2$	296.27153	InChI=1S/C19H36O2/c1-3-4-5-6-7-8-9-10-11-12-13-14-15-16-17-18-19(20)21-2/h10-11H,3-9,12-18H2,1-2H3/b11-10-	CCCCCCCCC=CCCCCCCCC(=O)OC	5364509	112-62-9	CHEMBL465725	Olive oil	199	4/ Identification via GC-FID based on Rt vs. FAME standards.	Level 4 ★★☆☆☆	4/ [199]
**6**	Vaccenic acid (*cis*-11-octadecenoic acid)	$C_{18}H_{34}O_2$	282.25588	InChI=1S/C18H34O2/c1-2-3-4-5-6-7-8-9-10-11-12-13-14-15-16-17-18(19)20/h7-8H,2-6,9-17H2,1H3,(H,19,20)/b8-7-	CCCCCCC=CCCCCCCCCCC(=O)O	5282761	506-17-2	CHEMBL1236642	Olive oil (VOO)	197	4/ Identification via 13C NMR based on its carbonyl resonance referenced to prior studies.	Level 4 ★★☆☆☆	4/ [200]
**7**	Gondoic acid (eicosenoic acid/ omega-9-fatty acid	$C_{20}H_{38}O_2$	310.28718	InChI=1S/C20H38O2/c1-2-3-4-5-6-7-8-9-10-11-12-13-14-15-16-17-18-19-20(21)22/h9-10H,2-8,11-19H2,1H3,(H,21,22)/b10-9-	CCCCCCCCC=CCCCCCCCCCC(=O)O	5282768	5561-99-9	CHEMBL4640620	Pomace, seeds, and olive oil (VOO& EVOO)	118, 143, 197	3/ Identification via gas chromatography [5] and comparison with legislative directives and fatty acid literature.<br><br>4/ Identification via 13C NMR based on its carbonyl resonance referenced to prior studies.	Level 3 ★★★☆☆<br><br>Level 4 ★★☆☆☆	3/ [186]<br><br>4/ [200]
**8**	2,3-dinor-8-iso-Prostaglandin F1 alpha	$C_{18}H_{32}O_5$	328.224975	InChI=1S/C18H32O5/c1-2-3-4-7-13(19)10-11-15-14(16(20)12-17(15)21)8-5-6-9-18(22)23/h10-11,13-17,19-21H,2-9,12H2,1H3,(H,22,23)/b11-10+/t13-,14-,15+,16-,17+/m0/s1	CCCCCC(C=CC1C(CC(C1CCCCC(=O)O)O)O)O	9548882	NF	NF	Leaves	124	3/ Identification via HRMS/MS2 (accurate mass, fragmentation) and comparison with Literature data.	Level 3 ★★★☆☆	3/ [124]
**c. Polyunsaturated fatty acids (PUSFAs)**													

No.	Compound name	Molecular formula	Exact mass	InChI	SMILES	CID	CAS Number	ChEMBL ID	Part used	References related to the part used	Identification Confidence*	Level 1 ★★★★★ Level 2 ★★★★☆ Level 3 ★★★☆☆ Level 4 ★★☆☆☆ Level 5 ★☆☆☆☆	References related to confidence level
**1**	Linoleic acid ((9Z,12Z)-octadeca-9,12-dienoic acid/ omega-6-fatty acid)	$C_{18}H_{32}O_2$	280.24023	InChI=1S/C18H32O2/c1-2-3-4-5-6-7-8-9-10-11-12-13-14-15-16-17-18(19)20/h6-7,9-10H,2-5,8,11-17H2,1H3,(H,19,20)/b7-6-,10-9-	CCCCCC=CC C=CCCCCCCC CC(=O)O	5280450	60-33-3	CHEMBL267476	Leaves, branches, fruit, pomace, seeds, and olive oil (VOO& EVOO)	47, 63, 118, 143	3/ HPLC (UV detection) and 13C NMR for regiospecific analysis, validated via literature.<br>4/ Detection by GC-MS without validation against standards or literature.	Level 3 ★★★☆☆<br>Level 4 ★★☆☆☆	3/ [191]<br>4/ [105]
**2**	α-Linolenic acid (9,12,15-octadecatrienoic acid/ omega-3-fatty acid)	$C_{18}H_{30}O_2$	278.22458	InChI=1S/C18H30O2/c1-2-3-4-5-6-7-8-9-10-11-12-13-14-15-16-17-18(19)20/h3-4,6-7,9-10H,2,5,8,11-17H2,1H3,(H,19,20)	CCC=CCC=C CC=CCCCCC CCC(=O)O	860	1955-33-5	CHEMBL3560296	Fruits, pomace, seeds, and olive oil (VOO& EVOO)	118, 143, 190, 201	3/ Identification via 1H/13C NMR chemical shifts compared with literature data. Cross-referenced with GC but no explicit use of co-chromatography with standards.	Level 3 ★★★☆☆	3/ [167]
					**13- Alkaloids**								
**1**	Cinchonine	$C_{19}H_{22}N_2O$	294.173213	InChI=1S/C19H22N2O/c1-2-13-12-21-10-8-14(13)11-18(21)19(22)16-7-9-20-17-6-4-3-5-15(16)17/h2-7,9,13-14,18-19,22H,1,8,10-12H2/t13-,14-,18+,19-/m0/s1	C=CC1CN2CC C1CC2C(C3= CC=NC4=CC= CC=C34)O	90454	118-10-5	CHEMBL588619	Leaves	107	5/ No experimental details or analytical proof provided.	Level 5 ★☆☆☆☆	5/ [107]
**2**	Cinchonidine	$C_{19}H_{22}N_2O$	294.173213	InChI=1S/C19H22N2O/c1-2-13-12-21-10-8-14(13)11-18(21)19(22)16-7-9-20-17-6-4-3-5-15(16)17/h2-7,9,13-14,18-19,22H,1,8,10-12H2/t13-,14-,18-,19+/m0/s1	C=CC1CN2CC C1CC2C(C3= CC=NC4=CC= CC=C34)O	101744	485-71-2	CHEMBL533841	Leaves	107	5/ No experimental details or analytical proof provided.	Level 5 ★☆☆☆☆	5/ [107]
**3**	10,11-Dihydrocinchonine	$C_{19}H_{24}N_2O$	296.188862	InChI=1S/C19H24N2O/c1-2-13-12-21-10-8-14(13)11-18(21)19(22)16-7-9-20-17-6-4-3-5-15(16)17/h3-7,9,13-14,18-19,22H,2,8,10-12H2,1H3	CCC1CN2CC C1CC2C(C3= CC=NC4=CC= CC=C34)O	101711	485-64-3	CHEMBL428695	Leaves	202	5/ No experimental details or analytical proof provided.	Level 5 ★☆☆☆☆	5/ [202]
					**14- Aldehyde and ketone compounds**								
**a. Aldehydic compounds**													
**1**	Vanillin	$C_8H_8O_3$	152.047345	InChI=1S/C8H8O3/c1-11-8-4-6(5-9)2-3-7(8)10/h2-5,10H,1H3	COC1=C(C=C C(=C1)C=O)O	1183	121-33-5	CHEMBL13883	Fruit, pomace, flowers, leaves, olive oil (EVOO), OOMW, wood, bark, and OLS	2, 9, 21, 30, 34	2/ Analysis by LC-SPE-NMR with Rt matching (13.6 min), structural elucidation via 1D (1H and 13C) and 2D NMR (TOCSY) compared with authentic standard.	Level 2 ★★★★☆	2/ [28]

No.	Compound name	Molecular formula	Exact mass	InChI	SMILES	CID	CAS Number	ChEMBL ID	Part used	References related to the part used	Identification Confidence*	Level 1 ★★★★★<br>Level 2 ★★★★☆<br>Level 3 ★★★☆☆<br>Level 4 ★★☆☆☆<br>Level 5 ★☆☆☆☆	References related to confidence level
**2**	Vanillin 4-*O*-β-D-glucoside (glucovanillin/ vanilloside)	$C_{14}H_{18}O_8$	314.10017	InChI=1S/C14H18O8/c1-20-9-4-7(5-15)2-3-8(9)21-14-13(19)12(18)11(17)10(6-16)22-14/h2-5,10-14,16-19H,6H2,1H3/t10-,11-,12+,13-,14-/m1/s1	COC1=C(C=CC(=C1)C=O)OC2C(C(C(C(O2)CO)O)O)O)O	6452133	494-08-6	NF	Pomace	[30]	4/ Identification via HPLC-DAD + HPLC-ESI-QToF-MSn (HRMS/MS²) and matched to literature/MassBank.	Level 4 ★★☆☆☆	4/ [30]
**3**	Benzaldehyde	C_7H_6O	106.041865	InChI=1S/C7H6O/c8-6-7-4-2-1-3-5-7/h1-6H	C1=CC=C(C=C1)C=O	240	100-52-7	CHEMBL15972	Stems, bark, and fruits	[35, 98, 203]	4/ Identification based on comparison of Rt, linear retention indices relative to n-hydrocarbons, and computer matching against commercial [NIST 98, ADAMS] and homemade library mass spectra... Molecular weights confirmed by GC-CIMS.	Level 4 ★★☆☆☆	4/ [35]
**4**	4-Hydroxybenzaldehyde	$C_7H_6O_2$	122.03678	InChI=1S/C7H6O2/c8-5-6-1-3-7(9)4-2-6/h1-5,9H	C1=CC(=CC=C1C=O)O	126	123-08-0	CHEMBL14193	Leaves	[64]	4/ Analyzed using RP/HPLC-Q-TOF MS/MS analysis, and the identification based on precursor ion mass, Rt, and MS/MS fragmentation pattern matched against spectral libraries (NIST20, LipidBlast, MoNA).	Level 4 ★★☆☆☆	4/ [64]
**5**	Phenylacetaldehyde	C_8H_8O	120.057515	InChI=1S/C8H8O/c9-7-6-8-4-2-1-3-5-8/h1-5,7H,6H2	C1=CC=C(C=C1)CC=O	998	122-78-1	CHEMBL1233464	Stems and fruits	[35, 203]	4/ Identification based on comparison of Rt, linear retention indices relative to n-hydrocarbons, and computer matching against commercial [NIST 98, ADAMS] and homemade library mass spectra. Molecular weights confirmed by GC-CIMS.	Level 4 ★★☆☆☆	4/ [35]
**6**	Coniferyl aldehyde (4-hydroxy-3-methoxycinnamaldehyde)	$C_{10}H_{10}O_3$	178.062995	InChI=1S/C10H10O3/c1-13-10-7-8(3-2-6-11)4-5-9(10)12/h2-7,12H,1H3/b3-2+	COC1=C(C=CC(=C1)C=CC=O)O	5280536	20649-42-7	CHEMBL242529	Leaves	[64]	4/ Analyzed using RP/HPLC-Q-TOF MS/MS analysis, and the identification based on precursor ion mass, Rt, and MS/MS fragmentation pattern matched against spectral libraries (NIST20, LipidBlast, MoNA)	Level 4 ★★☆☆☆	4/ [64]
**7**	Acetaldehyde	C_2H_4O	44.026215	InChI=1S/C2H4O/c1-2-3/h2H,1H3	CC=O	177	75-07-0	CHEMBL170365	Olive oil and wood	[12, 183, 204]	2/ Identification via HS-SPME-GC-FID (Rt, FID response) and comparison with spiked standards (Sigma-Aldrich) for calibration.	Level 2 ★★★★☆	2/ [204]
**8**	3-Methylbutanal (isovaleraldehyde)	$C_5H_{10}O$	86.073165	InChI=1S/C5H10O/c1-5(2)3-4-6/h4-5H,3H2,1-2H3	CC(C)CC=O	11552	590-86-3	CHEMBL18360	Fruits and olive oil (EVOO)	[57, 203]	2/ Identification via GC-IT-MS with HS-SPME and LRI matched to the NIST05 mass spectral library (Rt+MS), confirmed via pure standard.	Level 2 ★★★★☆	2/ [155]

No.	Compound name	Molecular formula	Exact mass	InChI	SMILES	CID	CAS Number	ChEMBL ID	Part used	References related to the part used	Identification Confidence*	Level 1 ★★★★★<br>Level 2 ★★★★☆<br>Level 3 ★★★☆☆<br>Level 4 ★★☆☆☆<br>Level 5 ★☆☆☆☆	References related to confidence level
**9**	2-Pentenal	C_5H_8O	84.057515	InChI=1S/C5H8O/c1-2-3-4-5-6/h3-5H,2H2,1H3/b4-3+	CCC=CC=O	5364752	1576-87-0	CHEMBL256368	Fruits and olive oil (EVOO)	[57, 203]	2/ Identification via GC-IT-MS with HS-SPME and LRI matched to the NIST05 mass spectral library (Rt+MS), confirmed via pure standard.	Level 2 ★★★★☆	2/ [155]
**10**	1-Hexanal	$C_6H_{12}O$	100.088815	InChI=1S/C6H12O/c1-2-3-4-5-6-7/h6H,2-5H2,1H3	CCCCCC=O	6184	66-25-1	CHEMBL280331	Fruits and olive oil (EVOO)	[57, 203]	2/ Identification via GC-IT-MS with HS-SPME and LRI matched to the NIST05 mass spectral library (Rt+MS), confirmed via pure standard.<br><br>4/ Identification by high-field 1H NMR and cross-validated with GC-MS data. Literature comparisons (e.g., Guth and Grosh, 1991), but no standards were explicitly used.	Level 2 ★★★★☆<br><br>Level 4 ★★☆☆☆	2/ [155]<br><br>4/ [167]
**11**	2-Hexenal	$C_6H_{10}O$	98.073165	InChI=1S/C6H10O/c1-2-3-4-5-6-7/h4-6H,2-3H2,1H3	CCCC=CC=O	10460	1335-39-3	NF	Stems, fruits, and olive oil (EVOO)	[35, 57, 203]	2/ Identification via GC-IT-MS with HS-SPME matched a pure chemical standard and the NIST05 mass spectral library (Rt+MS).<br><br>4/ Identified by high-field 1H NMR and cross-validated with GC-MS data. Literature comparisons (e.g., Guth and Grosh, 1991), but no standards were explicitly used.	Level 2 ★★★★☆<br><br>Level 4 ★★☆☆☆	2/ [155]<br><br>4/ [167]
**12**	Octanal	$C_8H_{16}O$	128.120115	InChI=1S/C8H16O/c1-2-3-4-5-6-7-8-9/h8H,2-7H2,1H3	CCCCCCCC=O	454	124-13-0	CHEMBL18407	Olive oil (EVOO) and olive callus	[35, 83, 183]	2/ Identification via GC-IT-MS with HS-SPME and LRI matched to the NIST05 mass spectral library (Rt+MS), confirmed via pure standard.<br><br>4/ Identification based on comparison of Rt, linear retention indices relative to n-hydrocarbons, and computer matching against commercial [NIST 98, ADAMS] and homemade library mass spectra... Molecular weights confirmed by GC-CIMS.	Level 2 ★★★★☆<br><br>Level 4 ★★☆☆☆	2/ [155]<br><br>4/ [35]

No.	Compound name	Molecular formula	Exact mass	InChI	SMILES	CID	CAS Number	ChEMBL ID	Part used	References related to the part used	Identification Confidence*	Level 1 ★★★★★<br>Level 2 ★★★★☆<br>Level 3 ★★★☆☆<br>Level 4 ★★☆☆☆<br>Level 5 ★☆☆☆☆	References related to confidence level
**13**	1-Nonanal (nonadienal)	$C_9H_{18}O$	142.135765	InChI=1S/C9H18O/c1-2-3-4-5-6-7-8-9-10/h9H,2-8H2,1H3	CCCCCCCCC=O	31289	124-19-6	CHEMBL2228376	Stems and olive oil (EVOO)	35, 83, 183	2/ Identification via GC-IT-MS with HS-SPME and LRI matched to the NIST05 mass spectral library (Rt+MS), confirmed via pure standard.<br><br>4/ Identification based on comparison of Rt, linear retention indices relative to n-hydrocarbons, and computer matching against commercial [NIST 98, ADAMS] and homemade library mass spectra... Molecular weights confirmed by GC-CIMS.	Level 2 ★★★★☆<br><br>Level 4 ★★☆☆☆	2/ [155]<br><br>4/ [35]
**14**	2-Decenal	$C_{10}H_{18}O$	154.135765	InChI=1S/C10H18O/c1-2-3-4-5-6-7-8-9-10-11/h8-10H,2-7H2,1H3/b9-8+	CCCCCCCC=CC=O	5283345	3913-81-3	CHEMBL507518	Stems and fruits	35, 203	4/ Identification based on comparison of Rt, linear retention indices relative to n-hydrocarbons, and computer matching against commercial [NIST 98, ADAMS] and homemade library mass spectra... Molecular weights confirmed by GC-CIMS.	Level 4 ★★☆☆☆	4/ [35]

b. Ketonic compounds

No.	Compound name	Molecular formula	Exact mass	InChI	SMILES	CID	CAS Number	ChEMBL ID	Part used	References related to the part used	Identification Confidence*	Level	References related to confidence level
**1**	Acetone	C_3H_6O	58.041865	InChI=1S/C3H6O/c1-3(2)4/h1-2H3	CC(=O)C	180	67-64-1	CHEMBL14253	Olive callus	183	5/ Tentative assignment based on NMR bucket (2.17 ppm). No MS or isolation. (Indirect evidence.).	Level 5 ★☆☆☆☆	5/ [6]
**2**	3-Pentanone	$C_5H_{10}O$	86.073165	InChI=1S/C5H10O/c1-3-5(6)4-2/h3-4H2,1-2H3	CCC(=O)CC	7288	96-22-0	CHEMBL45315	Extra olive oil	155	3/ Identification via GC-IT-MS and LRI with comparison of mass spectra to NIST05 library and literature data.	Level 3 ★★★☆☆	3/ [155]
**3**	Penten-3-one (ethyl vinyl ketone)	C_5H_8O	84.057515	InChI=1S/C5H8O/c1-3-5(6)4-2/h3H,1,4H2,2H3	CCC(=O)C=C	15394	1629-58-9	CHEMBL1506228	Olive oil (EVOO)	57	2/ Identification via GC-IT-MS with HS-SPME and LRI matched to the NIST05 mass spectral library (Rt+MS), confirmed via pure standard.	Level 2 ★★★★☆	2/ [155]
**4**	2,3-Dihydroxy-2,4-cyclopentadien-1-one	$C_5H_4O_3$	112.016045	InChI=1S/C5H4O3/c6-3-1-2-4(7)5(3)8/h1-2H,(H2,6,7,8)	C1=CC(=O)C(=C1O)O	14412554	124902-00-7	NF	Leaves	66	4/ Identification via HPLC-DAD ESI/MS-TOF.	Level 4 ★★☆☆☆	4/ [66]
**5**	Rengyolone (halleridone)	$C_8H_{10}O_3$	154.062995	InChI=1S/C8H10O3/c9-6-1-2-8(10)3-4-11-7(8)5-6/h1-2,7,10H,3-5H2/t7-,8+/m0/s1	C1COC2C1(C=CC(=O)C2)O	10725564	NF	NF	Leaves and fruits	33, 75, 193	3/ The separation was performed by preparative TLC, and identified by 1H NMR compared to literature.<br><br>3/ identified by GC-MS and confirmed by 1H NMR and 1D (COSY).	Level 3 ★★★☆☆	3/ [205]<br><br>3/ [29]

No.	Compound name	Molecular formula	Exact mass	InChI	SMILES	CID	CAS Number	ChEMBL ID	Part used	References related to the part used	Identification Confidence*	Level 1 ★★★★★<br>Level 2 ★★★★☆<br>Level 3 ★★★☆☆<br>Level 4 ★★☆☆☆<br>Level 5 ★☆☆☆☆	References related to confidence level
6	2,6-Dimethoxy-1,4-benzoquinone	$C_8H_8O_4$	168.04226	InChI=1S/C8H8O4/c1-11-6-3-5(9)4-7(12-2)8(6)10/h3-4H,1-2H3	COC1=CC(=O)C=C(C1=O)OC	68262	530-55-2	CHEMBL448515	Fruits	[39]	2/ Purification and structural elucidation via NMR spectroscopy + UHPLC-QQQ MS/MS (dMRM) and no explicit mention of 2D NMR or comparison to a commercial standard.	Level 2 ★★★★☆	2/ [39]
7	Cornoside	$C_{14}H_{20}O_8$	316.11582	InChI=1S/C14H20O8/c15-7-9-10(17)11(18)12(19)13(22-9)21-6-5-14(20)3-1-8(16)2-4-14/h1-4,9-13,15,17-20H,5-7H2/t9-,10-,11+,12-,13-/m1/s1	C1=CC(C=CC1=O)(CCOC2C(C(C(C(O2)CO)O)O)O)O	11809239	40661-45-8	NF	Pulp of the drupes and fruits	[33, 75]	2/ The isolation was performed by RP-MPLC, purified by preparative TLC, and identified by 1H NMR compared with the authentic cornoside standard.<br>3/ identified by GC-MS and confirmed by 1H NMR and 1D (COSY).	Level 2 ★★★★☆<br>Level 3 ★★★☆☆	2/ [205]<br>3/ [29]

15- Other Compounds

a. Amino acids

No.	Compound name	Molecular formula	Exact mass	InChI	SMILES	CID	CAS Number	ChEMBL ID	Part used	References related to the part used	Identification Confidence*	Level	References related to confidence level
1	Arginine	$C_6H_{14}N_4O_2$	174.111676	InChI=1S/C6H14N4O2/c7-4(5(11)12)2-1-3-10-6(8)9/h4H,1-3,7H2,(H,11,12)(H4,8,9,10)/t4-/m0/s1	C(CC(C(=O)O)N)CN=C(N)N	6322	74-79-3	CHEMBL1485	Fruits and pomace	[206, 207]	5/ Not explicitly identified or discussed	Level 5 ★☆☆☆☆	5/ [208]
2	Leucine	$C_6H_{13}NO_2$	131.094629	InChI=1S/C6H13NO2/c1-4(2)3-5(7)6(8)9/h4-5H,3,7H2,1-2H3,(H,8,9)/t5-/m0/s1	CC(C)CC(C(=O)O)N	61-90-5	61-90-5	CHEMBL291962	Fruits, pomace, and leaves	[206, 207]	5/ Not explicitly identified or discussed	Level 5 ★☆☆☆☆	5/ [209]
3	Valine	$C_5H_{11}NO_2$	117.078979	InChI=1S/C5H11NO2/c1-3(2)4(6)5(7)8/h3-4H,6H2,1-2H3,(H,7,8)/t4-/m0/s1	CC(C)C(C(=O)O)N	6287	72-18-4	CHEMBL43068	Fruits and pomace	[206, 207]	5/ Not explicitly identified or discussed	Level 5 ★☆☆☆☆	5/ [208]<br>5/ [209]
4	Aspartic acid	$C_4H_7NO_4$	133.037509	InChI=1S/C4H7NO4/c5-2(4(8)9)1-3(6)7/h2H,1,5H2,(H,6,7)(H,8,9)/t2-/m0/s1	C(C(C(=O)O)N)C(=O)O	5960	56-84-8	CHEMBL274323	Fruits and pomace	[145, 206]	5/ Not explicitly identified or discussed	Level 5 ★☆☆☆☆	5/ [208]<br>5/ [209]
5	Glutamic acid	$C_5H_9NO_4$	147.053159	InChI=1S/C5H9NO4/c6-3(5(9)10)1-2-4(7)8/h3H,1-2,6H2,(H,7,8)(H,9,10)/t3-/m0/s1	C(CC(=O)O)C(C(=O)O)N	33032	56-86-0	CHEMBL575060	Fruits and pomace	[145, 206]	5/ Not explicitly identified or discussed	Level 5 ★☆☆☆☆	5/ [208]<br>5/ [209]

b. Saturated hydrocarbons

No.	Compound name	Molecular formula	Exact mass	InChI	SMILES	CID	CAS Number	ChEMBL ID	Part used	References related to the part used	Identification Confidence*	Level	References related to confidence level
1	3,5-Dimethylheptane	C_9H_{20}	128.1565	InChI=1S/C9H20/c1-5-8(3)7-9(4)6-2/h8-9H,5-7H2,1-4H3	CCC(C)CC(C)CC	13558	926-82-9	CHEBI:229285	Extra virgin olive oil	[155]	4/ Tentative identification via spectral libraries only (GC-IT-MS with NIST05 library matching); no chromatographic validation.	Level 4 ★★☆☆☆	4/ [155]

No.	Compound name	Molecular formula	Exact mass	InChI	SMILES	CID	CAS Number	ChEMBL ID	Part used	References related to the part used	Identification Confidence*	Level 1 ★★★★★<br>Level 2 ★★★★☆<br>Level 3 ★★★☆☆<br>Level 4 ★★☆☆☆<br>Level 5 ★☆☆☆☆	References related to confidence level
**2**	Octane	C_8H_{18}	114.14085	InChI=1S/C8H18/c1-3-5-7-8-6-4-2/h3-8H2,1-2H3	CCCCCCCC	356	111-65-9	CHEMBL134886	Olive callus, and olive oil (EVOO)	[83, 183]	3/ Identification via GC-IT-MS and LRI with comparison of mass spectra to NIST05 library and literature data.<br><br>3/ Identification via GC–MS (retention time, MS spectra) and comparison with theoretical m/z values and spectral libraries.	Level 3 ★★★☆☆	3/ [155]<br><br>3/ [184]
**3**	(Z)-2-Pentene	C_5H_{10}	70.07825	InChI=1S/C5H10/c1-3-5-4-2/h3,5H,4H2,1-2H3/b5-3-	CC/C=C\C	5326160	627-20-3	NF	Olive oil (EVOO)	[155]	4/ Identification via GC-IT-MS with comparison of mass spectra to NIST05 library and literature data.	Level 4 ★★☆☆☆	4/ [155]
**4**	1,3-Pentadiene	C_5H_8	68.0626	InChI=1S/C5H8/c1-3-5-4-2/h3-5H,1H2,2H3/b5-4+	C/C=C/C=C	62204	2004-70-8	NF	Olive oil (EVOO)	[155]	3/ Identification via GC-IT-MS and LRI with comparison of mass spectra to NIST05 library and literature data.	Level 3 ★★★☆☆	4/ [155]
**5**	3,7-decadiene	$C_{10}H_{18}$	138.14085	InChI=1S/C10H18/c1-3-5-7-9-10-8-6-4-2/h5-8H,3-4,9-10H2,1-2H3/b7-5+,8-6+	CC/C=C/CC/C=C/CC	6429663	72015-36-2	NF	Olive oil (EVOO)	[155]	3/ Identification via GC-IT-MS and LRI with comparison of mass spectra to NIST05 library and literature data.	Level 3 ★★★☆☆	4/ [155]
**c. Aromatic compounds**													
**1**	1,4-Dimethoxybenzene	$C_8H_{10}O_2$	138.06808	InChI=1S/C8H10O2/c1-9-7-3-5-8(10-2)6-4-7/h3-6H,1-2H3	COC1=CC=C(C=C1)OC	9016	150-78-7	CHEMBL1668604	Stems	[35]	4/ Identification based on comparison of Rt, linear retention indices relative to n-hydrocarbons, and computer matching against commercial [NIST 98, ADAMS] and homemade library mass spectra... Molecular weights confirmed by GC-CIMS.	Level 4 ★★☆☆☆	4/ [35]
**2**	Pentylfuran	$C_9H_{14}O$	138.104465	InChI=1S/C9H14O/c1-2-3-4-6-9-7-5-8-10-9/h5,7-8H,2-4,6H2,1H3	CCCCCC1=CC=CO1	19602	3777-69-3	CHEMBL3182720	Stems	[35]	4/ Identification based on comparison of Rt, linear retention indices relative to n-hydrocarbons, and computer matching against commercial [NIST 98, ADAMS] and homemade library mass spectra... Molecular weights confirmed by GC-CIMS.	Level 4 ★★☆☆☆	4/ [35]
**3**	2-Phenylethyl β-primeveroside	$C_{19}H_{28}O_{10}$	416.16825	InChI=1S/C19H28O10/c20-11-8-27-18(16(24)13(11)21)28-9-12-14(22)15(23)17(25)19(29-12)26-7-6-10-4-2-1-3-5-10/h1-5,11-25H,6-9H2	C1C(C(C(C(O1)OCC2C(C(C(C(O2)OCCC3=CC=CC=C3)O)O)O)O)O)O	14704521	129932-48-5	NF	Stems, leaves, fruits, and olive cells	[17, 62, 96, 127]	4/ Identification based only on literature comparison using LC-ESI-MS and LC-APCI-MS.	Level 4 ★★☆☆☆	4/ [85]
**d. Alcoholic compounds**													

No.	Compound name	Molecular formula	Exact mass	InChI	SMILES	CID	CAS Number	ChEMBL ID	Part used	References related to the part used	Identification Confidence*	Level 1 ★★★★★<br>Level 2 ★★★★☆<br>Level 3 ★★★☆☆<br>Level 4 ★★☆☆☆<br>Level 5 ★☆☆☆☆	References related to confidence level
**1**	3-Pentanol	$C_5H_{12}O$	88.088815	InChI=1S/C5H12O/c1-3-5(6)4-2/h5-6H,3-4H2,1-2H3	CCC(CC)O	11428	584-02-1	CHEMBL47100	Pomace and olive oil (EVOO)	83, 210	3/ Identification via GC-IT-MS and LRI with comparison of mass spectra to NIST05 library and literature data.<br><br>3/ Identification via GC–MS (retention time, MS spectra) and comparison with theoretical m/z values and spectral libraries.	Level 3 ★★★☆☆	3/ 155<br><br>3/ 184
**2**	Penten-1-ol	$C_5H_{10}O$	86.073165	InChI=1S/C5H10O/c1-2-3-4-5-6/h3-4,6H,2,5H2,1H3/b4-3-	CCC=CCO	5364919	1576-95-0	CHEMBL2269088	Pomace and olive oil (EVOO)	57, 210	3/ Identification via GC–MS (retention time, MS spectra) and comparison with theoretical m/z values and spectral libraries.	Level 2 ★★★★☆	2/ 184
**3**	3-Methyl-1-butanol	$C_5H_{12}O$	88.088815	InChI=1S/C5H12O/c1-5(2)3-4-6/h5-6H,3-4H2,1-2H3	CC(C)CCO	31260	123-51-3	CHEMBL372396	Pomace and olive oil (EVOO)	210, 211	2/ Identification via GC-IT-MS with HS-SPME matched to the NIST05 mass spectral library (Rt+MS), confirmed via pure standard.	Level 2 ★★★★☆	2/ 155
**4**	Hexan-1-ol	$C_6H_{14}O$	102.104465	InChI=1S/C6H14O/c1-2-3-4-5-6-7/h7H,2-6H2,1H3	CCCCCCO	8103	111-27-3	CHEMBL14085	Stems and olive oil (EVOO)	57, 192	2/ Identification via GC-IT-MS with HS-SPME and LRI matched to the NIST05 mass spectral library (Rt+MS), confirmed via pure standard.<br><br>4/ Identification based on comparison of Rt, linear retention indices relative to n-hydrocarbons, and computer matching against commercial [NIST 98, ADAMS] and homemade library mass spectra... Molecular weights confirmed by GC-CIMS.	Level 2 ★★★★☆<br><br>Level 4 ★★☆☆☆	2/ 155<br><br>4/ 35
**5**	3-Hexen-1-ol	$C_6H_{12}O$	100.088815	InChI=1S/C6H12O/c1-2-3-4-5-6-7/h3-4,7H,2,5-6H2,1H3/b4-3+	CC/C=C/CCO	5284503	928-97-2	CHEMBL3184538	Pomace and olive oil (EVOO)	155	3/ Identification via GC-IT-MS and LRI with comparison of mass spectra to NIST05 library and literature data.	Level 3 ★★★☆☆	3/ 155

No.	Compound name	Molecular formula	Exact mass	InChI	SMILES	CID	CAS Number	ChEMBL ID	Part used	References related to the part used	Identification Confidence*	Level 1 ★★★★★<br>Level 2 ★★★★☆<br>Level 3 ★★★☆☆<br>Level 4 ★★☆☆☆<br>Level 5 ★☆☆☆☆	References related to confidence level
**6**	Octan-1-ol	$C_8H_{18}O$	130.135765	InChI=1S/C8H18O/c1-2-3-4-5-6-7-8-9/h9H,2-8H2,1H3	CCCCCCCCO	957	111-87-5	CHEMBL26215	Olive oil (EVOO)	[155]	2/ Identification via GC-IT-MS with HS-SPME and LRI matched to the NIST05 mass spectral library (Rt+MS), confirmed via pure standard.<br><br>4/ Identification based on comparison of Rt, linear retention indices relative to n-hydrocarbons, and computer matching against commercial [NIST 98, ADAMS] and homemade library mass spectra... Molecular weights confirmed by GC-CIMS.	Level 2 ★★★★☆<br><br>Level 4 ★★☆☆☆	2/ [155]<br><br>4/ [35]
**7**	Benzyl alcohol	C_7H_8O	108.057515	InChI=1S/C7H8O/c8-6-7-4-2-1-3-5-7/h1-5,8H,6H2	C1=CC=C(C=C1)CO	244	100-51-6	CHEMBL720	Stems, pomace, and olive oil (EVOO)	[35, 210, 211]	2/ Identification via GC-MS/MS (Rt + MS/MS) and direct comparison with authentic standard (Sigma-Aldrich).<br><br>4/ Identification based on comparison of Rt, linear retention indices relative to n-hydrocarbons, and computer matching against commercial [NIST 98, ADAMS] and homemade library mass spectra... Molecular weights confirmed by GC-CIMS.	Level 4 ★★☆☆☆	2/ [184]<br><br>4/ [35]
**8**	Phenylethyl alcohol	$C_8H_{10}O$	122.073165	InChI=1S/C8H10O/c9-7-6-8-4-2-1-3-5-8/h1-5,9H,6-7H2	C1=CC=C(C=C1)CCO	6054	60-12-8	CHEMBL448500	Stems, pomace, and olive oil (EVOO)	[35, 57, 210]	4/ Identification based on comparison of Rt, linear retention indices relative to n-hydrocarbons, and computer matching against commercial [NIST 98, ADAMS] and homemade library mass spectra... Molecular weights confirmed by GC-CIMS.	Level 4 ★★☆☆☆	4/ [35]
**e. Ester compounds**													
**1**	Ethyl acetate	$C_4H_8O_2$	88.05243	InChI=1S/C4H8O2/c1-3-6-4(2)5/h3H2,1-2H3	CCOC(=O)C	8857	141-78-6	CHEMBL14152	Pomace, olive oil (EVOO), and olive callus	[83, 183, 210]	2/ Identification via GC-MS/MS (Rt + MS/MS) and direct comparison with external standards (Sigma-Aldrich reference compounds).	Level 2 ★★★★☆	2/ [184]

No.	Compound name	Molecular formula	Exact mass	InChI	SMILES	CID	CAS Number	ChEMBL ID	Part used	References related to the part used	Identification Confidence*	Level 1 ★★★★★<br>Level 2 ★★★★☆<br>Level 3 ★★★☆☆<br>Level 4 ★★☆☆☆<br>Level 5 ★☆☆☆☆	References related to confidence level
**2**	Ethyl hexanoate	$C_8H_{16}O_2$	144.11503	InChI=1S/C8H16O2/c1-3-5-6-7-8(9)10-4-2/h3-7H2,1-2H3	CCCCCC(=O)OCC	31265	123-66-0	NF	Olive oil (EVOO)	[155]	2/ Identification via GC-IT-MS with HS-SPME and LRI matched to the NIST05 mass spectral library (Rt+MS), confirmed via pure standard.<br>4/ Identification via HS-SPME-GC–MS + RI (NIST) + MS library matched to databases.	Level 2 ★★★★☆<br>Level 4 ★★☆☆☆	2/ [155]<br>4/ [184]
**3**	Hexyl acetate	$C_8H_{16}O_2$	144.11503	InChI=1S/C8H16O2/c1-3-4-5-6-7-10-8(2)9/h3-7H2,1-2H3	CCCCCCOC(=O)C	8908	142-92-7	NF	Pomace, olive oil (EVOO), and olive callus	[83, 183, 210]	2/ Identification via GC-IT-MS with HS-SPME and LRI matched to the NIST05 mass spectral library (Rt+MS), confirmed via pure standard.<br>2/ Identification via GC-MS/MS (Rt + MS/MS) and direct comparison with external standards (Sigma-Aldrich reference compounds).	Level 2 ★★★★☆	2/ [155]<br>2/ [184]
**4**	(Z)-3-Hexen-1-yl acetate	$C_8H_{14}O_2$	142.09938	InChI=1S/C8H14O2/c1-3-4-5-6-7-10-8(2)9/h4-5H,3,6-7H2,1-2H3/b5-4-	CCC=CCCOC(=O)C	5363388	3681-71-8	CHEMBL2251454	Pomace, olive oil (EVOO), and olive callus	[155, 184]	2/ Identification via GC-IT-MS with HS-SPME and LRI matched to the NIST05 mass spectral library (Rt+MS), confirmed via pure standard.<br>2/ Identification via GC–MS (Rt + MS/MS) with external standards and direct comparison with Authentic standard (Z)-3-hexenyl acetate.	Level 2 ★★★★☆	2/ [155]<br>2/ [184]
**5**	Ethyl benzoate	$C_9H_{10}O_2$	150.06808	InChI=1S/C9H10O2/c1-2-11-9(10)8-6-4-3-5-7-8/h3-7H,2H2,1H3	CCOC(=O)C1=CC=CC=C1	7165	93-89-0	CHEMBL510714	Olive oil (EVOO)	[184]	4/ Identification via HS-SPME-GC–MS + RI (NIST) + MS library matched to databases.	Level 4 ★★☆☆☆	4/ [184]
**7**	Methyl salicylate	$C_8H_8O_3$	152.047345	InChI=1S/C8H8O3/c1-11-8(10)6-4-2-3-5-7(6)9/h2-5,9H,1H3	COC(=O)C1=CC=CC=C1O	4133	119-36-8	CHEMBL108545	Stems and olive oil (EVOO)	[35, 83]	3/ Identification based on comparison of Rt, linear retention indices relative to n-hydrocarbons, and computer matching against commercial [NIST 98, ADAMS] and homemade library mass spectra... Molecular weights confirmed by GC-CIMS.	Level 3 ★★★☆☆	3/ [35]
**8**	Methyl gallate	$C_8H_8O_5$	184.037175	InChI=1S/C8H8O5/c1-13-8(12)4-2-5(9)7(11)6(10)3-4/h2-3,9-11H,1H3	COC(=O)C1=CC(=C(C(=C1)O)O)O	7428	99-24-1	CHEMBL65675	Leaves	[212]	4/ Identification was based exclusively on matching retention times (Rt) with authentic chemical standards using HPLC-UV	Level 4 ★★☆☆☆	4/ [212]

No.	Compound name	Molecular formula	Exact mass	InChI	SMILES	CID	CAS Number	ChEMBL ID	Part used	References related to the part used	Identification Confidence*	Level 1 ★★★★★<br>Level 2 ★★★★☆<br>Level 3 ★★★☆☆<br>Level 4 ★★☆☆☆<br>Level 5 ★☆☆☆☆	References related to confidence level
**9**	Ethyl gallate	$C_9H_{10}O_5$	198.052824	InChI=1S/C9H10O5/c1-2-14-9(13)5-3-6(10)8(12)7(11)4-5/h3-4,10-12H,2H2,1H3	CCOC(=O)C1=CC(=C(C(=C1)O)O)O	13250	831-61-8	CHEMBL453196	Leaves	73	3/ Identification relies on Chromatographic behavior (Rt), UV spectrum, and MS/MS fragmentation pattern using LC-ESI-QTOF-MS, with Literature comparison.	Level 3 ★★★☆☆	3/ 73
**f. Other organic acids**													
**1**	Acetic acid	$C_2H_4O_2$	60.02113	InChI=1S/C2H4O2/c1-2(3)4/h1H3,(H,3,4)	CC(=O)O	176	64-19-7	CHEMBL539	Pomace, olive oil (EVOO), and wood	12, 210, 211	2/ Identification via GC-IT-MS with HS-SPME and LRI matched to the NIST05 mass spectral library (Rt+MS), confirmed via pure standard.	Level 2 ★★★★☆	2/ 155
**2**	Malic acid	$C_4H_6O_5$	134.021525	InChI=1S/C4H6O5/c5-2(4(8)9)1-3(6)7/h2,5H,1H2,(H,6,7)(H,8,9)	C(C(C(=O)O)O)C(=O)O	525	6915-15-7	CHEMBL1455497	Roots, flowers, leaves, pomace, OLS, and wood	1, 2, 9, 43	2/ combined NMR (chemical shifts, 2D-NMR support) and MS spectral data HRMS ($[M-H]^-$ and $[M-H-H_2O]^-$ fragments). The Chemonix database is used.	Level 2 ★★★★☆	2/ 6
**3**	Succinic acid	$C_4H_6O_4$	118.02661	InChI=1S/C4H6O4/c5-3(6)1-2-4(7)8/h1-2H2,(H,5,6)(H,7,8)	C(CC(=O)O)C(=O)O	1110	110-15-6	CHEMBL576	Olive oil, fruits, seeds, leaves, and branches	47	4/ Analyzed using RP/HPLC-Q-TOF MS/MS analysis, and the identification based on precursor ion mass, Rt, and MS/MS fragmentation pattern matched against spectral libraries (NIST20, LipidBlast, MoNA)	Level 4 ★★☆☆☆	4/ 64
**4**	Citric acid	$C_6H_8O_7$	192.027005	InChI=1S/C6H8O7/c7-3(8)1-6(13,5(11)12)2-4(9)10/h13H,1-2H2,(H,7,8)(H,9,10)(H,11,12)	C(C(=O)O)C(CC(=O)O)(C(=O)O)O	311	77-92-9	CHEMBL1261	Roots, flowers, wood, fruits, pomace, and OLS	1, 2, 9, 43, 127	3/ Identification via HPLC-DAD and LC-ESI-QTOF-MS/MS, relies on chromatographic alignment (Rt) and spectrometric data (HRMS/MSMS) compared to literature data.	Level 3 ★★★☆☆	3/ 1
**5**	Quinic acid	$C_7H_{12}O_6$	192.06339	InChI=1S/C7H12O6/c8-3-1-7(13,6(11)12)2-4(9)5(3)10/h3-5,8-10,13H,1-2H2,(H,11,12)/t3-,4-,5?,7?/m1/s1	C1C(C(C(CC1(C(=O)O)O)O)O)O	6508	77-95-2	CHEMBL465398	Flowers, fruits, pomace, leaves, stems, roots, wood, OOMW, and OLS	9, 96, 213	4/ Identification based only on literature comparison using LC-ESI-MS and LC-APCI-MS.	Level 4 ★★☆☆☆	4/ 85
**6**	Shikimic acid	$C_7H_{10}O_5$	174.052824	InChI=1S/C7H10O5/c8-4-1-3(7(11)12)2-5(9)6(4)10/h1,4-6,8-10H,2H2,(H,11,12)/t4-,5-,6-/m1/s1	C1C(C(C(C=C1C(=O)O)O)O)O	8742	138-59-0	CHEMBL290345	Pomace and olive oil	25	4/ The detection based on 1H NMR matched to the literature.	Level 4 ★★☆☆☆	4/ 68
**7**	Ascorbic acid (Vit. C)	$C_6H_8O_6$	176.03209	InChI=1S/C6H8O6/c7-1-2(8)5-3(9)4(10)6(11)12-5/h2,5,7-10H,1H2/t2-,5+/m0/s1	C(C(C1C(=C(C(=O)O1)O)O)O)O	54670067	50-81-7	CHEMBL196	Leaves	94	2/ Identification via HPLC-DAD (Rt, UV spectrum) and matched to authentic standard.	Level 2 ★★★★☆	2/ 94

No.	Compound name	Molecular formula	Exact mass	InChI	SMILES	CID	CAS Number	ChEMBL ID	Part used	References related to the part used	Identification Confidence*	Level 1 ★★★★★<br>Level 2 ★★★★☆<br>Level 3 ★★★☆☆<br>Level 4 ★★☆☆☆<br>Level 5 ★☆☆☆☆	References related to confidence level
**8**	Ascorbyl hexoside (glucopyranosyl-L-ascorbic acid)	$C_{12}H_{18}O_{11}$	338.084915	InChI=1S/C12H18O11/c13-1-3(15)9-8(19)10(11(20)22-9)23-12-7(18)6(17)5(16)4(2-14)21-12/h3-7,9,12-19H,1-2H2/t3-,4+,5+,6-,7+,9+,12+/m0/s1	C(C1C(C(C(C(O1)OC2=C(C(OC2=O)C(CO)O)O)O)O)O)O	54693473	129499-78-1	CHEMBL2011961	Leaves, wood, and OLS	[2]	3/ Identification relied on mass spectrometry and MS/MS fragments using RP-HPLC-DAD-ESI-QTOF-MS/MS and UV-Vis data, with literature comparison.	Level 3 ★★★☆☆	3/ [2]

***Confidence levels were assigned based on the identification criteria described in cited works, and categorized as follows:** 1/ **Level 1:** Full isolation and structure elucidation including 2D NMR; 2/ **Level 2:** Co-chromatography with authentic standard (Rt + UV/HRMS/MS/MS/HNMR); 3/ **Level 3:** Chromatography with spectral data matched to literature (Rt + UV/HRMS/MS/MS/HNMR); 4/ **Level 4:** Chromatography data only (e.g., Rt + UV or MS); and 5/ **Level 5:** No explicit identification data reported.

OLS: olive leaves with stems; OOMW: olive oil mill wastewater; VOO: virgin olive oil; EVOO: extra virgin olive oil; EA: elenolic acid; EDA: elenolic acid decarboxymethylated; FAB-MS: fast atom bombardment-mass spectrometry; TLC: thin layer chromatography; PTLC: preparative thin layer chromatography; HPLC: high performance liquid chromatography; PHPLC: preparative High performance liquid chromatography; NMR: nuclear magnetic resonance; COSY: correlated spectroscopy; HSQC: heteronuclear single quantum correlation; HMBC: heteronuclear multiple bond correlation; UV: ultraviolet; SPE: solid phase extraction; DAD: diode-array detector; ESI: electrospray ionization; ECD: electrochemical detection; FTIR: fourier transform infrared spectroscopy; APCI-MS: atmospheric pressure chemical ionization-mass spectrometry; GC–CIMS: gas chromatography–chemical ionization mass spectrometry; MALDI: matrix-assisted laser desorption/ionization; GC-IT-MS: gas chromatography-ion trap mass spectrometry; HS-SPME: Headspace solid-phase microextraction; QqQ: triple-quadrupole; LRI: linear retention index; FID: flame ionization detector; FAME: fatty acid methyl ester; IOOC: International Olive Oil Council; EC: European Community regulation; ND: not found.

1. K. Toumi, L. Swiatek, A. Boguszewska, K. Skalicka-Wozniak and M. Bouaziz, *Molecules*, 2023, **28**, 4829.
2. S. Ammar, M. D. M. Contreras, B. Gargouri, A. Segura-Carretero and M. Bouaziz, *Phytochem. Anal*, 2017, **28**, 217-229.
3. S. Gomez-Gonzalez, J. Ruiz-Jimenez, F. Priego-Capote and M. D. Luque de Castro, *J. Agric. Food Chem.*, 2010, **58**, 12292-12299.
4. M. Wodner, S. Lavee and E. Epstein, *Scientia Horticulturae*, 1988, **36**, 47-54.
5. T. Aree and S. Jongrungruangchok, *Carbohydr. Polym.*, 2018, **199**, 661-669.
6. A. Jlilat, R. Ragone, S. Gualano, F. Santoro, V. Gallo, L. Varvaro, P. Mastrorilli, M. Saponari, F. Nigro and A. M. D'Onghia, *Sci. Rep.*, 2021, **11**, 1070.
7. B. Mechri, M. Tekaya, H. Cheheb and M. Hammami, *J. Chromatogr. Sci.*, 2015, **53**, 1631-1638.
8. R. Abbattista, I. Losito, C. D. Calvano and T. R. I. Cataldi, *Foods*, 2021, **10**, 2050.
9. A. Angelis, P. Mavros, P. E. Nikolaou, S. Mitakou, M. Halabalaki and L. Skaltsounis, *Fitoterapia*, 2020, **143**, 104602.
10. R. Guodong, L. Xiaoxia, Z. Weiwei, W. Wenjun and Z. Jianguo, *Biology Open*, 2017, **6**, 1317-1323.
11. R. Quirantes-Piné, J. Lozano-Sánchez, M. Herrero, E. Ibáñez, A. Segura-Carretero and A. Fernández-Gutiérrez, *Phytochem. Anal*, 2013, **24**, 213-223.
12. V. Lo Giudice, I. Faraone, M. R. Bruno, M. Ponticelli, F. Labanca, D. Bisaccia, C. Massarelli, L. Milella and L. Todaro, *Molecules*, 2021, **26**, 5081.
13. A. Lama-Muñoz, M. del Mar Contreras, F. Espínola, M. Moya, I. Romero and E. Castro, *Food Chem.*, 2020, **329**, 127153.
14. M. Patumi, G. Fontanazza, L. Baldoni and I. Brambilla.
15. G. Mosleh, A. Mohagheghzadeh and P. Faridi, *Trends in Pharmaceutical Sciences*, 2016, **2**, 241-252.
16. E. Campeol, G. Flamini, P. L. Cioni, I. Morelli, F. D'Andrea and R. Cremonini, *Carbohydr. Res.*, 2004, **339**, 2731-2732.
17. G. Difonzo, M. A. Crescenzi, S. Piacente, G. Altamura, F. Caponio and P. Montoro, *Plants (Basel)*, 2022, **11**, 3321.
18. C. Conde, S. Delrot and H. Geros, *J. Plant Physiol.*, 2008, **165**, 1545-1562.
19. L. L. Flora and M. A. Madore, *Planta*, 1993, **189**, 484-490.
20. P. Luaces, R. Sanchez, J. Exposito, A. J. Perez-Pulido, A. G. Perez and C. Sanz, *Int. J. Mol. Sci.*, 2024, **25**, 10892.
21. Z. Zhu, X. Li, Y. Zhang, J. Wang, F. Dai and W. Wang, *Lwt*, 2023, **174**, 114424.
22. Á. Fernández-Prior, A. Bermúdez-Oria, J. Fernández-Bolaños, J. A. Espejo-Calvo, F. López-Maestro and G. Rodríguez-Gutiérrez, *Molecules*, 2022, **27**, 8380.
23. J. M. Silvan, E. Guerrero-Hurtado, A. Gutierrez-Docio, T. Alarcon-Cavero, M. Prodanov and A. J. Martinez-Rodriguez, *Antioxidants (Basel)*, 2021, **10**, 2030.
24. S. Salido, M. Perez-Bonilla, R. P. Adams and J. Altarejos, *J. Agric. Food Chem.*, 2015, **63**, 6493-6500.
25. A. Peralbo-Molina, F. Priego-Capote and M. D. Luque de Castro, *J. Agric. Food Chem.*, 2012, **60**, 11542-11550.
26. G. Rodríguez, A. Lama, R. Rodríguez, A. Jiménez, R. Guillén and J. Fernández-Bolaños, *Bioresour. Technol.*, 2008, **99**, 5261-5269.
27. R. Japon-Lujan and M. D. Luque de Castro, *J. Agric. Food Chem.*, 2007, **55**, 4584-4588.

28. S. Christophoridou, P. Dais, L.-H. Tseng and M. Spraul, *J. Agric. Food Chem.*, 2005, **53**, 4667-4679.
29. R. Limiroli, R. Consonni, A. Ranalli, G. Bianchi and L. Zetta, *J. Agric. Food Chem.*, 1996, **44**, 2040-2048.
30. H. Zhao, R. J. Avena-Bustillos and S. C. Wang, *Foods*, 2022, **11**, 174.
31. N. Talhaoui, A. M. Gómez-Caravaca, L. León, R. De la Rosa, A. Segura-Carretero and A. Fernández-Gutiérrez, *LWT-Food Science and Technology*, 2014, **58**, 28-34.
32. H. K. Obied, D. R. Bedgood Jr, P. D. Prenzler and K. Robards, *Anal. Chim. Acta*, 2007, **603**, 176-189.
33. M. A. Hashmi, A. Khan, M. Hanif, U. Farooq and S. Perveen, *Evid. Based Complement. Alternat. Med.*, 2015, **2015**, 541591.
34. G. Tóth, Á. Alberti, A. Sólyomváry, C. Barabás, I. Boldizsár and B. Noszál, *Industrial Crops and Products*, 2015, **67**, 432-438.
35. F. Brahmi, B. Mechri, G. Flamini, M. Dhibi and M. Hammami, *Acta Physiologiae Plantarum*, 2012, **35**, 1061-1070.
36. M. DellaGreca, L. Previtera, F. Temussi and A. Zarrelli, *Phytochemical Analysis: An International Journal of Plant Chemical and Biochemical Techniques*, 2004, **15**, 184-188.
37. C. S. Papageorgiou, P. Lyri, I. Xintaropoulou, I. Diamantopoulos, D. P. Zagklis and C. A. Paraskeva, *Antioxidants (Basel)*, 2022, **11**, 1042.
38. D. Maestri, D. Barrionuevo, R. Bodoira, A. Zafra, J. Jimenez-Lopez and J. D. Alche, *J. Food Sci. Technol.*, 2019, **56**, 4359-4370.
39. E. Melliou, J. A. Zweigenbaum and A. E. Mitchell, *J. Agric. Food Chem.*, 2015, **63**, 2400-2405.
40. T. Michel, I. Khlif, P. Kanakis, A. Termentzi, N. Allouche, M. Halabalaki and A.-L. Skaltsounis, *Phytochemistry letters*, 2015, **11**, 424-439.
41. C. Romero, M. Brenes, P. García and A. Garrido, *J. Agric. Food Chem.*, 2002, **50**, 3835-3839.
42. D. Maestri, D. Barrionuevo, R. Bodoira, A. Zafra, J. Jiménez-López and J. d. D. Alché, *J. Food Sci. Technol.*, 2019, **56**, 4359-4370.
43. C. M. Sánchez-Arévalo, F. Aldegheri, M. C. Vincent-Vela and S. Álvarez-Blanco, *Int. J. Mol. Sci.*, 2024, **25**, 5233.
44. A. Ghorbel, S. Wedel, I. Kallel, M. Cavinato, M. E. Sakavitsi, J. Fakhfakh, M. Halabalaki, P. Jansen-Dürr and N. Allouche, *Journal of Food Measurement and Characterization*, 2021, **15**, 4946-4959.
45. M. Pérez-Trujillo, A. M. Gómez-Caravaca, A. Segura-Carretero, A. Fernández-Gutiérrez and T. Parella, *J. Agric. Food Chem.*, 2010, **58**, 9129-9136.
46. A. Bianco, M. A. Chiacchio, G. Grassi, D. Iannazzo, A. Piperno and R. Romeo, *Food Chem.*, 2006, **95**, 562-565.
47. N. H. Bahtiti, F. M. A. Orabi, M. H. Kailani, I. Abdel-Rahman, A. Nahlé, Z. O. Alfaouri and H. H. Al Abdallat, *WSEAS Transactions on Environment and Development*, 2023, **19**, 903-916.
48. B. Zghari, P. Doumenq, A. Romane and A. Boukir, *J. Mater. Environ. Sci*, 2017, **8**, 4496-4509.
49. A. E. Derardja, M. Pretzler, M. Barkat and A. Rompel, *J. Agric. Food Chem.*, 2024, **72**, 3099-3112.
50. A.-L. Skaltsounis, A. Argyropoulou, N. Aligiannis and N. Xynos, in *Olive and Olive Oil Bioactive Constituents*, Elsevier, 2015, DOI: 10.1016/b978-1-63067-041-2.50017-3, pp. 333-356.
51. A. Toledano, L. Serrano and J. Labidi, *Journal of the Taiwan Institute of Chemical Engineers*, 2013, **44**, 552-559.
52. M. D. Rodriguez-Pérez, L. Santiago-Corral, L. Ortega-Hombrados, C. Verdugo, M. M. Arrebola, E. Martín-Aurioles, M. Á. Fernández-Prior, A. Bermúdez-Oria, J. P. De La Cruz and J. A. González-Correa, *Nutrients*, 2023, **15**, 377.
53. A. Bermúdez-Oria, G. Rodríguez-Gutiérrez, F. Rubio-Senent, A. Lama-Muñoz and J. Fernández-Bolaños, *Carbohydr. Polym.*, 2017, **163**, 292-300.
54. A. Lama-Muñoz, G. Rodríguez-Gutiérrez, F. Rubio-Senent, R. Palacios-Díaz and J. Fernández-Bolaños, *Food Chem.*, 2013, **140**, 154-160.
55. G. Rodríguez, A. Lama, M. Trujillo, J. L. Espartero and J. Fernández-Bolaños, *LWT-Food Science and Technology*, 2009, **42**, 483-490.
56. U. Vijakumaran, N. Y. Goh, R. A. Razali, N. A. H. Abdullah, M. D. Yazid and N. Sulaiman, *Antioxidants (Basel)*, 2023, **12**, 1140.
57. P. Masella, L. Guerrini, G. Angeloni, A. Spadi, F. Baldi and A. Parenti, *International Journal of Refrigeration*, 2019, **106**, 24-32.
58. S. Dermeche, M. Nadour, C. Larroche, F. Moulti-Mati and P. Michaud, *Process Biochem.*, 2013, **48**, 1532-1552.
59. A. A. Deeb, M. K. Fayyad and M. A. Alawi, *Journal of Chromatography Research International*, 2012, **2012**.
60. A. Nunes, J. Marto, L. Gonçalves, A. M. Martins, C. Fraga and H. M. Ribeiro, *Int. J. Food Sci. Technol.*, 2021, **57**, 173-187.
61. M. Bouaziz, M. Chamkha and S. Sayadi, *J. Agric. Food Chem.*, 2004, **52**, 5476-5481.
62. C. Jimenez-Sanchez, M. Olivares-Vicente, C. Rodriguez-Perez, M. Herranz-Lopez, J. Lozano-Sanchez, A. Segura-Carretero, A. Fernandez-Gutierrez, J. A. Encinar and V. Micol, *PLoS One*, 2017, **12**, e0173074.
63. A. D. Marrero, A. R. Quesada, B. Martinez-Poveda and M. A. Medina, *Nutrients*, 2024, **16**, 1283.
64. C. Dauber, T. Carreras, L. González, A. Gámbaro, A. Valdés, E. Ibañez and I. Vieitez, *Lwt*, 2022, **160**, 113274.
65. T. Ivancic, J. Jakopic, R. Veberic, V. Vesel and M. Hudina, *Agriculture*, 2022, **12**, 1347.
66. M. Vergine, S. Pavan, C. Negro, F. Nicolì, D. Greco, E. Sabella, A. Aprile, L. Ricciardi, L. De Bellis and A. Luvisi, *Journal of Plant Interactions*, 2022, **17**, 462-474.
67. A. Malapert, E. Reboul, M. Loonis, O. Dangles and V. Tomao, *Food Analytical Methods*, 2018, **11**, 1001-1010.
68. S. Christophoridou and P. Dais, *Anal. Chim. Acta*, 2009, **633**, 283-292.
69. I. Ben-Amor, M. Musarra-Pizzo, A. Smeriglio, M. D'Arrigo, R. Pennisi, H. Attia, B. Gargouri, D. Trombetta, G. Mandalari and M. T. Sciortino, *Viruses*, 2021, **13**, 1085.
70. R. Jurisic Grubesic, M. Nazlic, T. Miletic, E. Vuko, N. Vuletic, I. Ljubenkov and V. Dunkic, *Antioxidants (Basel)*, 2021, **10**, 1832.
71. I. Ben-Amor, M. Musarra-Pizzo, A. Smeriglio, M. D’Arrigo, R. Pennisi, H. Attia, B. Gargouri, D. Trombetta, G. Mandalari and M. T. Sciortino, *Viruses*, 2021, **13**, 1085.
72. H. A. Zahran and H. M. Soliman, *Curr Sci Int*, 2018, **7**, 307-319.
73. E. M. Kabbash, Z. T. Abdel-Shakour, S. H. El-Ahmady, M. Wink and I. M. Ayoub, *Sci. Rep.*, 2023, **13**, 612.
74. F. Alagna, R. Mariotti, F. Panara, S. Caporali, S. Urbani, G. Veneziani, S. Esposto, A. Taticchi, A. Rosati, R. Rao, G. Perrotta, M. Servili and L. Baldoni, *BMC Plant Biol.*, 2012, **12**, 162.
75. A. Esposito, P. F. De Luca, V. Graziani, B. D’Abrosca, A. Fiorentino and M. Scognamiglio, *Molecules*, 2021, **26**, 3845.
76. A. Ghorbel, S. Wedel, I. Kallel, M. Cavinato, M. E. Sakavitsi, J. Fakhfakh, M. Halabalaki, P. Jansen-Dürr and N. Allouche, *Journal of Food Measurement and Characterization*, 2021, **15**, 4946-4959.
77. P. Xie, L. Cecchi, M. Bellumori, D. Balli, L. Giovannelli, L. Huang and N. Mulinacci, *Foods*, 2021, **10**, 2823.
78. C. Zhang, J. Zhang, X. Xin, S. Zhu, E. Niu, Q. Wu, T. Li and D. Liu, *Front Nutr*, 2022, **9**, 854680.
79. C. Zhang, X. Xin, J. Zhang, S. Zhu, E. Niu, Z. Zhou and D. Liu, *Molecules*, 2022, **27**, 1292.
80. E. Kritikou, N. P. Kalogiouri, L. Kolyvira and N. S. Thomaidis, *Molecules*, 2020, **25**, 4889.
81. S. A. Rashed, T. I. Saad and S. M. El-Darier, *Rendiconti Lincei. Scienze Fisiche e Naturali*, 2022, **33**, 195-203.

82. M. Mattonai, A. Vinci, I. Degano, E. Ribechini, M. Franceschi and F. Modugno, *Natural Product Research*, 2019, **33**, 3171-3175.
83. F. Fratianni, R. Cozzolino, A. Martignetti, L. Malorni, A. d'Acierno, V. De Feo, A. G. da Cruz and F. Nazzaro, *Food Sci Nutr*, 2019, **7**, 3233-3243.
84. M. K. S. Morsi, S. M. Galal and O. Alabdulla, *J. Environ. Sci. Toxicol. Food Technol*, 2016, **10**, 95-100.
85. L. Olmo-García, N. Kessler, H. Neuweger, K. Wendt, J. M. Olmo-Peinado, A. Fernández-Gutiérrez, C. Baessmann and A. Carrasco-Pancorbo, *Molecules*, 2018, **23**, 2419.
86. F. He, Y. Du, Z. Pan, H. Zeng, H. Luo, J. Wang, Y. Sun and M. Li, *Frontiers in Nutrition*, 2024, **11**, 1334077.
87. A. V. Chatzikonstantinou, A. Giannakopoulou, S. Spyrou, Y. V. Simos, V. G. Kontogianni, D. Peschos, P. Katapodis, A. C. Polydera and H. Stamatis, *Environ. Sci. Pollut. Res. Int.*, 2022, **29**, 29624-29637.
88. D. Ryan, M. Antolovich, P. Prenzler, K. Robards and S. Lavee, *Scientia Horticulturae*, 2002, **92**, 147-176.
89. A. Agiomyrgianaki, P. V. Petrakis and P. Dais, *Food Chem.*, 2012, **135**, 2561-2568.
90. G. Boskou, in *Olives and olive oil in health and disease prevention*, Elsevier, 2010, pp. 925-934.
91. M. Kulak and H. Cetinkaya, *Polyphenols*, 2018, 3-20.
92. C. Mert, E. Barut and A. Ipek, 2013.
93. H. Elhrech, O. Aguerd, C. El Kourchi, M. Gallo, D. Naviglio, I. Chamkhi and A. Bouyahya, *Biomolecules*, 2024, **14**, 722.
94. S. Bensehaila, F. Ilias, F. Saadi and N. Zaouadi, *Asian Journal of Dairy and Food Research*, 2022, **41**, 237-241.
95. P. K. Revelou, S. J. Konteles, A. Batrinou, M. Xagoraris, P. A. Tarantilis and I. F. Strati, *Foods*, 2024, **13**, 3164.
96. I. Serrano-García, L. Olmo-García, O. Monago-Maraña, I. M. C. de Alba, L. León, R. de la Rosa, A. Serrano, A. M. Gómez-Caravaca and A. Carrasco-Pancorbo, *Antioxidants*, 2023, **12**, 2120.
97. A. Supriadin, H. Juliani and N. Tanyela.
98. M. J. Aliaño-González, J. Gabaston, V. Ortiz-Somovilla and E. Cantos-Villar, *Biomolecules*, 2022, **12**, 238.
99. P. Charisiadis, V. G. Kontogianni, C. G. Tsiafoulis, A. G. Tzakos and I. P. Gerothanassis, *Phytochem. Anal*, 2017, **28**, 159-170.
100. F. Rubio-Senent, S. Martos, A. Lama-Munoz, J. G. Fernandez-Bolanos, G. Rodriguez-Gutierrez and J. Fernandez-Bolanos, *Food Chem.*, 2015, **187**, 166-173.
101. R. Abbattista, G. Ventura, C. D. Calvano, T. R. I. Cataldi and I. Losito, *Foods*, 2021, **10**, 1236.
102. F. Rubio-Senent, S. Martos, A. Lama-Muñoz, J. G. Fernández-Bolaños, G. Rodríguez-Gutiérrez and J. Fernández-Bolaños, *Food Chem.*, 2015, **187**, 166-173.
103. A. Starzyńska-Janiszewska, C. Fernández-Fernández, B. Martín-García, V. Verardo and A. M. Gómez-Caravaca, *Antioxidants*, 2022, **11**, 1693.
104. B. Martin-Garcia, S. Pimentel-Moral, A. M. Gómez-Caravaca, D. Arráez-Román and A. Segura-Carretero, *Industrial Crops and Products*, 2020, **154**, 112741.
105. J. Milosevic, *Isolation and characterization of natural products from Olea europaea and some biological activities*, University of London, University College London (United Kingdom), 2000.
106. A. F. Vinha, F. Ferreres, B. M. Silva, P. Valentao, A. Gonçalves, J. A. Pereira, M. B. Oliveira, R. M. Seabra and P. B. Andrade, *Food Chem.*, 2005, **89**, 561-568.
107. H. Boutaj, *Diseases*, 2024, **12**, 246.
108. P. Katekar, D. Thakare, K. Singh, S. Pandey and M. Hasan.
109. G. Rigane, M. Bouaziz, S. Sayadi and R. Ben Salem, *Eur. Food Res. Technol.*, 2012, **234**, 1049-1054.
110. C. Lino, D. Bongiorno, R. Pitonzo, S. Indelicato, M. Barbera, G. Di Gregorio, D. Pane and G. Avellone, *Foods*, 2024, **13**, 2149.
111. G. Ventura, C. D. Calvano, R. Abbattista, M. Bianco, C. De Ceglie, I. Losito, F. Palmisano and T. R. I. Cataldi, *Rapid Commun. Mass Spectrom.*, 2019, **33**, 1670-1681.
112. A. Angelis, L. Antoniadi, P. Stathopoulos, M. Halabalaki and L. A. Skaltsounis, *Phytochemistry letters*, 2018, **26**, 190-194.
113. A. Bianco and N. Uccella, *Food Research International*, 2000, **33**, 475-485.
114. H. Nakayama, N. Nishi, Y. Matsuo, T. Tanaka, N. Kotoda and K. Ishimaru, *J. Asian Nat. Prod. Res.*, 2022, **24**, 1093-1100.
115. A. Karioti, A. Chatzopoulou, A. R. Bilia, G. Liakopoulos, S. Stavrianakou and H. Skaltsa, *Biosci. Biotechnol. Biochem.*, 2006, **70**, 1898-1903.
116. J. D. Sidda, L. Song, J. L. Parker, D. J. Studholme, C. Sambles and M. Grant, *Sci. Rep.*, 2020, **10**, 19566.
117. M. M. Solé, L. Pons, M. Conde, C. Gaidau and A. Bacardit, *Materials*, 2021, **14**, 5790.
118. M. Antonia Nunes, A. S. G. Costa, S. Bessada, J. Santos, H. Puga, R. C. Alves, V. Freitas and M. Oliveira, *Sci. Total Environ.*, 2018, **644**, 229-236.
119. M. Savarese, E. De Marco and R. Sacchi, *Food Chem.*, 2007, **105**, 761-770.
120. H. K. Obied, P. Karuso, P. D. Prenzler and K. Robards, *J. Agric. Food Chem.*, 2007, **55**, 2848-2853.
121. A. Spadi, G. Angeloni, L. Cecchi, F. Corti, D. Balli, L. Guerrini, L. Calamai, A. Parenti and P. Masella, *Waste and Biomass Valorization*, 2023, **14**, 1931-1944.
122. I. D'Antuono, A. Garbetta, B. Ciasca, V. Linsalata, F. Minervini, V. M. T. Lattanzio, A. F. Logrieco and A. Cardinali, *J. Agric. Food Chem.*, 2016, **64**, 5671-5678.
123. T. Jerman, P. Trebše and B. M. Vodopivec, *Food Chem.*, 2010, **123**, 175-182.
124. S. Fu, D. Arraez-Roman, A. Segura-Carretero, J. A. Menendez, M. P. Menendez-Gutierrez, V. Micol and A. Fernandez-Gutierrez, *Anal. Bioanal. Chem.*, 2010, **397**, 643-654.
125. L. Cecchi, V. Piazzini, M. D'Ambrosio, C. Luceri, F. Rocco, M. Innocenti, G. Vanti, N. Mulinacci and M. C. Bergonzi, *Molecules*, 2020, **25**, 3198.
126. X.-F. Wang, C. Li, Y.-P. Shi and D.-L. Di, *J. Asian Nat. Prod. Res.*, 2009, **11**, 940-944.
127. I. Serrano-García, L. Olmo-García, D. Polo-Megías, A. Serrano, L. León, R. de la Rosa, A. M. Gómez-Caravaca and A. Carrasco-Pancorbo, *Plants*, 2022, **11**, 1791.
128. A. Taamalli, D. Arraez-Roman, E. Ibanez, M. Zarrouk, A. Segura-Carretero and A. Fernandez-Gutierrez, *J. Agric. Food Chem.*, 2012, **60**, 791-798.
129. F. Rubio-Senent, A. Lama-Munoz, G. Rodriguez-Gutierrez and J. Fernandez-Bolanos, *J. Agric. Food Chem.*, 2013, **61**, 1235-1248.
130. E. Kritikou, N. P. Kalogiouri, M. Kostakis, D.-C. Kanakis, I. Martakos, C. Lazarou, M. Pentogennis and N. S. Thomaidis, *Foods*, 2021, **10**, 2102.
131. S. M. Cardoso, S. Guyot, N. Marnet, J. A. Lopes-da-Silva, A. M. S. Silva, C. M. G. C. Renard and M. A. Coimbra, *J. Sci. Food Agric.*, 2006, **86**, 1495-1502.
132. A. B. Smith, J. B. Sperry and Q. Han, *The Journal of organic chemistry*, 2007, **72**, 6891-6900.
133. D. Caruso, R. Colombo, R. Patelli, F. Giavarini and G. Galli, *J. Agric. Food Chem.*, 2000, **48**, 1182-1185.
134. M. Pérez-Bonilla, S. Salido, T. A. van Beek, P. d. Waard, P. J. Linares-Palomino, A. Sánchez and J. Altarejos, *Food Chem.*, 2011, **124**, 36-41.
135. T. Jerman Klen, A. Golc Wondra, U. Vrhovsek and B. Mozetič Vodopivec, *J. Agric. Food Chem.*, 2015, **63**, 3859-3872.
136. S. Silva, L. Gomes, F. Leitão, M. Bronze, A. V. Coelho and L. V. Boas, *Grasas y Aceites*, 2010, **61**, 157-164.

137. M. Servili, M. Baldioli, R. Selvaggini, A. Macchioni and G. Montedoro, *J. Agric. Food Chem.*, 1999, **47**, 12-18.
138. R. Maestro-Duran, R. L. Cabello, V. Ruiz-Gutierrez, P. Fiestas and A. Vazquez-Roncero, *Grasas y Aceites*, 1994, **45**, 332-335.
139. L. Cecchi, G. Ghizzani, M. Bellumori, C. Lammi, B. Zanoni and N. Mulinacci, *Molecules*, 2023, **28**, 2776.
140. T. Jerman, 2014.
141. D. Gabbia, *Biology*, 2024, **13**, 760.
142. N. Damak, N. Allouche, B. Hamdi, M. Litaudon and M. Damak, *Natural Product Research*, 2012, **26**, 125-131.
143. M. Tarapoulouzi, S. Agriopoulou, A. Koidis, C. Proestos, H. A. E. Enshasy and T. Varzakas, *Biomolecules*, 2022, **12**, 1180.
144. A. Bianco, F. Coccioli, M. Guiso and C. Marra, *Food Chem.*, 2002, **77**, 405-411.
145. F. Paiva-Martins and A. Kiritsakis, *Olives and Olive Oil as Functional Foods: Bioactivity, Chemistry and Processing*, 2017, 81-115.
146. M. Guiso, C. Marra and R. R. Arcos, *Nat Prod Res*, 2008, **22**, 1403-1409.
147. R. Ahmad, A. Alqathama, M. M. Alam, M. Riaz, A. N. Abdalla, M. Aldholmi, H. M. Al‒ Said, F. S. Aljishi, E. H. Althomali and M. M. Alabdullah, *Chemical and Biological Technologies in Agriculture*, 2023, **10**, 45.
148. E. Kadowaki, Y. Yoshida, T. Nitoda, N. Baba and S. Nakajima, *Biosci. Biotechnol. Biochem.*, 2003, **67**, 415-419.
149. C. Sarikurkcu, M. Locatelli, A. Tartaglia, V. Ferrone, A. M. Juszczak, M. S. Ozer, B. Tepe and M. Tomczyk, *Molecules*, 2020, **25**, 1202.
150. M. Brenes, F. J. Hidalgo, A. García, J. J. Rios, P. García, R. Zamora and A. Garrido, *Journal of the American Oil Chemists' Society*, 2000, **77**, 715-720.
151. H. Tsukamoto, S. Hisada and S. Nishibe, *Chem. Pharm. Bull. (Tokyo)*, 1984, **32**, 2730-2735.
152. B. Alaziqi, L. Beckitt, D. J. Townsend, J. Morgan, R. Price, A. Maerivoet, J. Madine, D. Rochester, G. Akien and D. A. Middleton, *ACS omega*, 2024, **9**, 32557-32578.
153. L. Abaza, A. Taamalli, H. Nsir and M. Zarrouk, *Antioxidants (Basel)*, 2015, **4**, 682-698.
154. T. Yangui, H. Chakroun, A. Dhouib and M. Bouaziz, *Journal of Essential Oil Bearing Plants*, 2022, **24**, 1389-1401.
155. I. Lukić, M. Lukić, M. Žanetić, M. Krapac, S. Godena and K. Brkić Bubola, *Foods*, 2019, **8**, 565.
156. H. Boukhebti, A. N. Chaker, T. Lograda and M. Ramdani, *Int J Pharmacol Toxicol*, 2015, **5**, 42-46.
157. B. Shasha and J. Leibowitz, *The Journal of Organic Chemistry*, 1961, **26**, 1948-1954.
158. M. Perez-Bonilla, S. Salido, T. A. van Beek and J. Altarejos, *J. Agric. Food Chem.*, 2014, **62**, 144-151.
159. Z. J. Suarez Montenegro, G. Alvarez-Rivera, J. D. Sanchez-Martinez, R. Gallego, A. Valdes, M. Bueno, A. Cifuentes and E. Ibanez, *Foods*, 2021, **10**, 1507.
160. E. Goicoechea-Oses and A. Ruiz-Aracama, *Foods*, 2024, **13**, 2298.
161. S. Parri, G. Cai, M. Romi, C. Cantini, D. Pinto, A. M. S. Silva and M. C. P. Dias, *Front Plant Sci*, 2024, **15**, 1408731.
162. P. Luaces, J. Exposito, P. Benabal, M. Pascual, C. Sanz and A. G. Perez, *Antioxidants (Basel)*, 2023, **13**, 12.
163. F. Dionisi, J. Prodolliet and E. Tagliaferri, *Journal of the American Oil Chemists' Society*, 1995, **72**, 1505-1511.
164. S. C. Cunha, J. S. Amaral, J. O. Fernandes and M. B. P. P. Oliveira, *J. Agric. Food Chem.*, 2006, **54**, 3351-3356.
165. C. Jimenez-Lopez, M. Carpena, C. Lourenco-Lopes, M. Gallardo-Gomez, J. M. Lorenzo, F. J. Barba, M. A. Prieto and J. Simal-Gandara, *Foods*, 2020, **9**, 1014.
166. F. Gelmini, M. Ruscica, C. Macchi, V. Bianchi, R. Maffei Facino, G. Beretta and P. Magni, *Planta Med.*, 2016, **82**, 273-278.
167. R. Sacchi, F. Addeo and L. Paolillo, *Magn. Reson. Chem.*, 1997, **35**, S133-S145.
168. N. Stiti, S. Triki and M. A. Hartmann, *Lipids*, 2007, **42**, 55-67.
169. H. Saimaru, Y. Orihara, P. Tansakul, Y. H. Kang, M. Shibuya and Y. Ebizuka, *Chem. Pharm. Bull. (Tokyo)*, 2007, **55**, 784-788.
170. G. Vlahov, G. Rinaldi, P. Del Re and A. A. Giuliani, *Anal. Chim. Acta*, 2008, **624**, 184-194.
171. R. Ghanbari, F. Anwar, K. M. Alkharfy, A. H. Gilani and N. Saari, *Int. J. Mol. Sci.*, 2012, **13**, 3291-3340.
172. E. Duquesnoy, V. Castola and J. Casanova, *Phytochemical Analysis: An International Journal of Plant Chemical and Biochemical Techniques*, 2007, **18**, 347-353.
173. A. Ali, F. Ahmadi, J. J. Cottrell and F. R. Dunshea, *Separations*, 2023, **10**, 354.
174. FooDB, FooDB: The Food Database, https://foodb.ca, (accessed March 8, 2024).
175. T. Li, W. Wu, J. Zhang, Q. Wu, S. Zhu, E. Niu, S. Wang, C. Jiang, D. Liu and C. Zhang, *Antioxidants (Basel)*, 2023, **12**, 2033.
176. N. Stiti and M. A. Hartmann, *J Lipids*, 2012, **2012**, 476595.
177. E. E. Rufino-Palomares, A. Perez-Jimenez, L. Garcia-Salguero, K. Mokhtari, F. J. Reyes-Zurita, J. Peragon-Sanchez and J. A. Lupianez, *Molecules*, 2022, **27**, 2341.
178. H. Mushtaq, S. Piccolella, J. A. Mendiola, L. Montero, E. Ibáñez and S. Pacifico, *Foods*, 2025, **14**, 297.
179. F. Sakouhi, C. Absalon, K. Sebei, E. Fouquet, S. Boukhchina and H. Kallel, *Food Chem.*, 2009, **116**, 345-350.
180. G. Gunduz and D. B. Konuskan, *J Oleo Sci*, 2023, **72**, 79-85.
181. R. M. Alonso-Salces, M. V. Holland, C. Guillou and K. Héberger, in *Olive Oil-Constituents, Quality, Health Properties and Bioconversions*, IntechOpen, 2012.
182. J. M. Romero-Marquez, M. D. Navarro-Hortal, T. Y. Forbes-Hernandez, A. Varela-Lopez, J. G. Puentes, R. D. Pino-Garcia, C. Sanchez-Gonzalez, I. Elio, M. Battino, R. Garcia, S. Sanchez and J. L. Quiles, *Antioxidants (Basel)*, 2023, **12**, 1538.
183. M. Williams, M. T. Morales, R. Aparicio and J. L. Harwood, *Phytochemistry*, 1998, **47**, 1253-1259.
184. L. Cecchi, M. Migliorini, E. Giambanelli, A. Cane, N. Mulinacci and B. Zanoni, *J. Agric. Food Chem.*, 2021, **69**, 5155-5166.
185. O. Ameixa, M. Pinho, M. R. Domingues and A. I. Lillebo, *PLoS One*, 2023, **18**, e0287986.
186. C. Aydin, M. M. Özcan and T. Gümüş, *Int. J. Food Sci. Nutr.*, 2009, **60**, 365-373.
187. W. Ahmad, N. Ali, M. S. Afridi, H. Rahman, M. Adnan, N. Ullah, U. Muhammad, M. Ilyas and H. Khan, *Pure and Applied Biology (PAB)*, 2017, **6**, 337-345.
188. S. M. Wabaidur, A. AlAmmari, A. Aqel, S. A. Al-Tamrah, Z. A. Alothman and A. Y. B. H. Ahmed, *J. Chromatogr. B*, 2016, **1031**, 109-115.
189. D. M. Ferreira, N. M. de Oliveira, M. H. Cheu, D. Meireles, L. Lopes, M. B. Oliveira and J. Machado, *Plants (Basel)*, 2023, **12**, 688.
190. M. Martínez, M. Fuentes, N. Franco, J. Sánchez and C. de Miguel, *Journal of the American Oil Chemists' Society*, 2014, **91**, 1921-1929.

191. G. Vlahov, C. Schiavone and N. Simone, *Lipid/Fett*, 1999, **101**, 146-150.
192. A. Korkmaz, *Molecules*, 2023, **28**, 1483.
193. T. S. Alnusaire, A. M. Sayed, A. H. Elmaidomy, M. M. Al-Sanea, S. Albogami, M. Albqmi, B. F. Alowaiesh, E. M. Mostafa, A. Musa, K. A. Youssif, H. Refaat, E. M. Othman, T. Dandekar, E. Alaaeldin, M. M. Ghoneim and U. R. Abdelmohsen, *Antioxidants (Basel)*, 2021, **10**, 1860.
194. M. Kalkan, A. Aygan, N. Çömlekçioglu and U. Çömlekçioğlu, 2023.
195. D. Kmiecik, M. Fedko, J. Malecka, A. Siger and P. L. Kowalczewski, *Molecules*, 2023, **28**, 4247.
196. H. S. Omar, S. N. Abd El-Rahman, S. M. AlGhannam, N. E.-H. A. Reyad and M. S. Sedeek, *Molecules*, 2021, **26**, 6118.
197. D. Ollivier, J. Artaud, C. Pinatel, J. P. Durbec and M. Guerere, *J. Agric. Food Chem.*, 2003, **51**, 5723-5731.
198. E. Niu, W. Hu, J. Ding, W. Wang, A. Romero, G. Shen and S. Zhu, *Scientia Horticulturae*, 2022, **299**, 111017.
199. A. T. Uncu, A. O. Uncu, A. Frary and S. Doganlar, *Food Chem.*, 2017, **221**, 1026-1033.
200. L. Mannina, G. Fontanazza, M. Patumi, G. Ansanelli and A. Segre, *Grasas y Aceites*, 2001, **52**, 380-388.
201. M. L. Hernández, M. D. Sicardo, A. Belaj and J. M. Martínez-Rivas, *Frontiers in Plant Science*, 2021, **12**, 653997.
202. G. Schneider and W. Kleinert, 1972.
203. A. López-López, A. Cortés-Delgado, A. de Castro, A. H. Sánchez and A. Montaño, *Food research international*, 2019, **125**, 108568.
204. A. Boudebouz, A. Romero, R. Boqué, L. Aceña, O. Busto and M. Mestres, *J. Sci. Food Agric.*, 2020, **100**, 3173-3181.
205. A. Bianco, R. L. Scalzo and M. L. Scarpati, *Phytochemistry*, 1993, **32**, 455-457.
206. E. López-Huertas, J. Rubí-Villegas, L. Sánchez-Moreno and R. Nieto, *Int. J. Mol. Sci.*, 2024, **25**, 3962.
207. A. López, A. Garrido and A. Montaño, *Italian Journal of Food Science/Rivista Italiana di Scienza degli Alimenti*, 2007, **19**.
208. A. G. Manoukas, B. Mazomenos and M. A. Patrinou, *J. Agric. Food Chem.*, 1973, **21**, 215-217.
209. E. H. Ibrahim, M. A. Abdelgaleel, A. A. Salama and S. M. Metwalli, *J. Agric. Res. Kafr. El-Sheikh Univ*, 2016, **42**, 445-459.
210. L. Cecchi, M. Migliorini, E. Giambanelli, V. Canuti, M. Bellumori, N. Mulinacci and B. Zanoni, *J. Sci. Food Agric.*, 2022, **102**, 2515-2525.
211. S. Kesen, H. Kelebek, K. Sen, M. Ulas and S. Selli, *Food Research International*, 2013, **54**, 1987-1994.
212. S. Albogami and A. M. Hassan, *Molecules*, 2021, **26**, 4069.
213. I. D'Antuono, V. G. Kontogianni, K. Kotsiou, V. Linsalata, A. F. Logrieco, M. Tasioula-Margari and A. Cardinali, *Food Research International*, 2014, **65**, 301-310.
